# Enantioselective [4+2] Annulation to the Concise Synthesis of Chiral Dihydrocarbazoles

**DOI:** 10.1016/j.isci.2020.100840

**Published:** 2020-01-17

**Authors:** Haiyang Wang, Qingdong Hu, Mingxu Wang, Chang Guo

**Affiliations:** 1 Hefei National Laboratory for Physical Sciences at the Microscale, University of Science and Technology of China, Hefei 230026, China

**Keywords:** Catalysis, Organic Synthesis, Organic Reaction

## Abstract

A highly efficient phosphine-catalyzed enantioselective [4 + 2] annulation of allenoates with 3-nitroindoles or 3-nitrobenzothiophenes has been developed. The protocol represents a unique dearomatization–aromatization process to access functionalized dihydrocarbazoles or dihydrodibenzothiophenes with high optical purity (up to 97% ee) under mild reaction conditions. The synthetic utility of the highly enantioselective [4 + 2] annulation enables a concise synthesis of analgesic agent.

## Highlights

• High regio-, chemo-, and enantioselectivity
• Broad substrate scope
• Dearomatization/aromatization steps proceed under mild conditions
• Concise synthesis of chiral analgesic agent

## Graphical Abstract

**Figure:**
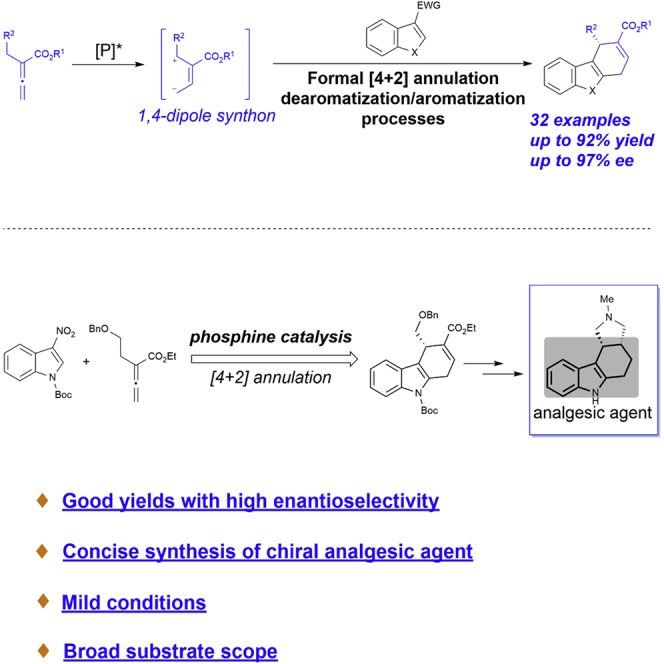

## Introduction

**Scheme 1:**
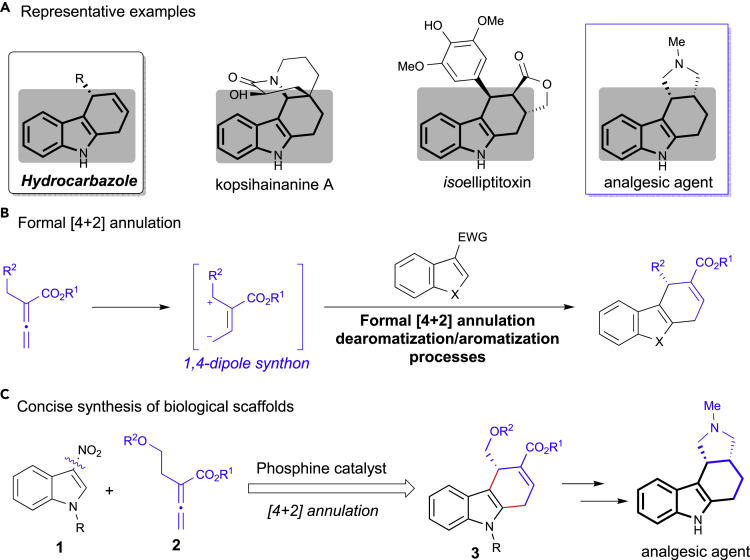
Phosphine-catalyzed [4 + 2] Dearomatization/Aromatization Reactions for the Formation of Enantioenriched Heterocycles (A) Representative examples of chiral hydrocarbazole derivatives. (B) Formal [4 + 2] annulation for the preparation of hydrocarbazole. (C) Concise approach to the enantioselective synthesis of analgesic agent.

Fused polycyclic indoles are common structural motifs found in a vast array of natural and biologically active molecules (Saxton, 1996, Knölker and Reddy, 2002, Schmidt et al., 2012, Tan and Cheng, 2019), such as kopsihainanine A, isoelliptitoxin, and analgesic agents (Scheme 1A) (Madalengoitia and Macdonald, 1993, Carmosin et al., 2000, Chen et al., 2011). In this regard, the development of efficient methods for enantioselective construction of hydrocarbazole skeleton is still highly demanded (Sings et al., 2001, Lu et al., 2012, Zhou et al., 2015, Gu et al., 2016). The group of Jørgensen disclosed a novel [4 + 2] annulation by trienamine catalysis, thus obtaining dihydrocarbazoles in good yields and enantioselectivities (Li et al., 2016b). In this context, we hypothesized that the development of new methods through the enantioselective phosphine-catalyzed [4 + 2] dearomatization would provide practical and efficient approach to this class of enantioenriched heterocycles (Scheme 1B).

Phosphine catalysis has been recognized as a reliable tool for the development of unique transformations of allenoates, allowing for the discovery of novel asymmetric synthetic methodology (Lu et al., 2001, Methot and Roush, 2004, Ye et al., 2008, Cowen and Miller, 2009, Wei and Shi, 2010, Wei and Shi, 2017, Fan and Kwon, 2013, Wang et al., 2014, Wang et al., 2016, López and Mascareñas, 2014, Xie and Huang, 2015, Li and Zhang, 2016a, Li and Lu, 2017, Ni et al., 2018, Guo et al., 2018). Considerable research efforts have been devoted to the development of new methods for the phosphine-catalyzed enantioselective reactions. The use of phosphine catalysts has introduced a set of elementary steps that operate via discrete reactive species, allowing access to natural products and pharmaceuticals (Tran and Kwon, 2005, Andrews and Kwon, 2012, Han et al., 2012, Wang and Krische, 2003, Cai et al., 2016). One particularly versatile and reactive species is the phosphine-mediated 1,4-dipole generated upon addition of the phosphine catalyst to an allenoate substrate, thus providing a concise approach for accessing enantioselective annulations. Specially, Kwon group reported the result of their pioneering studies toward the development of a novel [4 + 2] annulation reaction of allenoates and *N*-tosylimines in the presence of phosphine catalyst (Zhu et al., 2003). Later, Fu group reported the phosphine-catalyzed highly enantioselective [4 + 2] annulation of *N*-tosylimines with allenoates (Wurz and Fu, 2005). Although great achievements have been made, concise syntheses of useful heterocycles involving phosphine-catalyzed [4 + 2] annulations in asymmetric versions were still rare (Tran and Kwon, 2007, Wang and Ye, 2010, Tran et al., 2011, Xiao et al., 2011, Baskar et al., 2011, Yu and Ma, 2012, Zhong et al., 2012, Takizawa et al., 2014, Yu et al., 2014, Liu et al., 2016, Wang and Guo, 2019a). Moreover, the development of an enantioselective phosphine-catalyzed [4 + 2] dearomatization reaction would provide an attractive and complementary approach for construction of privileged motifs, which will be of great value for the synthesis of bioactive molecules (Scheme 1C).

Enantioselective dearomatization reactions of heteroaromatic compounds are very powerful transformations because they provide direct access to a wide variety of chiral heterocycles (You, 2016, Roche and Porco, 2011, Zhuo et al., 2012, Zhuo et al., 2014, Zheng and You, 2016, Sun et al., 2016, Wu et al., 2016). In recent years, 3-nitroindole was demonstrated to be a good substrate for various dearomatization processes, and a number of enantioselective approaches have been reported (Awata and Arai, 2014, Zhao et al., 2015a, Zhao et al., 2015b, Zhao et al., 2018, Zhao et al., 2019, Gerten and Stanley, 2016, Trost et al., 2014, Cheng et al., 2018, Zhang et al., 2018, Sun et al., 2018, Yue et al., 2017, Yang et al., 2019). Importantly, Lu group (Li et al., 2019) and Zhang group (Wang et al., 2019b) independently reported the efficient phosphine-catalyzed enantioselective [3 + 2] annulation of 3-nitroindoles with allenoates to afford cyclopentaindoline products in high yields and excellent enantioselectivities. We envisaged that heteroaromatic systems bearing an electron-withdrawing group could react with phosphine-mediated zwitterionic intermediate in a process involving the [4 + 2] reaction to achieve the chiral dihydrocarbazole scaffold (Scheme 1C). With this objective in mind, a readily available 3-nitroindole derivative was selected as a model substrate to investigate the optimum reaction condition for the enantioselective [4 + 2] dearomatization reaction using a phosphine catalyst.

## Results and Discussion

Based on our previous work on phosphine chemistry (Wang and Guo, 2019a), we initiated the study by investigating the reaction between **1a** and **2a** in the presence of the phosphine **4a** (Table 1, entry 1). Initially, diverse chiral phosphine catalysts were examined (entries 1–5). However, the catalyst **4a** to **4c** did not work for this reaction (entries 1–3). To our great delight, the desired dihydrocarbozole**3a** could be obtained when the chiral phosphine **4d** was employed (entry 4). After surveying an array of additives, we determined that silica gel can promote elimination of HNO_2_ for the aromatization process to afford the corresponding adduct in 92% yield with 94% ee (entry 4) (So and Mattson, 2012, Long et al., 2016). Other additives, such as Sc(OTf)_3_, Et_3_N, and SnCl_2_ led to byproducts (for further details, see Table S1 in the Supplemental Information). Furthermore, the ee values of the **3a** decreased to 80% with low yield in the presence of **4e** as catalyst (entry 5). Varying the solvents led to no improvement in the reaction, and toluene was proven to be the best choice (entries 4 vs 6–8). Further optimization studies revealed that the protection group of the 3-nitroindole was also sensitive to the reaction, and the variation of the *N*-substituent of the 3-nitroindole **1a′** or **1a’’** generated no product at all (entries 9 and 10) (Rivinoja et al., 2017, Suo et al., 2018).

**Table 1:** Optimization of Reaction Conditions


Entry	1	4	Solvent	Yield (%)^a^	ee (%)^b^
1	**1a**	**4a**	Toluene	nr	–
2	**1a**	**4b**	Toluene	nr	–
3	**1a**	**4c**	Toluene	nr	–
**4**	**1a**	**4d**	**Toluene**	**92**	**94**
5	**1a**	**4e**	Toluene	26	80
6	**1a**	**4d**	CH_2_Cl_2_	23	73
7	**1a**	**4d**	THF	73	84
8	**1a**	**4d**	Dioxane	57	90
9	**1a′**	**4d**	Toluene	nr	–
10	**1a’’**	**4d**	Toluene	nr	–

Unless indicated otherwise, the reaction were conducted with **1** (0.1 mmol), **2a** (0.15 mmol), and **4** (0.01 mmol) in toluene (1.0 mL) at room temperature for 18 h. Then silica gel (200 mg) was added to the reaction mixture to complete elimination of HNO_2_. nr = no reaction.

a Yield of isolated product.

b Determined by HPLC analysis.

**Scheme 2:**
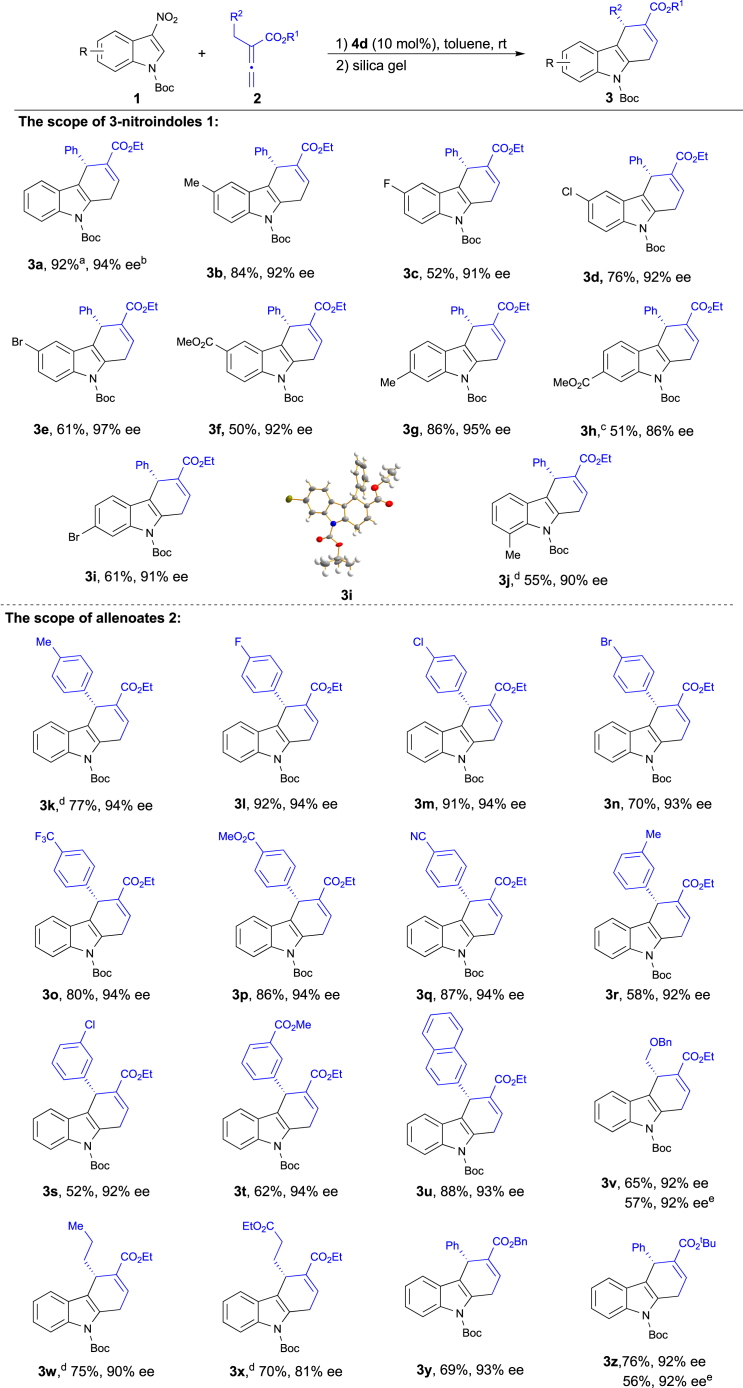
Substrate Scope of Enantioselective [4 + 2] Annulation Unless indicated otherwise, the reactions were conducted with **1** (0.1 mmol), **2** (0.15 mmol), and catalyst **4d** (0.01 mmol) in toluene at room temperature for 12–48 h. Then silica gel was added to the reaction mixture to complete elimination of HNO_2_. ^a^Yield of the isolated product after purification by chromatography on silica gel. ^b^Enantiomeric excess determined by HPLC analysis. ^c^Aromatization process was performed at 50°C. ^d^20 mol% of **4d**. ^e^The reaction was performed on 1 mmol scale.

With the optimal reaction conditions in hand, we set out to explore the substrate scope of the procedure. As shown in Scheme 2, various electron-withdrawing or donating groups on the indole ring were well tolerated and resulted in excellent levels of enantioselectivities ranging from 86% to 97% ee (**3a**–**3j**). The extension of the protocol to the 3-nitroindole with a variety of substitution patterns at the 5-position was successful to afford corresponding adducts with excellent enantioselectivities (**3b**–**3f**). To our delight, substrates bearing substituents on different positions of the indole ring also facilitate the annulation with high yields and ee values (**3b**, **3g**, and **3j**). The absolute configuration of the enantiopure **3i**, recrystallized from ethyl acetate and petroleum ether, was assigned by single-crystal X-ray diffraction analysis.

The generality of the reaction with respect to the scope of the allenoates **2** was also investigated using 3-nitroindole **1a** as the reaction partner under the optimized conditions. A diverse array of allenoates (**2**) with a variety of functional groups (methyl, fluoro, chloro, bromo, ester, trifluoromethyl, and cyano) performed well in this annulation reaction, and the corresponding products were isolated in good yields with high ee values (**3k**-**3q**). Remarkably, this method was compatible with alkyl allenoate, affording the desired products in good yields with good enantioselectivity (**3v**-**3x**). Additionally, all reactions with different esters attached to the allenoates proceeded smoothly, giving the corresponding products in good yields and excellent ee (**3y** and **3z**). To test the synthetic utility of the current annulation, we performed the reaction on a 1 mmol scale with the formation of **3z** in 56% yield and 92% ee.

**Scheme 3:**
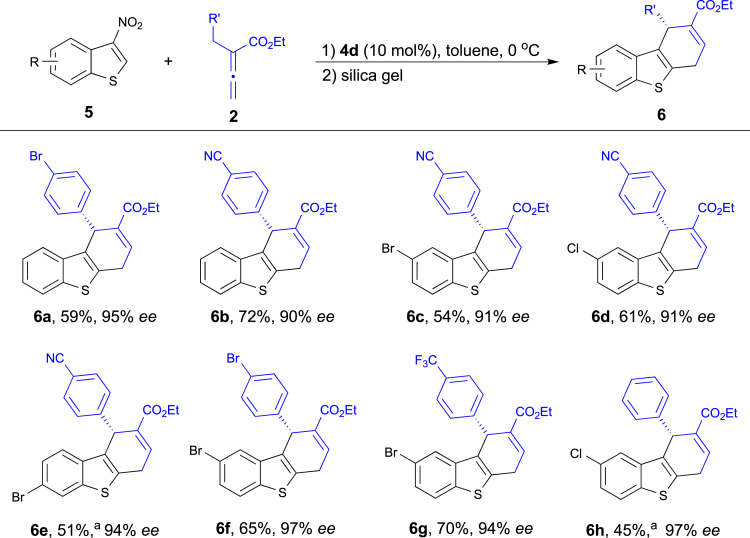
Enantioselective [4 + 2] Annulation of 3-Nitrobenzothiophene **5** Unless indicated otherwise, the reactions was conducted with **5** (0.1 mmol), **2** (0.15 mmol), and catalyst **4d** (0.01 mmol) in toluene (1.0 mL) at 0°C for 48–60 h. Then silica gel was added to the reaction mixture to complete elimination of HNO_2_. Yield of the isolated product after purification by chromatography on silica gel. Enantiomeric excess determined by HPLC analysis. ^a^0.02 mmol of **4d** was used.

Encouraged by the excellent results with various 3-nitroindoles, we then investigated the [4 + 2] annulation reaction with a range of 3-nitrobenzothiophenes (**5**). Remarkably, process where the 3-nitrobenzothiophene as a reactive partner for asymmetric annulation has been much less studied (Tran and Kwon, 2007, Wang and Ye, 2010, Tran et al., 2011, Xiao et al., 2011, Baskar et al., 2011, Yu and Ma, 2012, Zhong et al., 2012, Takizawa et al., 2014, Yu et al., 2014, Liu et al., 2016, Wang and Guo, 2019a, Cheng et al., 2000, Cheng et al., 2017, Suo et al., 2018, Yue et al., 2018, Chen et al., 2019). Using phosphine **4d** in toluene at 0°C, we were able to access dihydrodibenzothiophene products **6** (Scheme 3). Under the optimized reaction condition (for further details, see Table S2 in the Supplemental Information), a broad range of allenoates **2** and 3-nitrobenzothiophenes **5** were investigated. Allenoates with different substituents on the aromatic ring underwent this catalytic transformation smoothly in good yields with excellent ee (**6a** and **6b**). Furthermore, various substitutions of 3-nitrobenzothiophenes **5** at the aromatic ring had little impact on the reactions (**6c**–**6h**, 91%–97% ee).

**Scheme 4:**
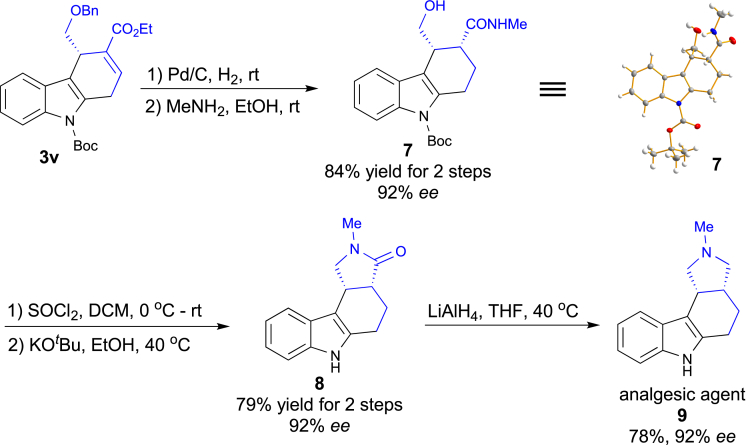
Enantioselective Synthesis of Analgesic Agent 9

To highlight the synthetic potential of the present method, the dihydrocabazole **3v**, which was obtained from the enantioselective [4 + 2] annulation, can be easily converted into analgesic agent **9** (Scheme 4). In 2000, Carmosin and co-workers obtained the racemic analgesic agent **9** via the Diels-Alder reaction, and the optical product was obtained by using preparative chromatography (Carmosin et al., 2000). Taking advantage of our current phosphine-catalyzed enantioselective [4 + 2] reaction, we can easily obtain the analgesic agent **9** with excellent enantioselectivity. Hydrogenation of **3v** in the presence of a catalytic amount of Pd/C, followed by amidation with MeNH_2_ gave rise to the desired amide **7** in 84% yield over two steps. The configuration of compound **7** was assigned by X-ray analysis. The subsequent chlorination of alcohol, deprotection of the N-Boc group and cyclization furnished **8** in good yield. Finally, the amide **8** was reduced to generate the corresponding analgesic agent **9** in 78% yield and 92% ee.

**Figure 1:**
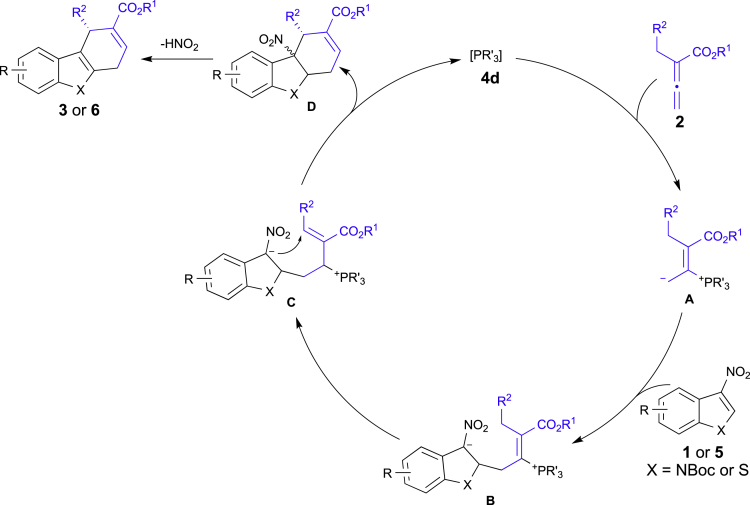
Proposed Mechanism

The proposed catalytic cycle for the enantioselective [4 + 2] annulation is illustrated in Figure 1. The addition of phosphine catalyst **4d** to the allenoate **2** gives the intermediate **A**, which could react with the 3-nitroindole **1** or 3-nitrobenzothiophenes **5** to form the intermediate **B**. Following migration and intramolecular conjugate addition give rise to the intermediate **D** and regenerate the phosphine **4d**. This species **D** then undergoes elimination of HNO_2_ through the aromatization process to furnish the final dihydrocarbzole **3** or dihydrodibenzothiophene **6**.

In summary, we have developed simple and efficient synthetic routes to highly enriched hydrocarbozoles through chiral phosphine-catalyzed [4 + 2] annulation utilizing 3-nitroindole and allenoates as starting materials. This phosphine-catalyzed enantioselective [4 + 2] annulation procedure involving tandem dearomatization and aromatization steps proceeds under mild conditions. This reaction displays a broad substrate scope with respect to the substituents. Additionally, the obtained dihydrocarbozole could be efficient transformed to an analgesic agent containing polycyclic indole frameworks.

### Limitations of the Study

The synthesis of the products needs two steps in one pot. No product was formed with the initial addition of silica gel.

## Methods

All methods can be found in the accompanying Transparent Methods supplemental file.

## Data and Code Availability

Crystallographic data for the structures reported in this article have been deposited at the Cambridge Crystallographic Data Center (CCDC) under accession numbers CCDC 1938371 (**3i**) and CCDC 1955757 (**7**). Copies of the data can be obtained free of charge from www.ccdc.cam.ac.uk/structures/.

## Supplemental Information

Document S1. Transparent Methods, Figures S1–S158, Schemes S1–S3, and Tables S1 and S2

## Footnotes

Supplemental Information can be found online at https://doi.org/10.1016/j.isci.2020.100840.
