# Supplementary material for: Enantioselective [4+2] Annulation to the Concise Synthesis of Chiral Dihydrocarbazoles
Source: iScience. 2020 Jan 17;23(2):100840. doi: 10.1016/j.isci.2020.100840 (PMC6995259; doi:10.1016/j.isci.2020.100840)
Supplement: Document S1. Transparent Methods, Figures S1–S158, Schemes S1–S3, and Tables S1 and S2 [file mmc1.pdf]

iScience, Volume *23*

Supplemental Information

Enantioselective [4+2] Annulation to the Concise Synthesis of Chiral Dihydrocarbazoles

Haiyang Wang, Qingdong Hu, Mingxu Wang, and Chang Guo

Supplemental Figures for NMR spectrums:

Figure S1. ^{1}H NMR spectrum of **3a**, related to **Scheme 2**.

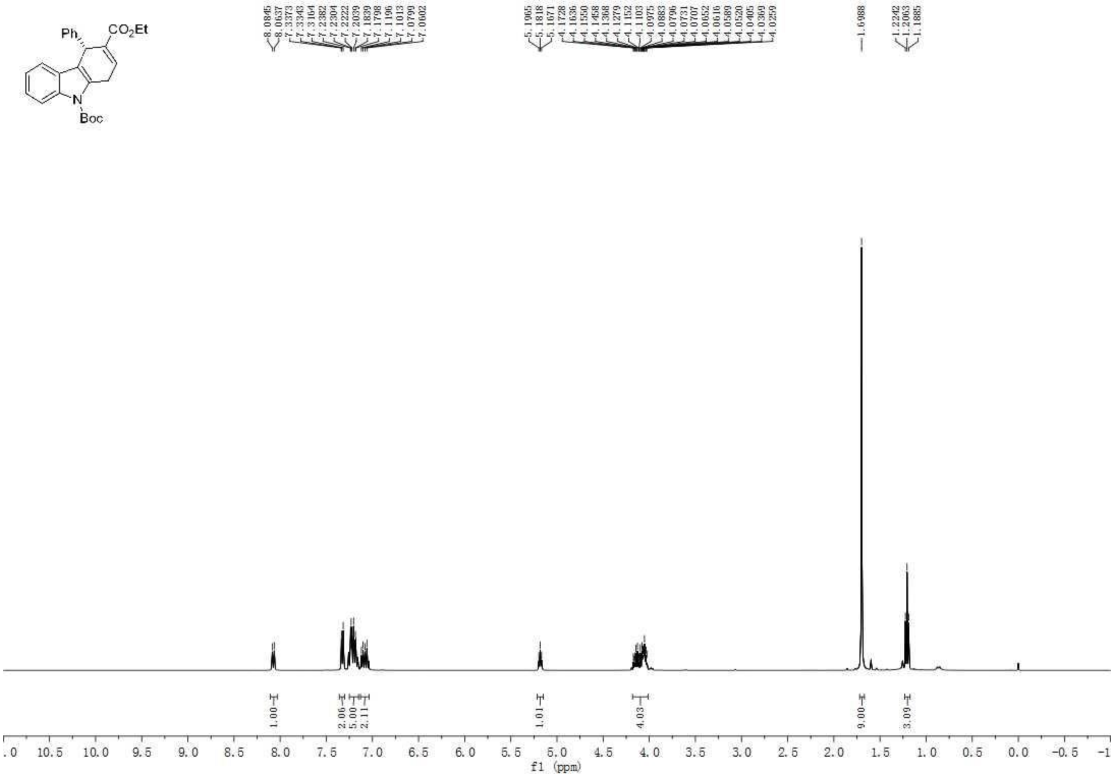

Figure S2. ^{13}C NMR spectrum of **3a**, related to **Scheme 2**.

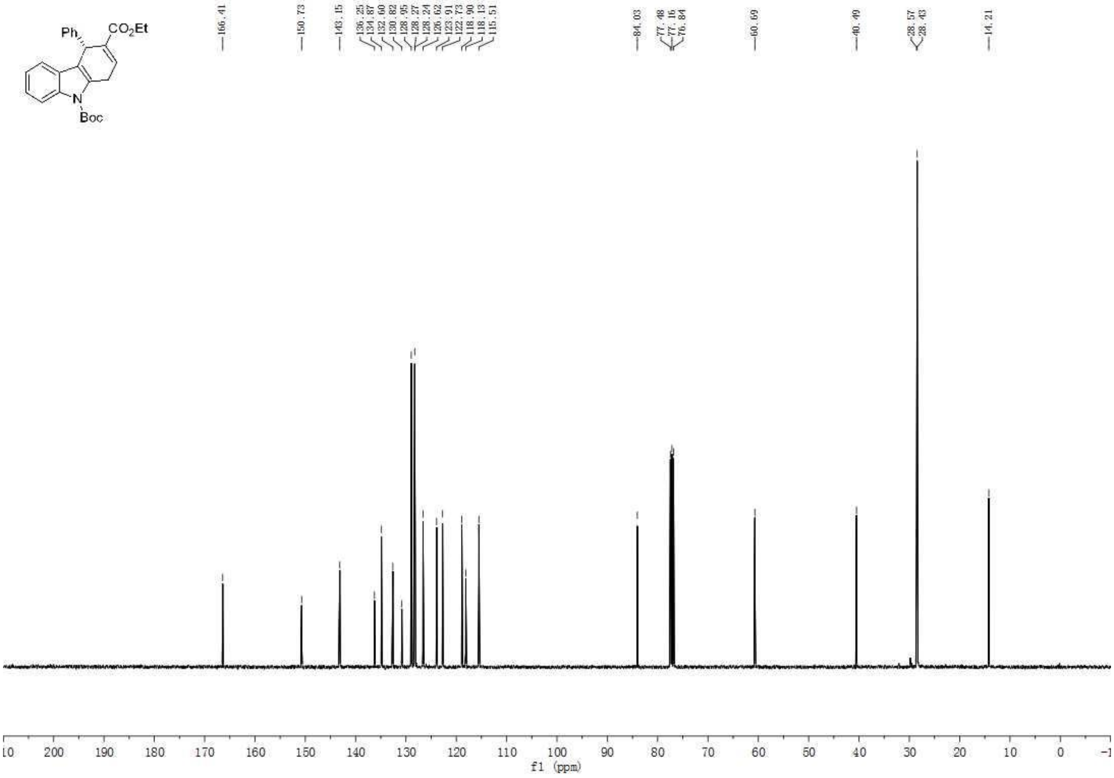

Figure S3. ^{1}H NMR spectrum of **3b**, related to **Scheme 2**.

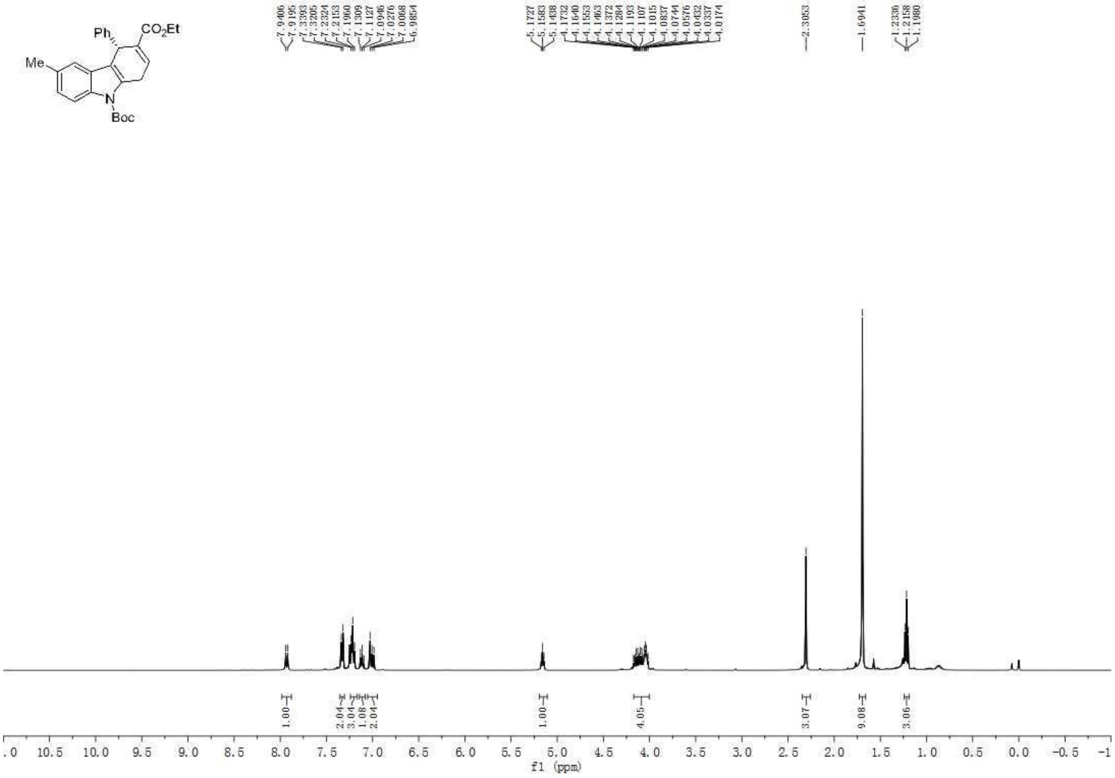

Figure S4. ^{13}C NMR spectrum of **3b**, related to **Scheme 2**.

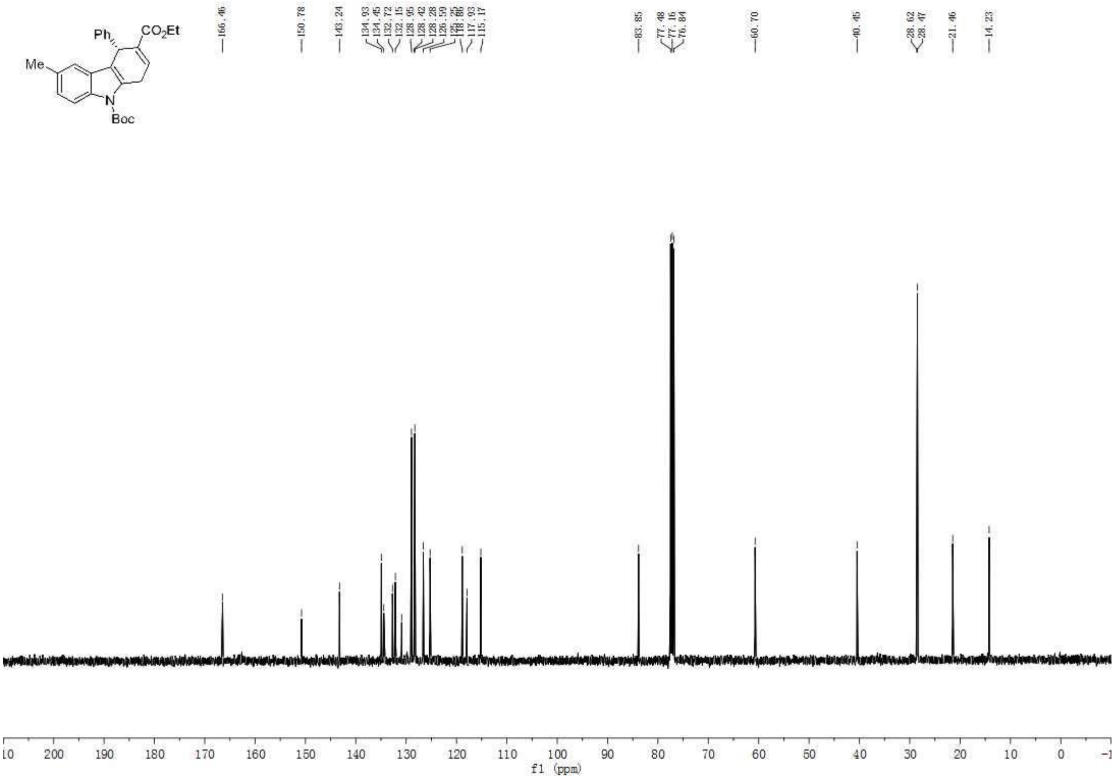

Figure S5. ^{1}H NMR spectrum of **3c**, related to **Scheme 2**.

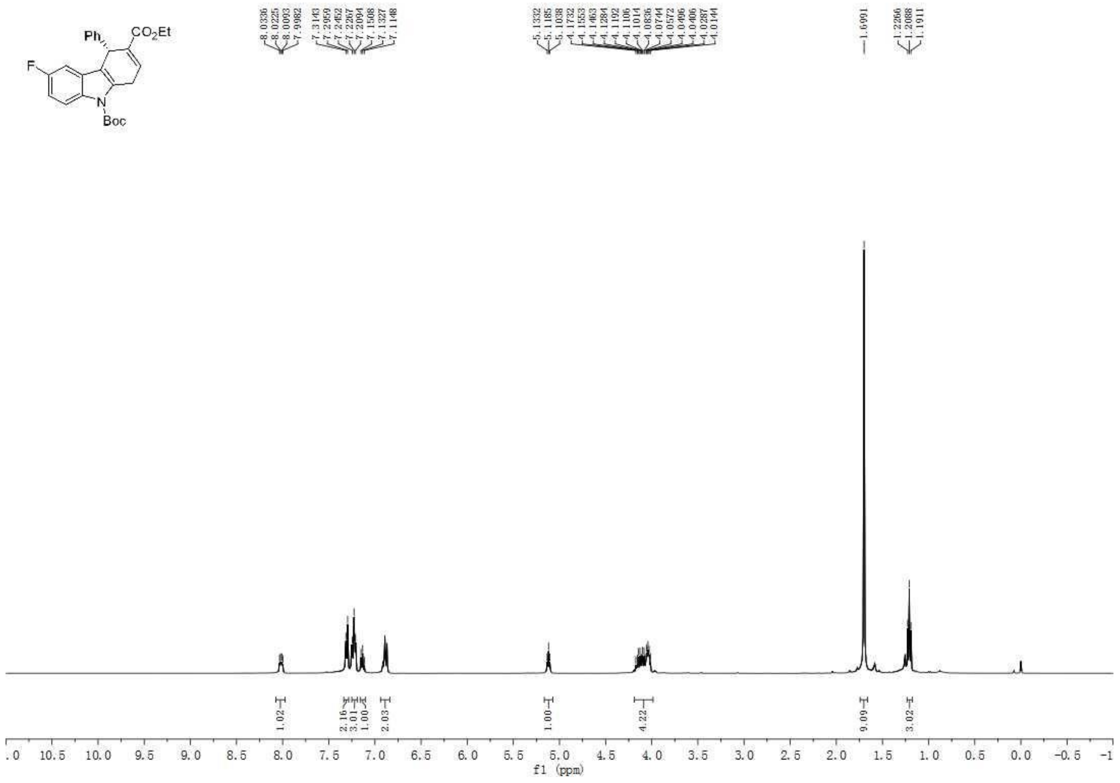

Figure S6. ^{13}C NMR spectrum of **3c**, related to **Scheme 2**.

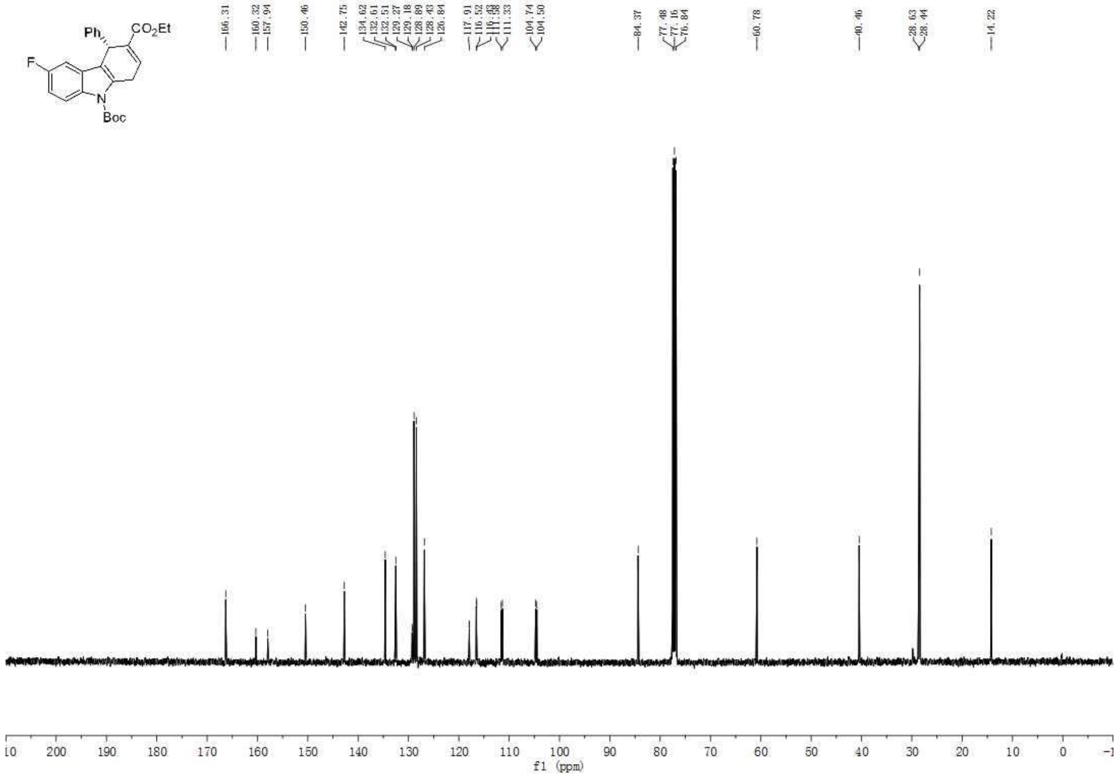

Figure S7. ^{19}F NMR spectrum of **3c**, related to **Scheme 2**.

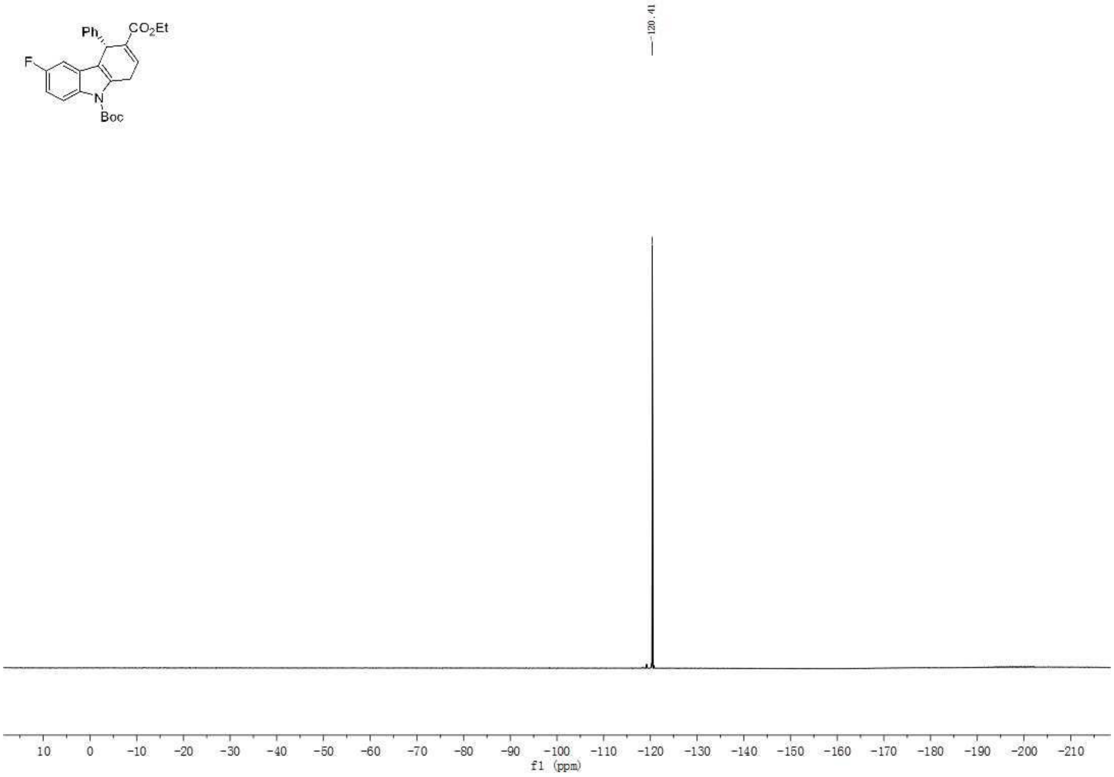

Figure S8. ^{1}H NMR spectrum of **3d**, related to **Scheme 2**.

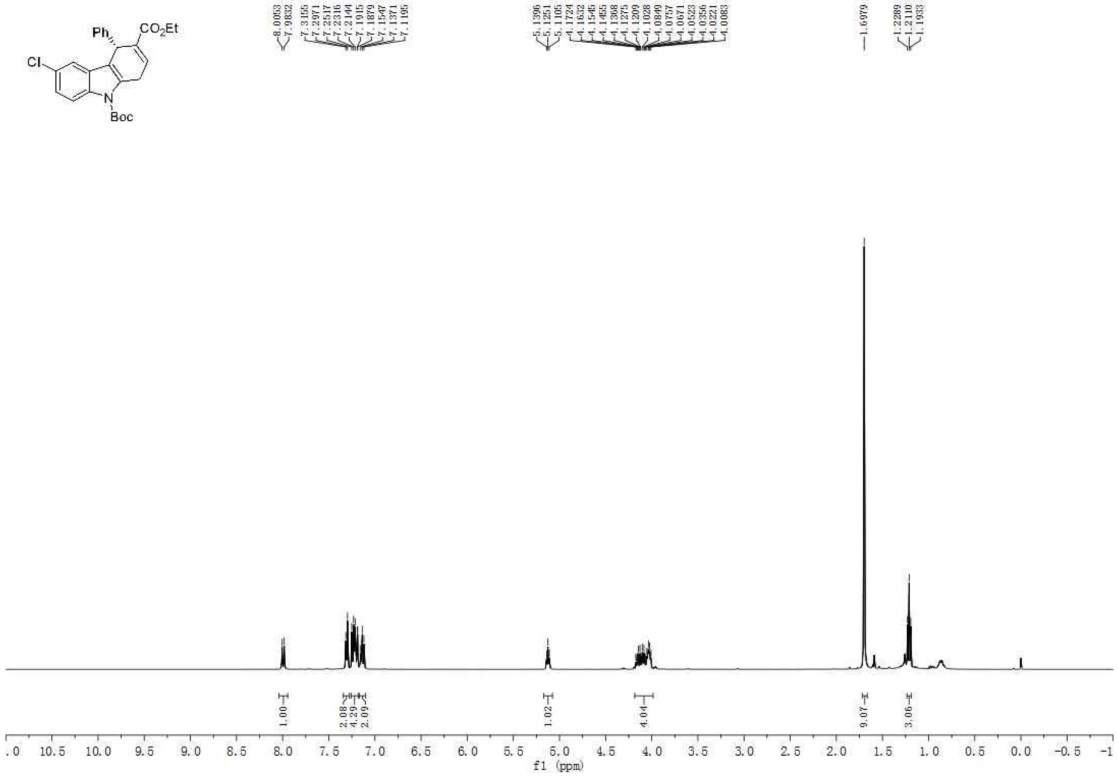

Figure S9. ^{13}C NMR spectrum of **3d**, related to **Scheme 2**.

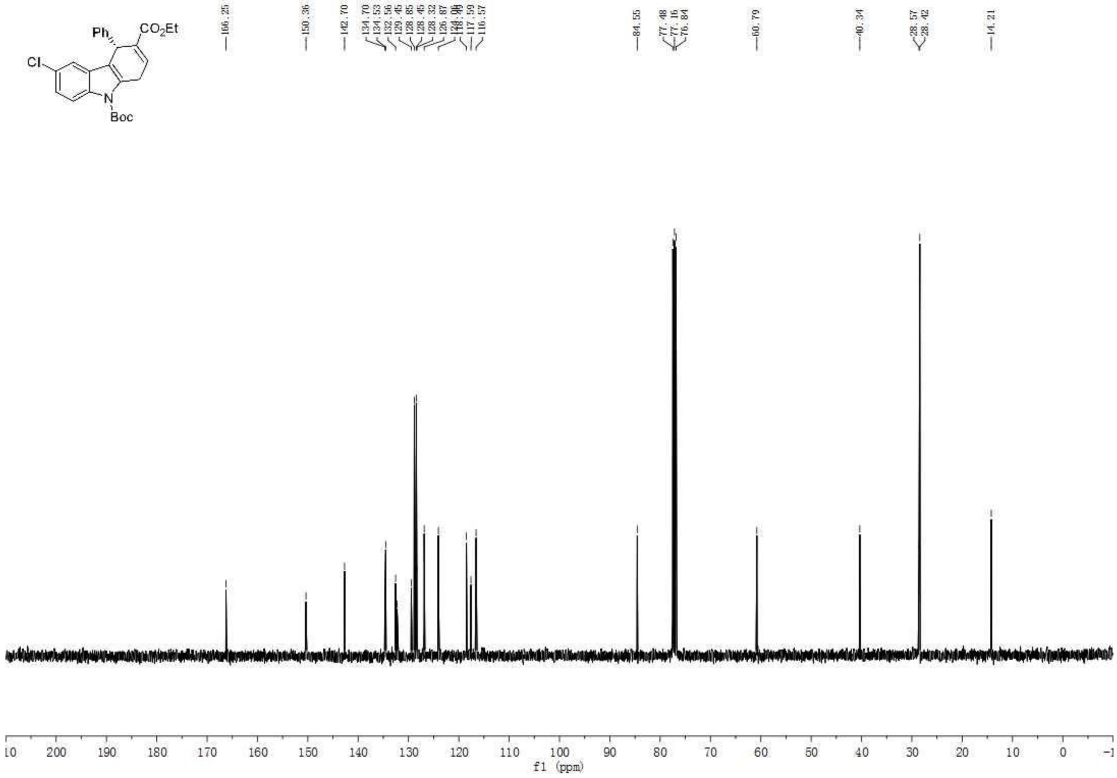

Figure S10. ^{1}H NMR spectrum of **3e**, related to **Scheme 2**.

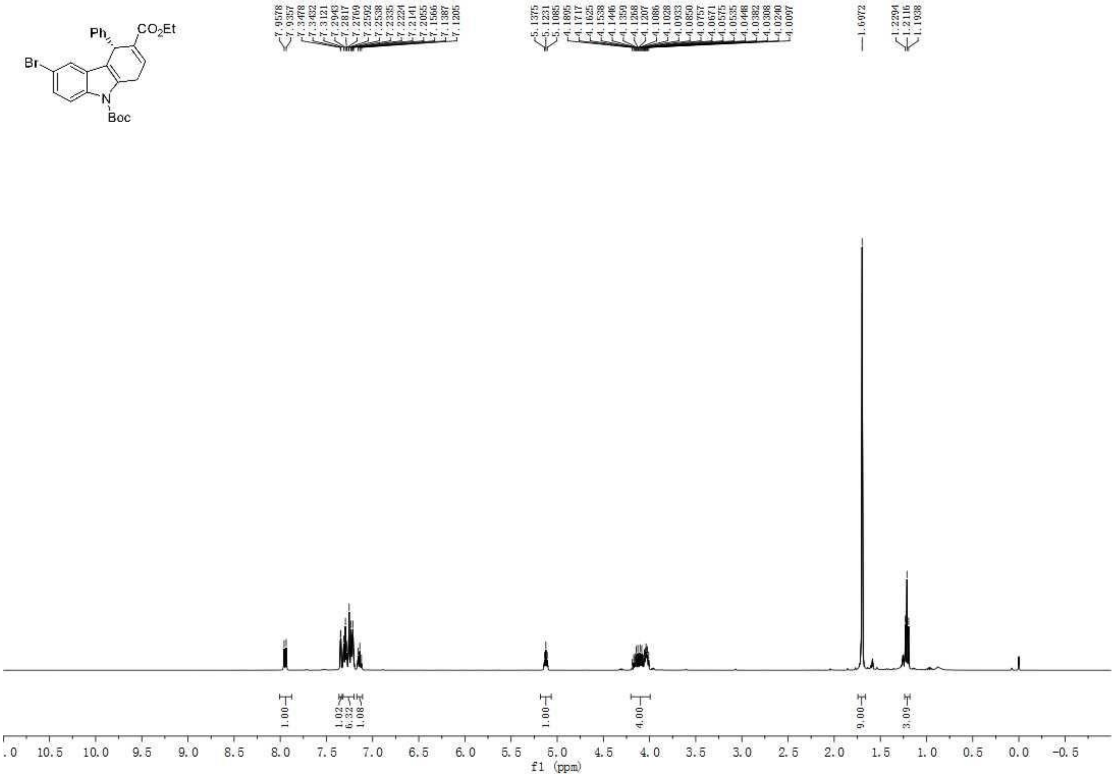

Figure S11. ^{13}C NMR spectrum of **3e**, related to **Scheme 2**.

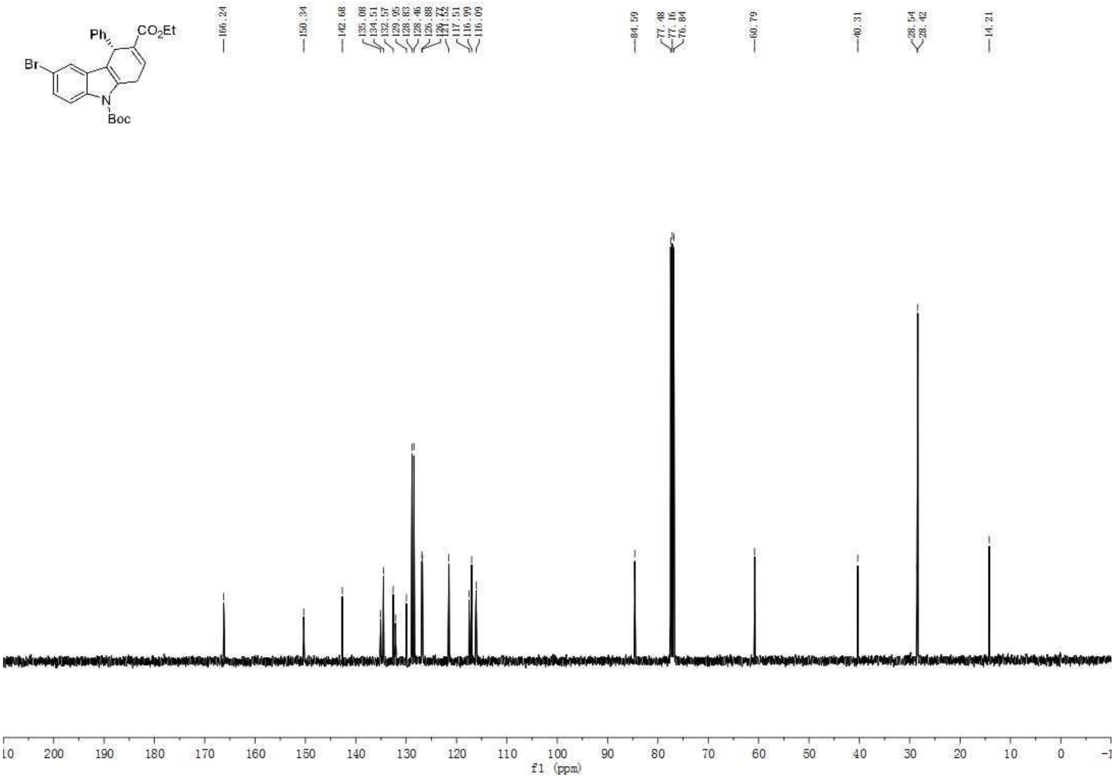

Figure S12. ^{1}H NMR spectrum of **3f**, related to **Scheme 2**.

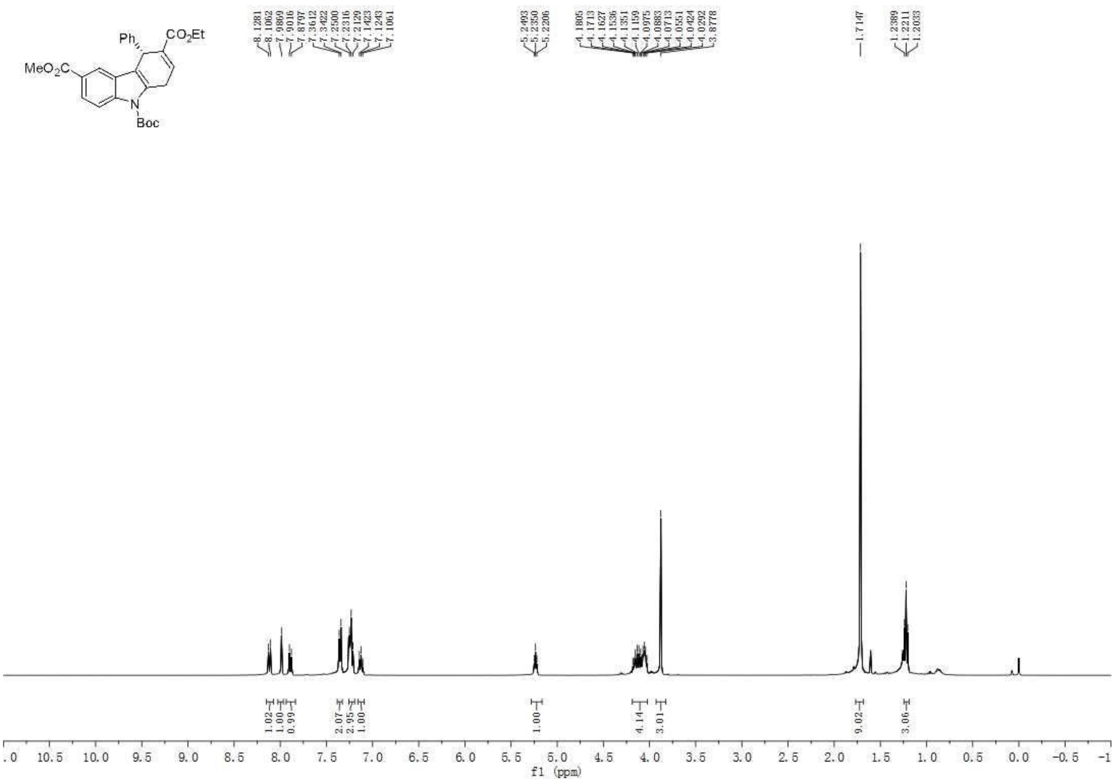

Figure S13. ^{13}C NMR spectrum of **3f**, related to **Scheme 2**.

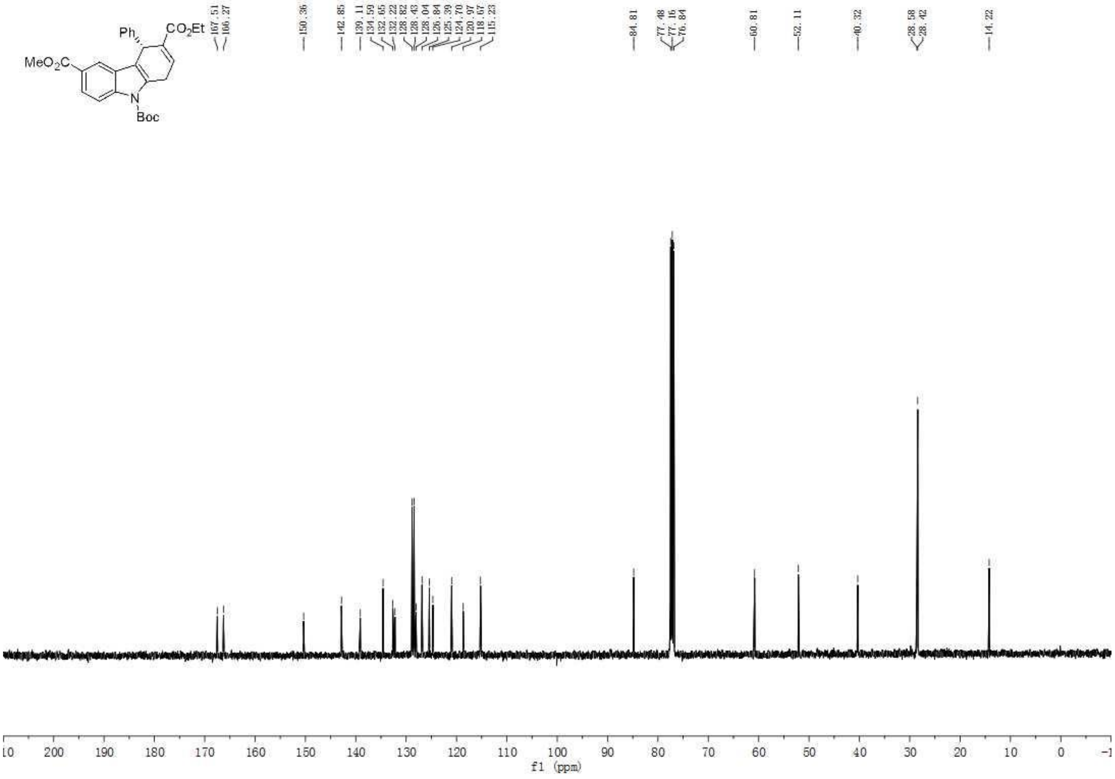

Figure S14. ^{1}H NMR spectrum of **3g**, related to **Scheme 2**.

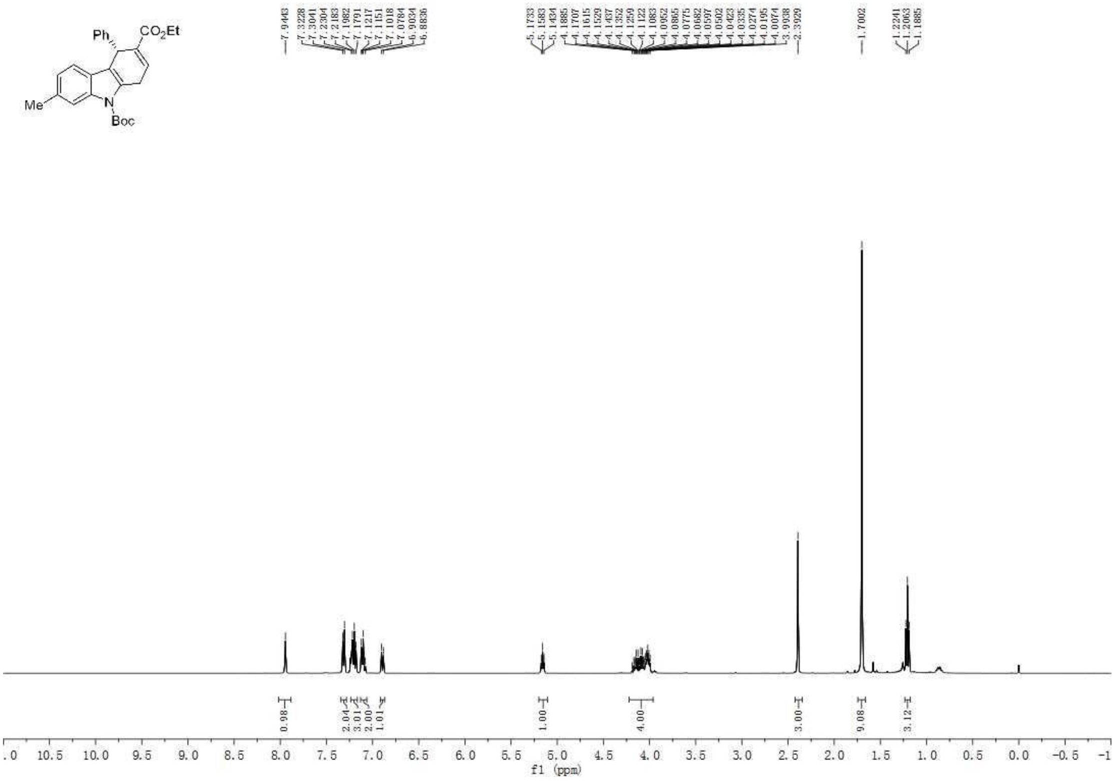

Figure S15. ^{13}C NMR spectrum of **3g**, related to **Scheme 2**.

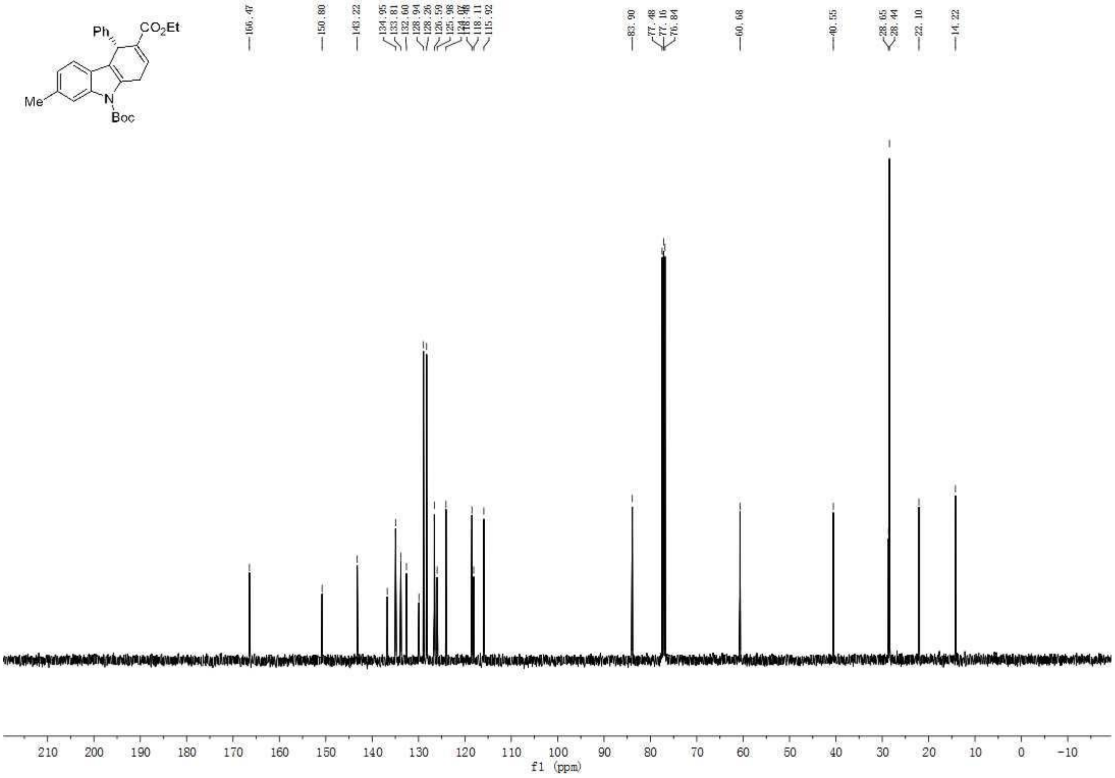

Figure S16. ^{1}H NMR spectrum of **3h**, related to **Scheme 2**.

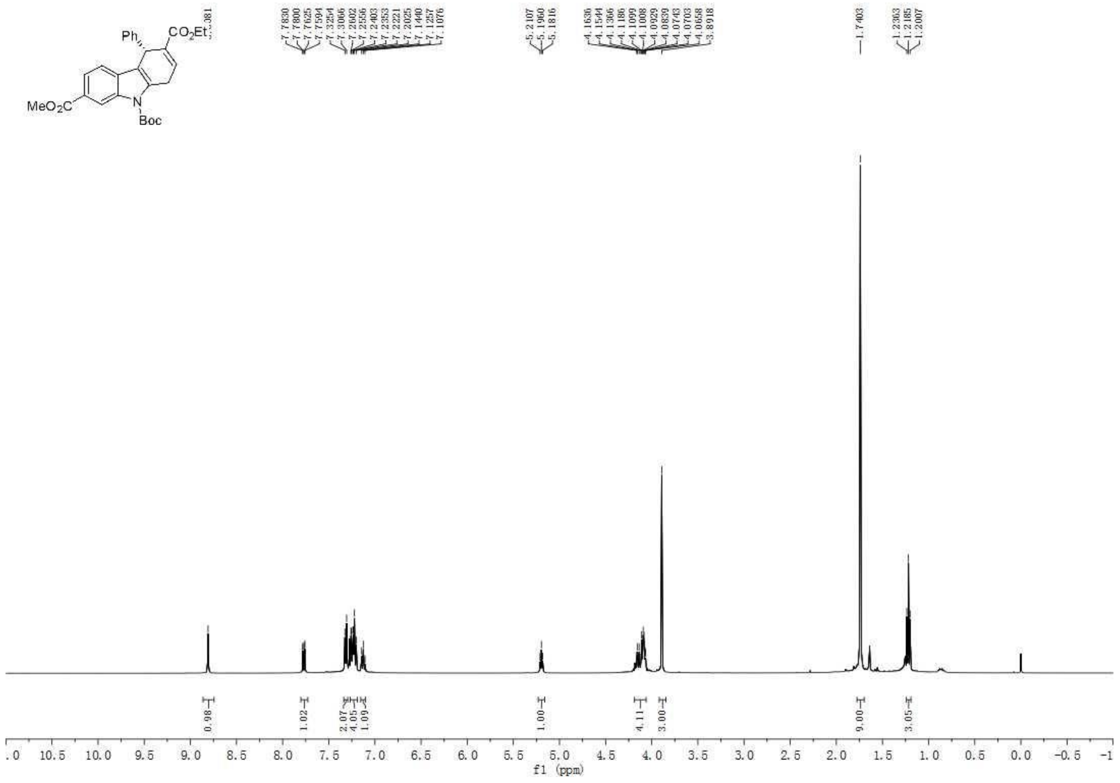

Figure S17. ^{13}C NMR spectrum of **3h**, related to **Scheme 2**.

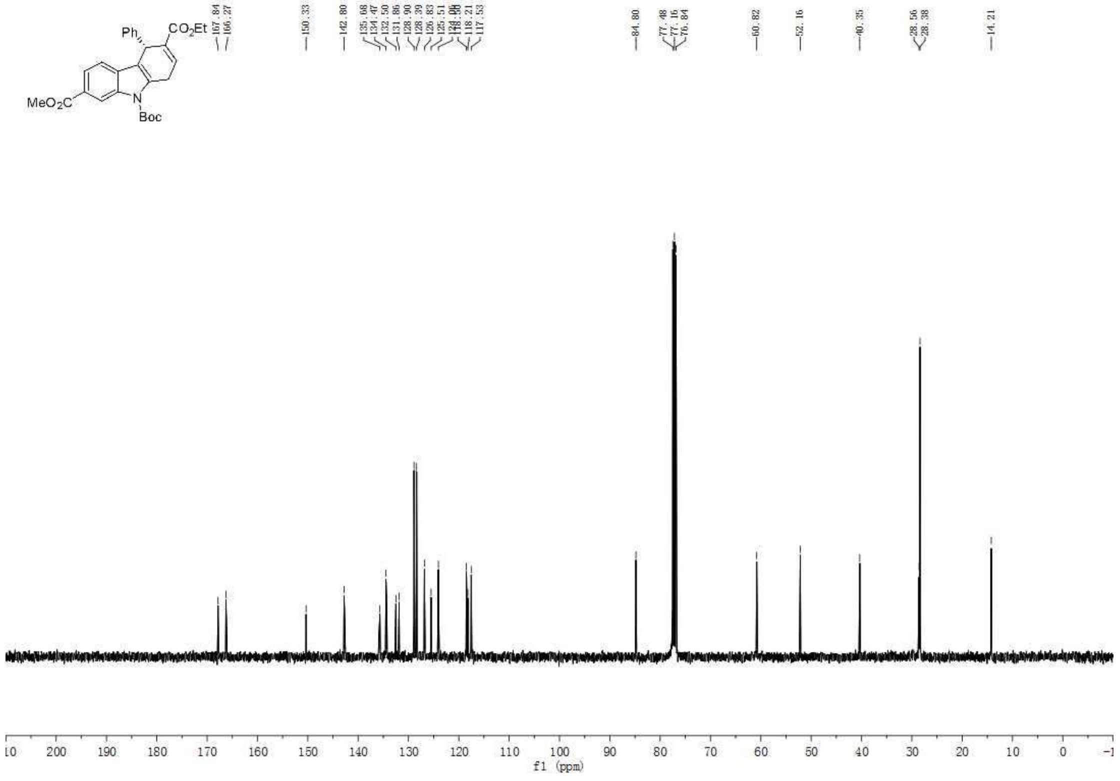

Figure S18. ^{1}H NMR spectrum of **3i**, related to **Scheme 2**.

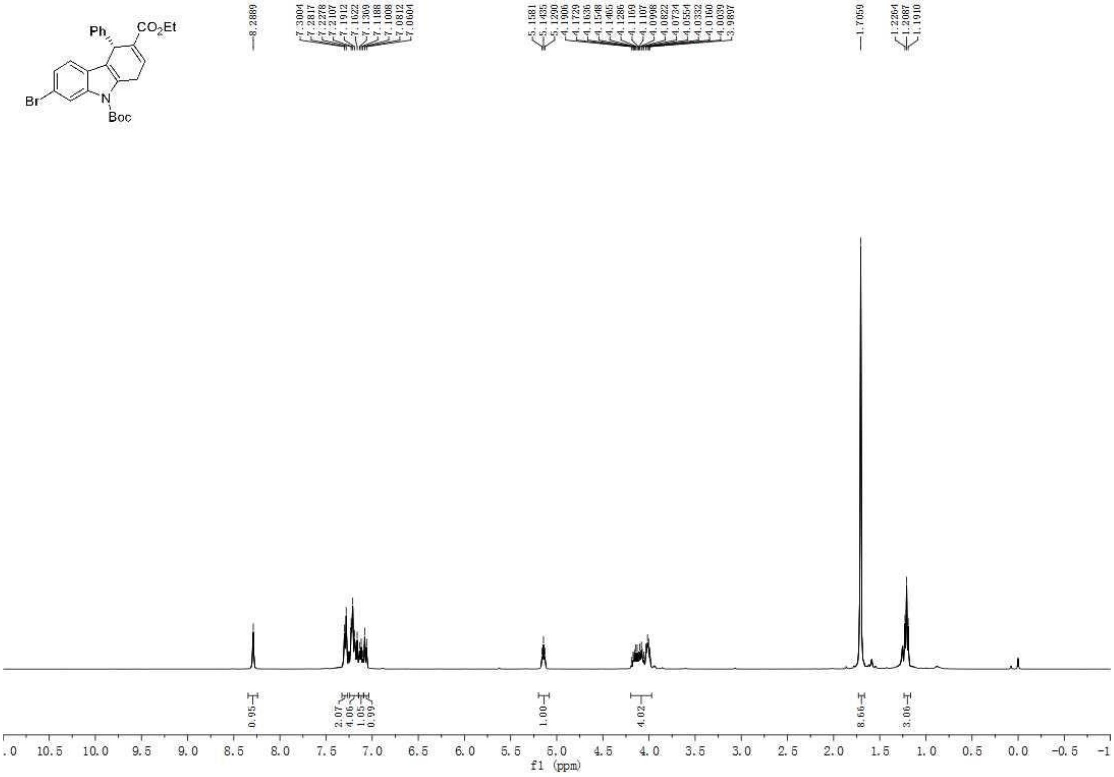

Figure S19. ^{13}C NMR spectrum of **3i**, related to **Scheme 2**.

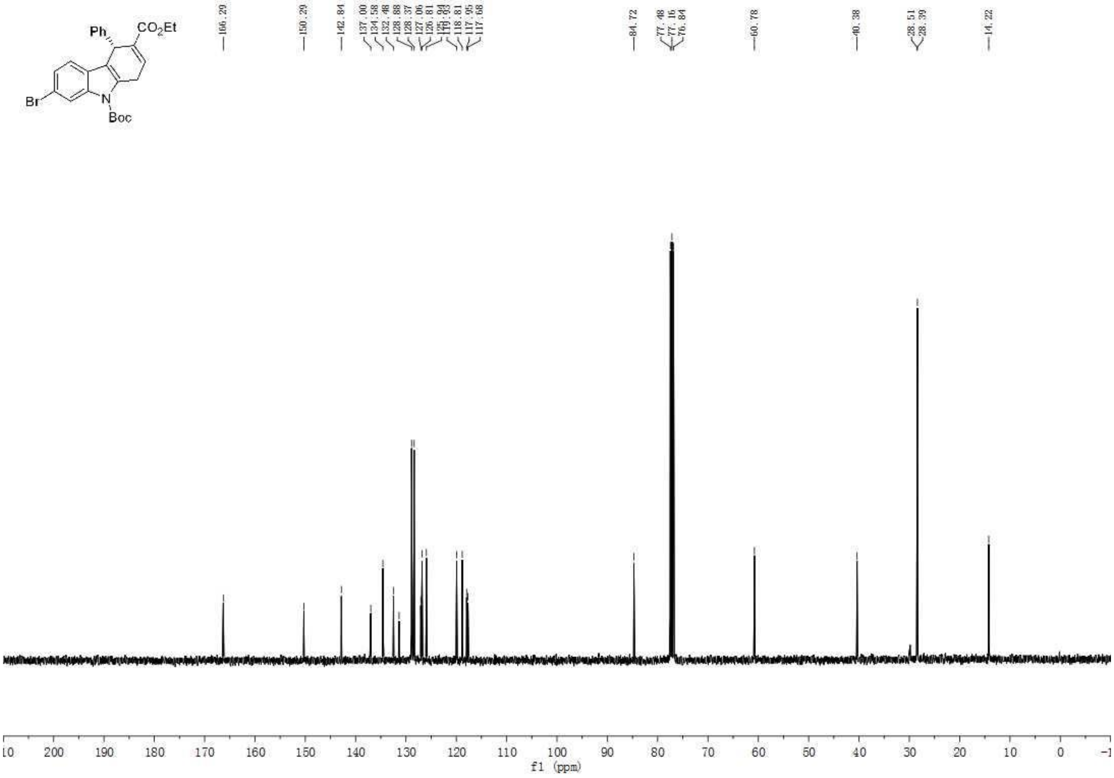

Figure S20. ^{1}H NMR spectrum of **3j**, related to **Scheme 2**.

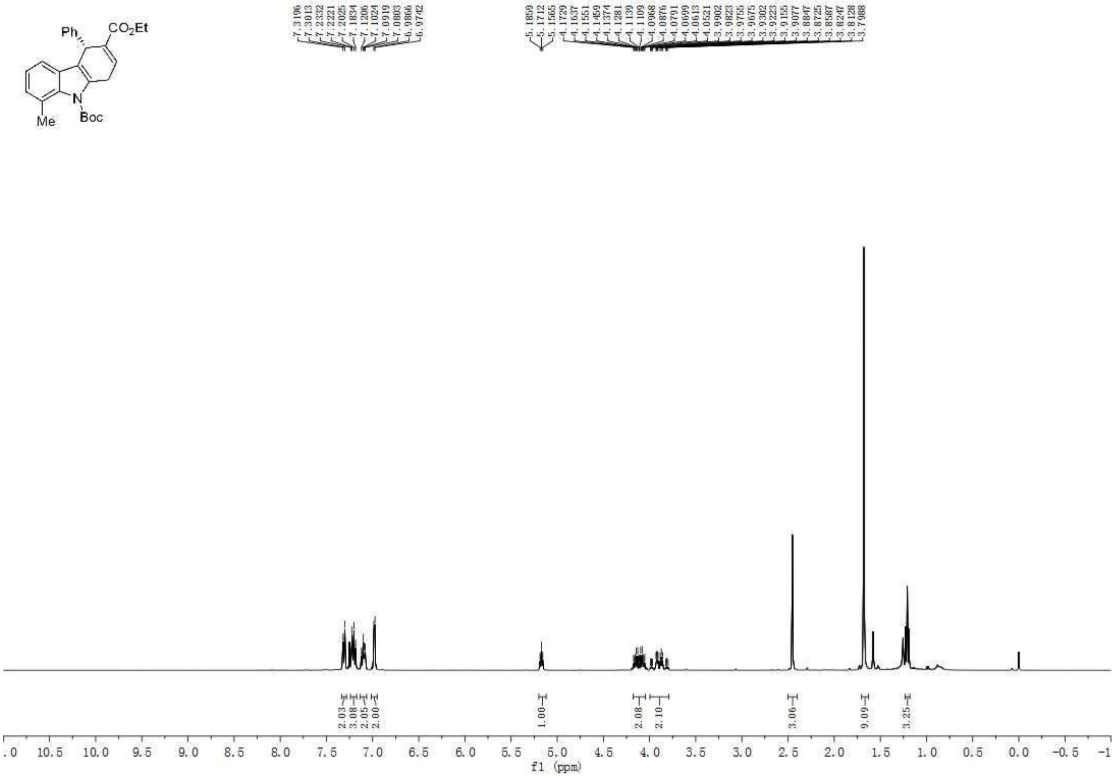

Figure S21. ^{13}C NMR spectrum of **3j**, related to **Scheme 2**.

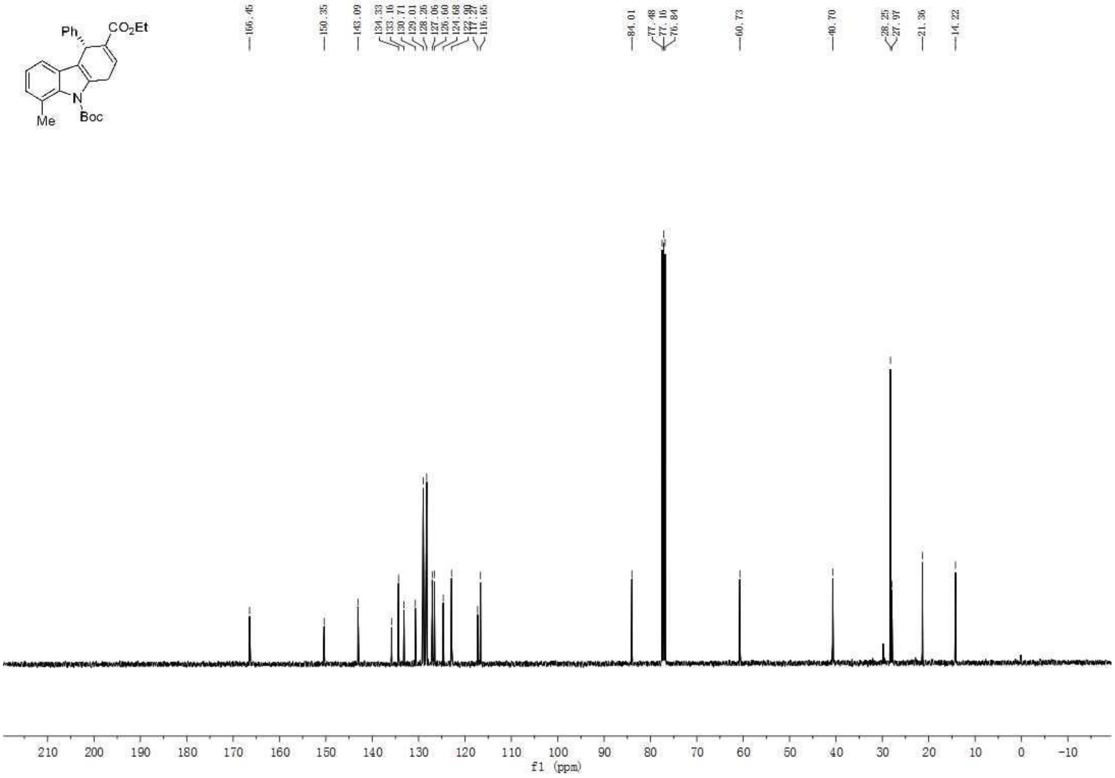

Figure S22. ^{1}H NMR spectrum of **3k**, related to **Scheme 2**.

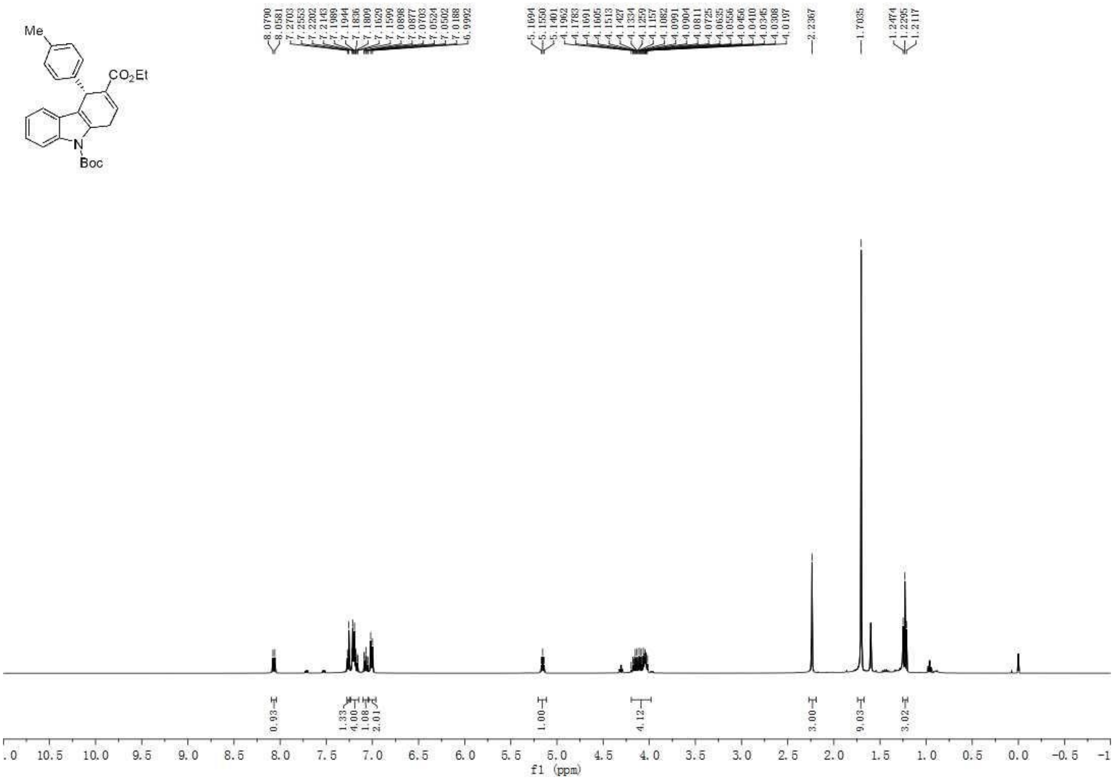

Figure S23. ^{13}C NMR spectrum of **3k**, related to **Scheme 2**.

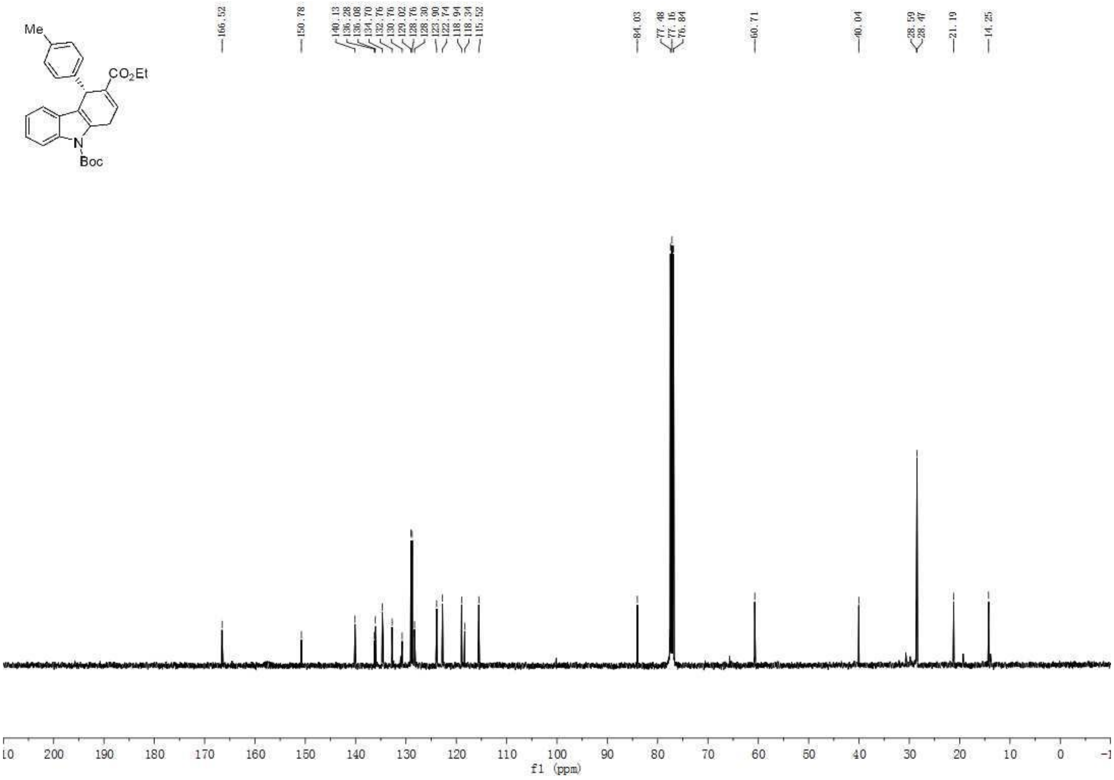

Figure S24. ^{1}H NMR spectrum of **3l**, related to **Scheme 2**.

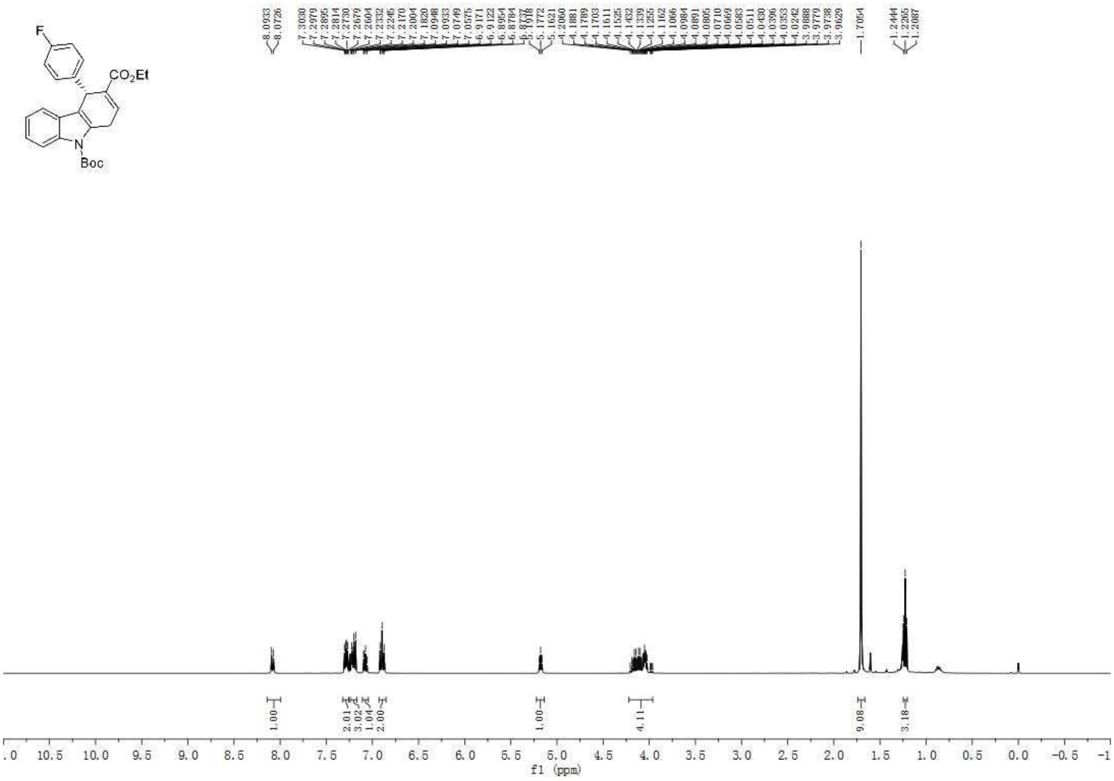

Figure S25. ^{13}C NMR spectrum of **3l**, related to **Scheme 2**.

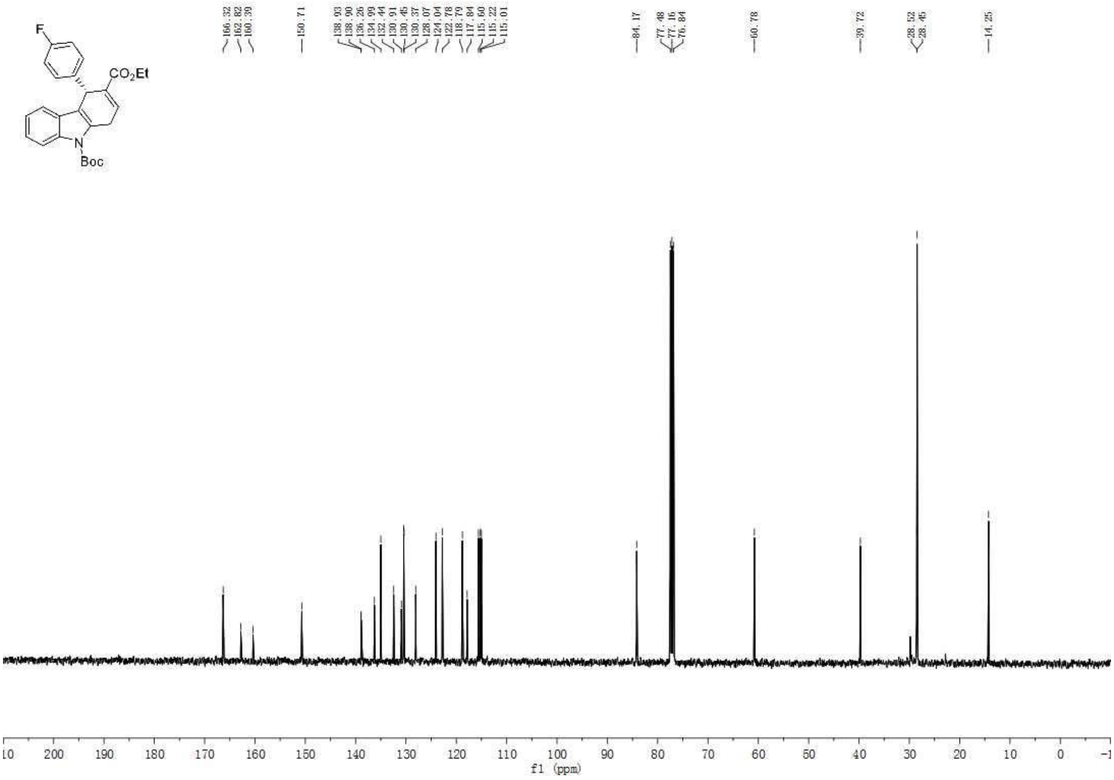

Figure S26. ^{19}F NMR spectrum of **3l**, related to **Scheme 2**.

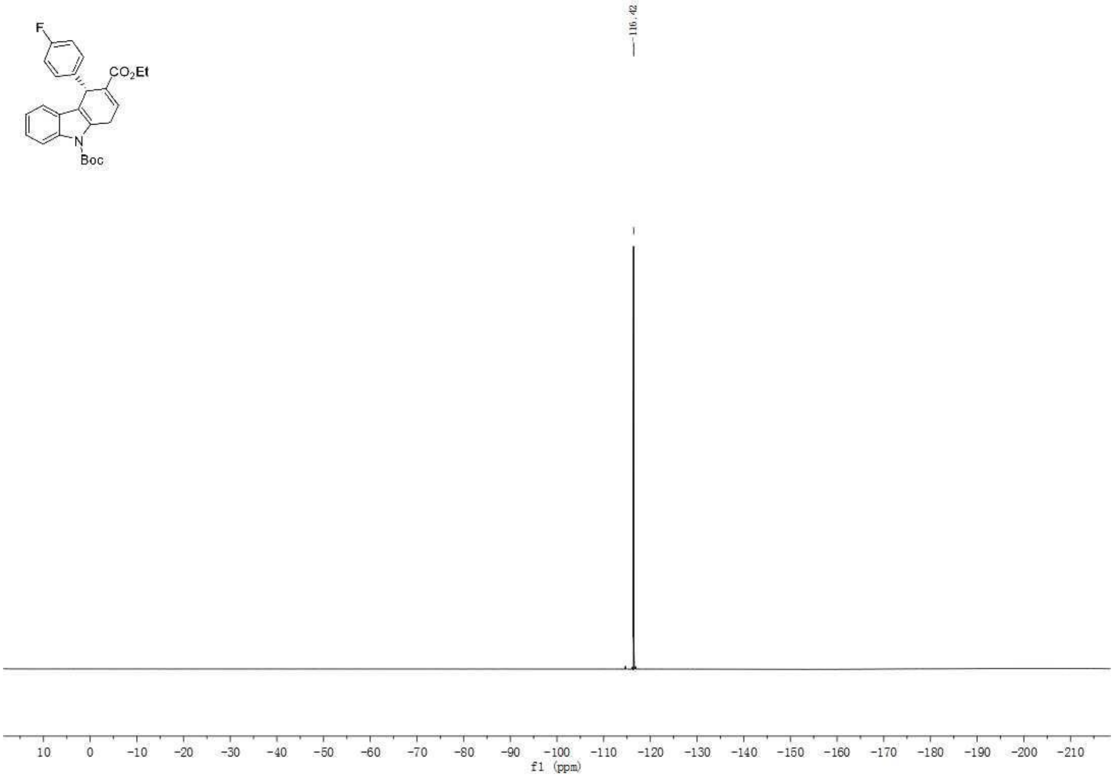

Figure S27. ^{1}H NMR spectrum of **3m,** related to **Scheme 2**.

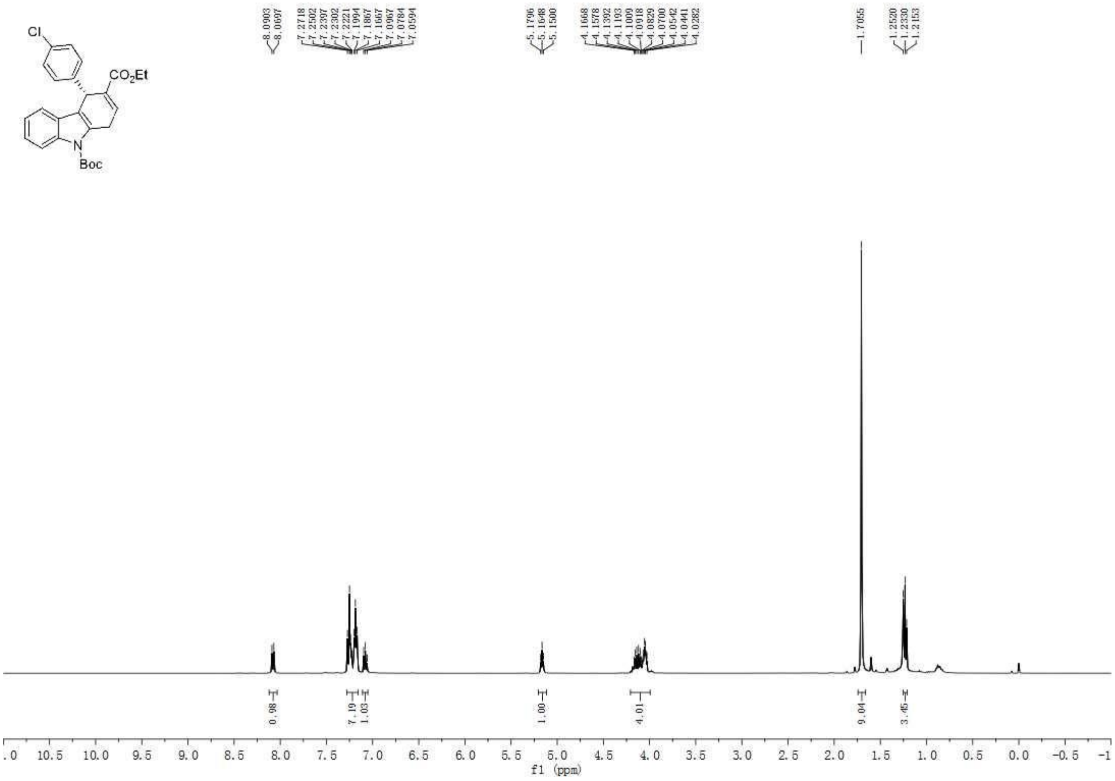

Figure S28. ^{13}C NMR spectrum of **3m**, related to **Scheme 2**.

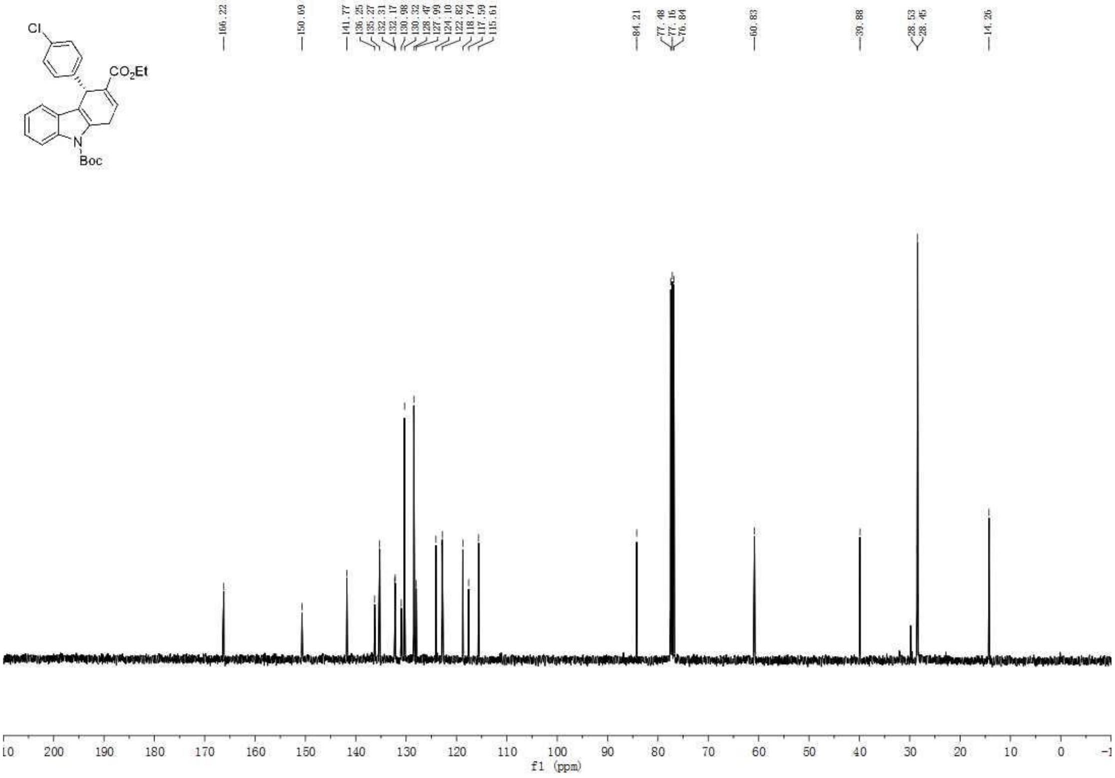

Figure S29. ^{1}H NMR spectrum of **3n**, related to **Scheme 2**.

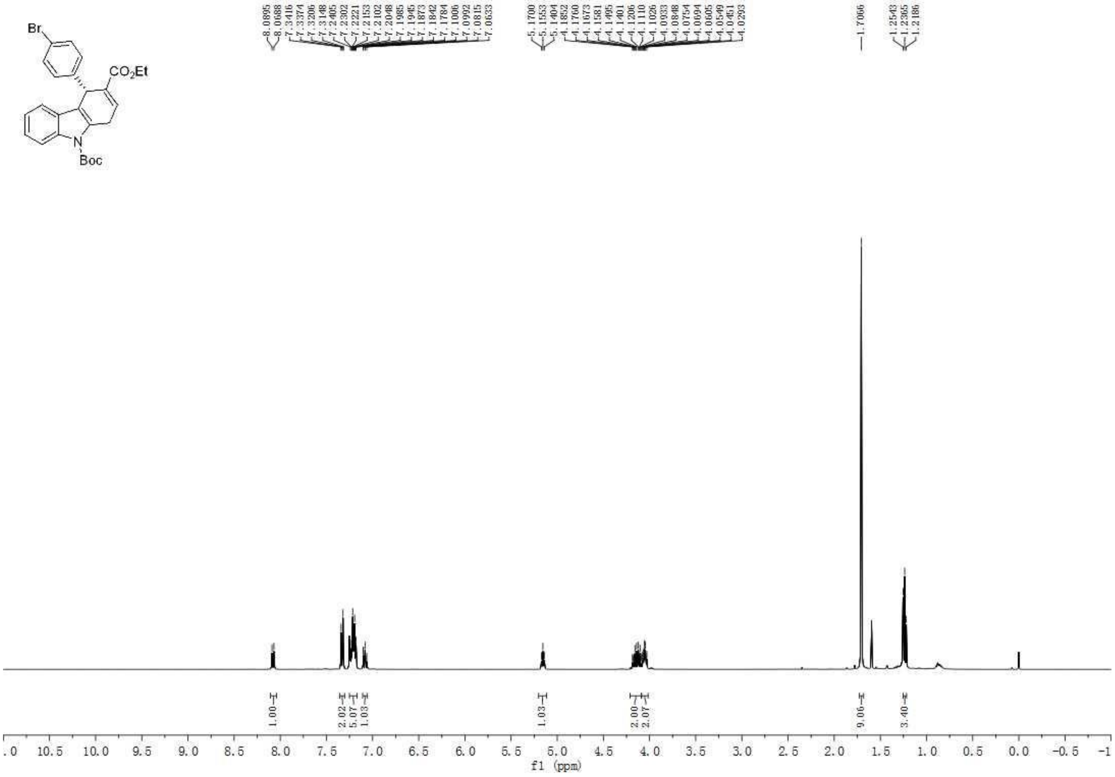

Figure S30. ^{13}C NMR spectrum of **3n**, related to **Scheme 2**.

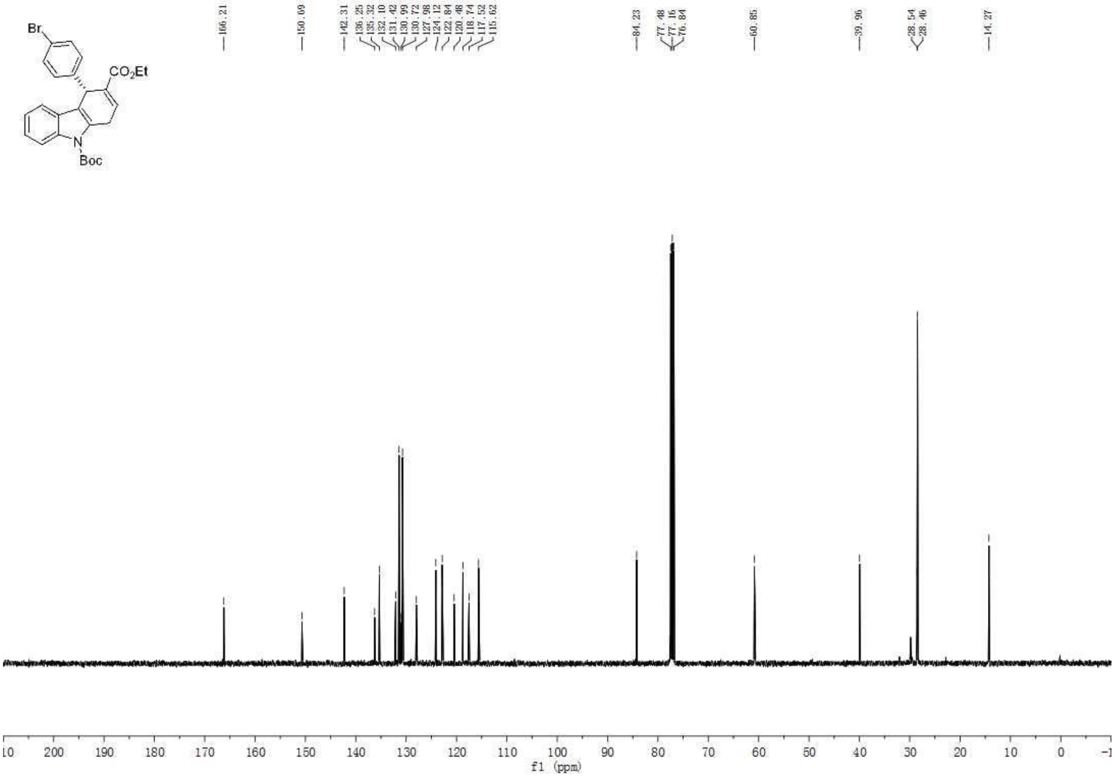

Figure S31. ^{1}H NMR spectrum of **3o**, related to **Scheme 2**.

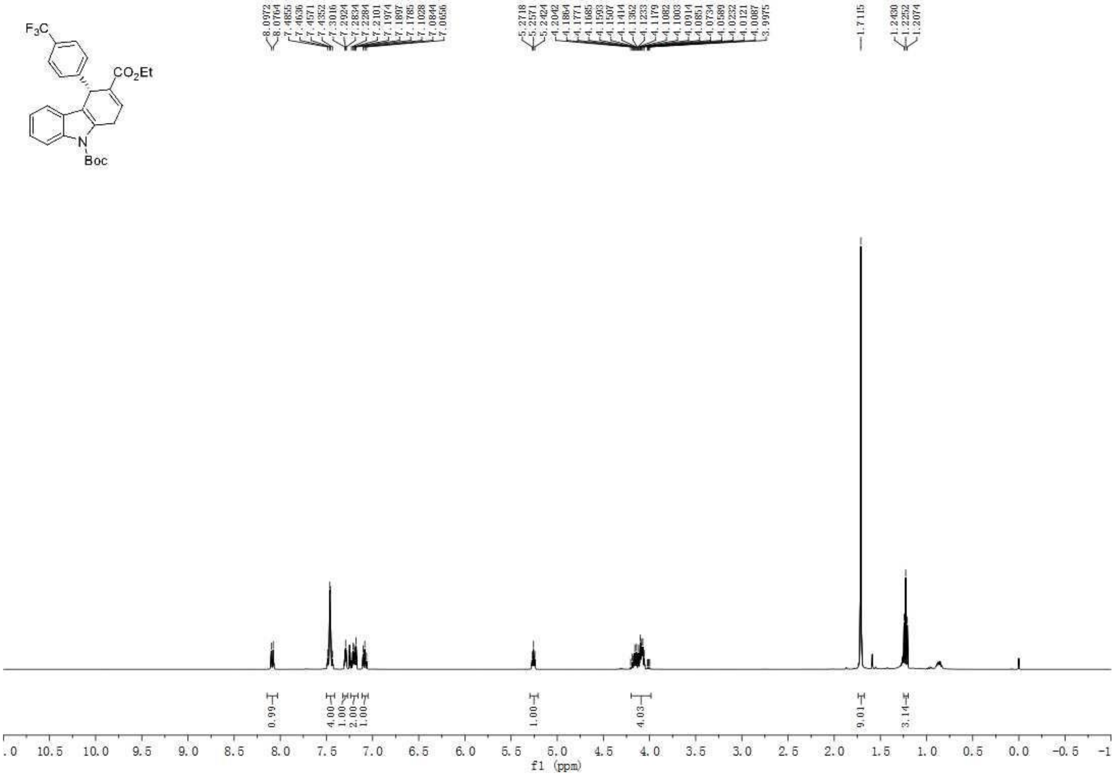

Figure S32. ^{13}C NMR spectrum of **3o**, related to **Scheme 2**.

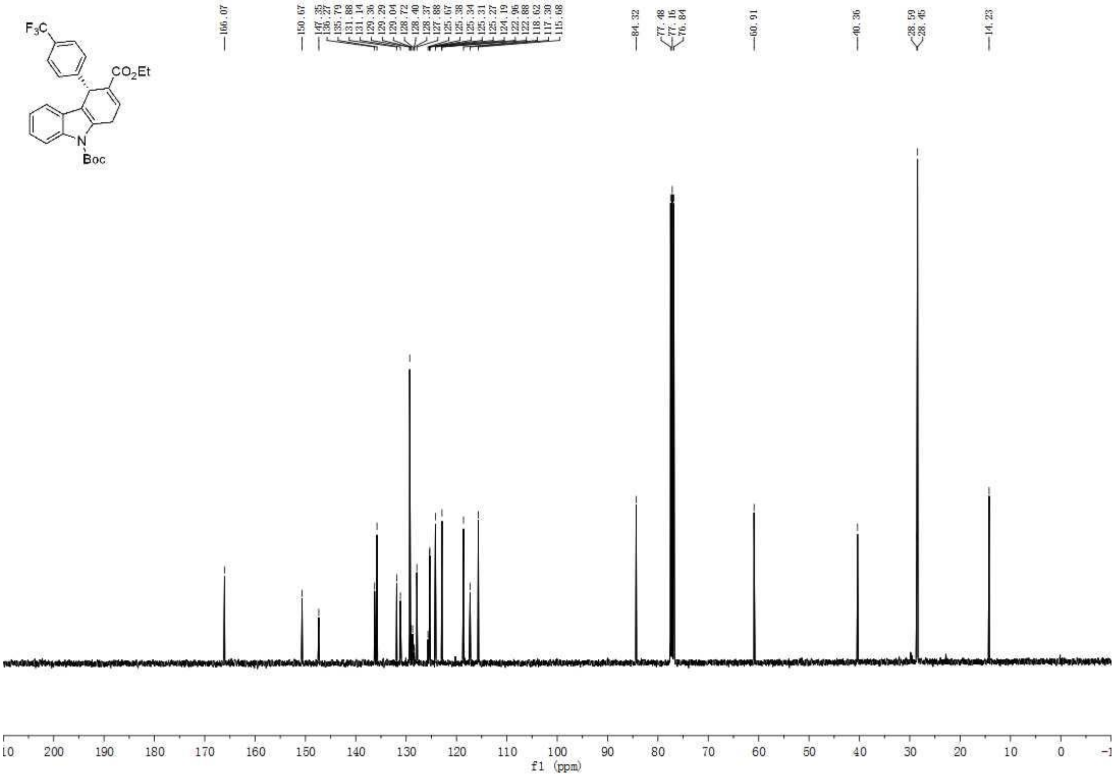

Figure S33. ^{19}F NMR spectrum of **3o**, related to **Scheme 2**.

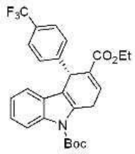

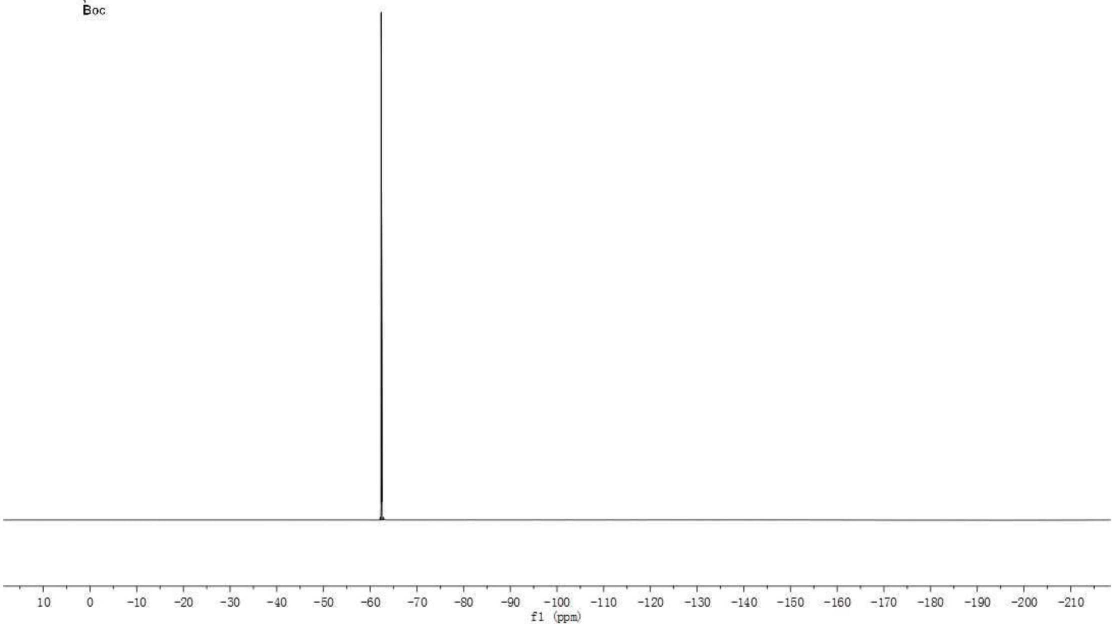

Figure S34. ^{1}H NMR spectrum of **3p**, related to **Scheme 2**.

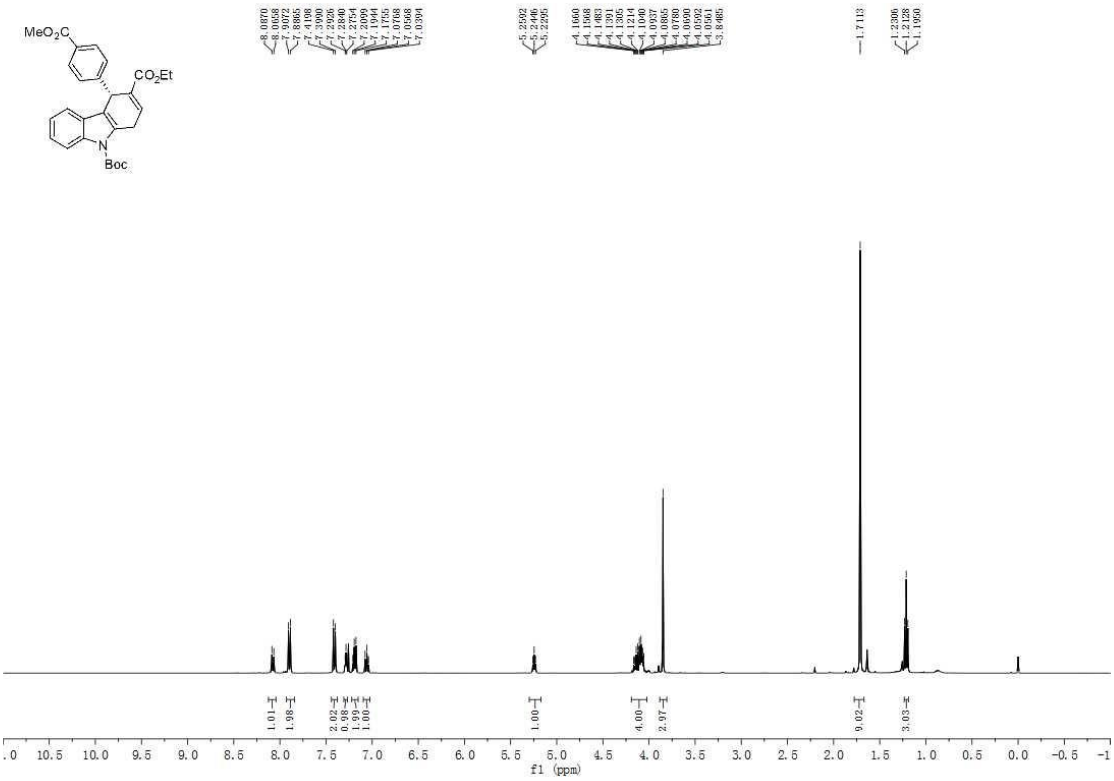

Figure S35. ^{13}C NMR spectrum of **3p**, related to **Scheme 2**.

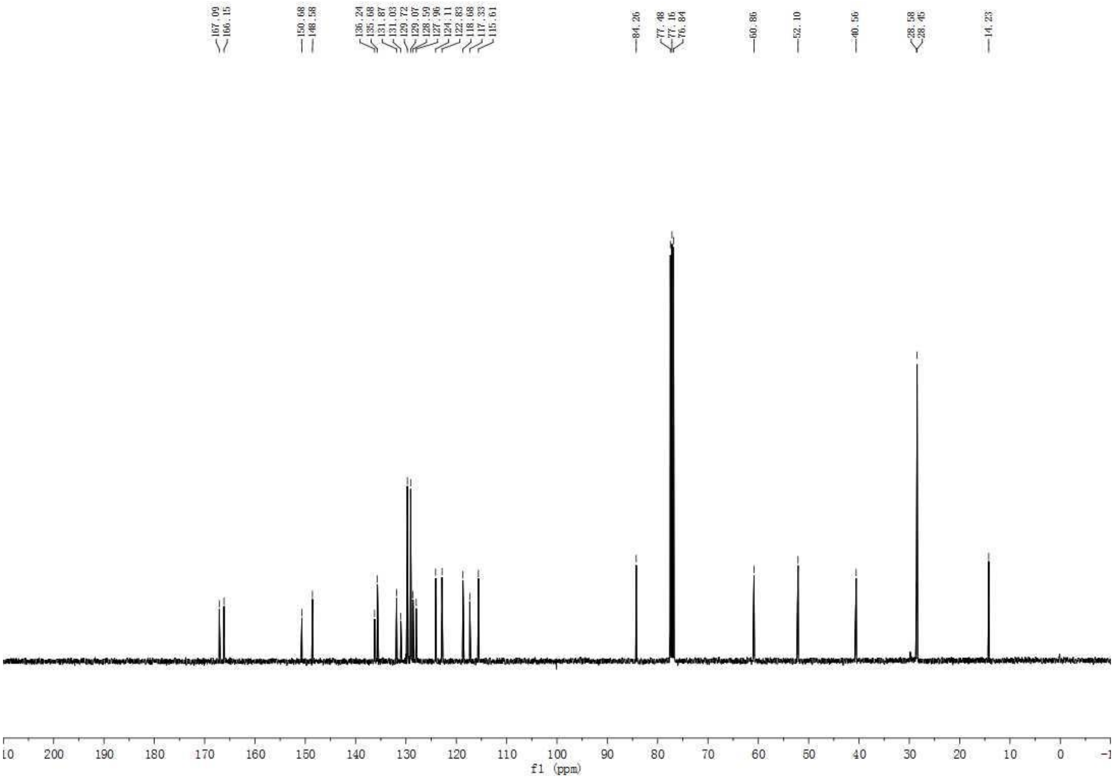

Figure S36. ^{1}H NMR spectrum of **3q**, related to **Scheme 2.**

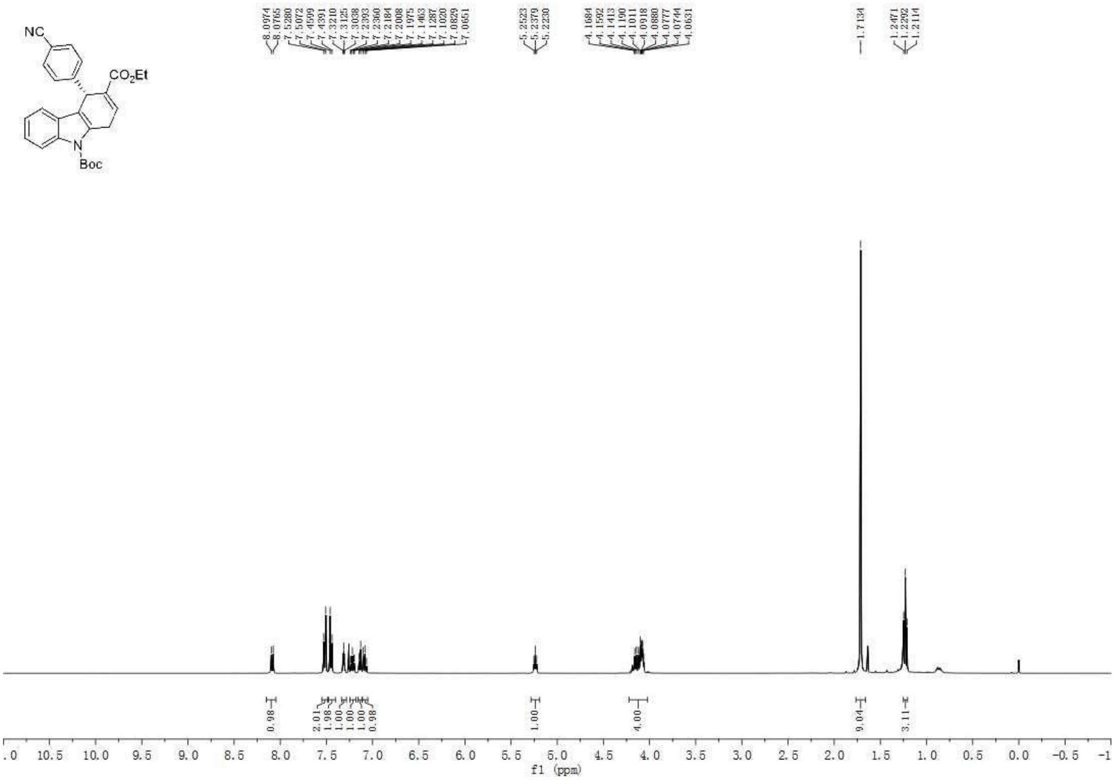

Figure S37. ^{13}C NMR spectrum of **3q**, related to **Scheme 2**.

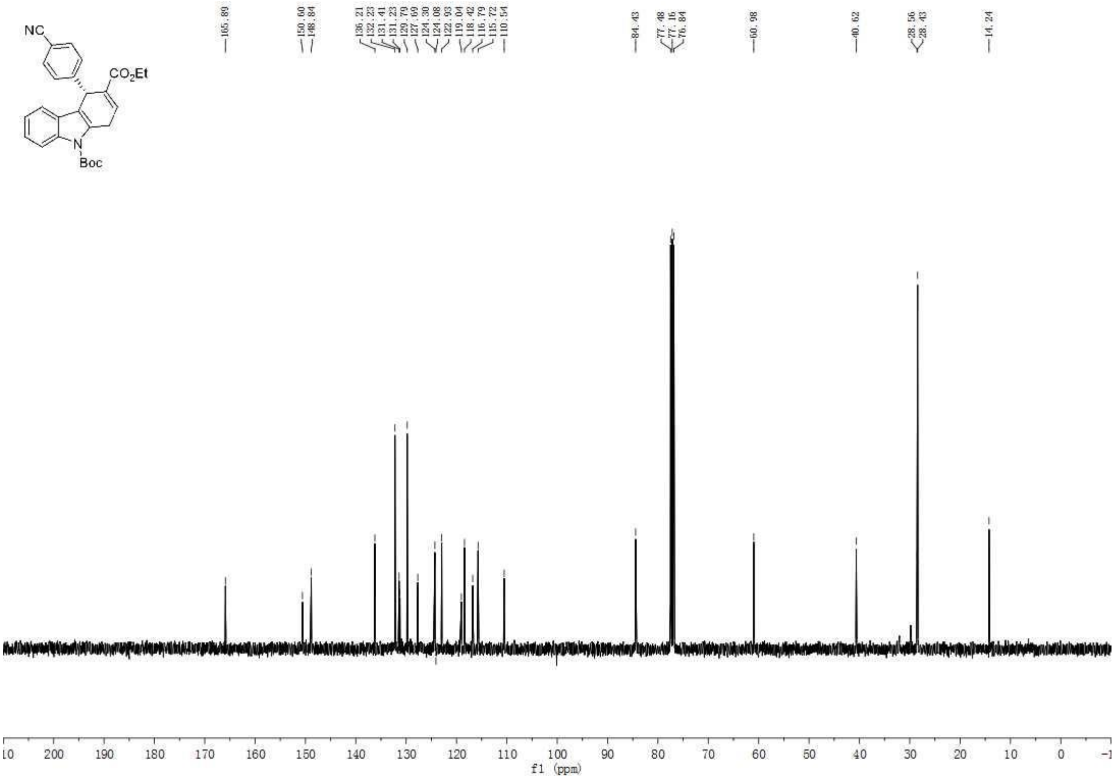

Figure S38. ^{1}H NMR spectrum of **3r**, related to **Scheme 2**.

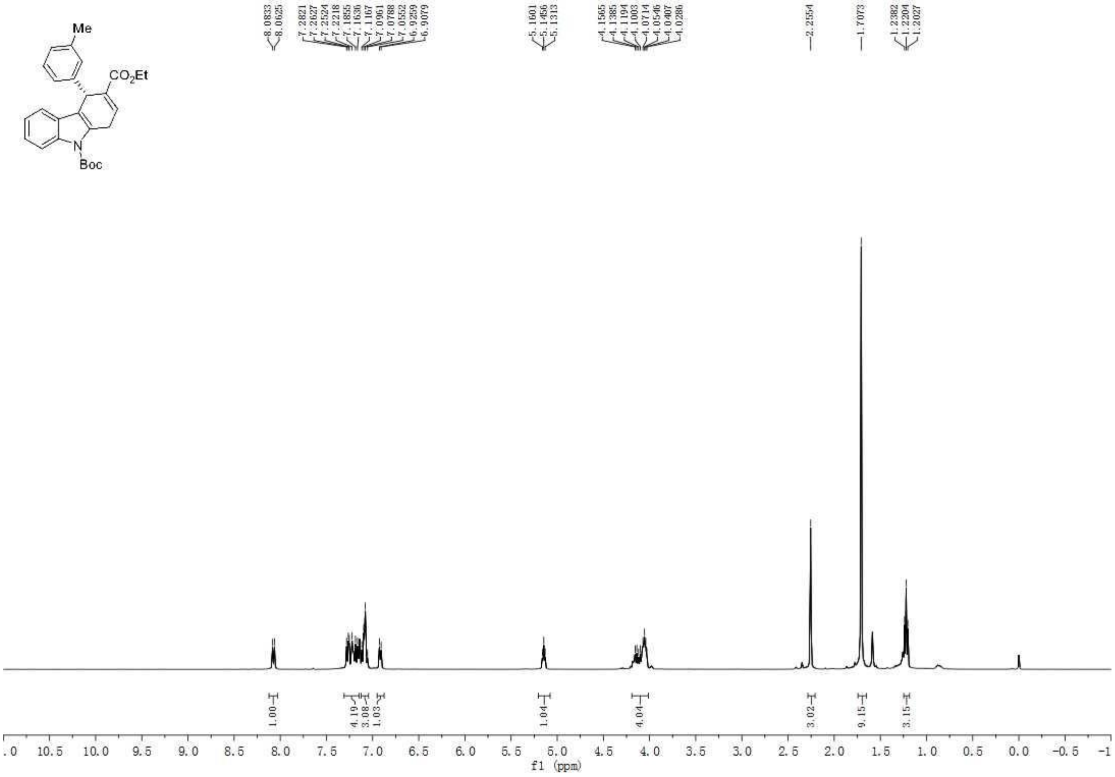

Figure S39. ^{13}C NMR spectrum of **3r**, related to **Scheme 2**.

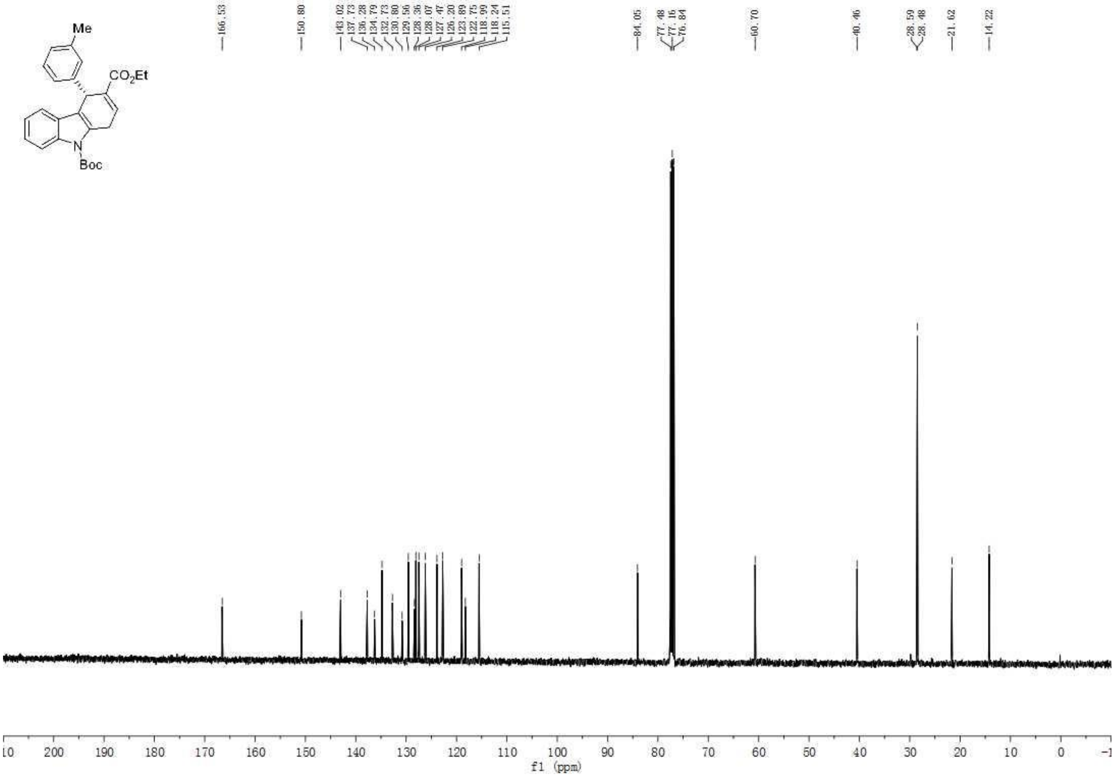

Figure S40. ^{1}H NMR spectrum of **3s**, related to **Scheme 2**.

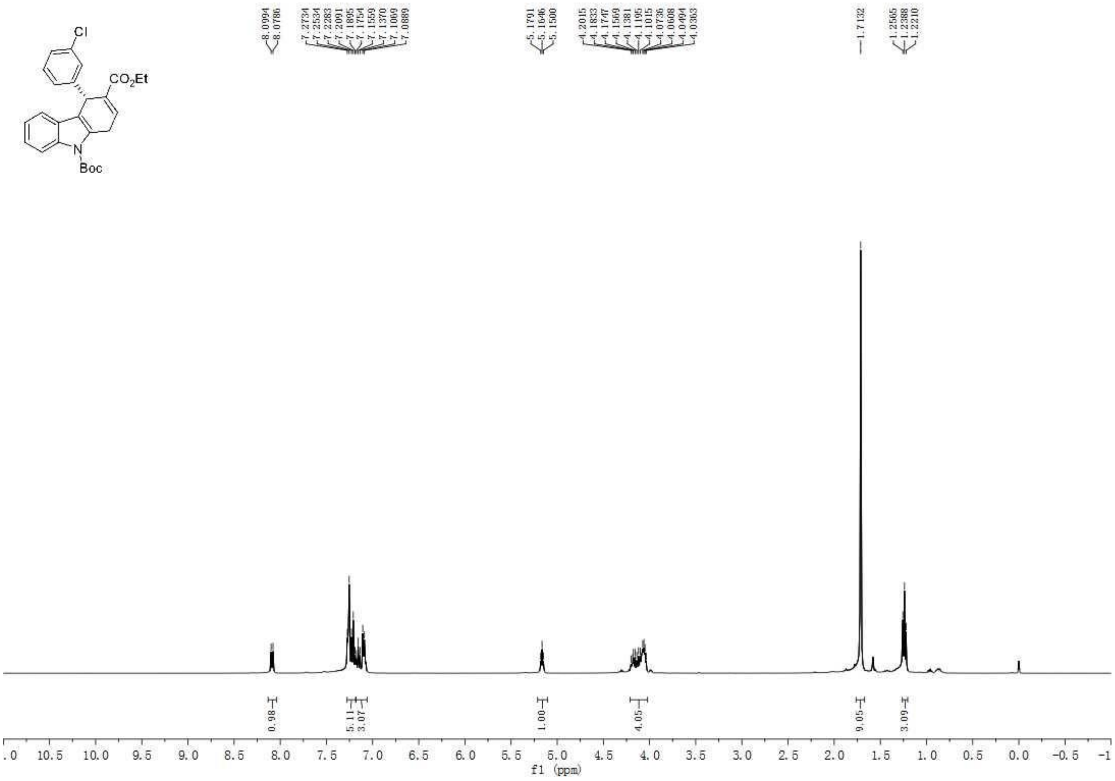

Figure S41. ^{13}C NMR spectrum of **3s**, related to **Scheme 2**.

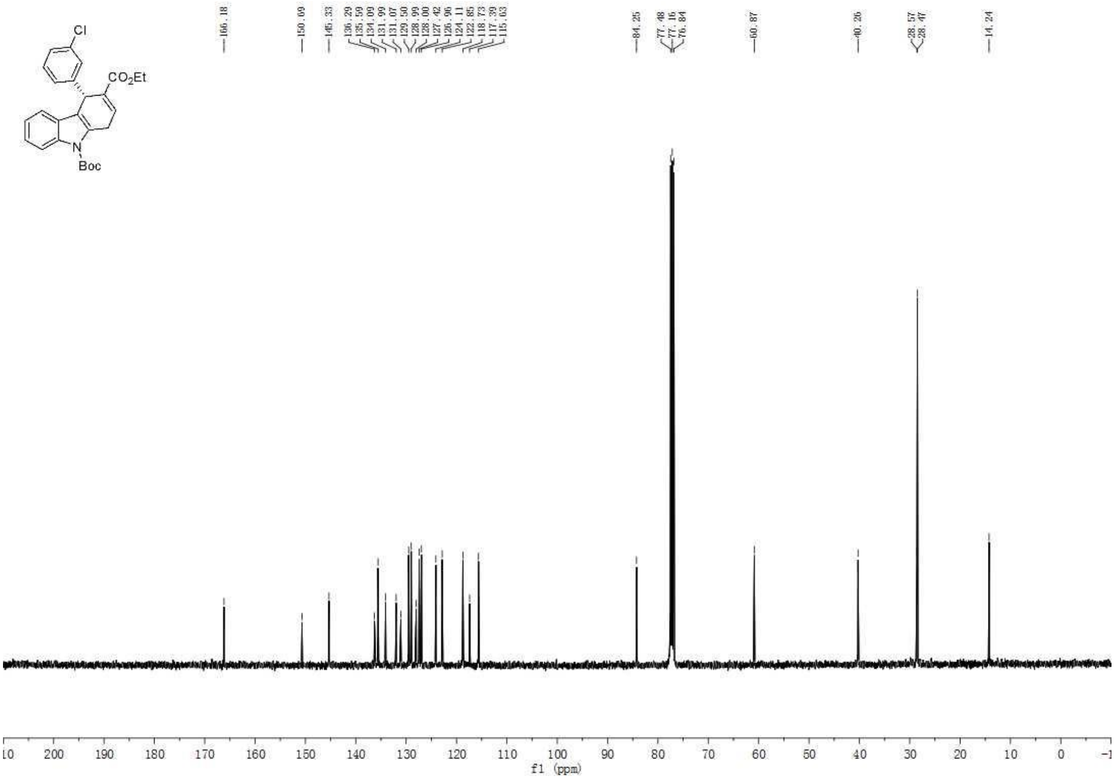

Figure S42. ^{1}H NMR spectrum of **3t**, related to **Scheme 2**.

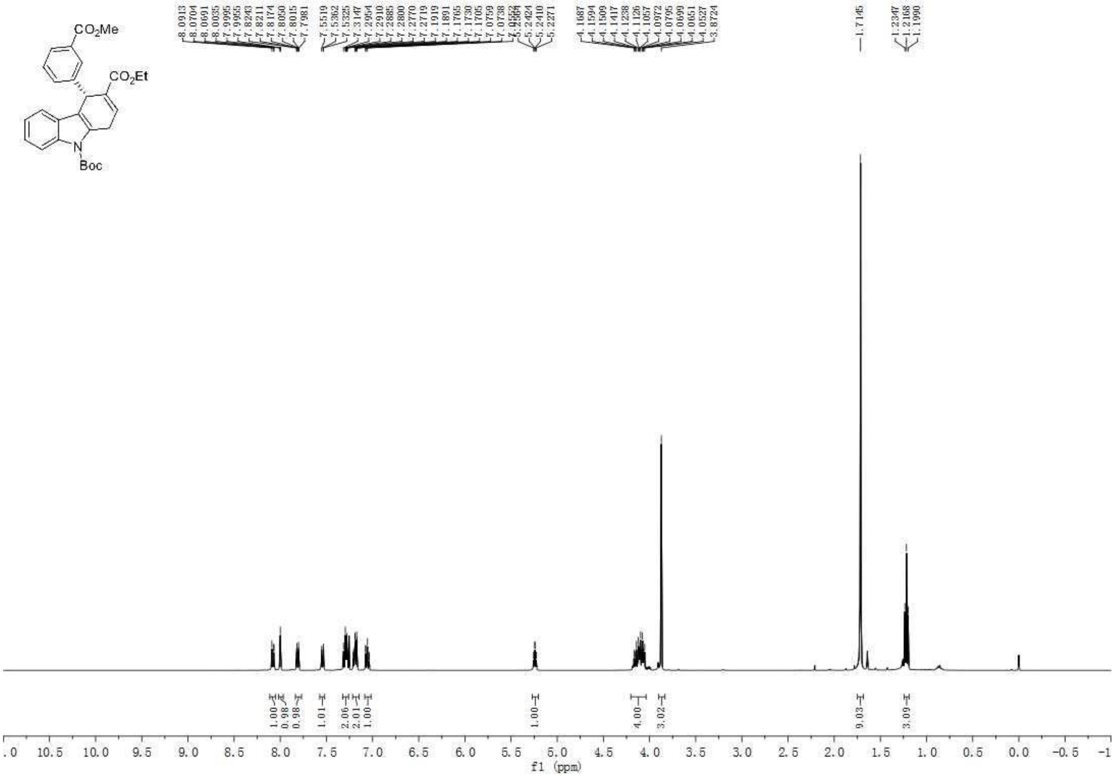

Figure S43. ^{13}C NMR spectrum of **3t**, related to **Scheme 2**.

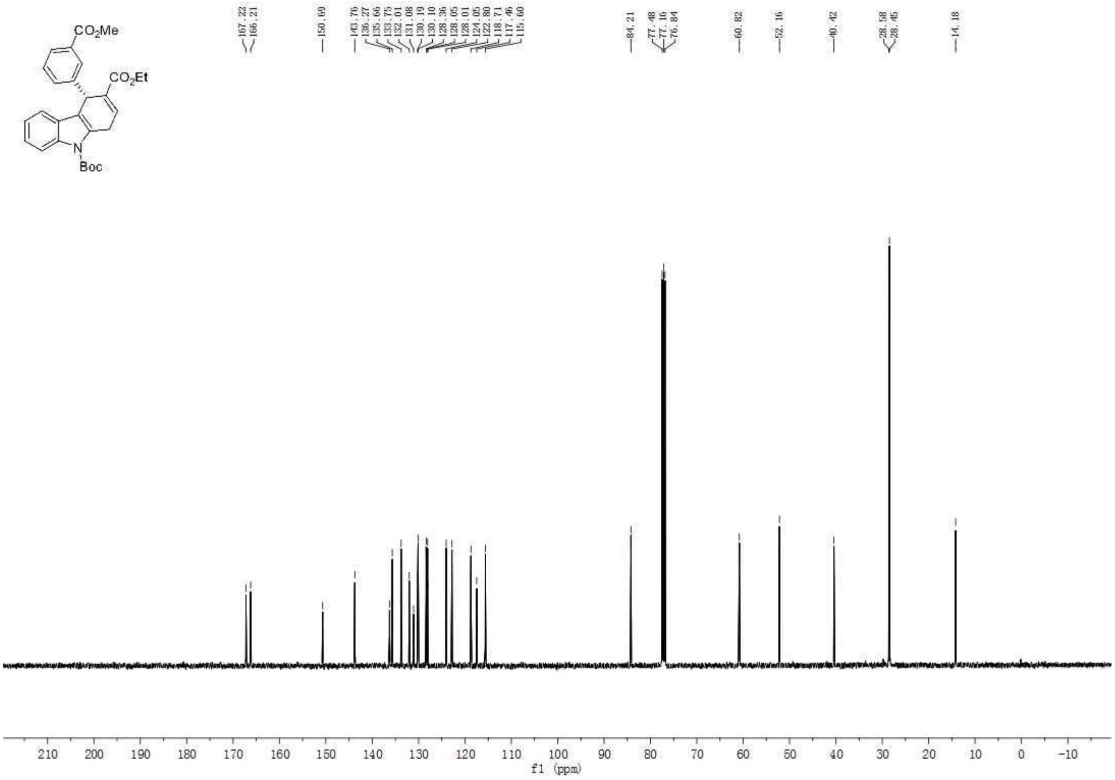

Figure S44. ^{1}H NMR spectrum of **3u**, related to **Scheme 2**.

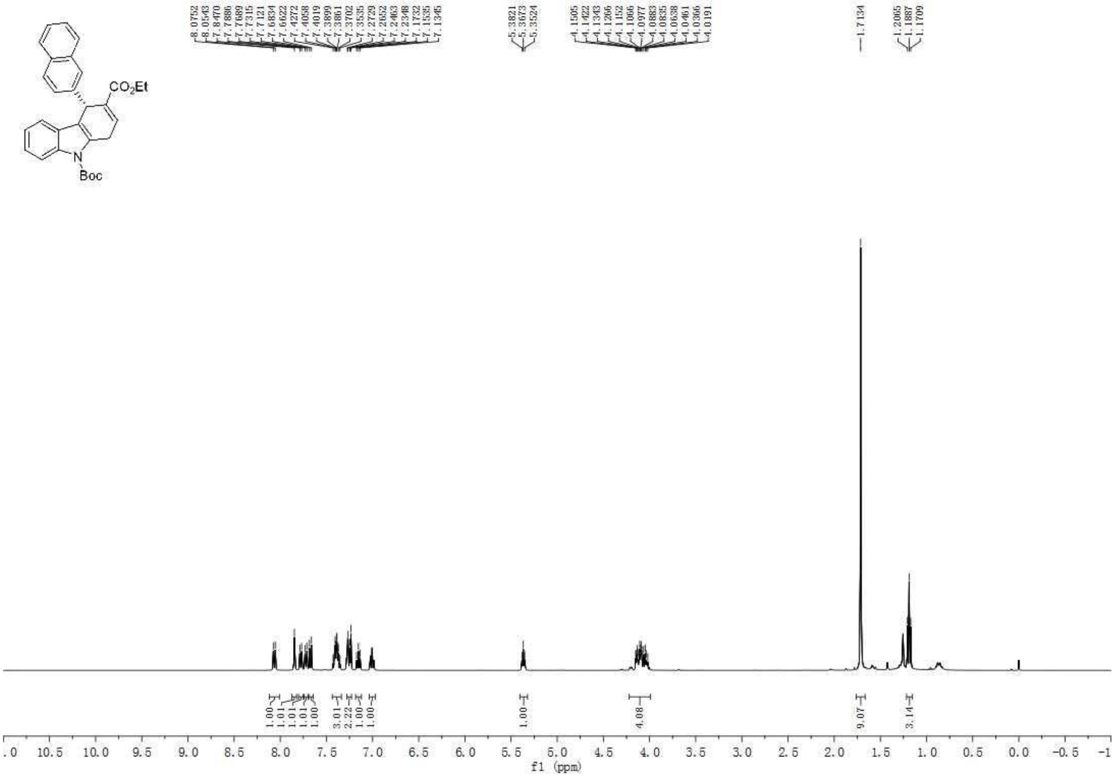

Figure S45. ^{13}C NMR spectrum of **3u**, related to **Scheme 2**.

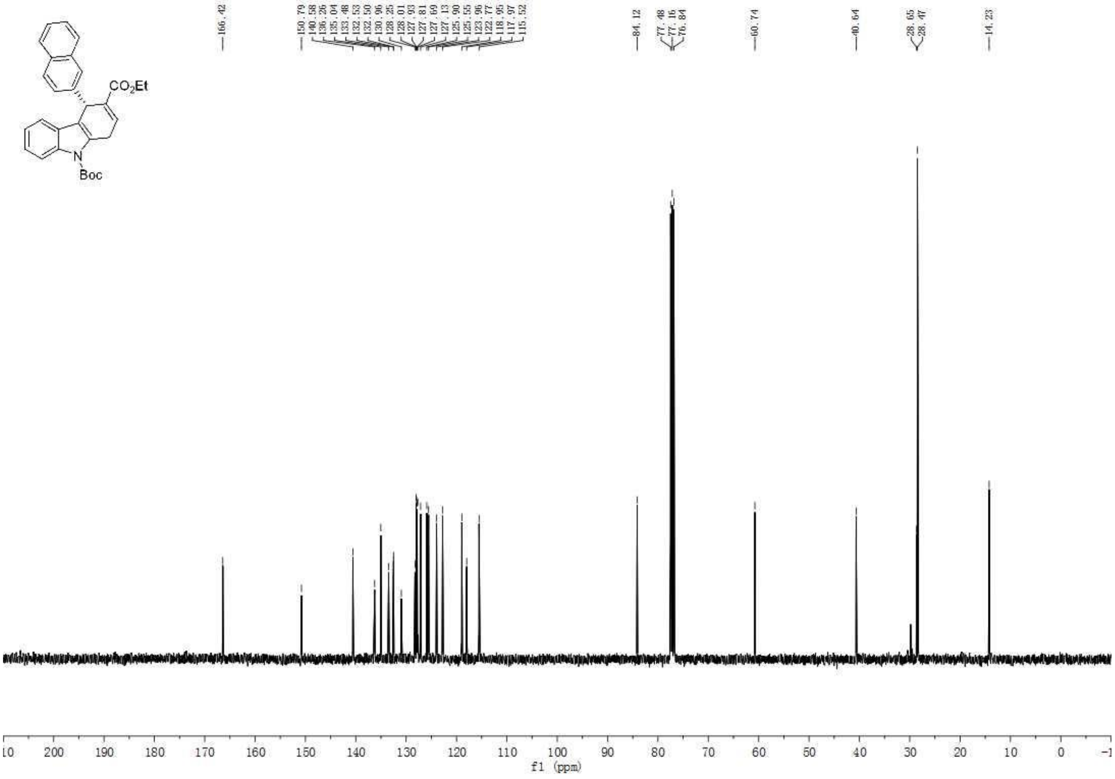

Figure S46. ^{1}H NMR spectrum of **3v**, related to **Scheme 2.**

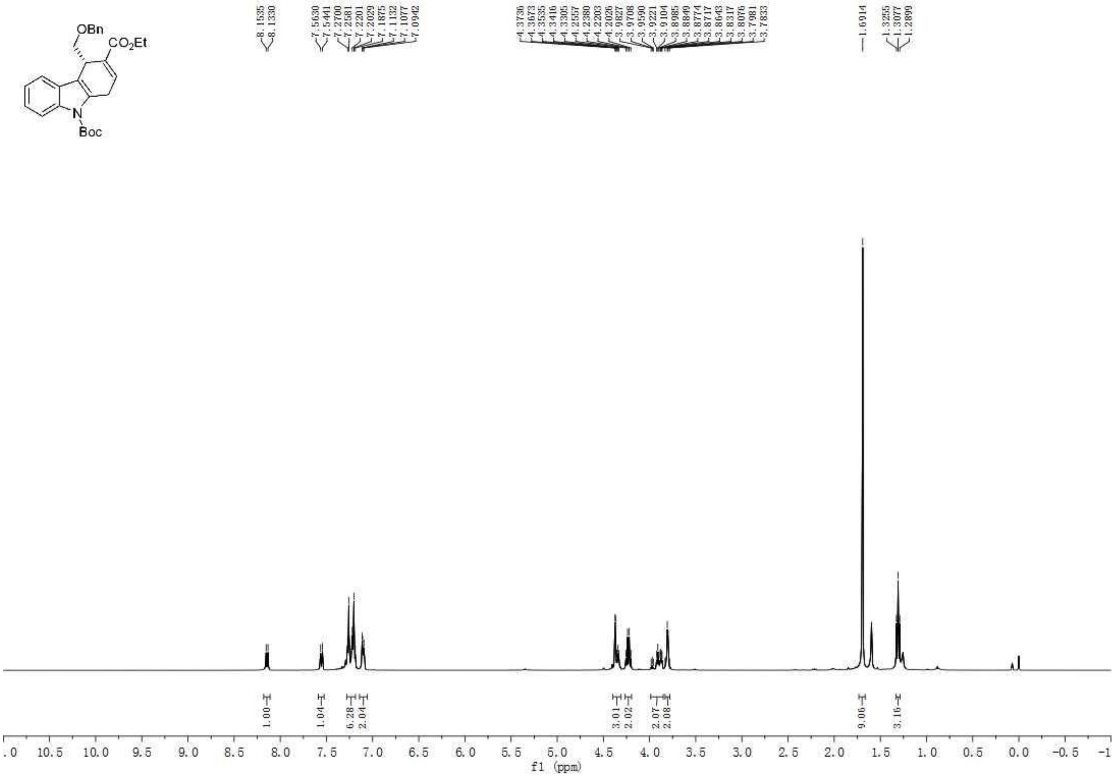

Figure S47. ^{13}C NMR spectrum of **3v**, related to **Scheme 2**.

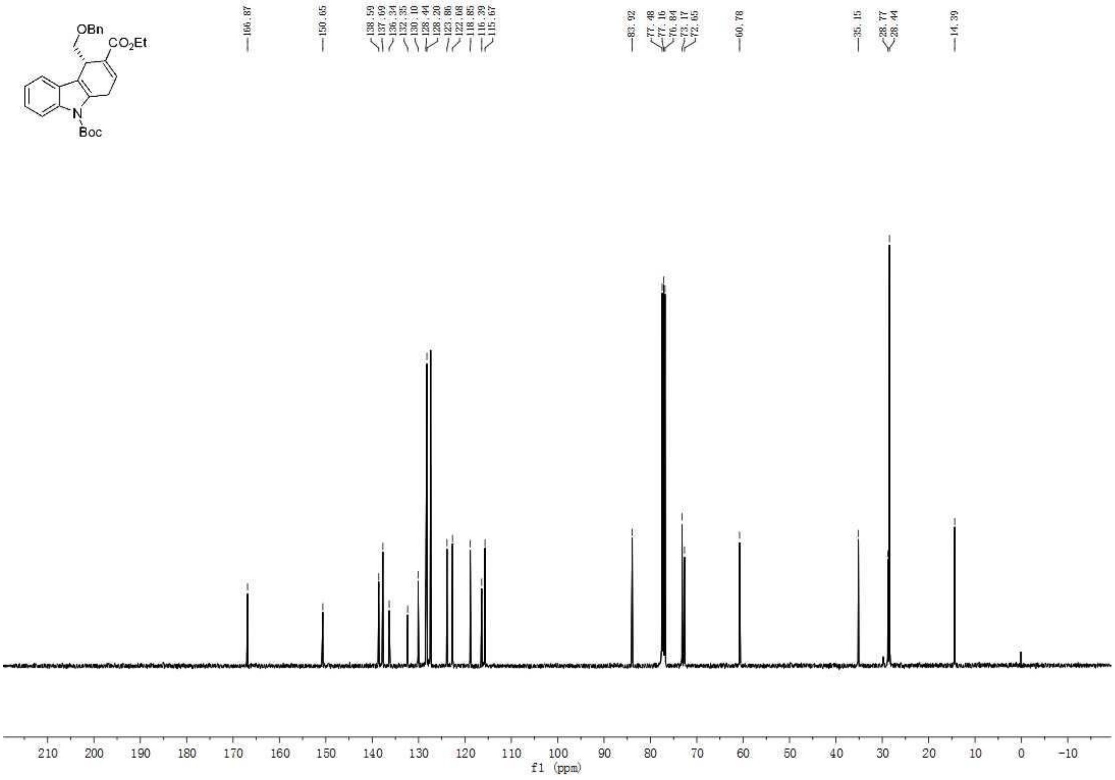

Figure S48. ^{1}H NMR spectrum of **3w**, related to **Scheme 2**.

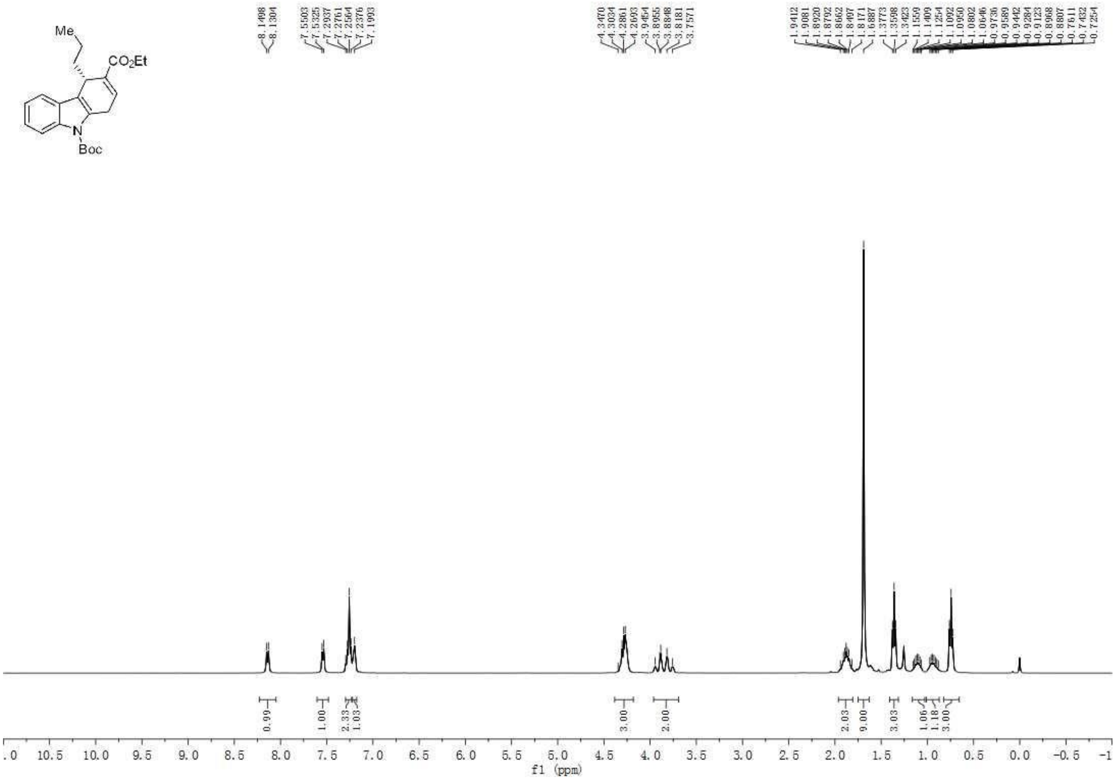

Figure S49. ^{13}C NMR spectrum of **3w**, related to **Scheme 2**.

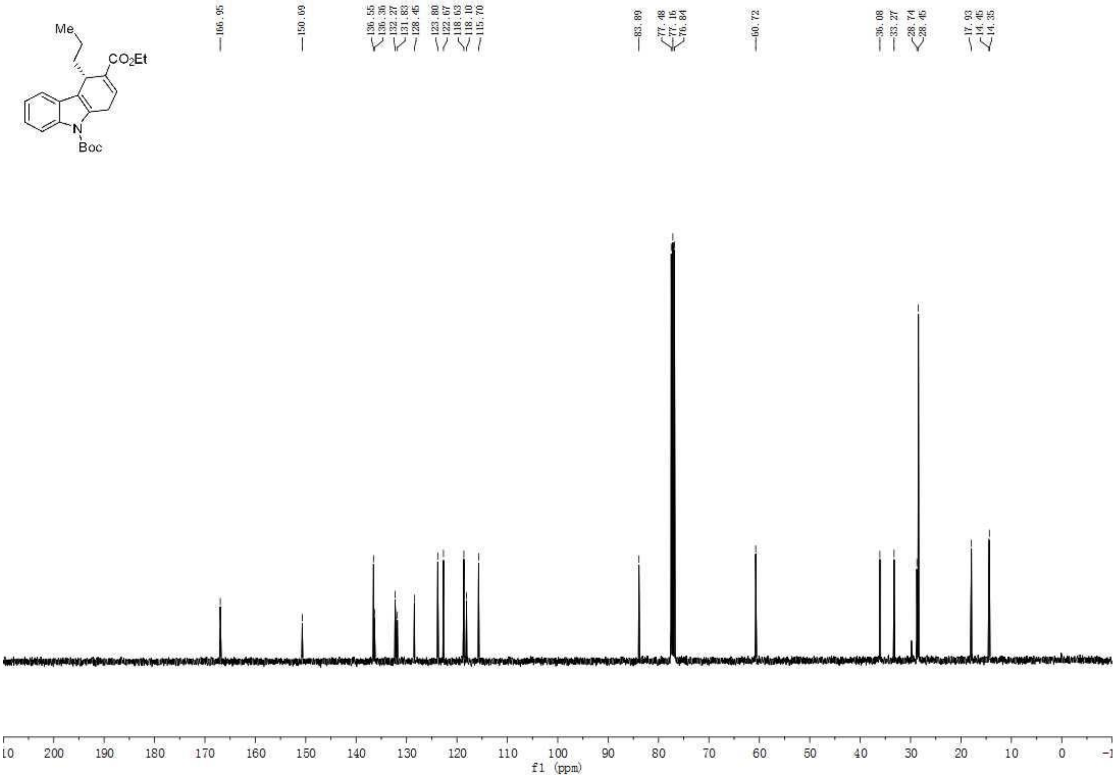

Figure S50. ^{1}H NMR spectrum of **3x**, related to **Scheme 2**.

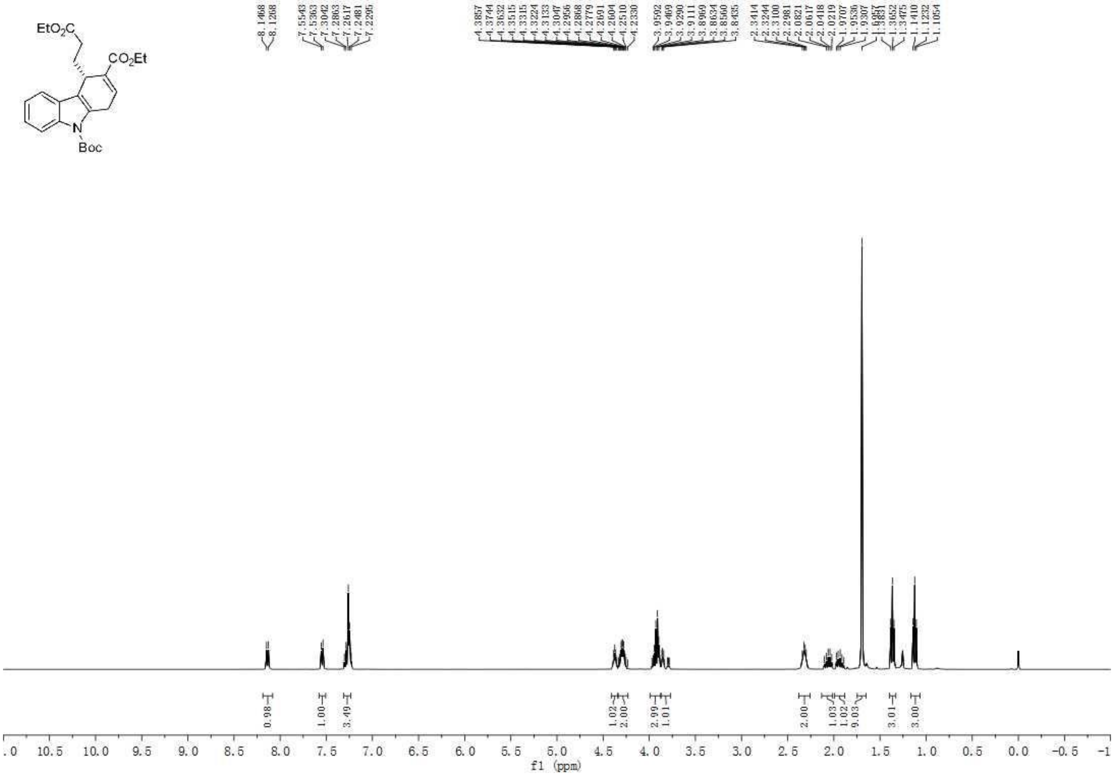

Figure S51. ^{13}C NMR spectrum of **3x**, related to **Scheme 2**.

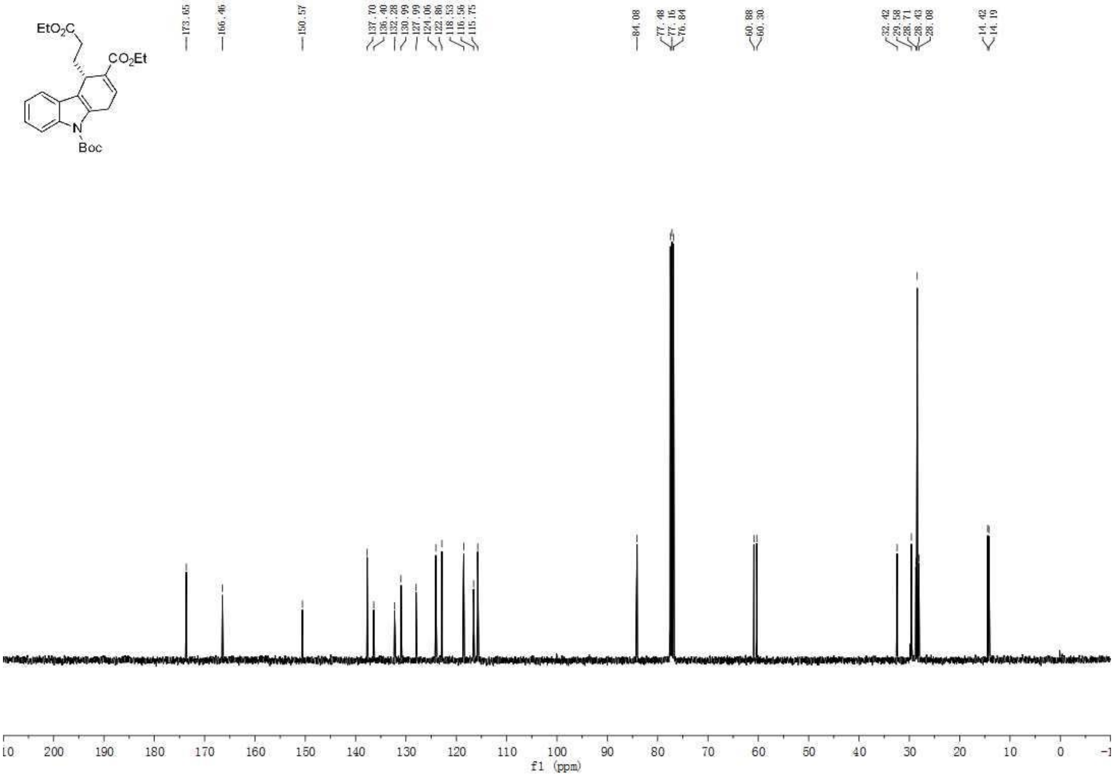

Figure S52. ^{1}H NMR spectrum of **3y**, related to **Scheme 2**.

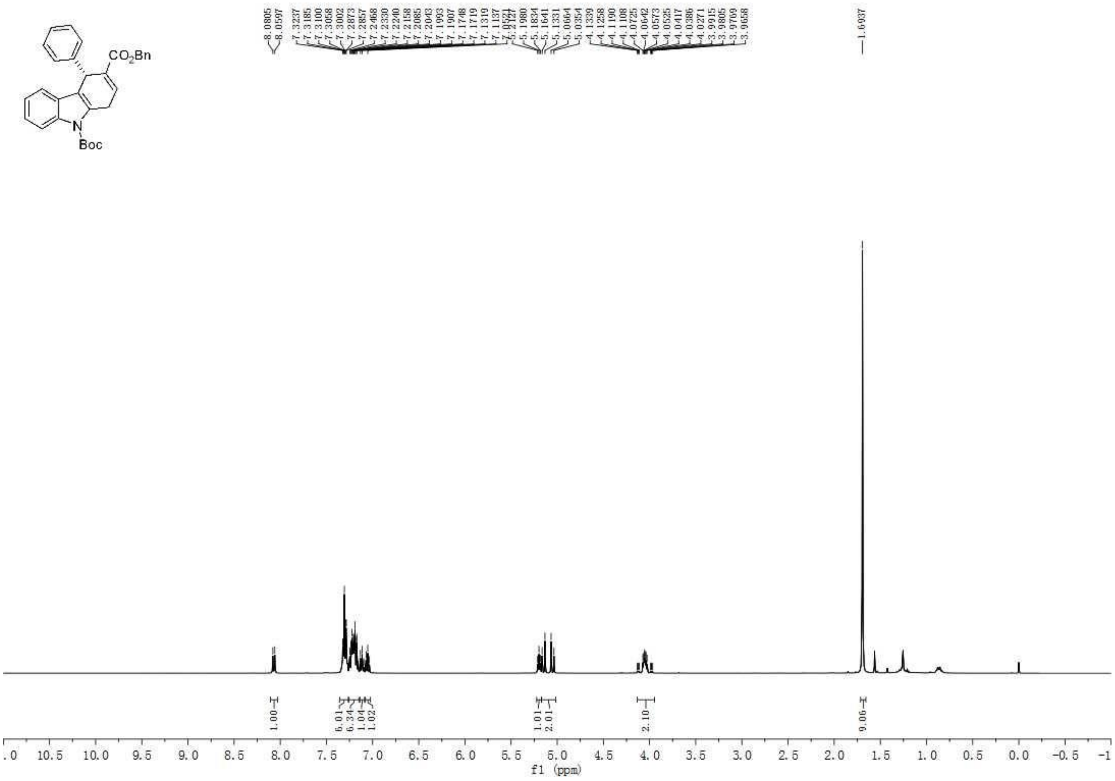

Figure S53. ^{13}C NMR spectrum of **3y**, related to **Scheme 2**.

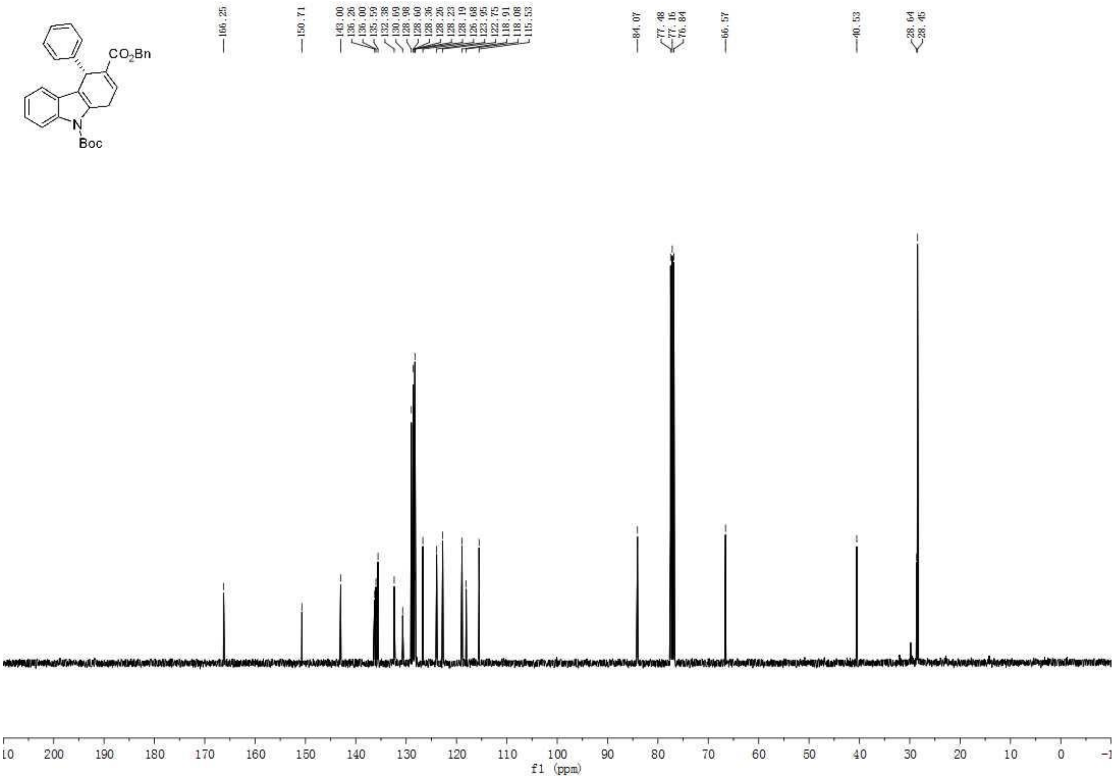

Figure S54. ^{1}H NMR spectrum of **3z**, related to **Scheme 2.**

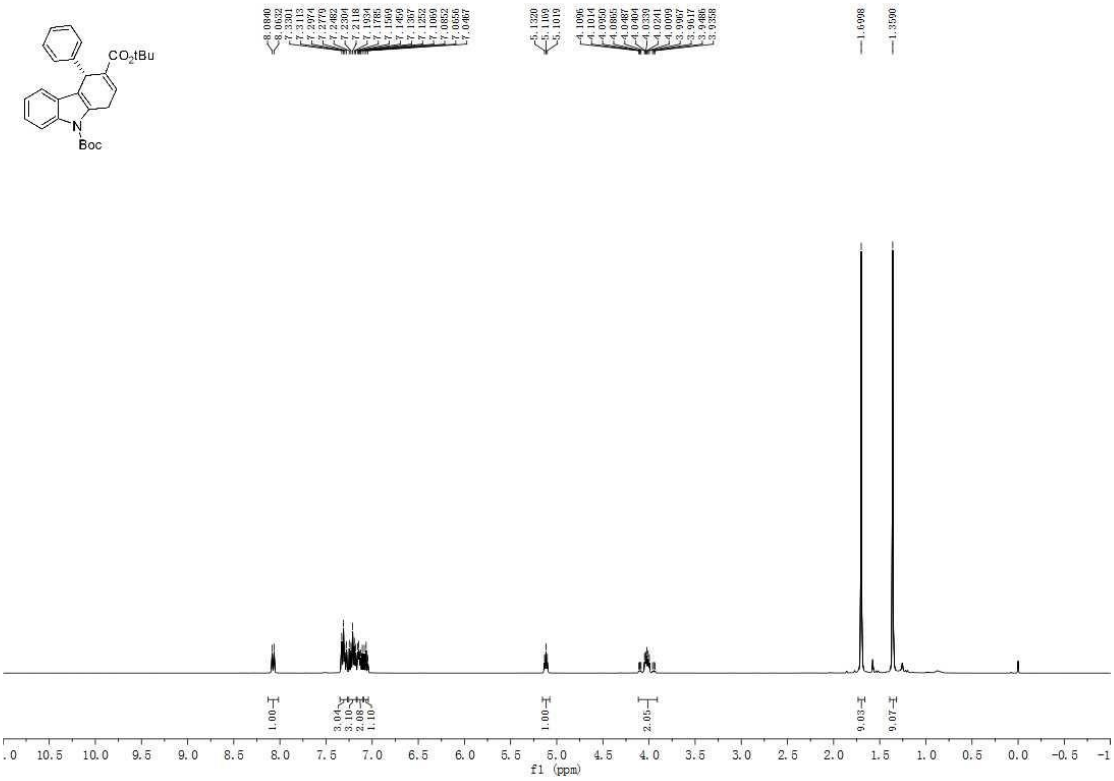

Figure S55. ^{13}C NMR spectrum of **3z**, related to **Scheme 2**.

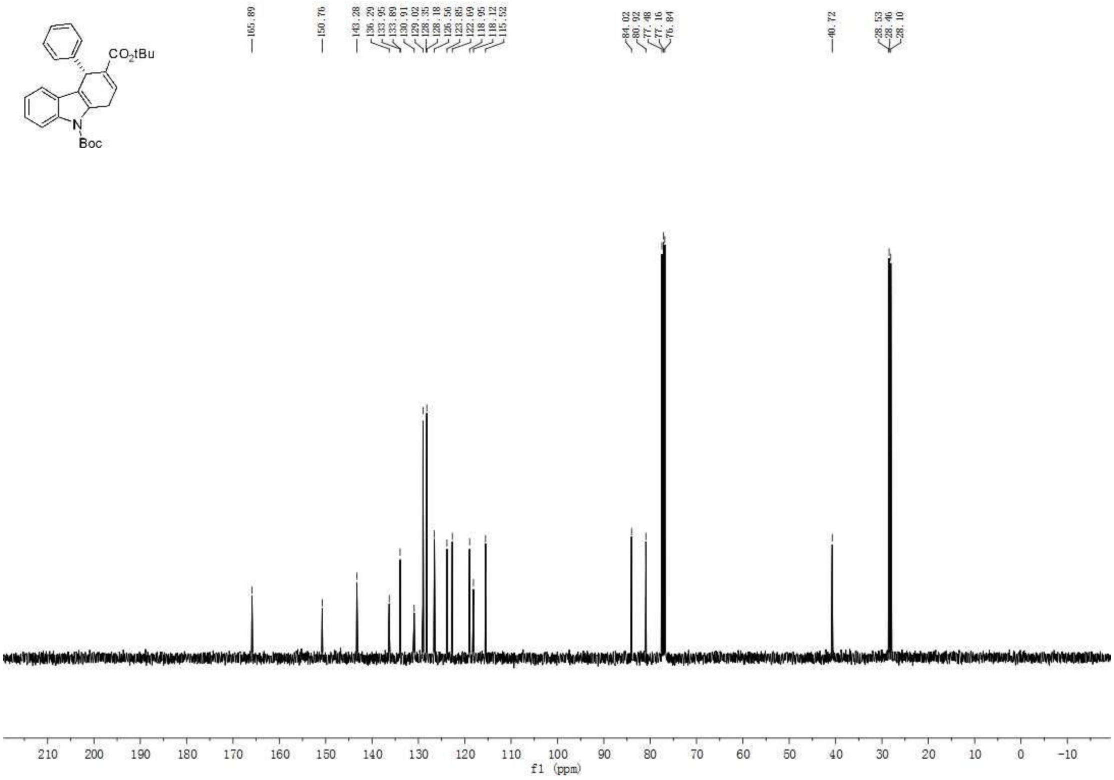

Figure S56. ^{1}H NMR spectrum of **6a**, related to **Scheme 3**.

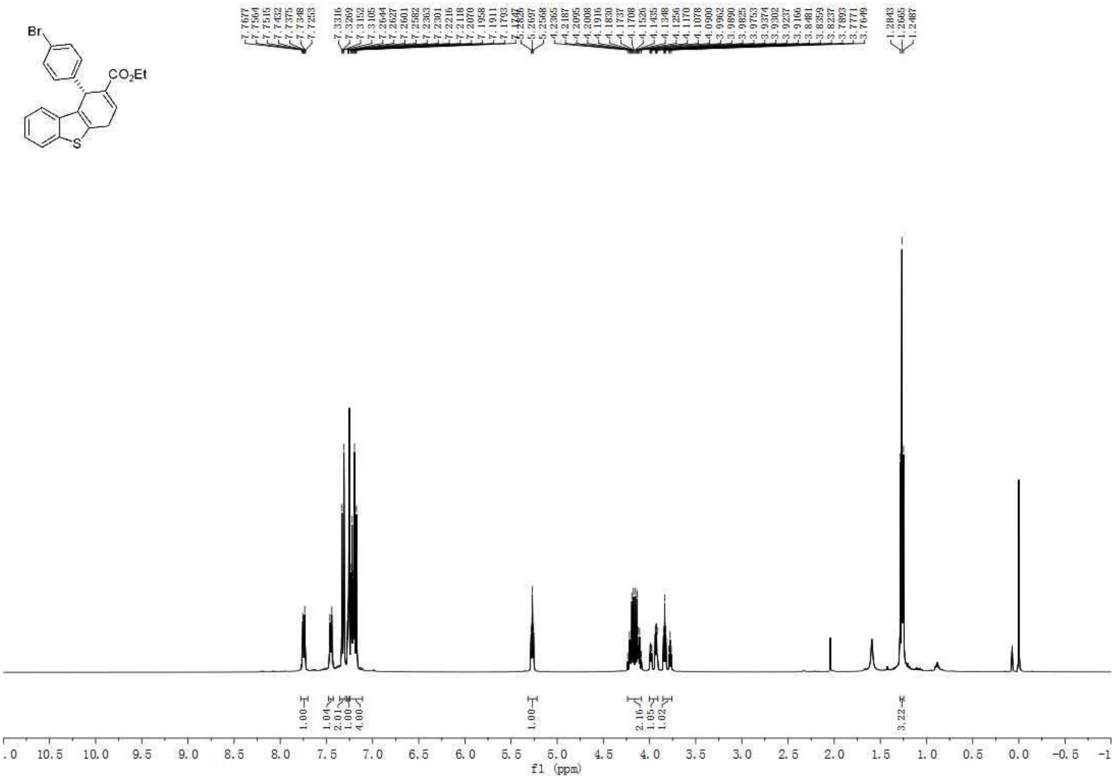

Figure S57. ^{13}C NMR spectrum of **6a**, related to **Scheme 3**.

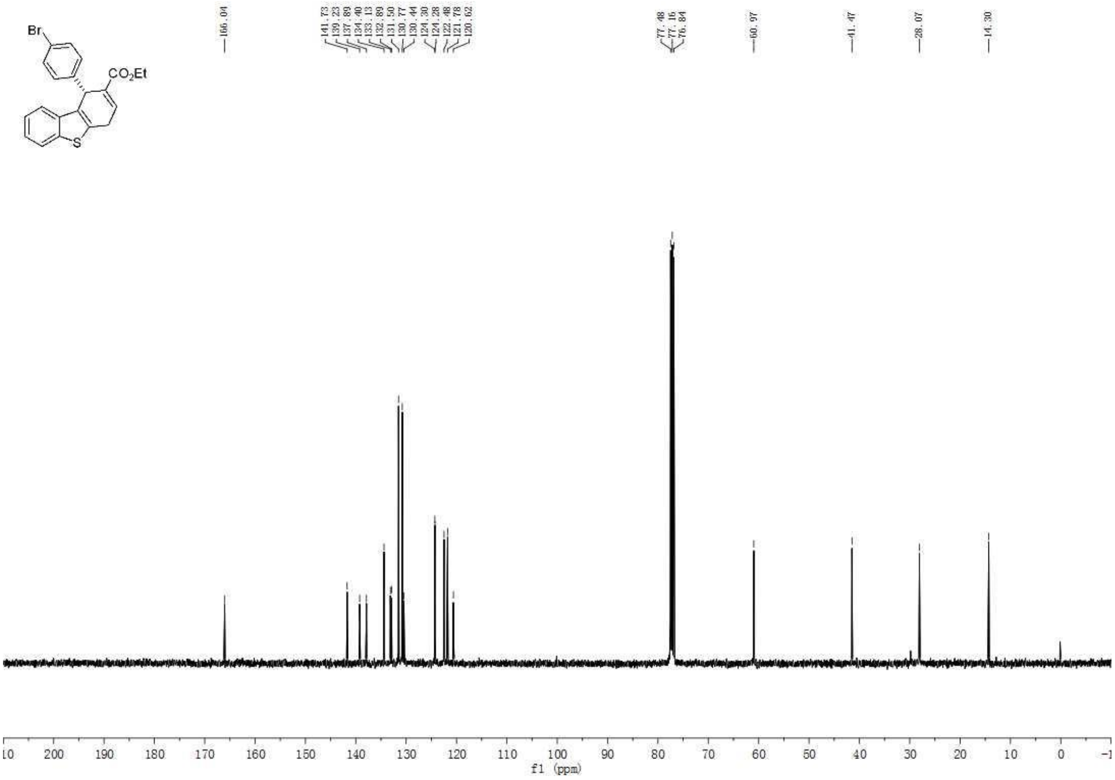

Figure S58. ^{1}H NMR spectrum of **6b**, related to **Scheme 3**.

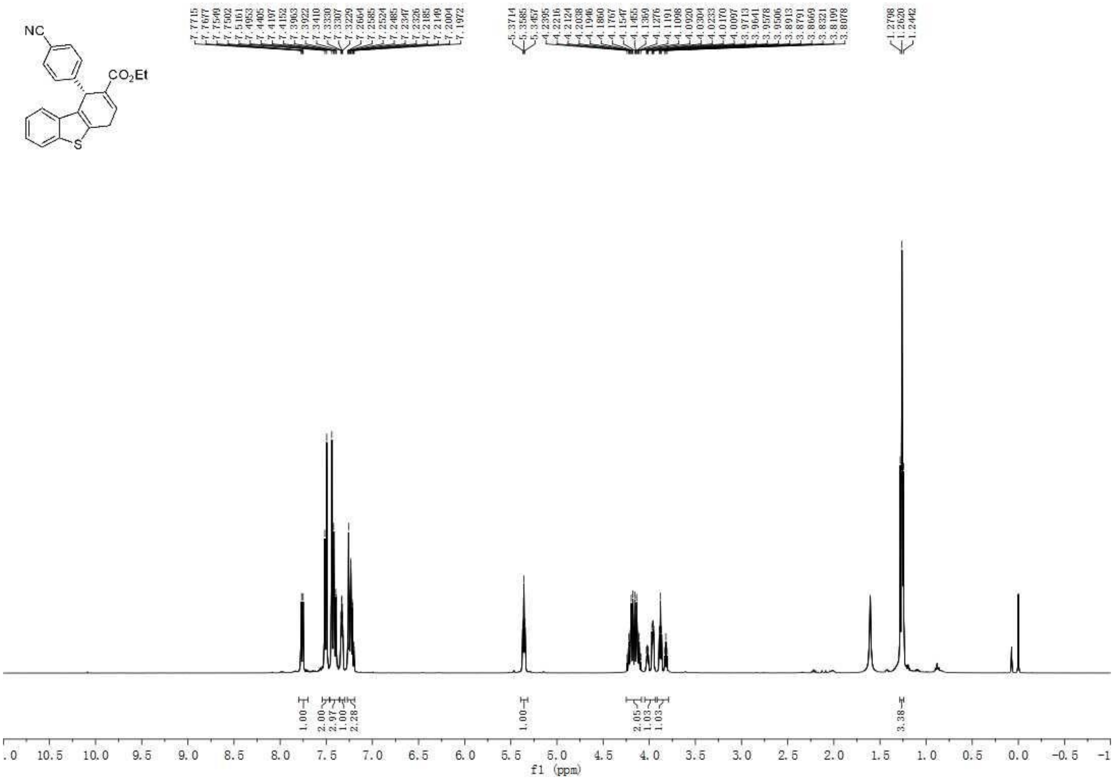

Figure S59. ^{13}C NMR spectrum of **6b**, related to **Scheme 3**.

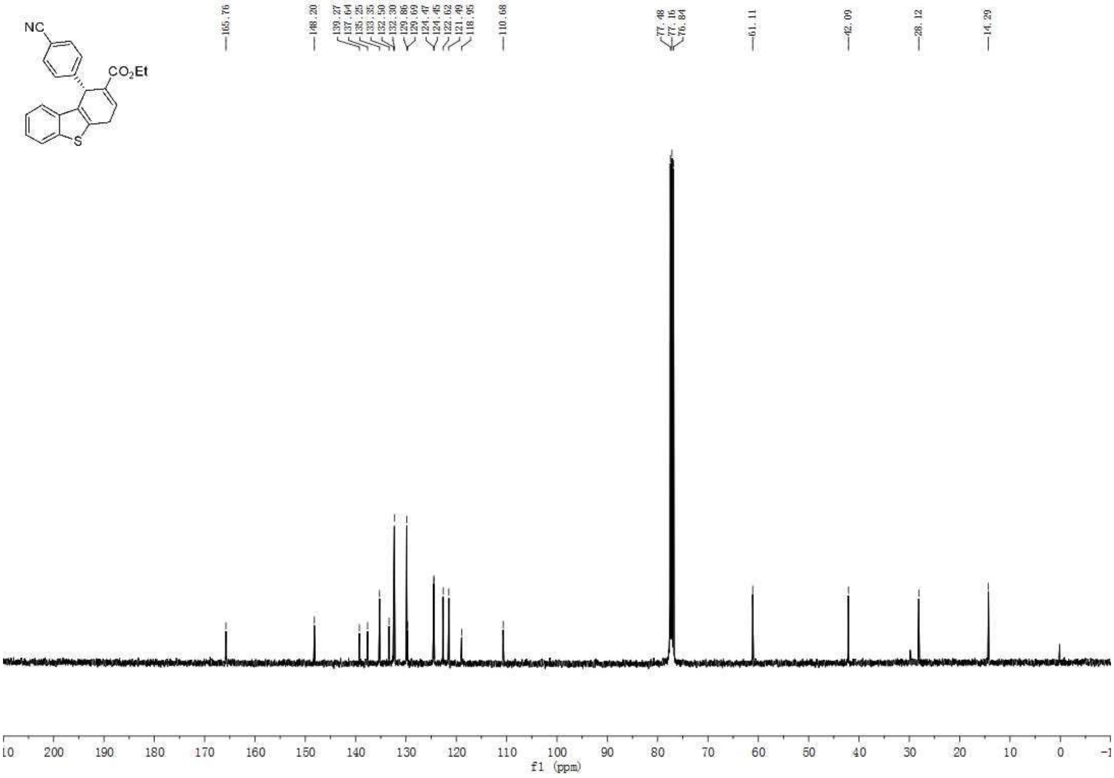

Figure S60. ^{1}H NMR spectrum of **6c**, related to **Scheme 3**.

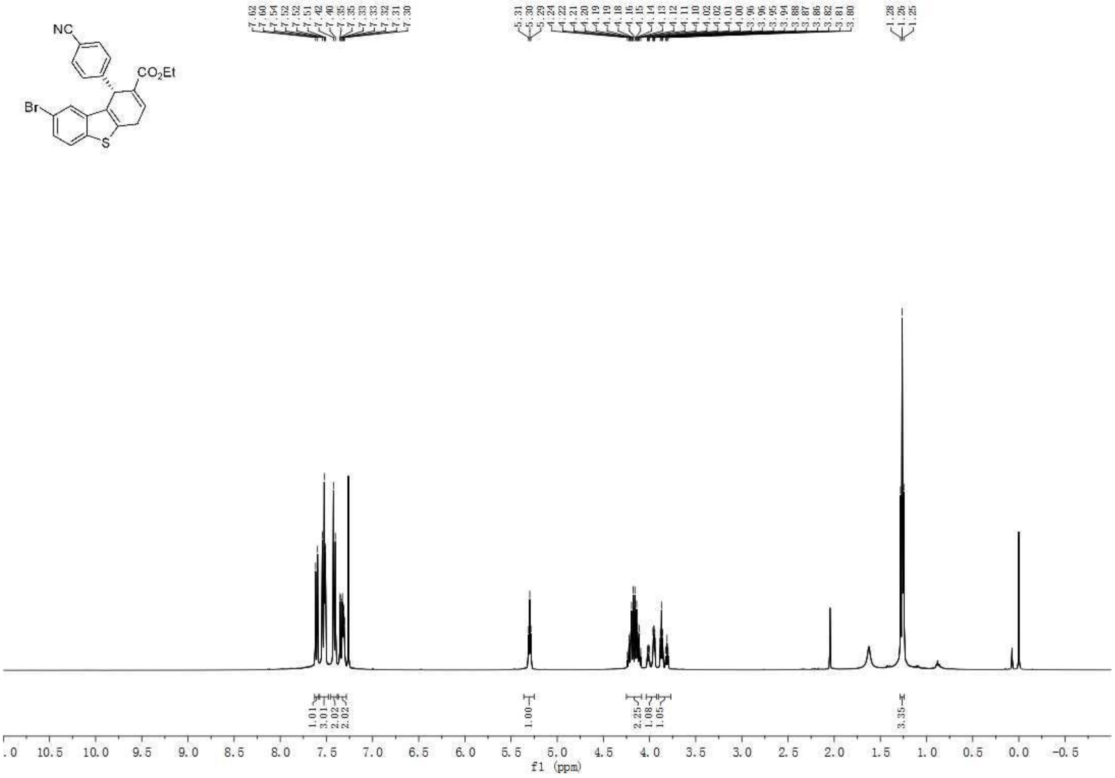

Figure S61. ^{13}C NMR spectrum of **6c**, related to **Scheme 3**.

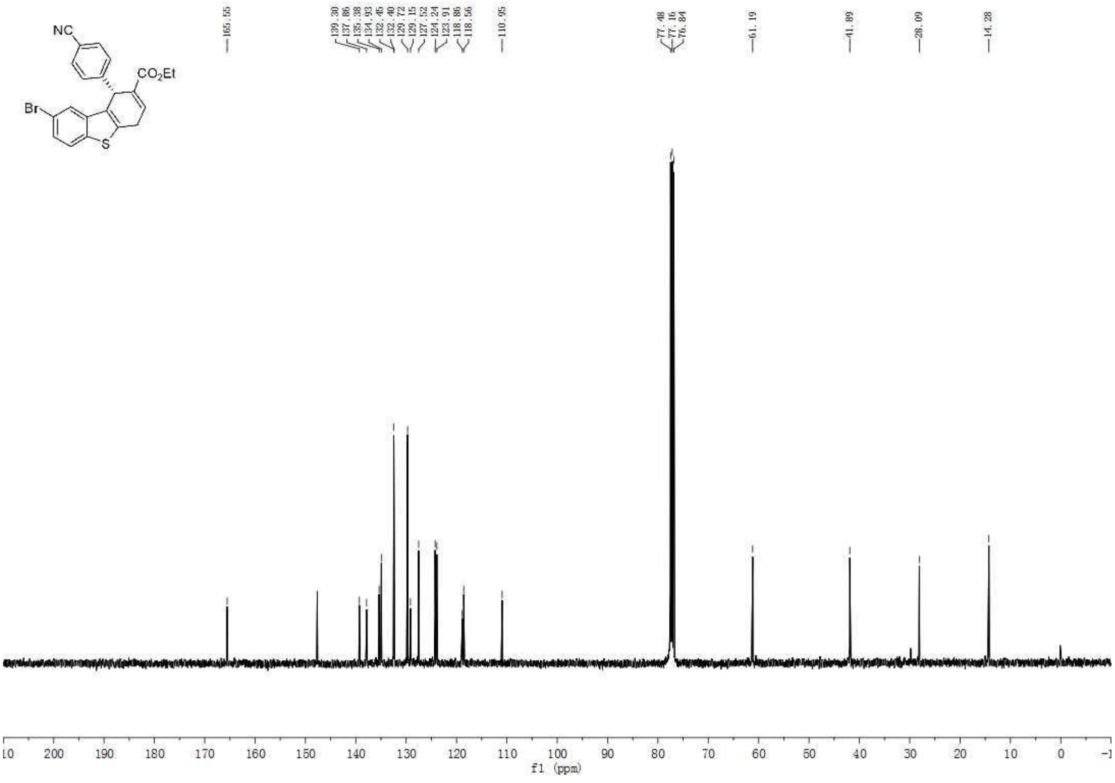

Figure S62. ^{1}H NMR spectrum of **6d,** related to **Scheme 3**.

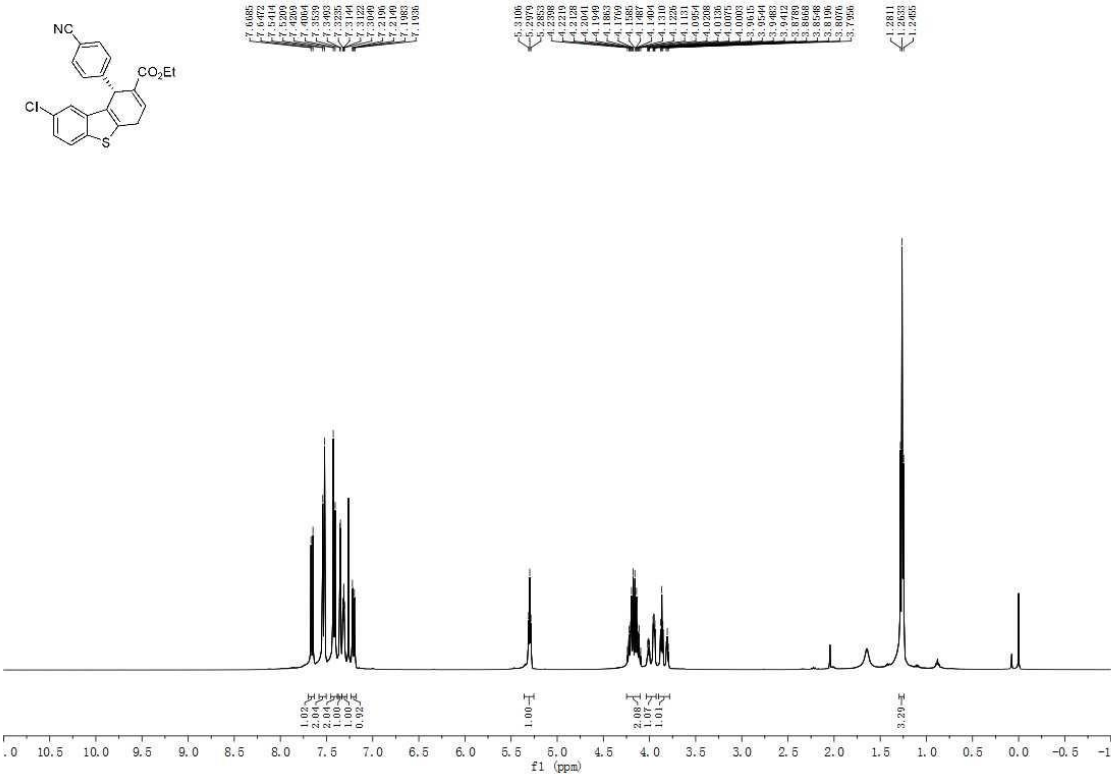

Figure S63. ^{13}C NMR spectrum of **6d**, related to **Scheme 3**.

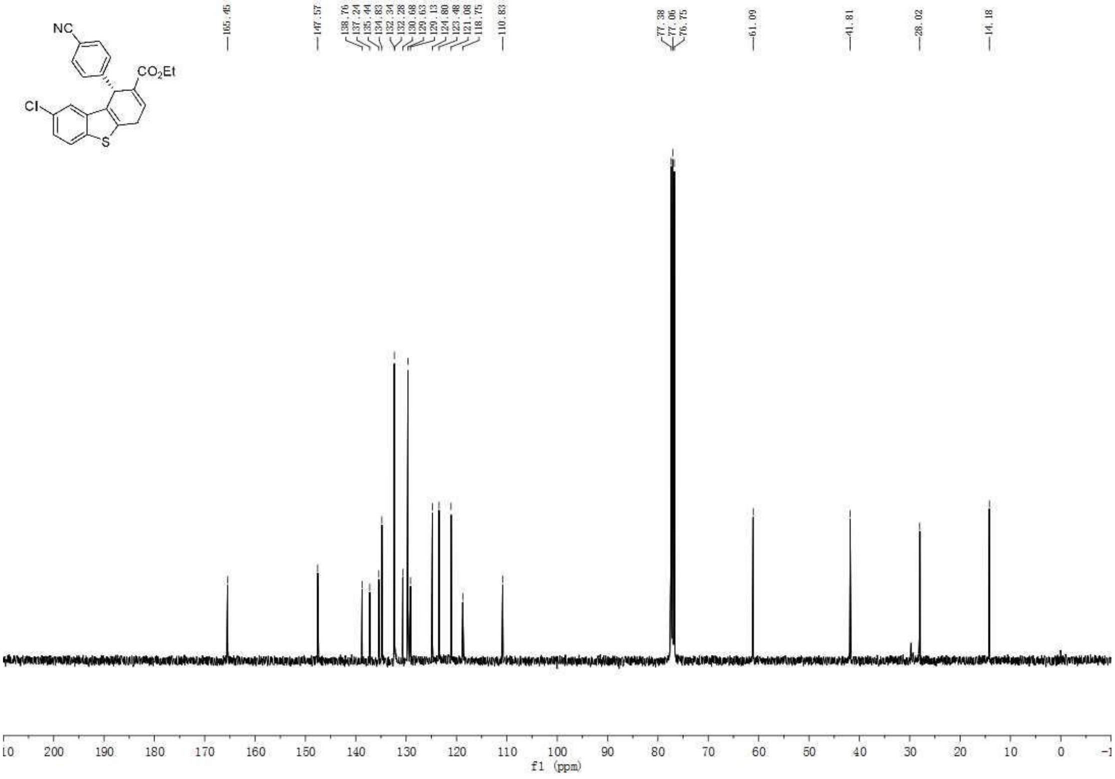

Figure S64. ^{1}H NMR spectrum of **6e,** related to **Scheme 3**.

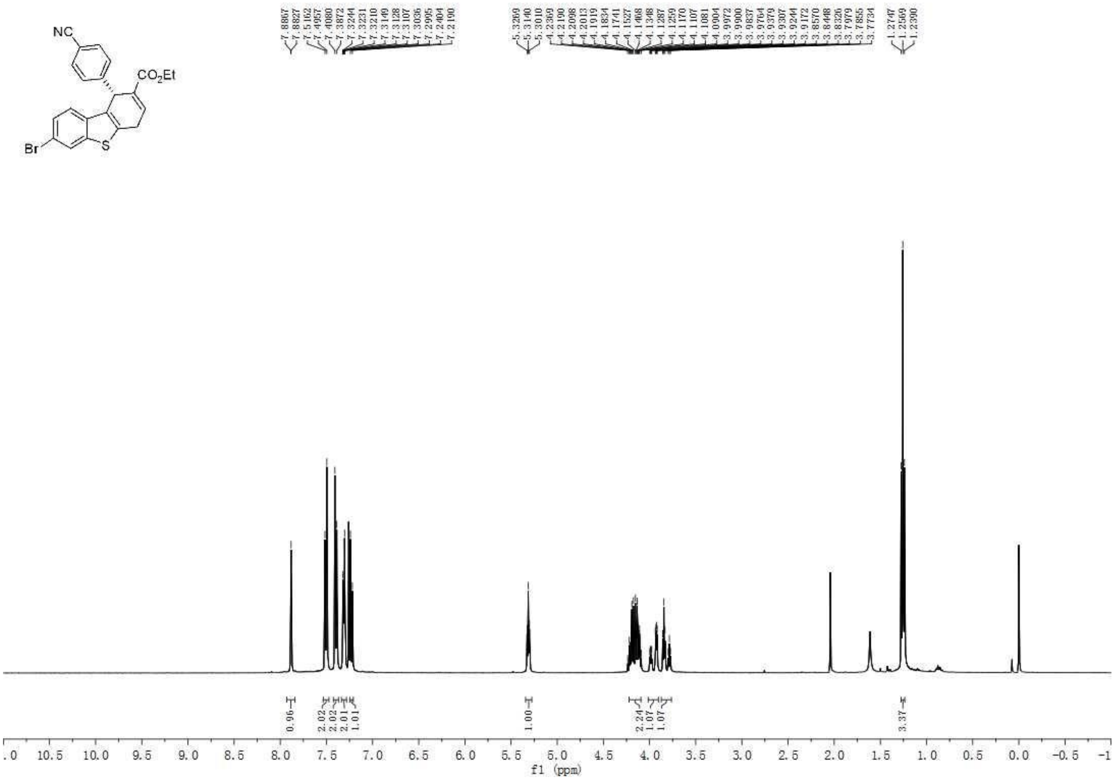

Figure S65. ^{13}C NMR spectrum of **6e**, related to **Scheme 3**.

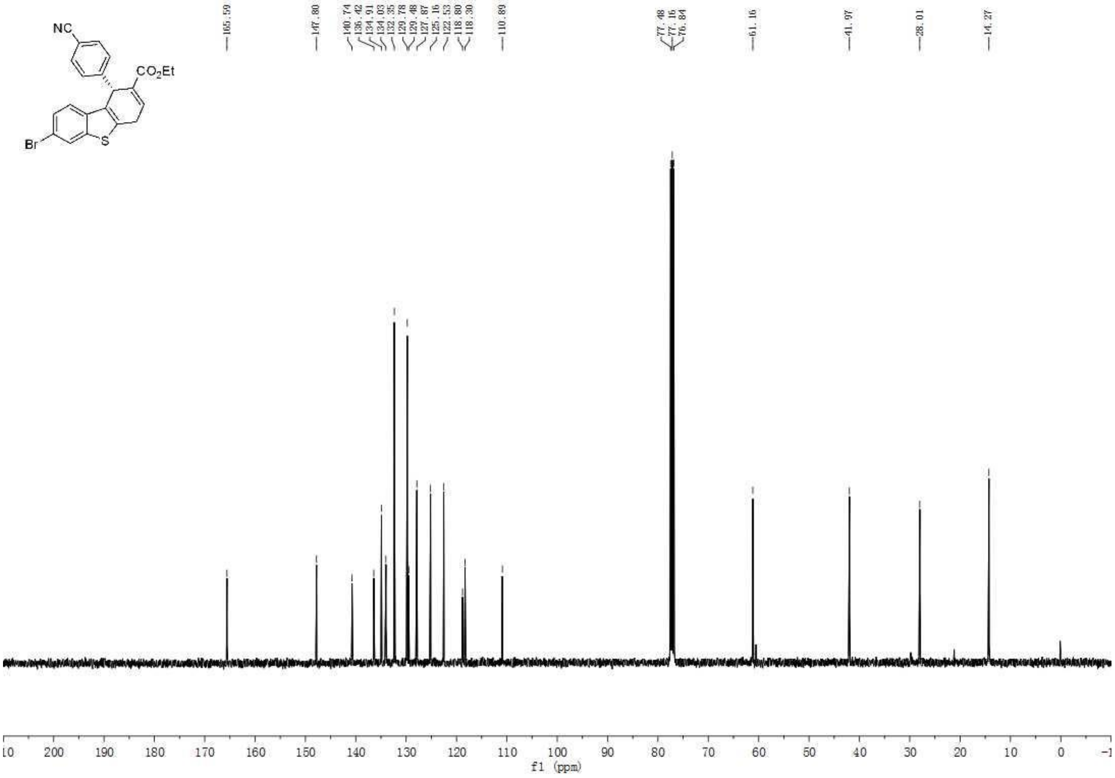

Figure S66. ^{1}H NMR spectrum of **6f,** related to **Scheme 3**.

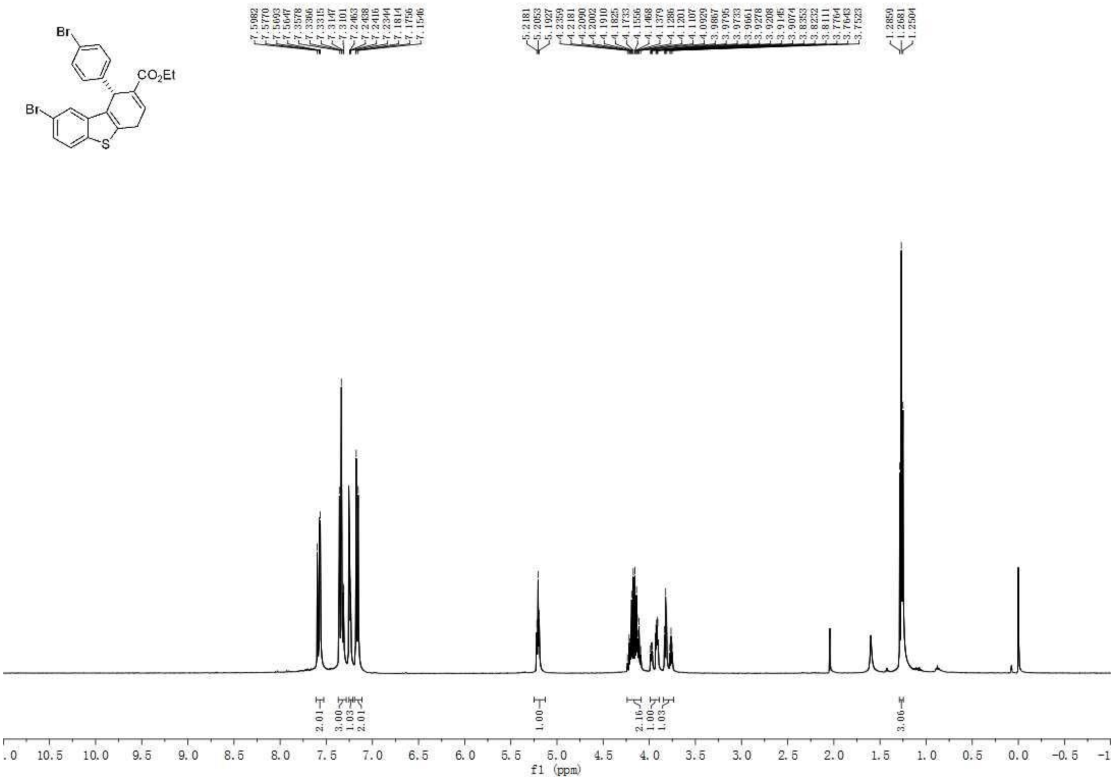

Figure S67. ^{13}C NMR spectrum of **6f**, related to **Scheme 3**.

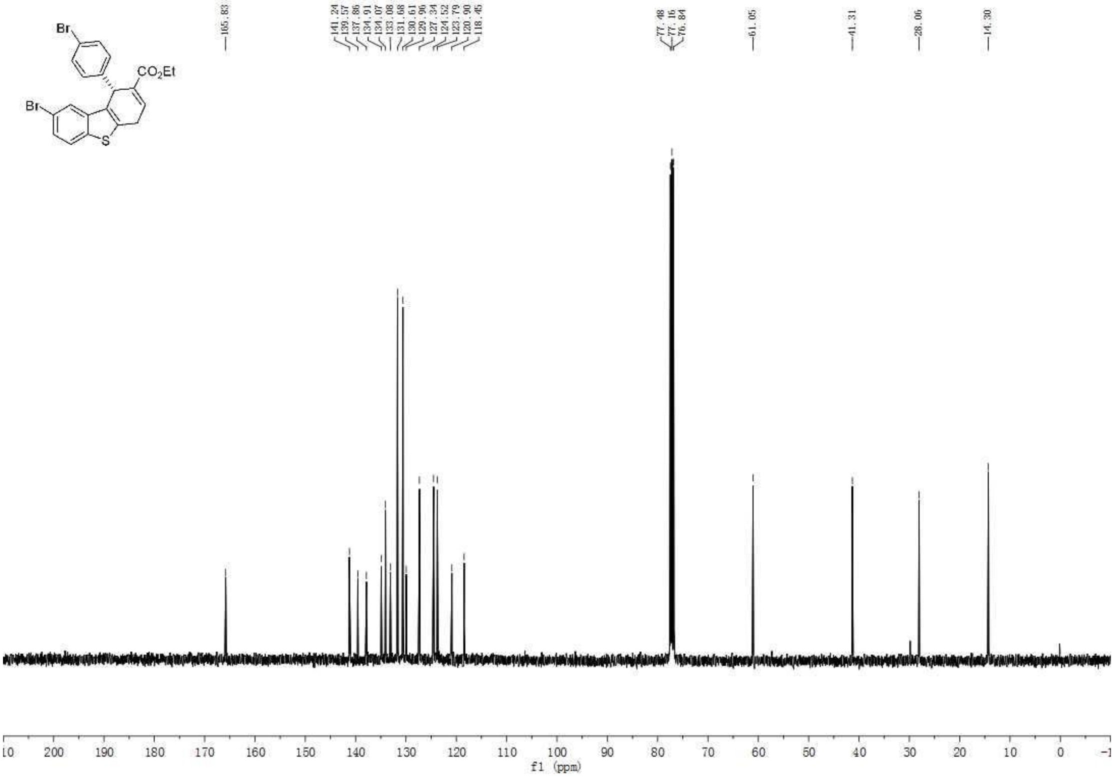

Figure S68. ^{1}H NMR spectrum of **6g**, related to **Scheme 3**.

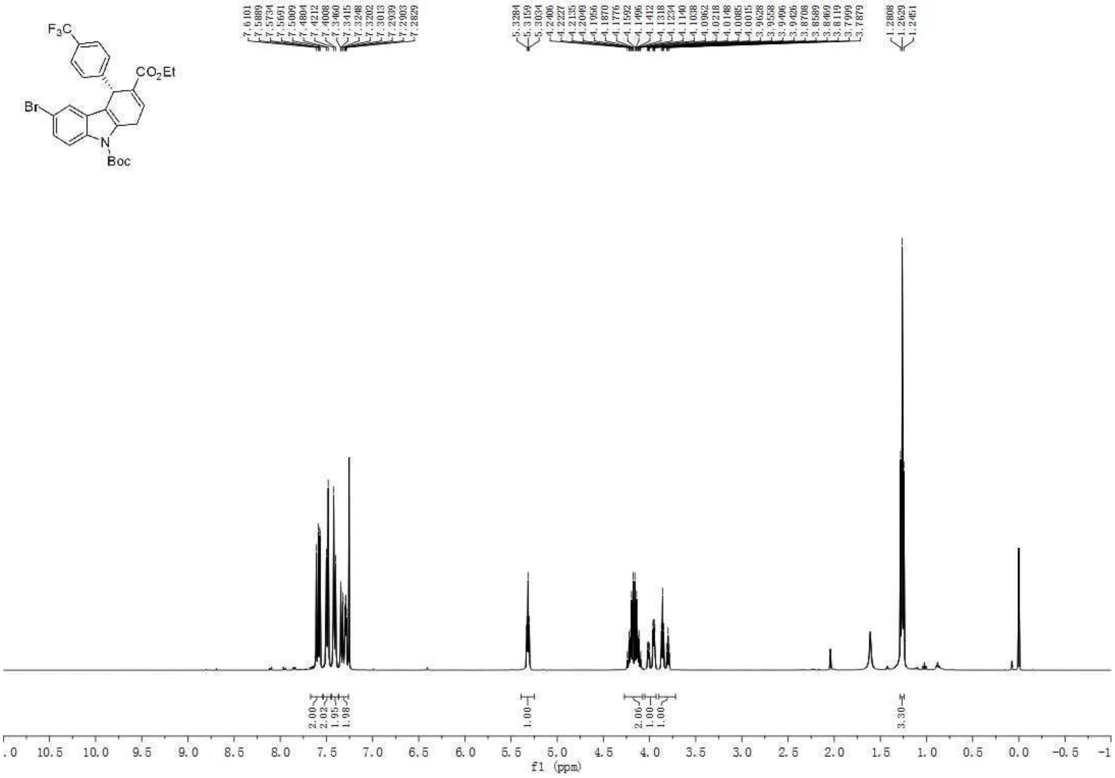

Figure S69. ^{13}C NMR spectrum of **6g**, related to **Scheme 3**.

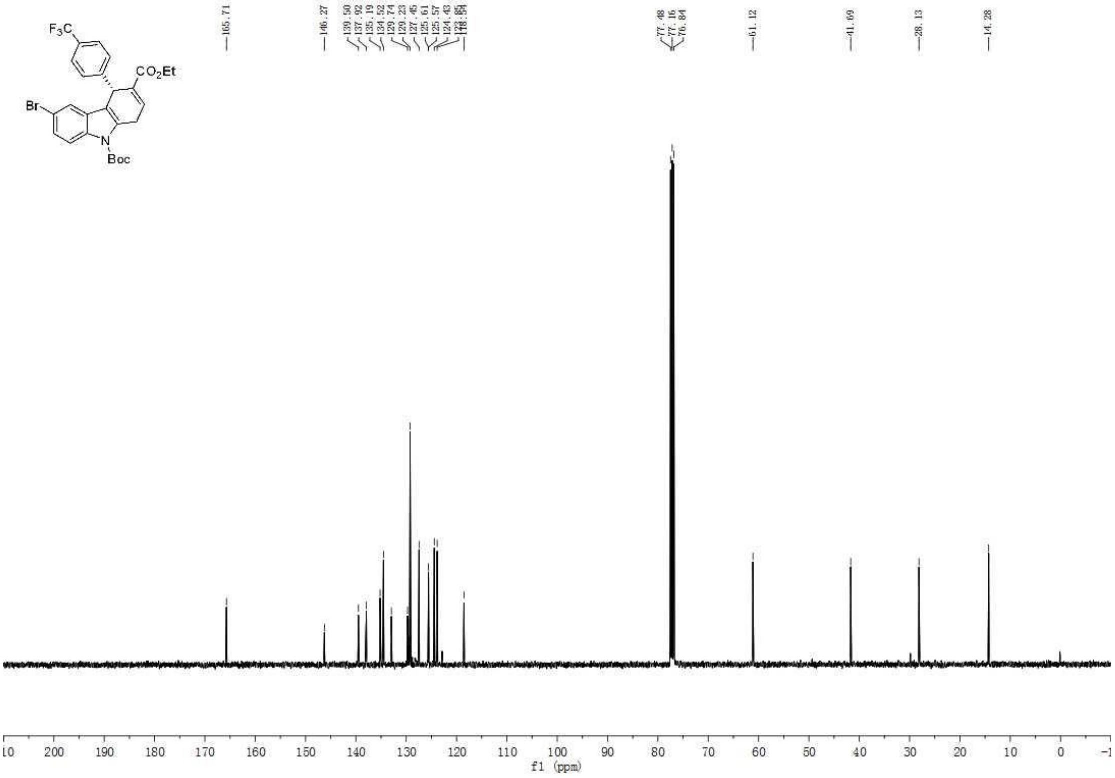

Figure S70. ^{19}F NMR spectrum of **6g**, related to **Scheme 3**.

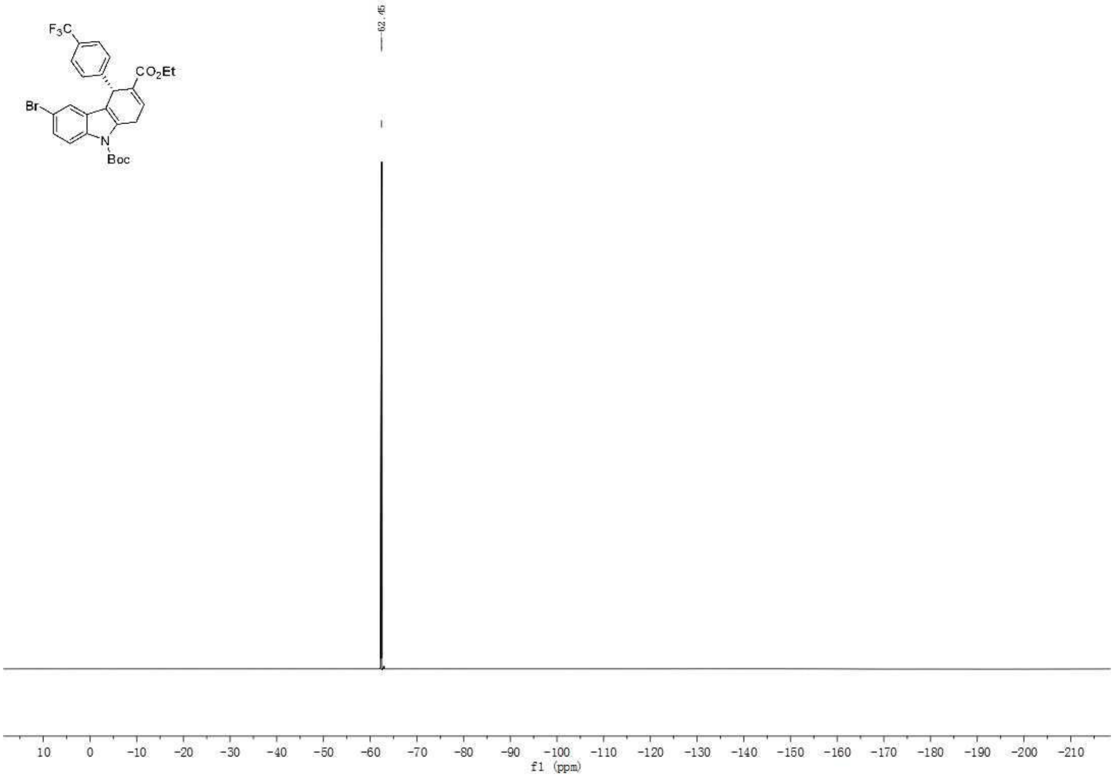

Figure S71. ^{1}H NMR spectrum of **6h**, related to **Scheme 3**.

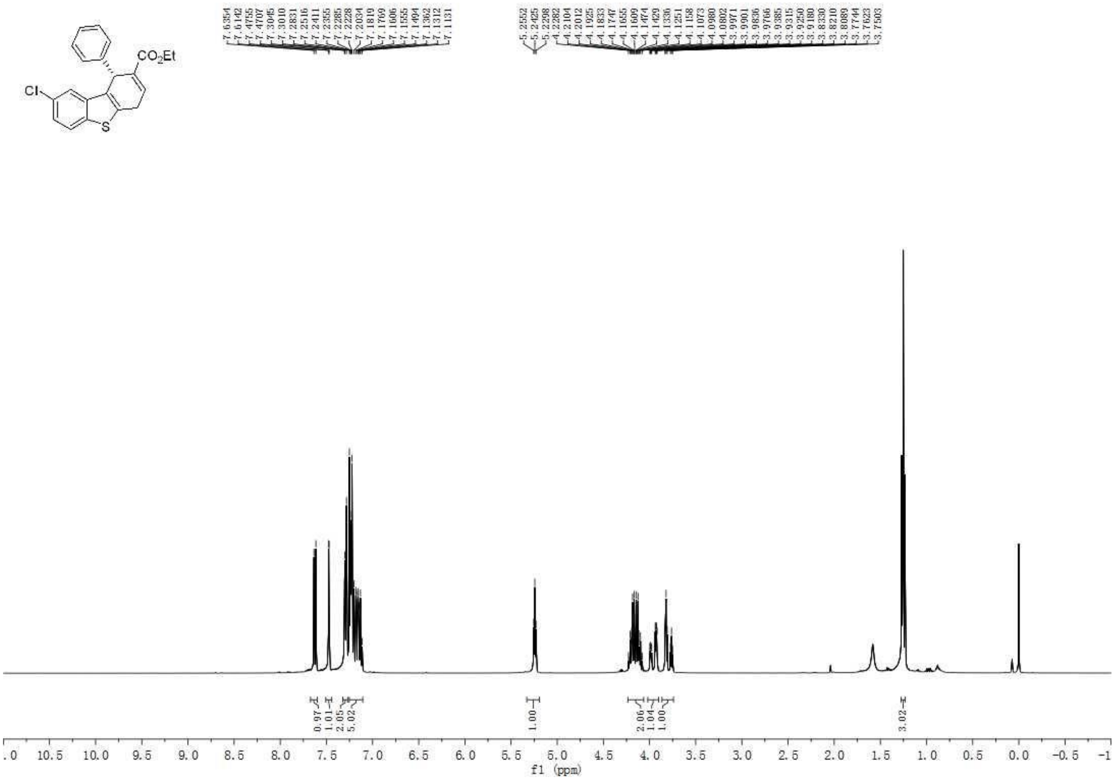

Figure S72. ^{13}C NMR spectrum of **6h**, related to **Scheme 3**.

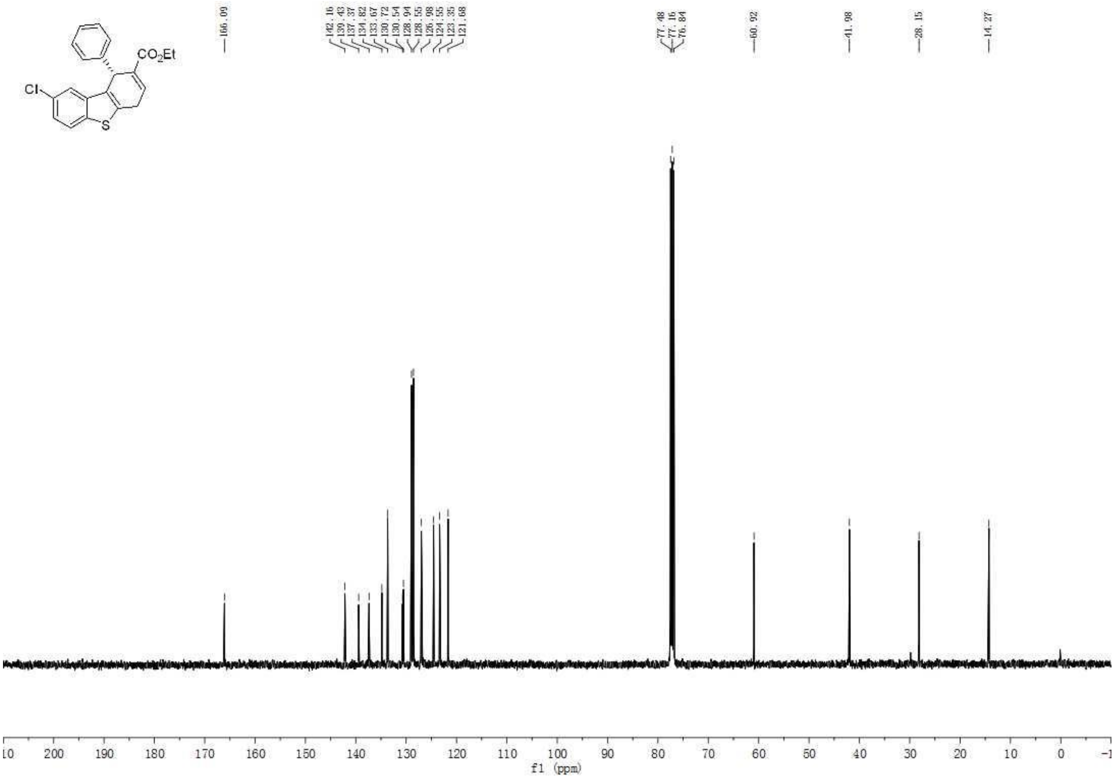

Figure S73. ^{1}H NMR spectrum of **7**, related to **Scheme 4.**

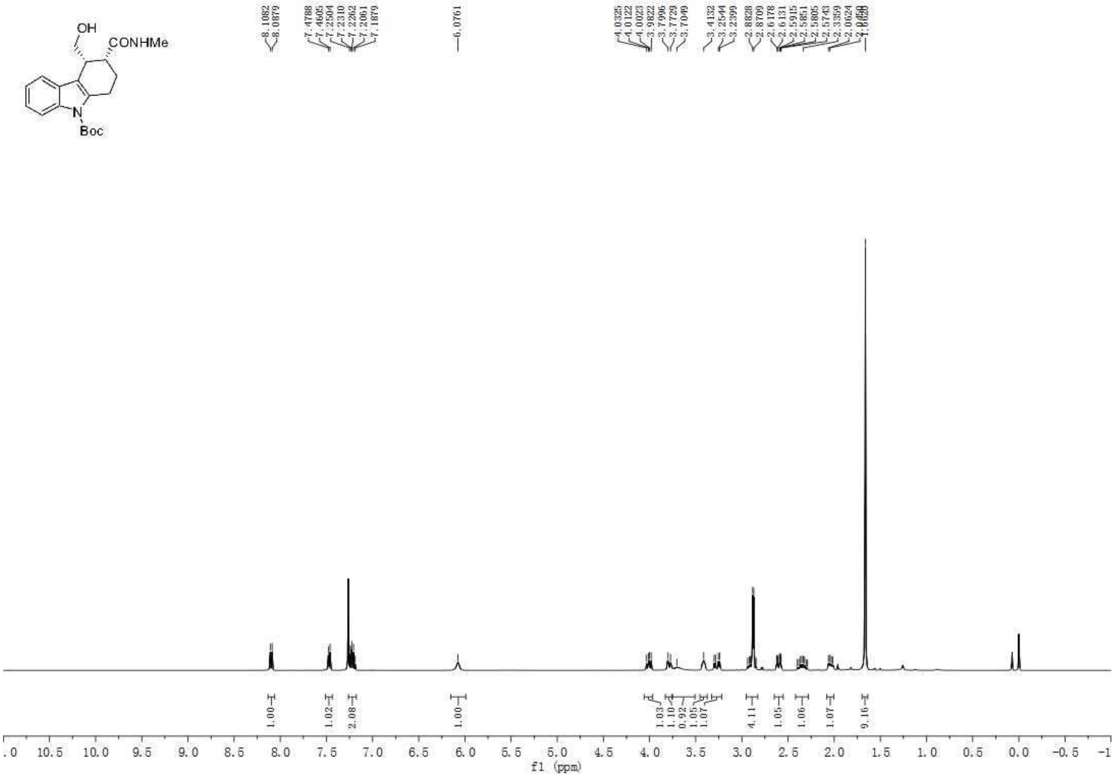

Figure S74. ^{13}C NMR spectrum of **7**, related to **Scheme 4**.

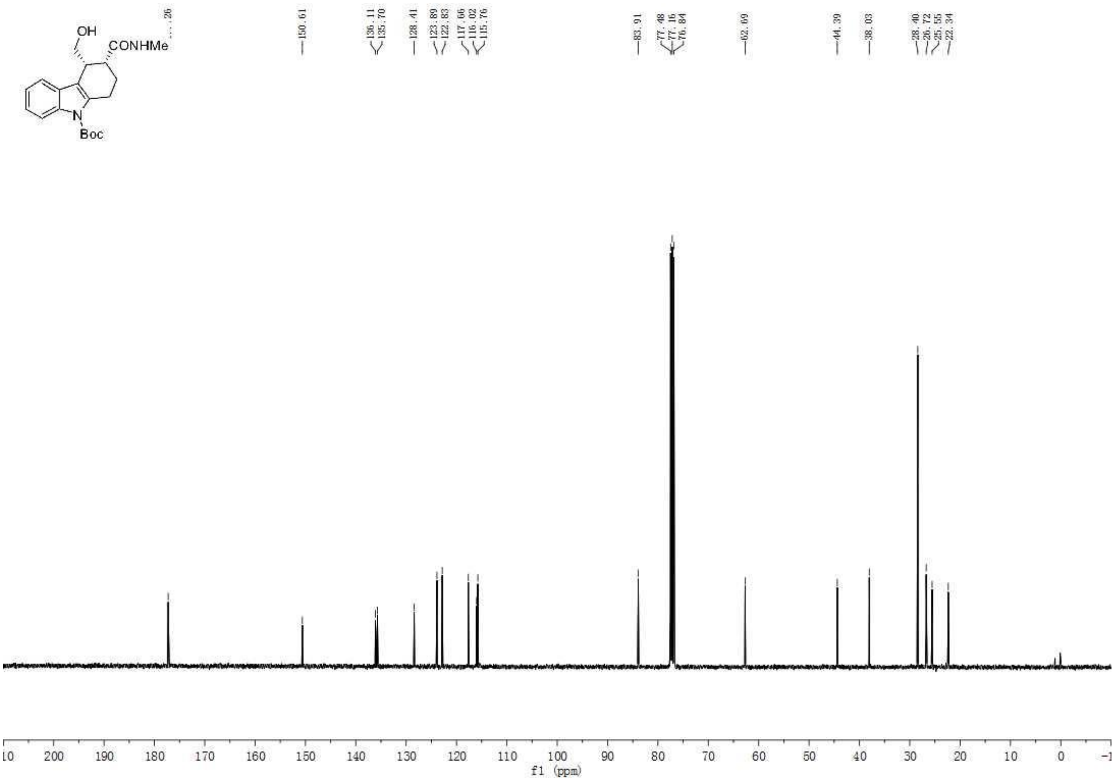

Figure S75. ^{1}H NMR spectrum of **8**, related to **Scheme 4.**

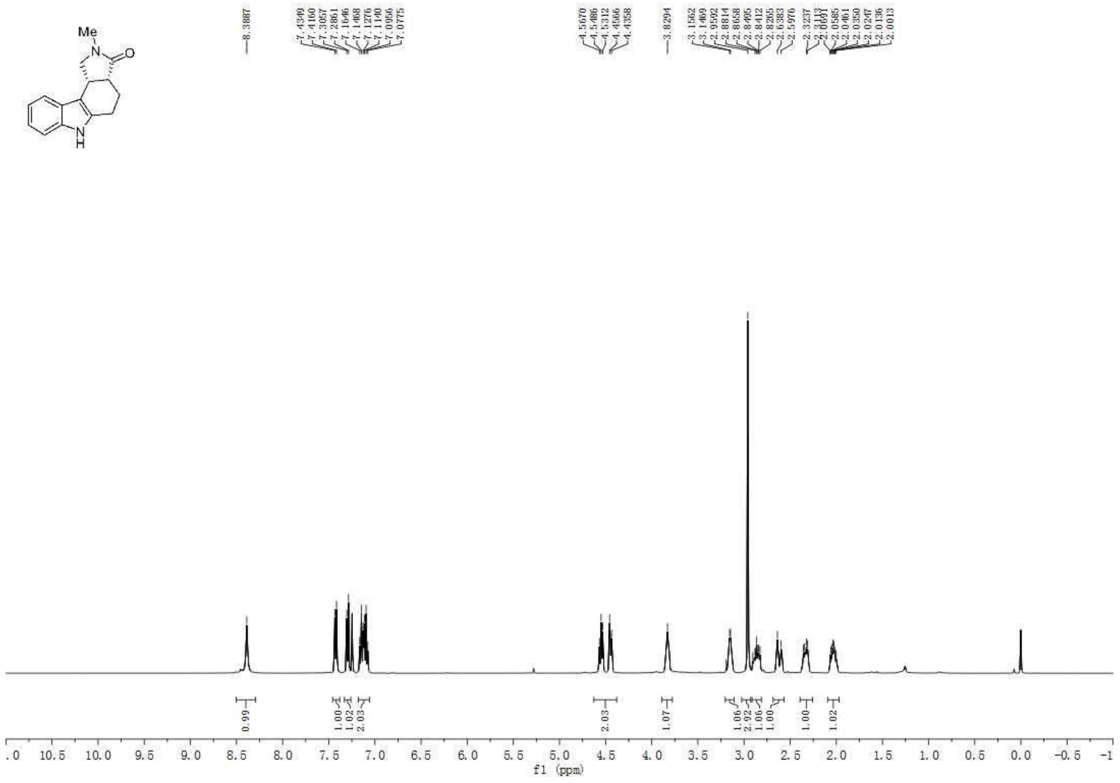

Figure S76. ^{13}C NMR spectrum of **8**, related to **Scheme 4**.

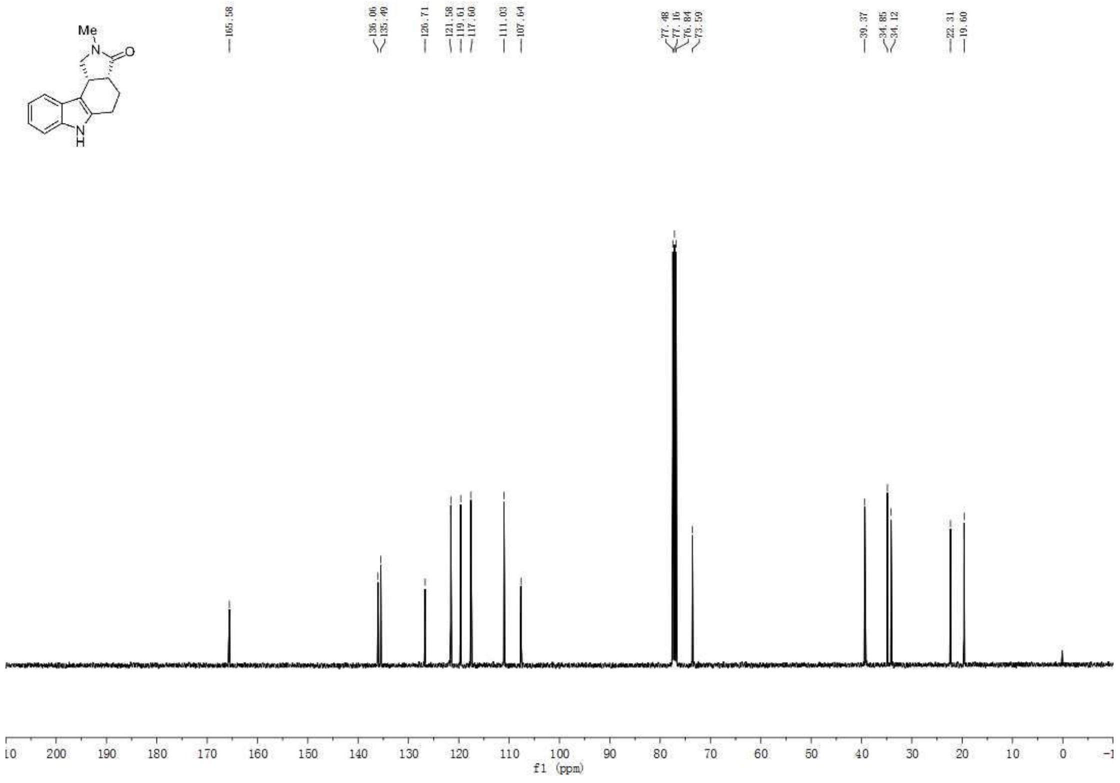

Figure S77. ^{1}H NMR spectrum of **9**, related to **Scheme 4.**

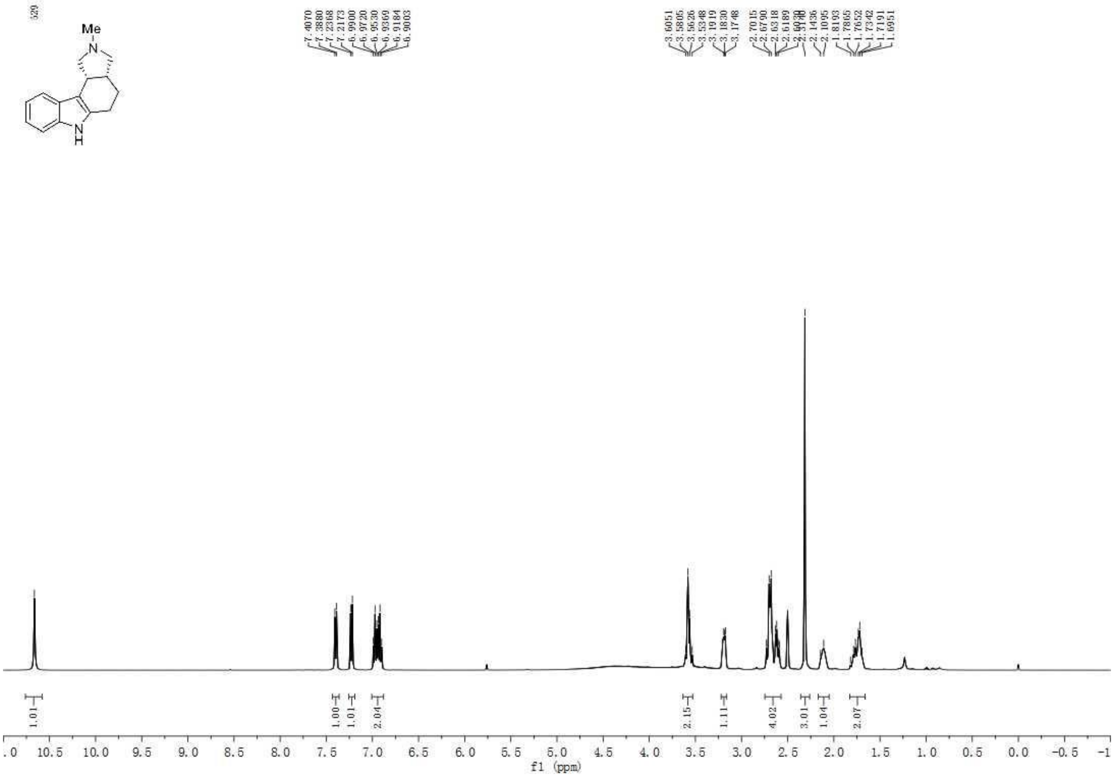

Figure S78. ^{13}C NMR spectrum of **9**, related to **Scheme 4**.

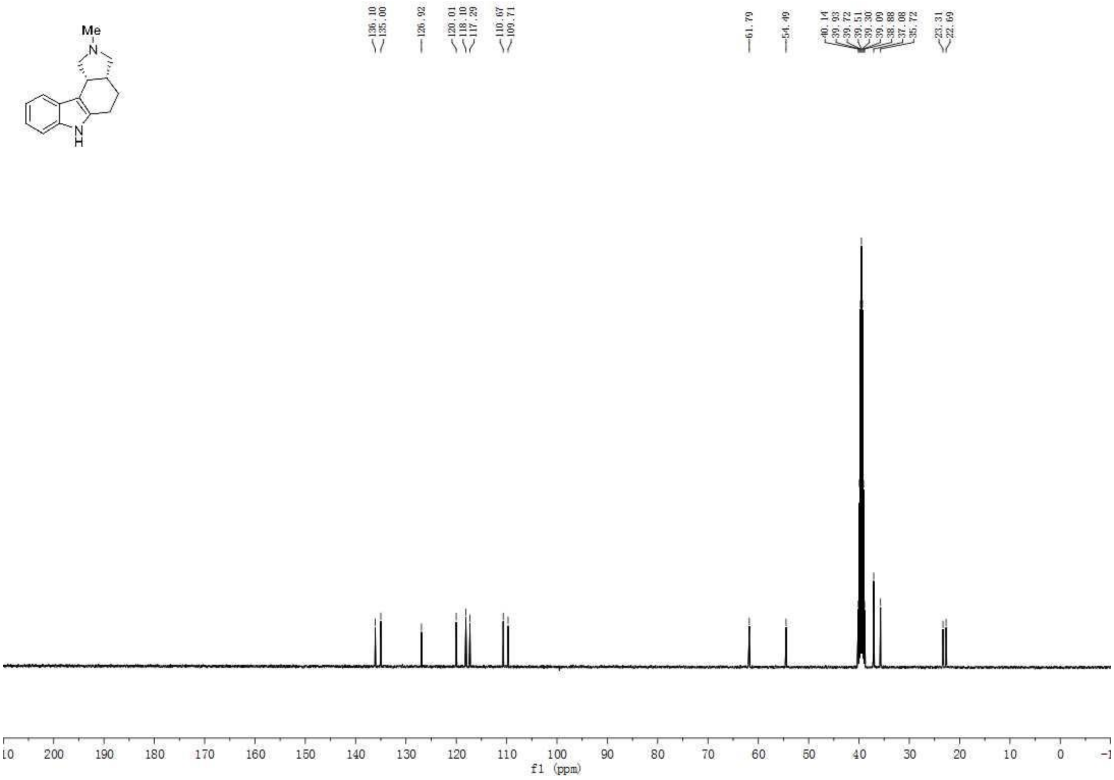

Figure S79. ^{1}H NMR spectrum of **10**, related to **Table 1.**

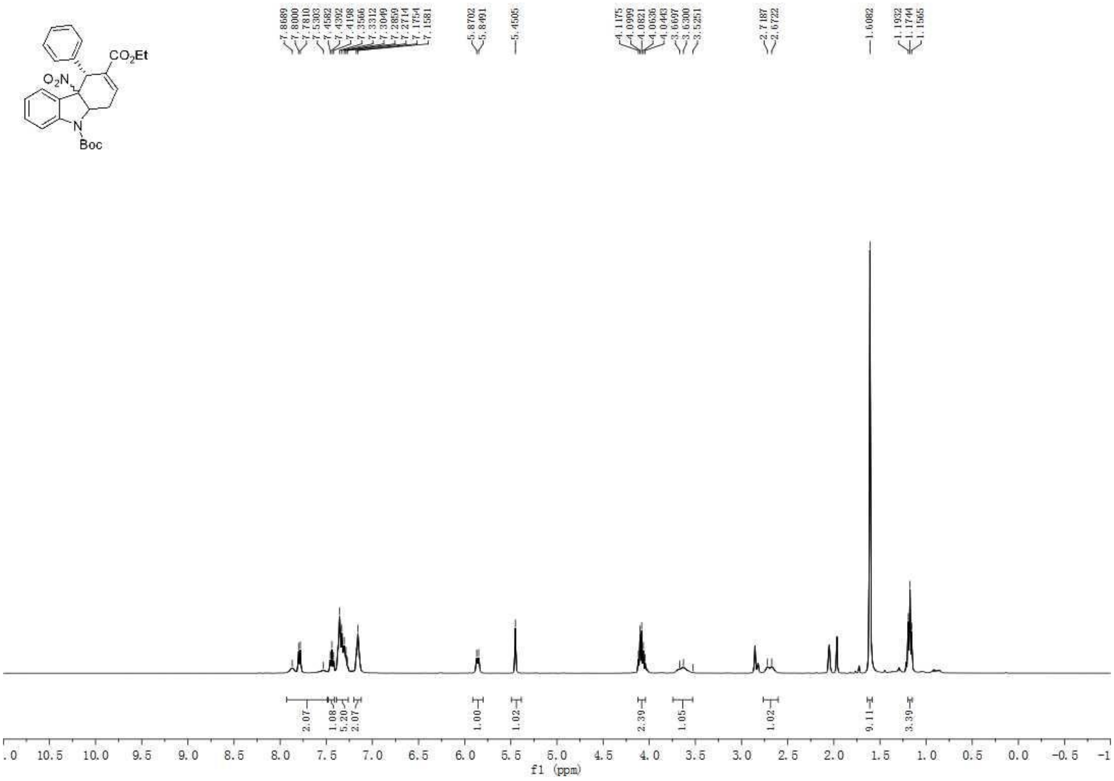

Figure S80. ^{13}C NMR spectrum of **10**, related to **Table 1**.

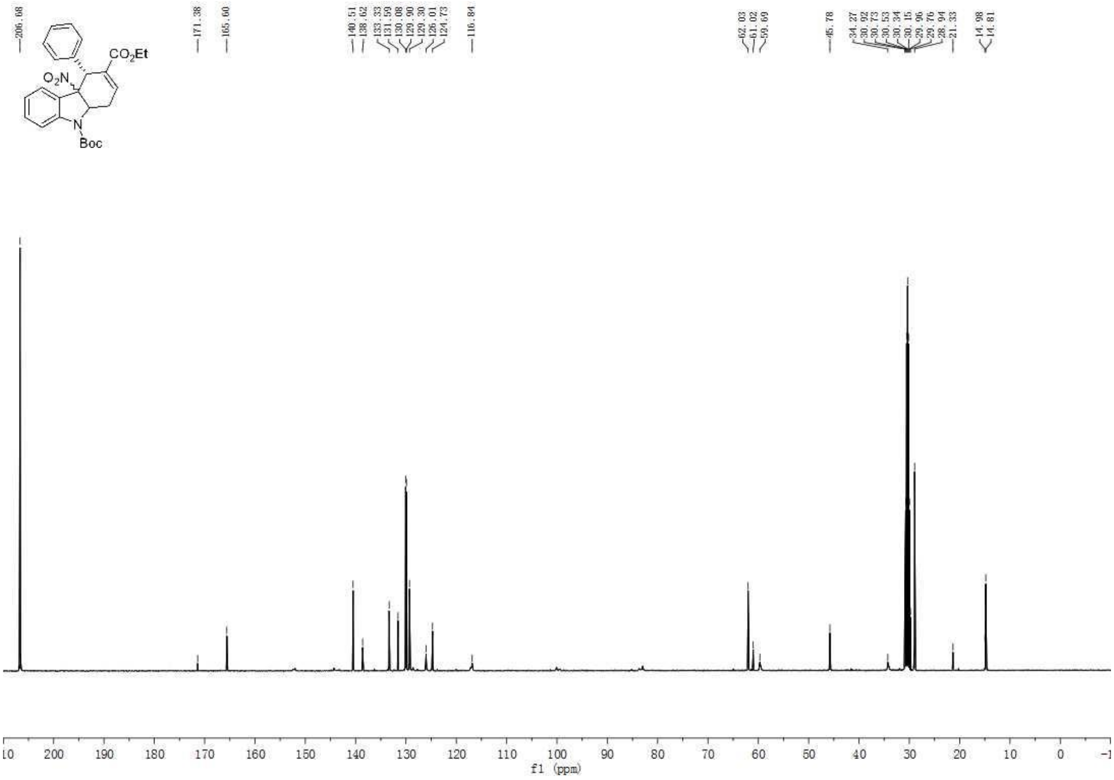

Supplemental Figures for HPLC spectra

Figure S81. HPLC spectra of *rac*-**3a,** related to **Scheme 2**.

SAMPLE INFORMATION

Sample Name:	why-g05-72-5-rac-IA-3%	Acquired By:	System
Sample Type:	Unknown	Sample Set Name	
Vial:	61	Acq. Method Set:	3%
Injection #:	2	Processing Method	5 72 5 RAC
Injection Volume:	10.00 ul	Channel Name:	2998 Ch1 254nm@1.2nm
Run Time:	100.0 Minutes	Proc. Chnl. Descr.:	2998 Ch1 254nm@1.2nm
Date Acquired:	9/24/2019 2:39:18 PM CST		
Date Processed:	9/24/2019 2:53:07 PM CST		

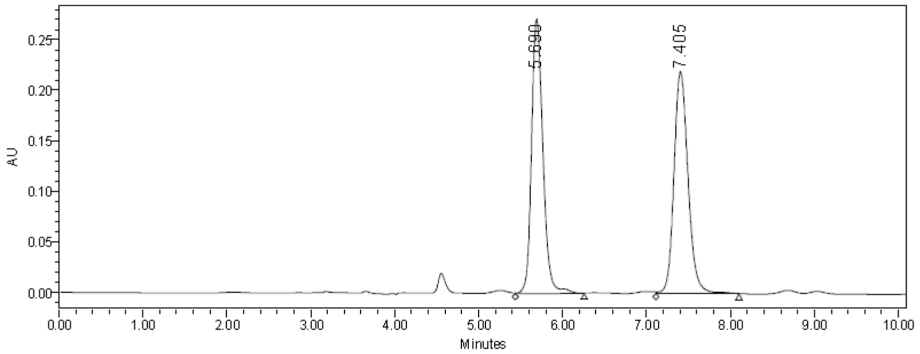

	RT	Area	% Area	Height
1	5.690	2560919	50.07	271284
2	7.405	2554055	49.93	219595

Figure S82. HPLC spectra of **3a,** related to **Scheme 2**.

SAMPLE INFORMATION

Sample Name:	why-g05-72-5-IA-3%	Acquired By:	System
Sample Type:	Unknown	Sample Set Name	
Vial:	37	Acq. Method Set:	3%
Injection #:	1	Processing Method	5 72 5
Injection Volume:	10.00 ul	Channel Name:	2998 Ch1 254nm@1.2nm
Run Time:	100.0 Minutes	Proc. Chnl. Descr.:	2998 Ch1 254nm@1.2nm
Date Acquired:	4/13/2019 4:32:02 PM CST		
Date Processed:	7/19/2019 4:19:16 PM CST		

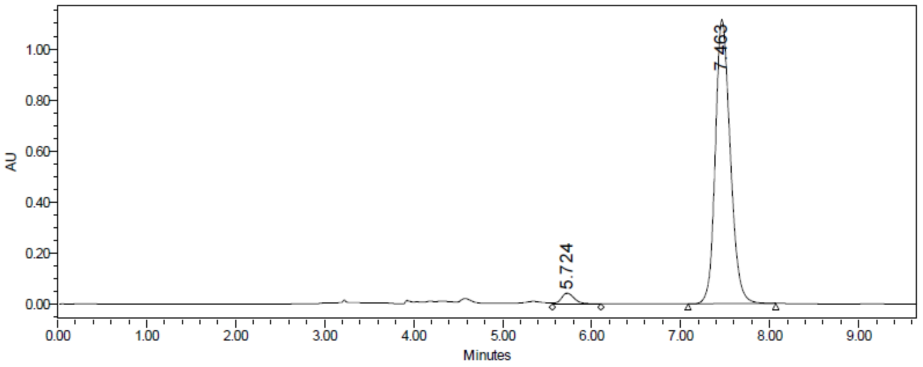

	RT	Area	% Area	Height
1	5.724	434402	3.24	42393
2	7.463	12978962	96.76	1111415

Figure S83. HPLC spectra of *rac*-**3b**, related to **Scheme 2**.

SAMPLE INFORMATION

Sample Name:	why-g05-102-1-IA-3%	Acquired By:	System
Sample Type:	Unknown	Sample Set Name	
Vial:	45	Acq. Method Set:	3%
Injection #:	1	Processing Method	5 102 1
Injection Volume:	10.00 ul	Channel Name:	2998 Ch1 254nm@1.2nm
Run Time:	100.0 Minutes	Proc. Chnl. Descr.:	2998 Ch1 254nm@1.2nm
Date Acquired:	5/5/2019 4:48:39 PM CST		
Date Processed:	7/19/2019 4:24:54 PM CST		

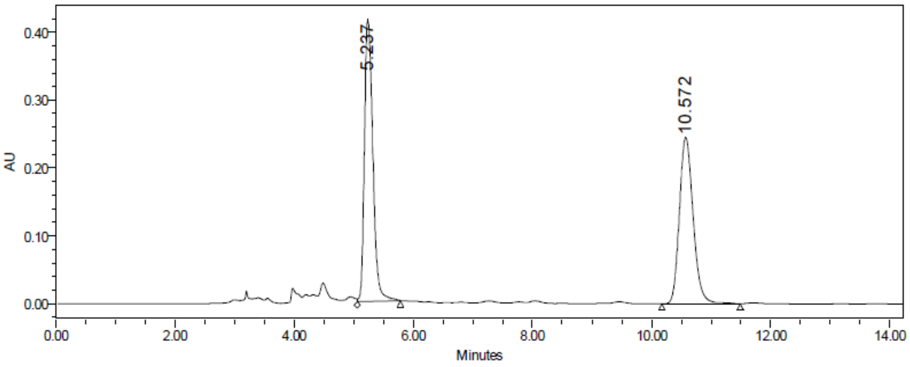

	RT	Area	% Area	Height
1	5.237	4069692	50.92	415006
2	10.572	3922670	49.08	245275

Figure S84. HPLC spectra of **3b**, related to **Scheme 2**.

SAMPLE INFORMATION

Sample Name:	why-g05-102-2-IA-3%	Acquired By:	System
Sample Type:	Unknown	Sample Set Name	
Vial:	80	Acq. Method Set:	3%
Injection #:	1	Processing Method	5 102 2
Injection Volume:	10.00 ul	Channel Name:	2998 Ch1 254nm@1.2nm
Run Time:	100.0 Minutes	Proc. Chnl. Descr.:	2998 Ch1 254nm@1.2nm
Date Acquired:	5/7/2019 3:50:53 PM CST		
Date Processed:	9/23/2019 8:46:36 PM CST		

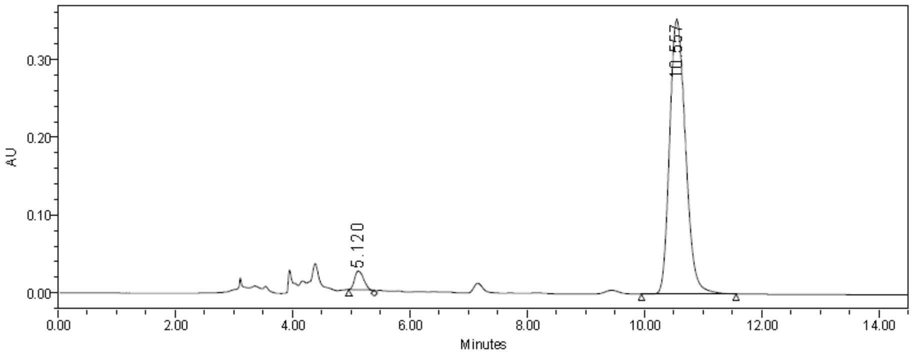

	RT	Area	% Area	Height
1	5.120	260126	3.78	24045
2	10.557	6618616	96.22	352534

Figure S85. HPLC spectra of *rac*-**3c**, related to **Scheme 2**.

SAMPLE INFORMATION

Sample Name:	why-g05-118-1-IA-1.5%	Acquired By:	System
Sample Type:	Unknown	Sample Set Name	
Vial:	7	Acq. Method Set:	1 5%
Injection #:	1	Processing Method	5 118 1
Injection Volume:	10.00 ul	Channel Name:	2998 Ch1 254nm@1.2nm
Run Time:	100.0 Minutes	Proc. Chnl. Descr.:	2998 Ch1 254nm@1.2nm
Date Acquired:	5/23/2019 10:26:54 AM CST		
Date Processed:	7/19/2019 4:27:37 PM CST		

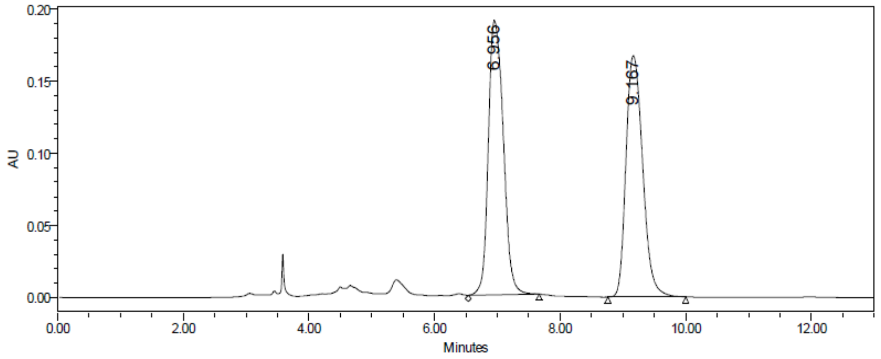

	RT	Area	% Area	Height
1	6.956	3151355	50.36	189896
2	9.167	3106347	49.64	167240

Figure S86. HPLC spectra of **3c**, related to **Scheme 2**.

SAMPLE INFORMATION

Sample Name:	why-g05-118-2-IA-1.5%	Acquired By:	System
Sample Type:	Unknown	Sample Set Name	
Vial:	43	Acq. Method Set:	1 5%
Injection #:	1	Processing Method	5 118 2
Injection Volume:	10.00 ul	Channel Name:	2998 Ch1 254nm@1.2nm
Run Time:	100.0 Minutes	Proc. Chnl. Descr.:	2998 Ch1 254nm@1.2nm
Date Acquired:	5/23/2019 10:41:01 AM CST		
Date Processed:	7/19/2019 4:28:16 PM CST		

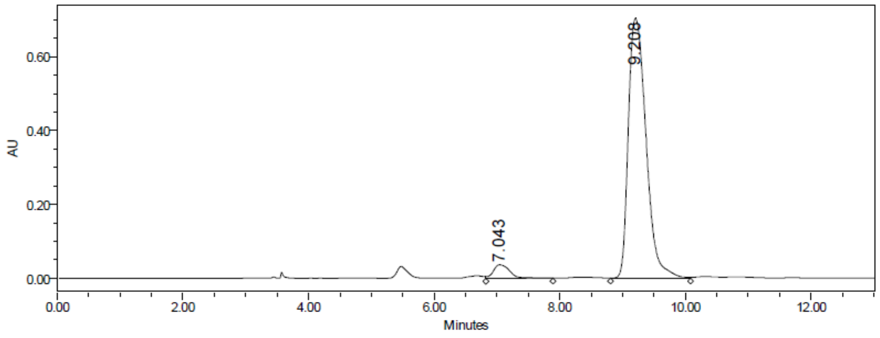

	RT	Area	% Area	Height
1	7.043	663366	4.57	36756
2	9.208	13837500	95.43	702659

Figure S87. HPLC spectra of *rac*-**3d**, related to **Scheme 2**.

SAMPLE INFORMATION

Sample Name:	why-g05-100-1-IA-1.5%	Acquired By:	System
Sample Type:	Unknown	Sample Set Name	
Vial:	29	Acq. Method Set:	1 5%
Injection #:	1	Processing Method	5 100 1
Injection Volume:	10.00 ul	Channel Name:	2998 Ch1 254nm@1.2nm
Run Time:	100.0 Minutes	Proc. Chnl. Descr.:	2998 Ch1 254nm@1.2nm
Date Acquired:	5/6/2019 8:44:04 PM CST		
Date Processed:	7/19/2019 4:29:17 PM CST		

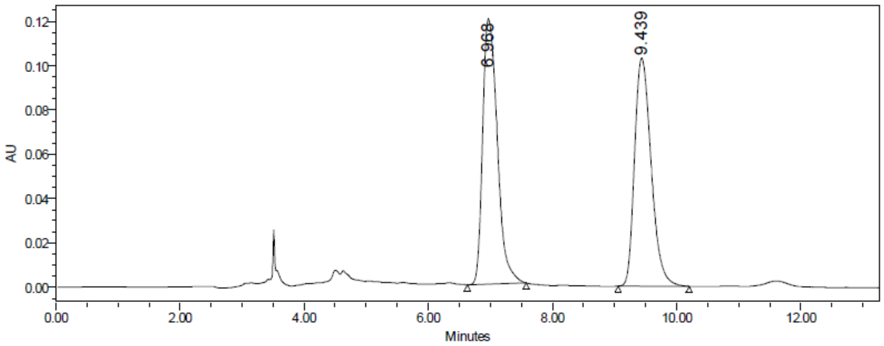

	RT	Area	% Area	Height
1	6.968	1977187	50.74	119534
2	9.439	1919566	49.26	102818

Figure S88. HPLC spectra of **3d**, related to **Scheme 2**.

SAMPLE INFORMATION

Sample Name:	why-g05-100-2-IA-1.5%	Acquired By:	System
Sample Type:	Unknown	Sample Set Name	
Vial:	9	Acq. Method Set:	1 5%
Injection #:	1	Processing Method	5 100 2
Injection Volume:	10.00 ul	Channel Name:	2998 Ch1 254nm@1.2nm
Run Time:	100.0 Minutes	Proc. Chnl. Descr.:	2998 Ch1 254nm@1.2nm
Date Acquired:	5/6/2019 8:58:25 PM CST		
Date Processed:	7/19/2019 4:29:46 PM CST		

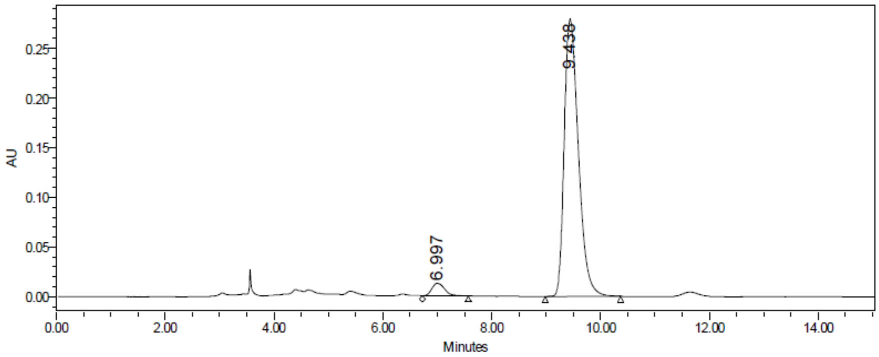

	RT	Area	% Area	Height
1	6.997	207406	3.95	12618
2	9.438	5042981	96.05	278668

Figure S89. HPLC spectra of *rac*-**3e**, related to **Scheme 2**.

SAMPLE INFORMATION

Sample Name:	why-g05-101-1-IA-1.5%	Acquired By:	System
Sample Type:	Unknown	Sample Set Name	
Vial:	37	Acq. Method Set:	1 5%
Injection #:	1	Processing Method	5 101 1
Injection Volume:	10.00 ul	Channel Name:	2998 Ch1 254nm@1.2nm
Run Time:	100.0 Minutes	Proc. Chnl. Descr.:	2998 Ch1 254nm@1.2nm
Date Acquired:	5/4/2019 8:54:53 PM CST		
Date Processed:	7/19/2019 4:31:14 PM CST		

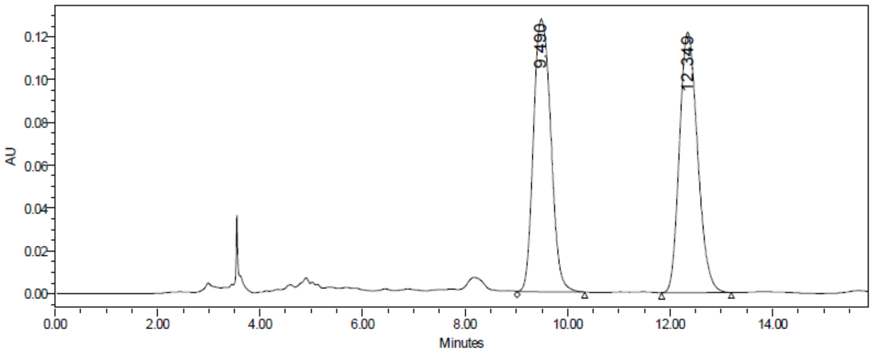

	RT	Area	% Area	Height
1	9.490	3002746	50.01	127223
2	12.349	3001033	49.99	121658

Figure S90. HPLC spectra of **3e**, related to **Scheme 2**.

SAMPLE INFORMATION

Sample Name:	why-g05-101-2-IA-1.5%	Acquired By:	System
Sample Type:	Unknown	Sample Set Name	20190506
Vial:	10	Acq. Method Set:	1 5%
Injection #:	1	Processing Method	5 101 2
Injection Volume:	10.00 ul	Channel Name:	2998 Ch1 254nm@1.2nm
Run Time:	16.0 Minutes	Proc. Chnl. Descr.:	2998 Ch1 254nm@1.2nm
Date Acquired:	5/6/2019 8:06:10 PM CST		
Date Processed:	8/4/2019 2:53:57 PM CST		

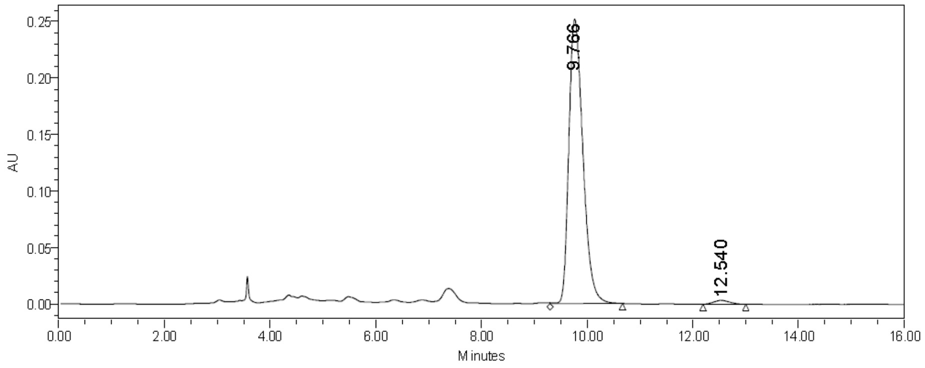

	RT	Area	% Area	Height
1	9.766	4614701	98.50	251558
2	12.540	70049	1.50	3342

Figure S91. HPLC spectra of *rac*-**3f**, related to **Scheme 2**.

SAMPLE INFORMATION

Sample Name:	why-g05-103-1-IA-10%	Acquired By:	System
Sample Type:	Unknown	Sample Set Name	
Vial:	46	Acq. Method Set:	10%
Injection #:	1	Processing Method	5 103 1
Injection Volume:	10.00 ul	Channel Name:	2998 Ch1 254nm@1.2nm
Run Time:	100.0 Minutes	Proc. Chnl. Descr.:	2998 Ch1 254nm@1.2nm
Date Acquired:	5/5/2019 5:09:33 PM CST		
Date Processed:	7/19/2019 4:32:50 PM CST		

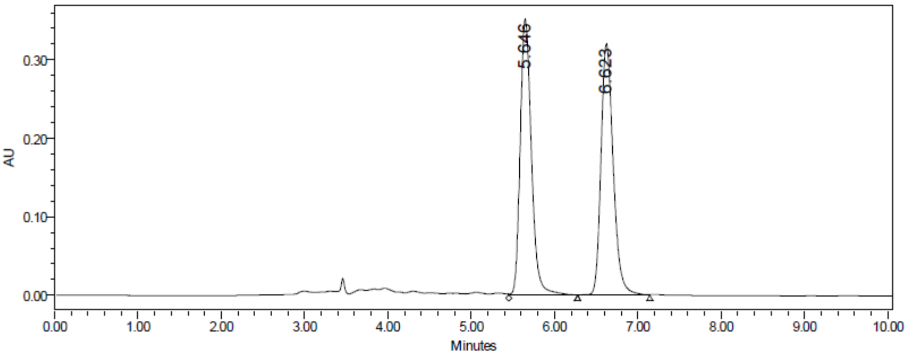

	RT	Area	% Area	Height
1	5.646	3262228	50.18	350856
2	6.623	3238870	49.82	319764

Figure S92. HPLC spectra of **3f,** related to **Scheme 2**.

SAMPLE INFORMATION

Sample Name:	why-g05-103-2-IA-10%	Acquired By:	System
Sample Type:	Unknown	Sample Set Name	
Vial:	105	Acq. Method Set:	10%
Injection #:	1	Processing Method	5 103 2
Injection Volume:	10.00 ul	Channel Name:	2998 Ch1 254nm@1.2nm
Run Time:	100.0 Minutes	Proc. Chnl. Descr.:	2998 Ch1 254nm@1.2nm
Date Acquired:	5/8/2019 8:52:48 PM CST		
Date Processed:	7/19/2019 4:33:25 PM CST		

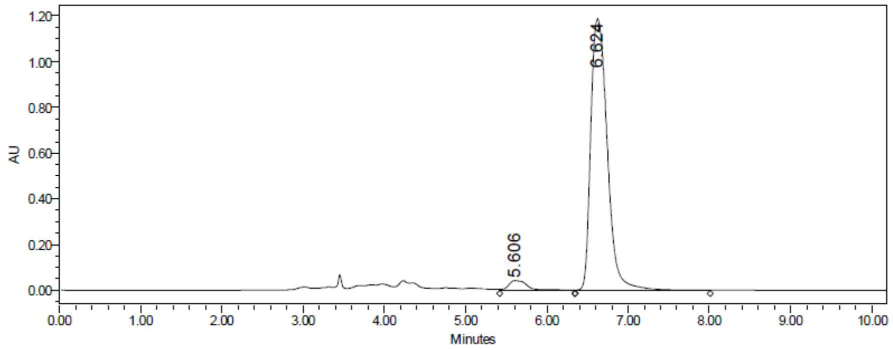

	RT	Area	% Area	Height
1	5.606	719834	4.09	42857
2	6.624	16862017	95.91	1186073

Figure S93. HPLC spectra of *rac*-**3g**, related to **Scheme 2**.

SAMPLE INFORMATION

Sample Name:	why-g05-104-1-IA-3%	Acquired By:	System
Sample Type:	Unknown	Sample Set Name	
Vial:	117	Acq. Method Set:	3%
Injection #:	2	Processing Method	5 104 1
Injection Volume:	10.00 ul	Channel Name:	2998 Ch1 254nm@1.2nm
Run Time:	100.0 Minutes	Proc. Chnl. Descr.:	2998 Ch1 254nm@1.2nm
Date Acquired:	5/8/2019 8:09:13 PM CST		
Date Processed:	7/19/2019 4:34:27 PM CST		

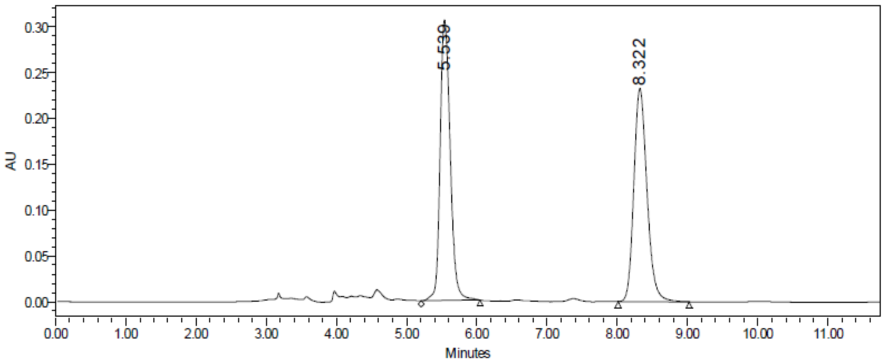

	RT	Area	% Area	Height
1	5.539	3070027	50.27	305813
2	8.322	3037606	49.73	232393

Figure S94. HPLC spectra of **3g,** related to **Scheme 2**.

SAMPLE INFORMATION

Sample Name:	why-g05-104-2-IA-3%	Acquired By:	System
Sample Type:	Unknown	Sample Set Name	
Vial:	3	Acq. Method Set:	3%
Injection #:	1	Processing Method	5 104 2
Injection Volume:	10.00 ul	Channel Name:	2998 Ch1 254nm@1.2nm
Run Time:	100.0 Minutes	Proc. Chnl. Descr.:	2998 Ch1 254nm@1.2nm
Date Acquired:	5/10/2019 2:32:38 PM CST		
Date Processed:	7/19/2019 4:35:27 PM CST		

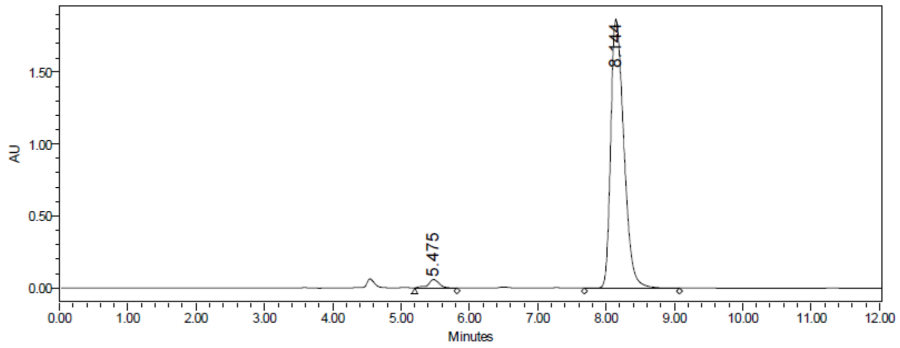

	RT	Area	% Area	Height
1	5.475	619159	2.43	57367
2	8.144	24875543	97.57	1866265

Figure S95. HPLC spectra of *rac*-**3h**, related to **Scheme 2**.

SAMPLE INFORMATION

Sample Name:	why-g05-105-1-IA-5%	Acquired By:	System
Sample Type:	Unknown	Sample Set Name	
Vial:	5	Acq. Method Set:	5%
Injection #:	1	Processing Method	5 105 1
Injection Volume:	10.00 ul	Channel Name:	2998 Ch1 254nm@1.2nm
Run Time:	100.0 Minutes	Proc. Chnl. Descr.:	2998 Ch1 254nm@1.2nm
Date Acquired:	5/24/2019 6:37:39 PM CST		
Date Processed:	7/19/2019 4:38:03 PM CST		

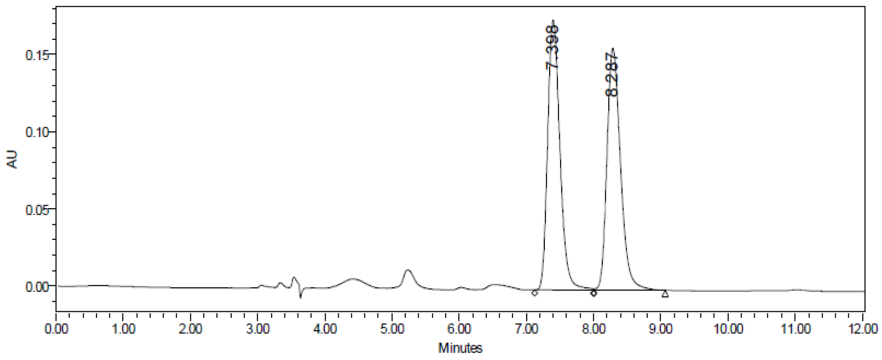

	RT	Area	% Area	Height
1	7.398	2203266	50.09	174497
2	8.287	2195265	49.91	156786

Figure S96. HPLC spectra of **3h**, related to **Scheme 2**.

SAMPLE INFORMATION

Sample Name:	why-g05-105-2-IA-5%	Acquired By:	System
Sample Type:	Unknown	Sample Set Name	
Vial:	46	Acq. Method Set:	5%
Injection #:	2	Processing Method	5 105 2
Injection Volume:	10.00 ul	Channel Name:	2998 Ch1 254nm@1.2nm
Run Time:	12.0 Minutes	Proc. Chnl. Descr.:	2998 Ch1 254nm@1.2nm
Date Acquired:	5/24/2019 7:01:27 PM CST		
Date Processed:	7/19/2019 4:38:38 PM CST		

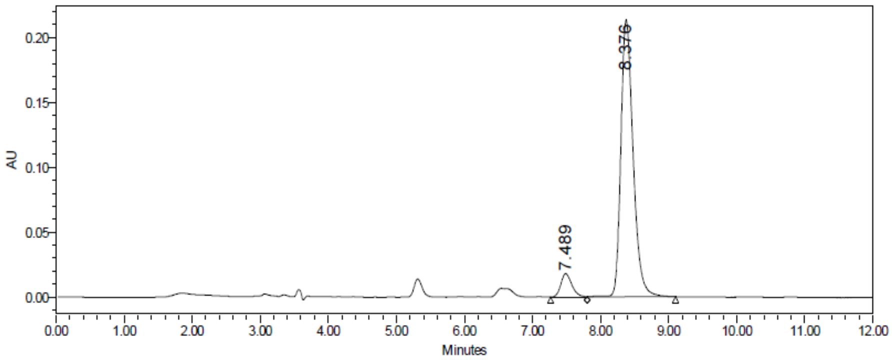

	RT	Area	% Area	Height
1	7.489	201190	6.99	17976
2	8.376	2677362	93.01	213264

Figure S97. HPLC spectra of *rac*-**3i**, related to **Scheme 2**.

SAMPLE INFORMATION

Sample Name:	why-g05-107-1-IA-3%	Acquired By:	System
Sample Type:	Unknown	Sample Set Name	
Vial:	11	Acq. Method Set:	3%
Injection #:	1	Processing Method	5 107 1
Injection Volume:	10.00 ul	Channel Name:	2998 Ch1 254nm@1.2nm
Run Time:	100.0 Minutes	Proc. Chnl. Descr.:	2998 Ch1 254nm@1.2nm
Date Acquired:	5/8/2019 8:22:16 PM CST		
Date Processed:	7/19/2019 4:39:40 PM CST		

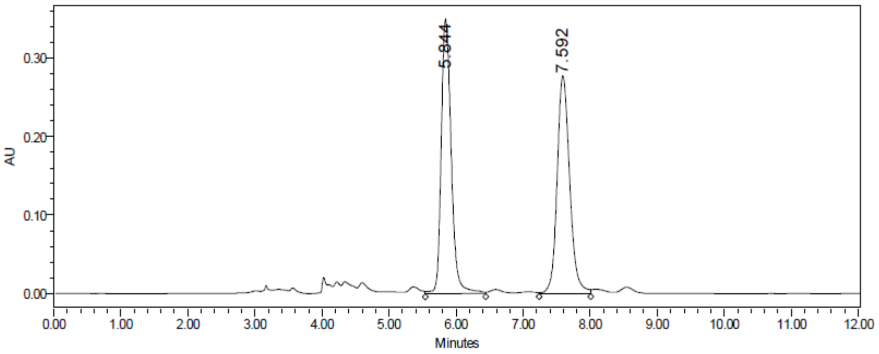

	RT	Area	% Area	Height
1	5.844	3603379	49.93	348868
2	7.592	3613658	50.07	276907

Figure S98. HPLC spectra of **3i**, related to **Scheme 2**.

SAMPLE INFORMATION

Sample Name:	why-g05-107-2-IA-3%	Acquired By:	System
Sample Type:	Unknown	Sample Set Name	
Vial:	87	Acq. Method Set:	3%
Injection #:	1	Processing Method	5 107 2
Injection Volume:	10.00 ul	Channel Name:	2998 Ch1 254nm@1.2nm
Run Time:	100.0 Minutes	Proc. Chnl. Descr.:	2998 Ch1 254nm@1.2nm
Date Acquired:	5/9/2019 6:55:36 PM CST		
Date Processed:	7/19/2019 4:40:21 PM CST		

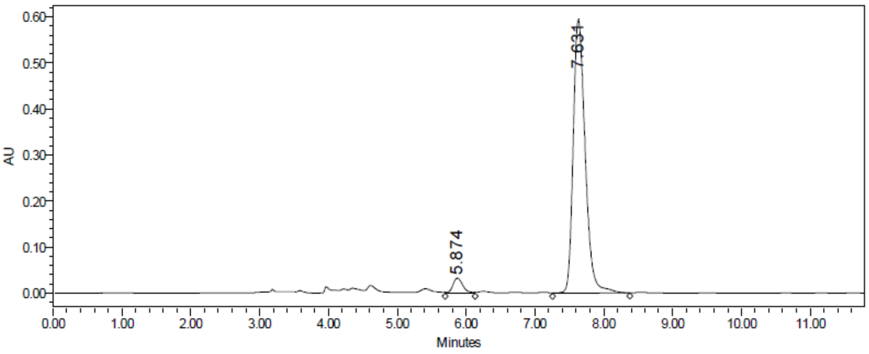

	RT	Area	% Area	Height
1	5.874	332891	4.41	33060
2	7.631	7220776	95.59	593965

Figure S99. HPLC spectra of *rac*-**3j**, related to **Scheme 2**.

SAMPLE INFORMATION

Sample Name:	why-g05-81-2-IA-3%	Acquired By:	System
Sample Type:	Unknown	Sample Set Name	
Vial:	60	Acq. Method Set:	3%
Injection #:	1	Processing Method	5 81 2
Injection Volume:	10.00 ul	Channel Name:	2998 Ch1 254nm@1.2nm
Run Time:	100.0 Minutes	Proc. Chnl. Descr.:	2998 Ch1 254nm@1.2nm
Date Acquired:	4/22/2019 4:50:13 PM CST		
Date Processed:	7/19/2019 4:44:40 PM CST		

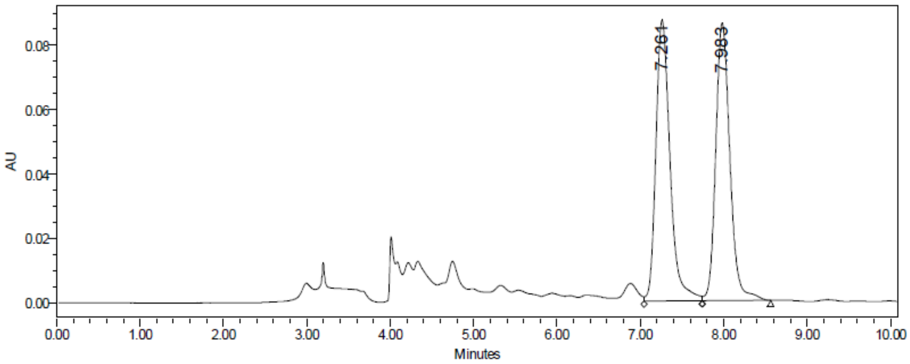

	RT	Area	% Area	Height
1	7.261	1026355	50.26	87261
2	7.983	1015759	49.74	86324

Figure S100. HPLC spectra of **3j**, related to **Scheme 2**.

SAMPLE INFORMATION

Sample Name:	why-g05-81-1-IA-3%	Acquired By:	System
Sample Type:	Unknown	Sample Set Name	
Vial:	3	Acq. Method Set:	3%
Injection #:	1	Processing Method	5 81 1
Injection Volume:	10.00 ul	Channel Name:	2998 Ch1 254nm@1.2nm
Run Time:	100.0 Minutes	Proc. Chnl. Descr.:	2998 Ch1 254nm@1.2nm
Date Acquired:	4/22/2019 5:01:20 PM CST		
Date Processed:	7/19/2019 4:45:09 PM CST		

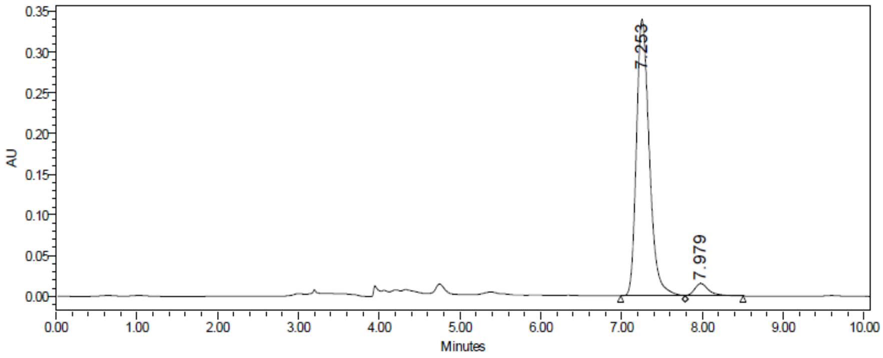

	RT	Area	% Area	Height
1	7.253	3759068	95.17	338669
2	7.979	190617	4.83	15191

Figure S101. HPLC spectra of *rac*-**3k**, related to **Scheme 2**.

SAMPLE INFORMATION

Sample Name:	why-g05-77-1-IA-3%	Acquired By:	System
Sample Type:	Unknown	Sample Set Name	
Vial:	17	Acq. Method Set:	3%
Injection #:	1	Processing Method	5 77 1
Injection Volume:	10.00 ul	Channel Name:	2998 Ch1 254nm@1.2nm
Run Time:	100.0 Minutes	Proc. Chnl. Descr.:	2998 Ch1 254nm@1.2nm
Date Acquired:	4/18/2019 7:20:40 PM CST		
Date Processed:	7/19/2019 4:52:42 PM CST		

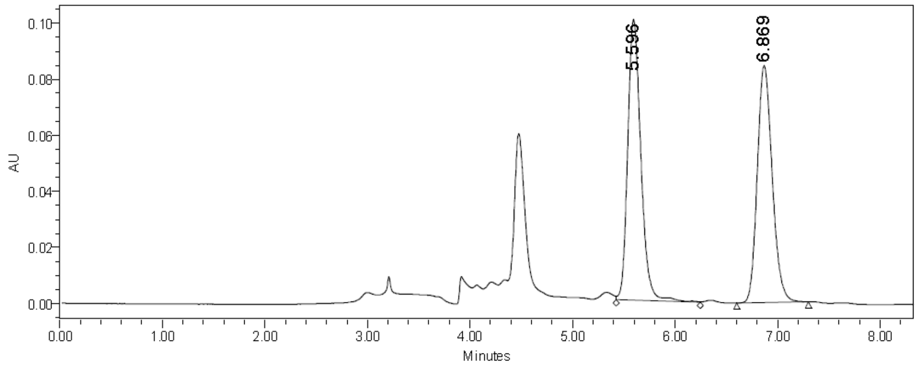

	RT	Area	% Area	Height
1	5.596	897583	51.14	100114
2	6.869	857680	48.86	84508

Figure S102. HPLC spectra of **3k**, related to **Scheme 2**.

SAMPLE INFORMATION

Sample Name:	why-g05-77-2-IA-3%	Acquired By:	System
Sample Type:	Unknown	Sample Set Name	
Vial:	61	Acq. Method Set:	3%
Injection #:	1	Processing Method	5 77 2
Injection Volume:	10.00 ul	Channel Name:	2998 Ch1 254nm@1.2nm
Run Time:	100.0 Minutes	Proc. Chnl. Descr.:	2998 Ch1 254nm@1.2nm
Date Acquired:	7/23/2019 4:09:48 PM CST		
Date Processed:	8/5/2019 4:15:42 PM CST		

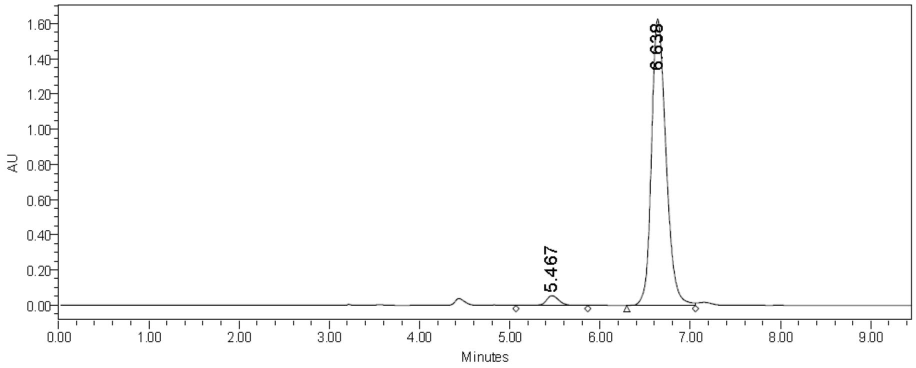

	RT	Area	% Area	Height
1	5.467	548142	2.91	54996
2	6.638	18266140	97.09	1626763

Figure S103. HPLC spectra of *rac*-**3l**, related to **Scheme 2**.

SAMPLE INFORMATION

Sample Name:	why-g05-94-1-IA-3%	Acquired By:	System
Sample Type:	Unknown	Sample Set Name	
Vial:	68	Acq. Method Set:	3%
Injection #:	1	Processing Method	5 94 1
Injection Volume:	10.00 ul	Channel Name:	2998 Ch1 254nm@1.2nm
Run Time:	100.0 Minutes	Proc. Chnl. Descr.:	2998 Ch1 254nm@1.2nm
Date Acquired:	4/25/2019 7:45:58 PM CST		
Date Processed:	7/19/2019 4:56:54 PM CST		

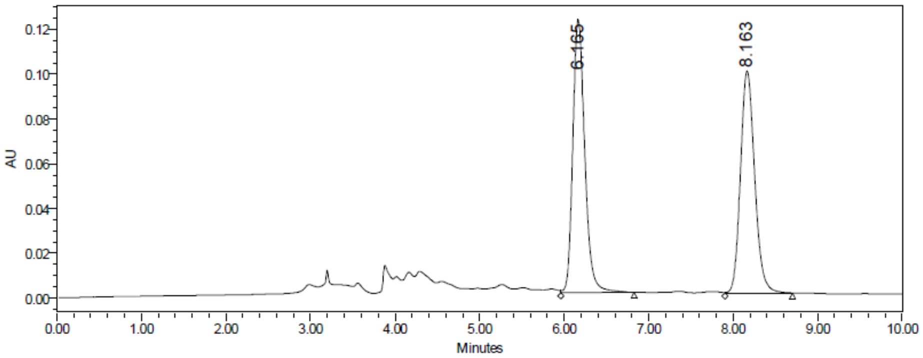

	RT	Area	% Area	Height
1	6.165	1193006	50.69	121836
2	8.163	1160651	49.31	99218

Figure S104. HPLC spectra of **3l**, related to **Scheme 2**.

SAMPLE INFORMATION

Sample Name:	why-g05-94-2-IA-3%	Acquired By:	System
Sample Type:	Unknown	Sample Set Name	
Vial:	20	Acq. Method Set:	3%
Injection #:	1	Processing Method	5 94 2
Injection Volume:	10.00 ul	Channel Name:	2998 Ch1 254nm@1.2nm
Run Time:	10.0 Minutes	Proc. Chnl. Descr.:	2998 Ch1 254nm@1.2nm
Date Acquired:	4/26/2019 2:41:42 PM CST		
Date Processed:	7/19/2019 4:57:19 PM CST		

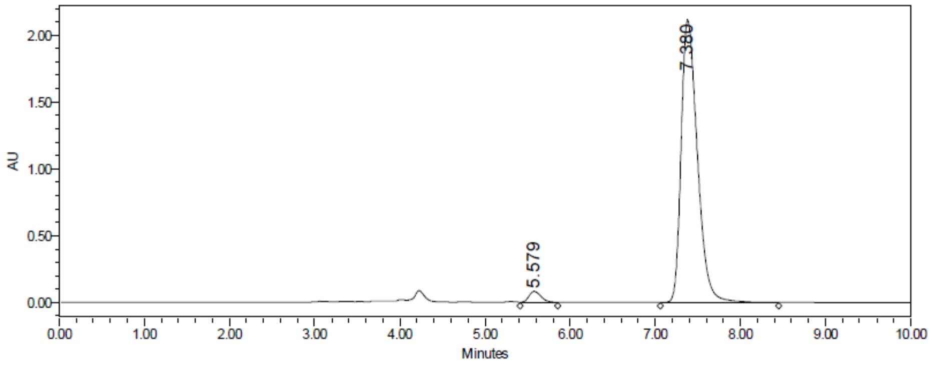

	RT	Area	% Area	Height
1	5.579	847402	2.94	83595
2	7.380	27946061	97.06	2114242

Figure S105. HPLC spectra of *rac*-**3m**, related to **Scheme 2**.

SAMPLE INFORMATION

Sample Name:	why-g05-93-1-IA-3%	Acquired By:	System
Sample Type:	Unknown	Sample Set Name	
Vial:	93	Acq. Method Set:	3%
Injection #:	1	Processing Method	5 93 1
Injection Volume:	10.00 ul	Channel Name:	2998 Ch1 254nm@1.2nm
Run Time:	100.0 Minutes	Proc. Chnl. Descr.:	2998 Ch1 254nm@1.2nm
Date Acquired:	4/24/2019 7:21:42 PM CST		
Date Processed:	7/19/2019 4:55:33 PM CST		

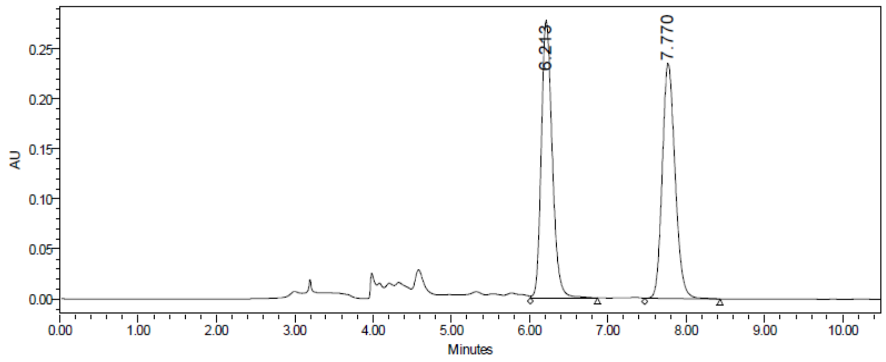

	RT	Area	% Area	Height
1	6.213	2689605	50.26	276948
2	7.770	2661667	49.74	234692

Figure S106. HPLC spectra of **3m**, related to **Scheme 2**.

SAMPLE INFORMATION

Sample Name:	why-g05-93-2-IA-3%	Acquired By:	System
Sample Type:	Unknown	Sample Set Name	
Vial:	39	Acq. Method Set:	3%
Injection #:	1	Processing Method	5 93 2
Injection Volume:	10.00 ul	Channel Name:	2998 Ch1 254nm@1.2nm
Run Time:	100.0 Minutes	Proc. Chnl. Descr.:	2998 Ch1 254nm@1.2nm
Date Acquired:	4/25/2019 7:28:12 PM CST		
Date Processed:	7/19/2019 4:56:19 PM CST		

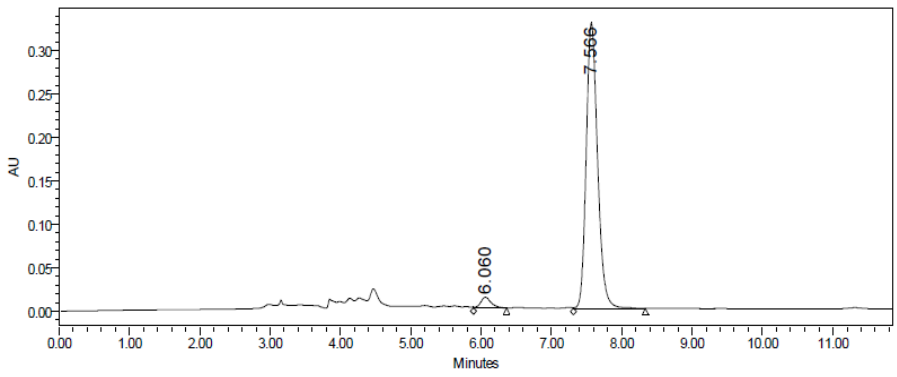

	RT	Area	% Area	Height
1	6.060	122657	3.21	12140
2	7.566	3695531	96.79	328030

Figure S107. HPLC spectra of *rac*-**3n**, related to **Scheme 2**.

SAMPLE INFORMATION

Sample Name:	why-g05-92-1-IA-3%	Acquired By:	System
Sample Type:	Unknown	Sample Set Name	
Vial:	62	Acq. Method Set:	3%
Injection #:	1	Processing Method	5 92 1
Injection Volume:	10.00 ul	Channel Name:	2998 Ch1 254nm@1.2nm
Run Time:	100.0 Minutes	Proc. Chnl. Descr.:	2998 Ch1 254nm@1.2nm
Date Acquired:	4/24/2019 7:33:32 PM CST		
Date Processed:	7/19/2019 4:54:10 PM CST		

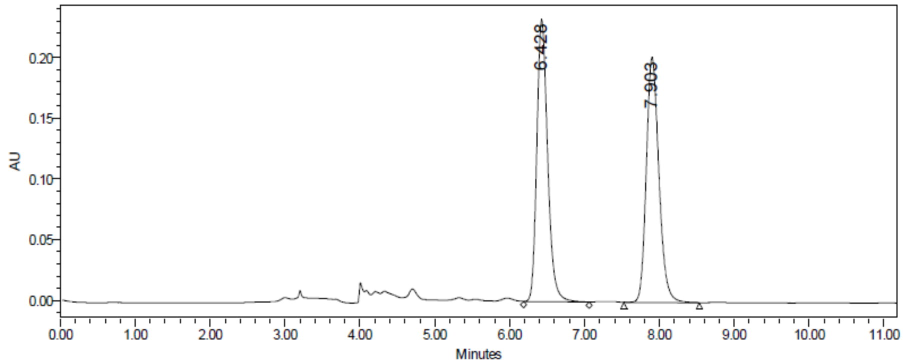

	RT	Area	% Area	Height
1	6.428	2345053	49.77	232209
2	7.903	2367186	50.23	202032

Figure S108. HPLC spectra of **3n**, related to **Scheme 2**.

SAMPLE INFORMATION

Sample Name:	why-g05-92-2-IA-3%	Acquired By:	System
Sample Type:	Unknown	Sample Set Name	
Vial:	7	Acq. Method Set:	3%
Injection #:	1	Processing Method	5 92 2 2
Injection Volume:	10.00 ul	Channel Name:	2998 Ch1 254nm@1.2nm
Run Time:	100.0 Minutes	Proc. Chnl. Descr.:	2998 Ch1 254nm@1.2nm
Date Acquired:	4/25/2019 2:53:22 PM CST		
Date Processed:	7/19/2019 4:54:40 PM CST		

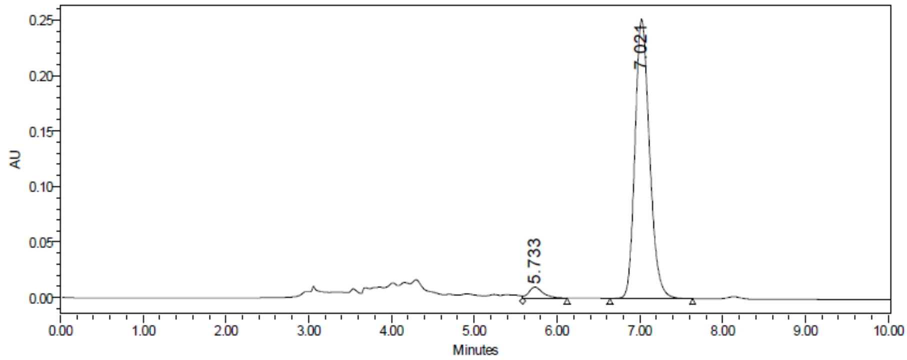

	RT	Area	% Area	Height
1	5.733	112010	3.49	9656
2	7.021	3096909	96.51	251145

Figure S109. HPLC spectra of *rac*-**3o**, related to **Scheme 2**.

SAMPLE INFORMATION

Sample Name:	why-g05-108-1-IA-3%	Acquired By:	System
Sample Type:	Unknown	Sample Set Name	
Vial:	29	Acq. Method Set:	3%
Injection #:	1	Processing Method	5 108 1
Injection Volume:	10.00 ul	Channel Name:	2998 Ch1 254nm@1.2nm
Run Time:	100.0 Minutes	Proc. Chnl. Descr.:	2998 Ch1 254nm@1.2nm
Date Acquired:	5/8/2019 8:35:18 PM CST		
Date Processed:	7/19/2019 5:00:18 PM CST		

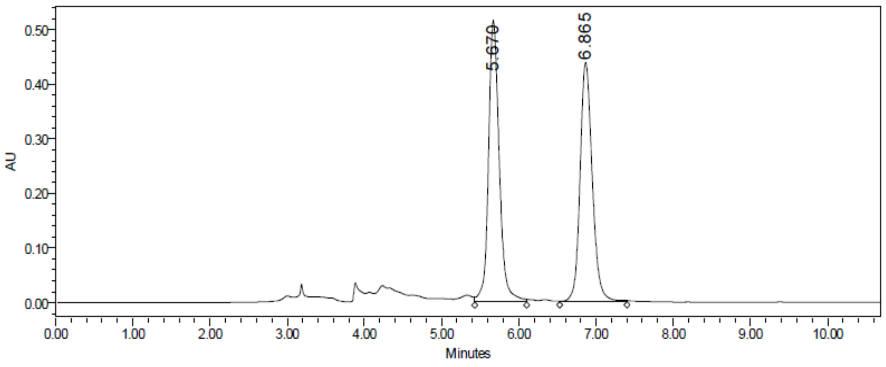

	RT	Area	% Area	Height
1	5.670	5001622	50.69	514650
2	6.865	4864935	49.31	438508

Figure S110. HPLC spectra of **3o**, related to **Scheme 2**.

SAMPLE INFORMATION

Sample Name:	why-g05-108-2-IA-3%	Acquired By:	System
Sample Type:	Unknown	Sample Set Name	
Vial:	54	Acq. Method Set:	3%
Injection #:	1	Processing Method	5 108 2
Injection Volume:	10.00 ul	Channel Name:	2998 Ch1 254nm@1.2nm
Run Time:	100.0 Minutes	Proc. Chnl. Descr.:	2998 Ch1 254nm@1.2nm
Date Acquired:	5/9/2019 6:44:31 PM CST		
Date Processed:	7/19/2019 5:00:44 PM CST		

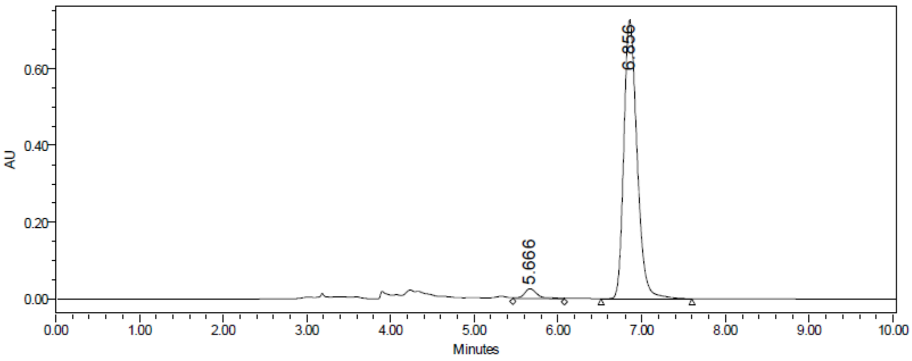

	RT	Area	% Area	Height
1	5.666	262467	3.21	25190
2	6.856	7924180	96.79	724173

Figure S111. HPLC spectra of *rac*-**3p**, related to **Scheme 2**.

SAMPLE INFORMATION

Sample Name:	why-g05-78-1-IA-3%	Acquired By:	System
Sample Type:	Unknown	Sample Set Name	
Vial:	18	Acq. Method Set:	3%
Injection #:	1	Processing Method	5 78 1
Injection Volume:	10.00 ul	Channel Name:	2998 Ch1 254nm@1.2nm
Run Time:	100.0 Minutes	Proc. Chnl. Descr.:	2998 Ch1 254nm@1.2nm
Date Acquired:	4/18/2019 7:29:57 PM CST		
Date Processed:	7/19/2019 4:58:20 PM CST		

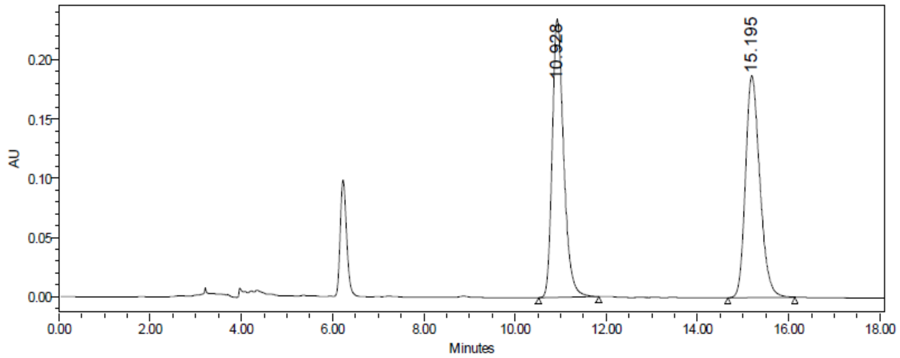

	RT	Area	% Area	Height
1	10.928	4064719	49.84	234399
2	15.195	4090337	50.16	187143

Figure S112. HPLC spectra of **3p**, related to **Scheme 2**.

SAMPLE INFORMATION

Sample Name:	why-g05-78-2-IA-3%	Acquired By:	System
Sample Type:	Unknown	Sample Set Name	
Vial:	28	Acq. Method Set:	3%
Injection #:	1	Processing Method	5 78 2
Injection Volume:	10.00 ul	Channel Name:	2998 Ch1 254nm@1.2nm
Run Time:	100.0 Minutes	Proc. Chnl. Descr.:	2998 Ch1 254nm@1.2nm
Date Acquired:	4/18/2019 7:59:30 PM CST		
Date Processed:	7/19/2019 4:58:55 PM CST		

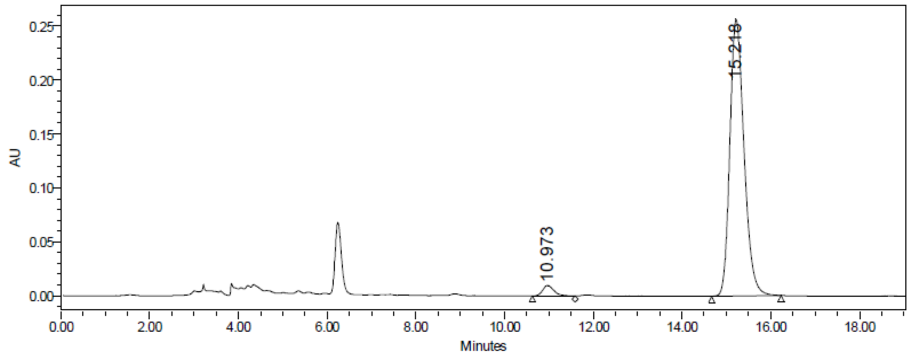

	RT	Area	% Area	Height
1	10.973	171013	2.94	9718
2	15.218	5652888	97.06	256407

Figure S113. HPLC spectra of *rac*-**3q**, related to **Scheme 2**.

SAMPLE INFORMATION

Sample Name:	why-g05-95-1-IA-10%	Acquired By:	System
Sample Type:	Unknown	Sample Set Name	
Vial:	95	Acq. Method Set:	10%
Injection #:	1	Processing Method	5 95 1
Injection Volume:	10.00 ul	Channel Name:	2998 Ch1 254nm@1.2nm
Run Time:	100.0 Minutes	Proc. Chnl. Descr.:	2998 Ch1 254nm@1.2nm
Date Acquired:	4/25/2019 8:49:00 PM CST		
Date Processed:	7/19/2019 5:02:52 PM CST		

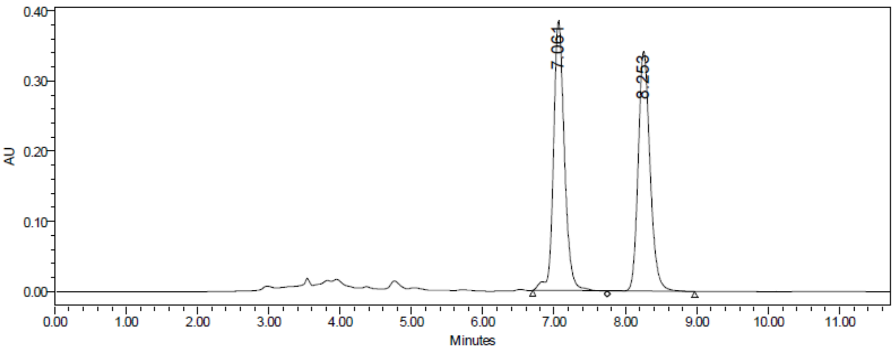

	RT	Area	% Area	Height
1	7.061	4062898	50.61	383542
2	8.253	3964240	49.39	341550

Figure S114. HPLC spectra of **3q**, related to **Scheme 2**.

SAMPLE INFORMATION

Sample Name:	why-g05-95-2-IA-10%	Acquired By:	System
Sample Type:	Unknown	Sample Set Name	
Vial:	25	Acq. Method Set:	10%
Injection #:	1	Processing Method	5 95 2
Injection Volume:	10.00 ul	Channel Name:	2998 Ch1 254nm@1.2nm
Run Time:	100.0 Minutes	Proc. Chnl. Descr.:	2998 Ch1 254nm@1.2nm
Date Acquired:	4/26/2019 7:23:15 PM CST		
Date Processed:	7/19/2019 5:03:23 PM CST		

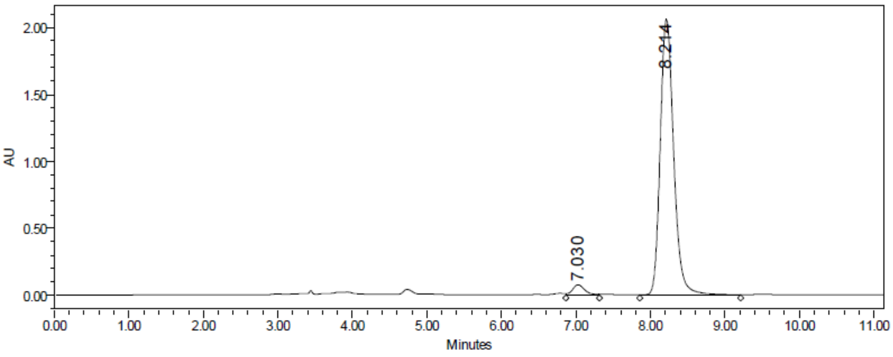

	RT	Area	% Area	Height
1	7.030	827616	3.15	74981
2	8.214	25472113	96.85	2056802

Figure S115. HPLC spectra of *rac*-**3r**, related to **Scheme 2**.

SAMPLE INFORMATION

Sample Name:	why-g05-97-1-IA-1.5%	Acquired By:	System
Sample Type:	Unknown	Sample Set Name	
Vial:	5	Acq. Method Set:	1 5%
Injection #:	1	Processing Method	5 97 1
Injection Volume:	10.00 ul	Channel Name:	2998 Ch1 254nm@1.2nm
Run Time:	100.0 Minutes	Proc. Chnl. Descr.:	2998 Ch1 254nm@1.2nm
Date Acquired:	4/27/2019 8:11:01 PM CST		
Date Processed:	7/19/2019 5:03:57 PM CST		

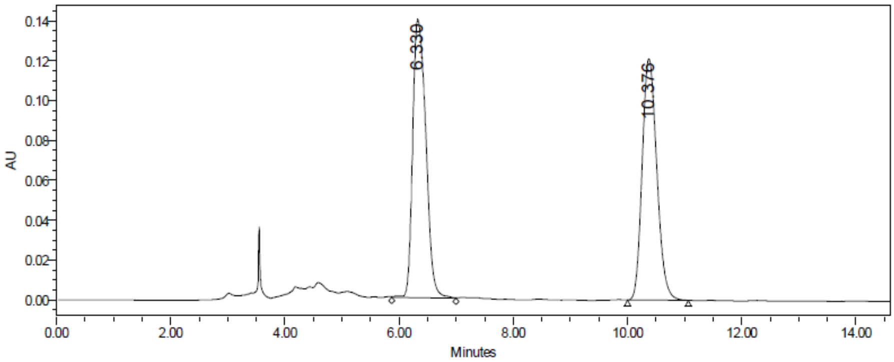

	RT	Area	% Area	Height
1	6.330	2216005	50.51	139512
2	10.376	2171620	49.49	121179

Figure S116. HPLC spectra of **3r**, related to **Scheme 2**.

SAMPLE INFORMATION

Sample Name:	why-g05-97-2-IA-1.5%	Acquired By:	System
Sample Type:	Unknown	Sample Set Name	
Vial:	4	Acq. Method Set:	1 5%
Injection #:	1	Processing Method	5 97 2
Injection Volume:	10.00 ul	Channel Name:	2998 Ch1 254nm@1.2nm
Run Time:	14.0 Minutes	Proc. Chnl. Descr.:	2998 Ch1 254nm@1.2nm
Date Acquired:	4/27/2019 8:26:29 PM CST		
Date Processed:	7/19/2019 5:04:21 PM CST		

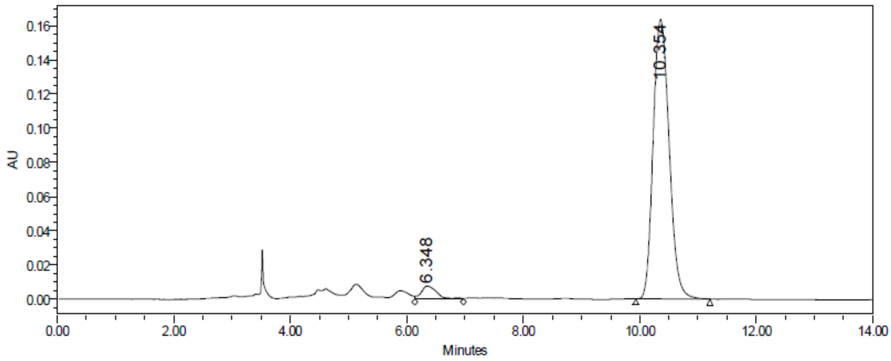

	RT	Area	% Area	Height
1	6.348	132661	4.09	7406
2	10.354	3111023	95.91	163191

Figure S117. HPLC spectra of *rac*-**3s**, related to **Scheme 2**.

SAMPLE INFORMATION

Sample Name:	why-g05-115-1-IA-3%	Acquired By:	System
Sample Type:	Unknown	Sample Set Name	
Vial:	51	Acq. Method Set:	3%
Injection #:	1	Processing Method	5 115 1
Injection Volume:	10.00 ul	Channel Name:	2998 Ch1 254nm@1.2nm
Run Time:	100.0 Minutes	Proc. Chnl. Descr.:	2998 Ch1 254nm@1.2nm
Date Acquired:	5/20/2019 8:55:09 PM CST		
Date Processed:	7/19/2019 5:05:32 PM CST		

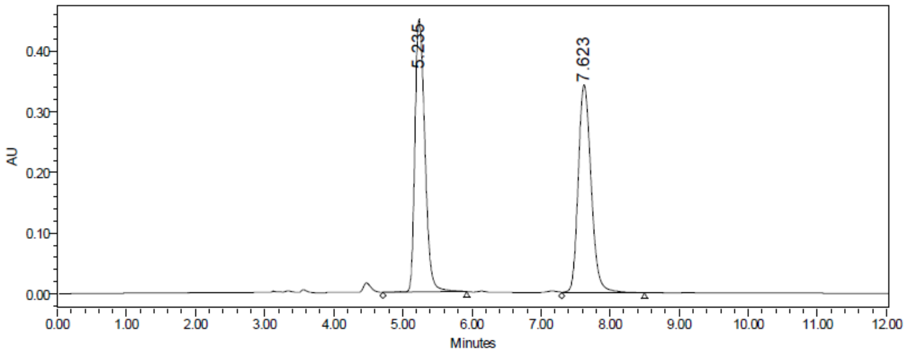

	RT	Area	% Area	Height
1	5.235	4473670	50.07	448631
2	7.623	4461737	49.93	342190

Figure S118. HPLC spectra of **3s**, related to **Scheme 2**.

SAMPLE INFORMATION

Sample Name:	why-g05-115-2-IA-3%	Acquired By:	System
Sample Type:	Unknown	Sample Set Name	
Vial:	110	Acq. Method Set:	3%
Injection #:	1	Processing Method	5 115 2
Injection Volume:	10.00 ul	Channel Name:	2998 Ch1 254nm@1.2nm
Run Time:	100.0 Minutes	Proc. Chnl. Descr.:	2998 Ch1 254nm@1.2nm
Date Acquired:	5/21/2019 8:08:57 PM CST		
Date Processed:	7/19/2019 5:06:16 PM CST		

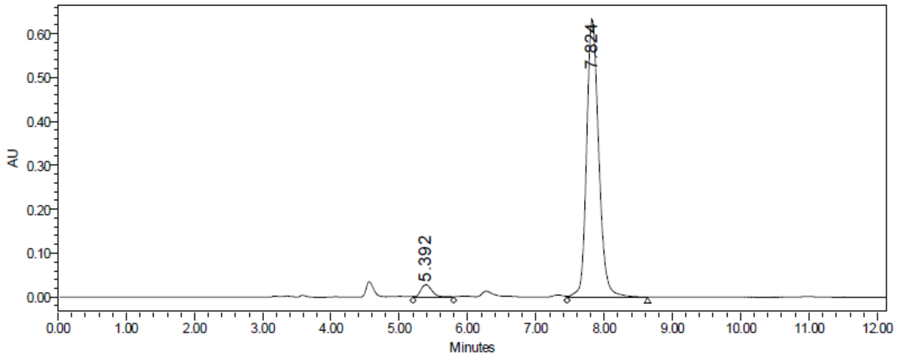

	RT	Area	% Area	Height
1	5.392	322979	3.95	28062
2	7.824	7850224	96.05	631496

Figure S119. HPLC spectra of *rac*-**3t**, related to **Scheme 2**.

SAMPLE INFORMATION

Sample Name:	why-g05-116-1-IA-3%	Acquired By:	System
Sample Type:	Unknown	Sample Set Name	
Vial:	75	Acq. Method Set:	3%
Injection #:	1	Processing Method	5 116 2
Injection Volume:	10.00 ul	Channel Name:	2998 Ch1 254nm@1.2nm
Run Time:	100.0 Minutes	Proc. Chnl. Descr.:	2998 Ch1 254nm@1.2nm
Date Acquired:	5/20/2019 9:53:04 PM CST		
Date Processed:	7/19/2019 5:06:54 PM CST		

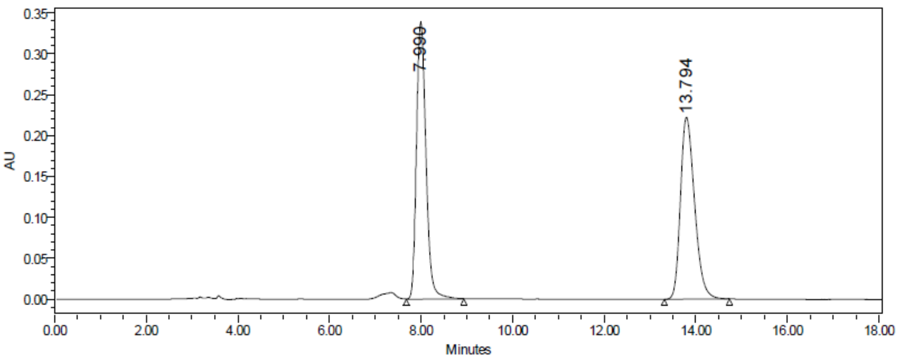

	RT	Area	% Area	Height
1	7.990	4847139	50.24	338076
2	13.794	4801700	49.76	222331

Figure S120. HPLC spectra of **3t**, related to **Scheme 2**.

SAMPLE INFORMATION

Sample Name:	why-g05-116-2-IA-3%	Acquired By:	System
Sample Type:	Unknown	Sample Set Name	
Vial:	21	Acq. Method Set:	3%
Injection #:	1	Processing Method	5 116 2
Injection Volume:	10.00 ul	Channel Name:	2998 Ch1 254nm@1.2nm
Run Time:	100.0 Minutes	Proc. Chnl. Descr.:	2998 Ch1 254nm@1.2nm
Date Acquired:	5/21/2019 8:52:38 PM CST		
Date Processed:	7/19/2019 5:08:01 PM CST		

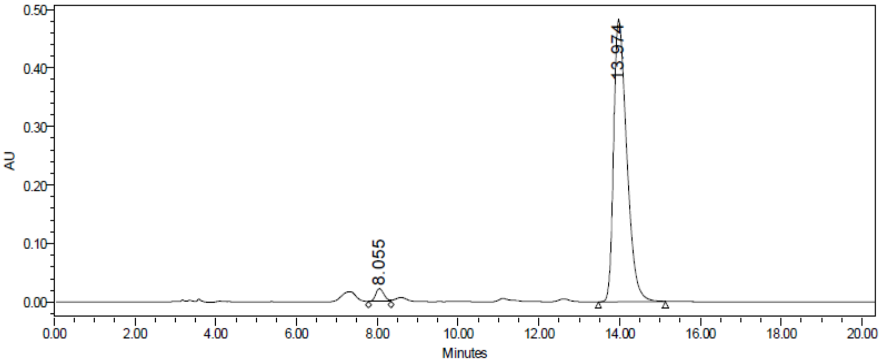

	RT	Area	% Area	Height
1	8.055	318489	2.85	21925
2	13.974	10860475	97.15	481624

Figure S121. HPLC spectra of *rac*-**3u**, related to **Scheme 2**.

SAMPLE INFORMATION

Sample Name:	why-g05-98-1-IA-3%	Acquired By:	System
Sample Type:	Unknown	Sample Set Name	
Vial:	46	Acq. Method Set:	3%
Injection #:	1	Processing Method	5 98 1
Injection Volume:	10.00 ul	Channel Name:	2998 Ch1 254nm@1.2nm
Run Time:	100.0 Minutes	Proc. Chnl. Descr.:	2998 Ch1 254nm@1.2nm
Date Acquired:	4/27/2019 9:57:01 AM CST		
Date Processed:	7/19/2019 4:47:50 PM CST		

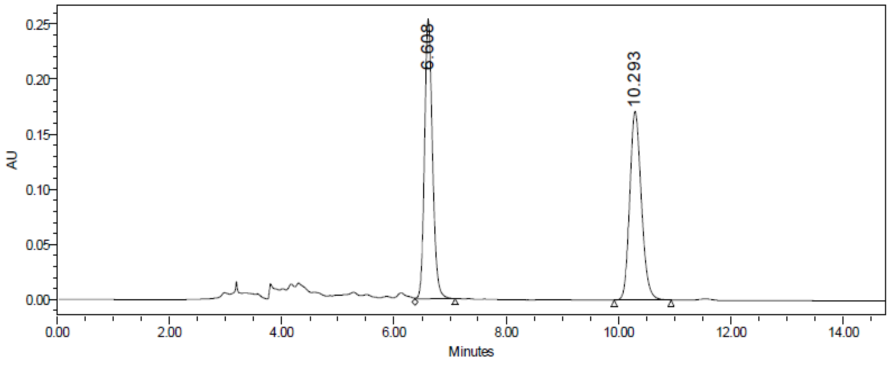

	RT	Area	% Area	Height
1	6.608	2497231	50.16	253252
2	10.293	2481349	49.84	171382

Figure S122. HPLC spectra of **3u**, related to **Scheme 2**.

SAMPLE INFORMATION

Sample Name:	why-g05-98-2-IA-3%	Acquired By:	System
Sample Type:	Unknown	Sample Set Name	
Vial:	14	Acq. Method Set:	3%
Injection #:	1	Processing Method	5 98 2
Injection Volume:	10.00 ul	Channel Name:	2998 Ch1 254nm@1.2nm
Run Time:	100.0 Minutes	Proc. Chnl. Descr.:	2998 Ch1 254nm@1.2nm
Date Acquired:	4/28/2019 4:33:51 PM CST		
Date Processed:	7/19/2019 4:48:45 PM CST		

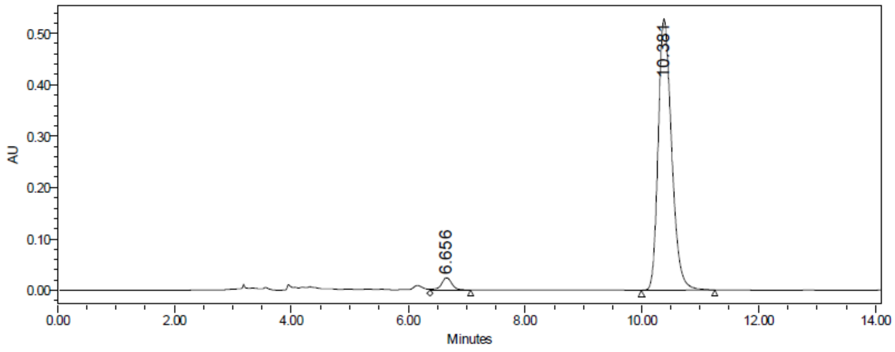

	RT	Area	% Area	Height
1	6.656	294513	3.41	24072
2	10.381	8344780	96.59	526504

Figure S123. HPLC spectra of *rac*-**3v**, related to **Scheme 2**.

SAMPLE INFORMATION

Sample Name:	why-g05-168-2-2-IA-3%	Acquired By:	System
Sample Type:	Unknown	Sample Set Name	
Vial:	33	Acq. Method Set:	3%
Injection #:	1	Processing Method	5 168 2 2
Injection Volume:	10.00 ul	Channel Name:	2998 Ch1 254nm@1.2nm
Run Time:	100.0 Minutes	Proc. Chnl. Descr.:	2998 Ch1 254nm@1.2nm
Date Acquired:	6/24/2019 7:44:30 PM CST		
Date Processed:	8/3/2019 5:52:45 PM CST		

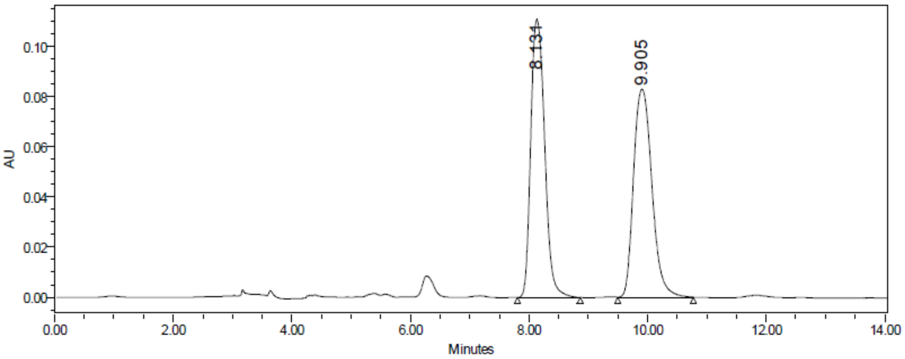

	RT	Area	% Area	Height
1	8.131	1819609	50.38	110730
2	9.905	1791927	49.62	82895

Figure S124. HPLC spectra of **3v**, related to **Scheme 2**.

SAMPLE INFORMATION

Sample Name:	why-g05-168-1-2-IA-3%	Acquired By:	System
Sample Type:	Unknown	Sample Set Name	
Vial:	63	Acq. Method Set:	3%
Injection #:	1	Processing Method	5 168 1 2
Injection Volume:	10.00 ul	Channel Name:	2998 Ch1 254nm@1.2nm
Run Time:	100.0 Minutes	Proc. Chnl. Descr.:	2998 Ch1 254nm@1.2nm
Date Acquired:	6/24/2019 7:59:47 PM CST		
Date Processed:	8/3/2019 5:52:01 PM CST		

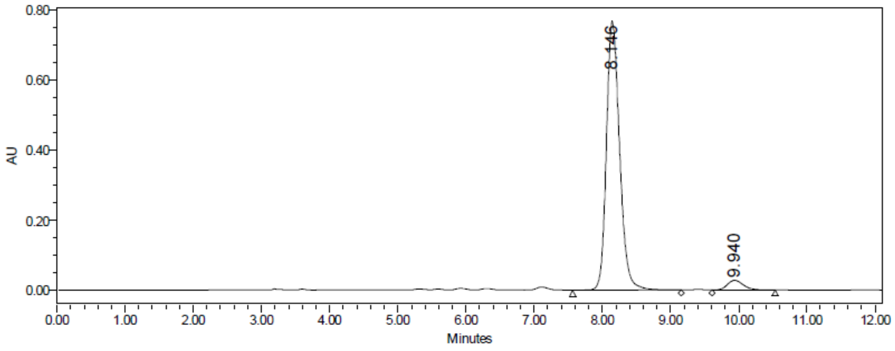

	RT	Area	% Area	Height
1	8.146	10277030	95.77	766342
2	9.940	453768	4.23	27838

Figure S125. HPLC spectra of *rac*-**3w**, related to **Scheme 2**.

SAMPLE INFORMATION

Sample Name:	why-g06-68-2-IA-1%	Acquired By:	System
Sample Type:	Unknown	Sample Set Name	
Vial:	115	Acq. Method Set:	1%
Injection #:	1	Processing Method	6 68 2
Injection Volume:	10.00 ul	Channel Name:	2998 Ch1 254nm@1.2nm
Run Time:	100.0 Minutes	Proc. Chnl. Descr.:	2998 Ch1 254nm@1.2nm
Date Acquired:	10/28/2019 9:46:58 PM CST		
Date Processed:	10/29/2019 7:56:09 PM CST		

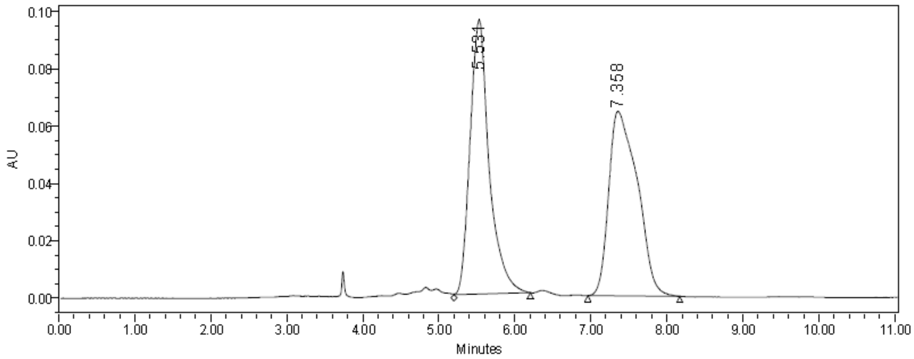

	RT	Area	% Area	Height
1	5.531	1662937	49.74	95598
2	7.358	1680242	50.26	64357

Figure S126. HPLC spectra of **3w**, related to **Scheme 2**.

SAMPLE INFORMATION

Sample Name:	why-g06-66-2-IA-1%	Acquired By:	System
Sample Type:	Unknown	Sample Set Name	
Vial:	96	Acq. Method Set:	1%
Injection #:	1	Processing Method	6 66 2
Injection Volume:	10.00 ul	Channel Name:	2998 Ch1 254nm@1.2nm
Run Time:	100.0 Minutes	Proc. Chnl. Descr.:	2998 Ch1 254nm@1.2nm
Date Acquired:	10/29/2019 12:01:45 PM CST		
Date Processed:	10/29/2019 12:19:04 PM CST		

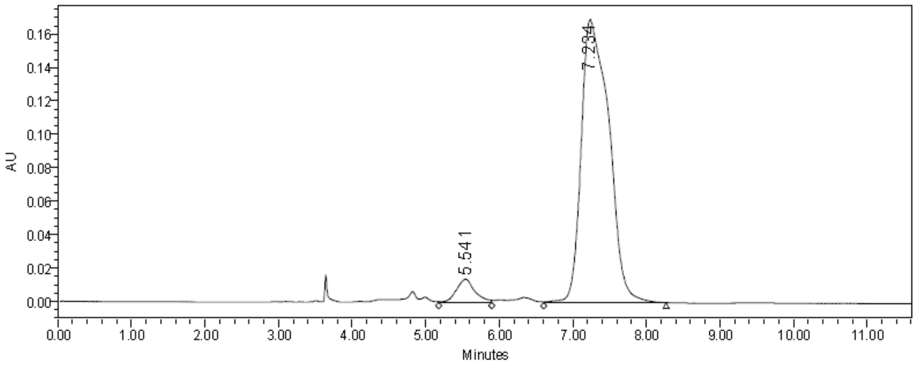

	RT	Area	% Area	Height
1	5.541	243961	5.21	13942
2	7.234	4438154	94.79	169210

Figure S127. HPLC spectra of *rac*-**3x**, related to **Scheme 2**.

SAMPLE INFORMATION

Sample Name:	why-g06-69-2-AS-H-3%	Acquired By:	System
Sample Type:	Unknown	Sample Set Name	
Vial:	30	Acq. Method Set:	3%
Injection #:	1	Processing Method	6 69 2
Injection Volume:	10.00 ul	Channel Name:	2998 Ch1 254nm@1.2nm
Run Time:	100.0 Minutes	Proc. Chnl. Descr.:	2998 Ch1 254nm@1.2nm
Date Acquired:	10/28/2019 5:30:58 PM CST		
Date Processed:	10/29/2019 8:31:18 PM CST		

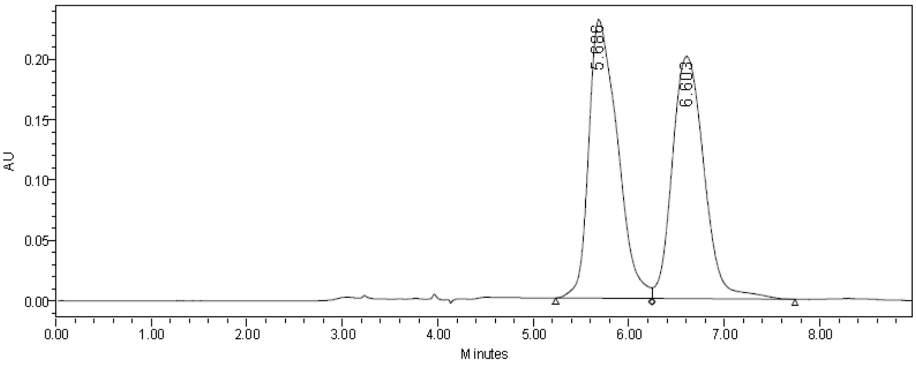

	RT	Area	% Area	Height
1	5.686	4814427	50.89	230369
2	6.603	4646848	49.11	200356

Figure S128. HPLC spectra of **3x**, related to **Scheme 2**.

SAMPLE INFORMATION

Sample Name:	why-g06-67-2-AS-H-3%	Acquired By:	System
Sample Type:	Unknown	Sample Set Name	
Vial:	117	Acq. Method Set:	3%
Injection #:	1	Processing Method	6 67 2
Injection Volume:	10.00 ul	Channel Name:	2998 Ch1 254nm@1.2nm
Run Time:	100.0 Minutes	Proc. Chnl. Descr.:	2998 Ch1 254nm@1.2nm
Date Acquired:	10/29/2019 12:21:53 PM CST		
Date Processed:	10/29/2019 8:29:38 PM CST		

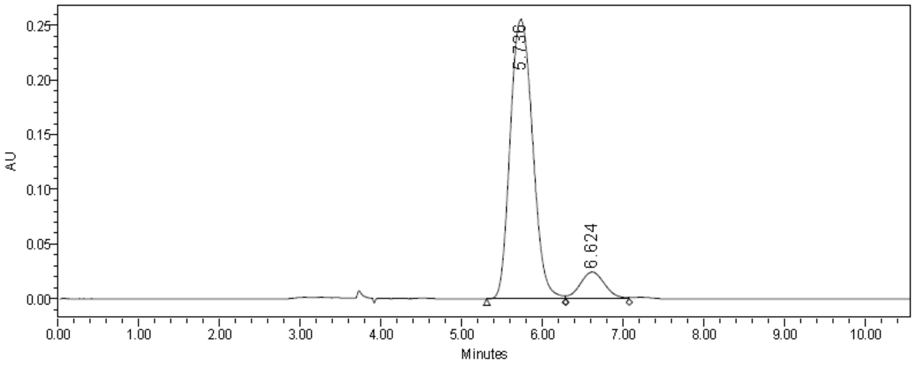

	RT	Area	% Area	Height
1	5.736	4932155	90.43	255229
2	6.624	522203	9.57	24555

Figure S129. HPLC spectra of *rac*-**3y**, related to **Scheme 2**.

SAMPLE INFORMATION

Sample Name:	why-g05-99-2-rac-IA-3%	Acquired By:	System
Sample Type:	Unknown	Sample Set Name	
Vial:	27	Acq. Method Set:	3%
Injection #:	1	Processing Method	5 99 2rac
Injection Volume:	10.00 ul	Channel Name:	2998 Ch1 254nm@1.2nm
Run Time:	100.0 Minutes	Proc. Chnl. Descr.:	2998 Ch1 254nm@1.2nm
Date Acquired:	4/29/2019 4:33:41 PM CST		
Date Processed:	8/3/2019 6:54:41 PM CST		

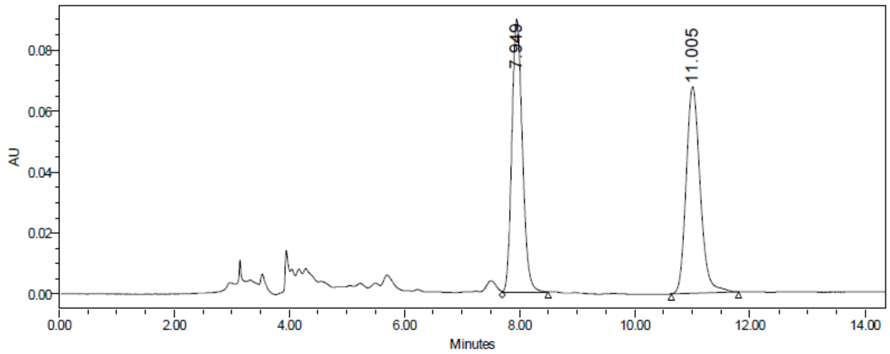

	RT	Area	% Area	Height
1	7.949	1119569	49.23	89261
2	11.005	1154401	50.77	67748

Figure S130. HPLC spectra of **3y**, related to **Scheme 2**.

SAMPLE INFORMATION

Sample Name:	why-g05-99-2-IA-3%	Acquired By:	System
Sample Type:	Unknown	Sample Set Name	
Vial:	35	Acq. Method Set:	3%
Injection #:	1	Processing Method	5 99 2
Injection Volume:	10.00 ul	Channel Name:	2998 Ch1 254nm@1.2nm
Run Time:	100.0 Minutes	Proc. Chnl. Descr.:	2998 Ch1 254nm@1.2nm
Date Acquired:	4/28/2019 5:00:33 PM CST		
Date Processed:	8/3/2019 6:55:47 PM CST		

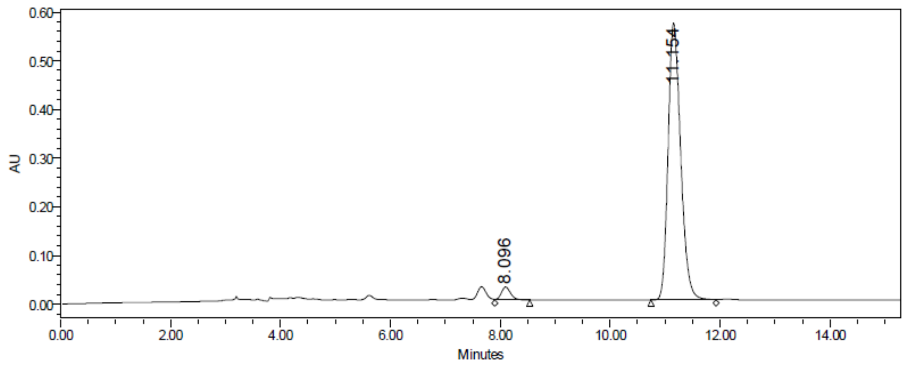

	RT	Area	% Area	Height
1	8.096	313855	3.39	26703
2	11.154	8945885	96.61	567758

Figure S131. HPLC spectra of *rac*-**3z**, related to **Scheme 2**.

SAMPLE INFORMATION

Sample Name:	why-g05-99-1-rac-IA-1.5%	Acquired By:	System
Sample Type:	Unknown	Sample Set Name	
Vial:	44	Acq. Method Set:	1 5%
Injection #:	1	Processing Method	5 99 1 rac
Injection Volume:	10.00 ul	Channel Name:	2998 Ch1 254nm@1.2nm
Run Time:	100.0 Minutes	Proc. Chnl. Descr.:	2998 Ch1 254nm@1.2nm
Date Acquired:	5/5/2019 5:25:59 PM CST		
Date Processed:	7/19/2019 4:50:47 PM CST		

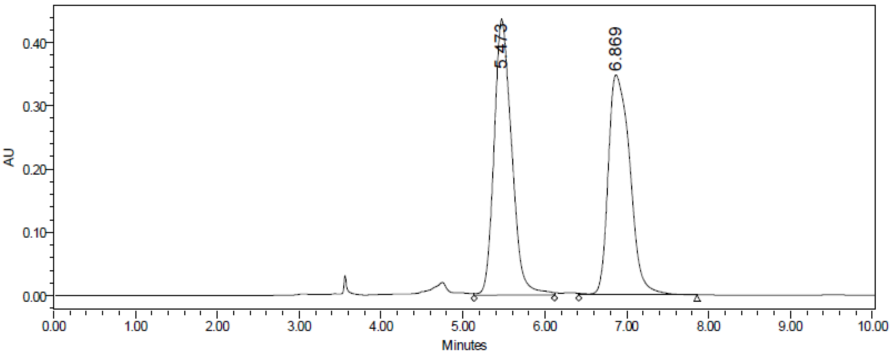

	RT	Area	% Area	Height
1	5.473	6503943	50.32	435332
2	6.869	6420979	49.68	347760

Figure S132. HPLC spectra of **3z**, related to **Scheme 2**.

SAMPLE INFORMATION

Sample Name:	why-g05-99-1-IA-1.5%	Acquired By:	System
Sample Type:	Unknown	Sample Set Name	
Vial:	99	Acq. Method Set:	1 5%
Injection #:	1	Processing Method	5 99 1 asy
Injection Volume:	10.00 ul	Channel Name:	2998 Ch1 254nm@1.2nm
Run Time:	100.0 Minutes	Proc. Chnl. Descr.:	2998 Ch1 254nm@1.2nm
Date Acquired:	5/6/2019 7:38:31 PM CST		
Date Processed:	7/19/2019 4:51:16 PM CST		

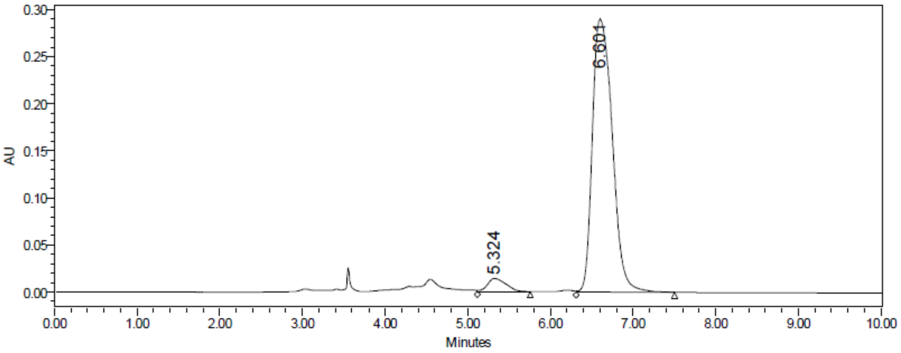

	RT	Area	% Area	Height
1	5.324	209937	4.16	13908
2	6.601	4839867	95.84	289449

Figure S133. HPLC spectra of *rac*-**6a**, related to **Scheme 3**.

SAMPLE INFORMATION

Sample Name:	why-g05-199-2-IA-10%	Acquired By:	System
Sample Type:	Unknown	Sample Set Name	
Vial:	29	Acq. Method Set:	10%
Injection #:	1	Processing Method	5 199 1
Injection Volume:	10.00 ul	Channel Name:	2998 Ch1 254nm@1.2nm
Run Time:	100.0 Minutes	Proc. Chnl. Descr.:	2998 Ch1 254nm@1.2nm
Date Acquired:	7/28/2019 9:04:15 PM CST		
Date Processed:	8/3/2019 3:33:49 PM CST		

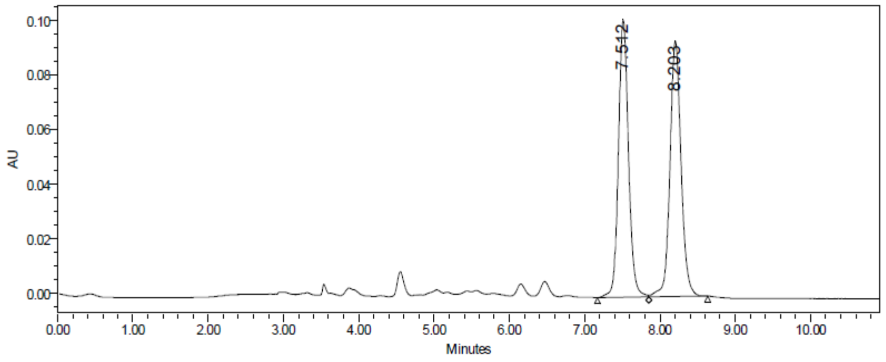

	RT	Area	% Area	Height
1	7.512	941725	49.42	101796
2	8.203	963979	50.58	93716

Figure S134. HPLC spectra of **6a**, related to **Scheme 3**.

SAMPLE INFORMATION

Sample Name:	why-g05-191-1-IA-10%	Acquired By:	System
Sample Type:	Unknown	Sample Set Name	
Vial:	54	Acq. Method Set:	10%
Injection #:	1	Processing Method	5 191 1
Injection Volume:	10.00 ul	Channel Name:	2998 Ch1 254nm@1.2nm
Run Time:	100.0 Minutes	Proc. Chnl. Descr.:	2998 Ch1 254nm@1.2nm
Date Acquired:	7/28/2019 9:19:41 PM CST		
Date Processed:	8/3/2019 3:34:59 PM CST		

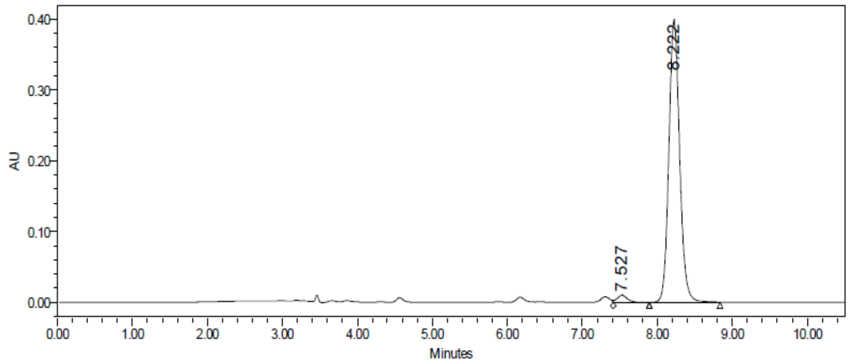

	RT	Area	% Area	Height
1	7.527	92638	2.28	9991
2	8.222	3964946	97.72	398670

Figure S135. HPLC spectra of *rac*-**6b**, related to **Scheme 3**.

SAMPLE INFORMATION

Sample Name:	why-g05-159-2-IA-10%	Acquired By:	System
Sample Type:	Unknown	Sample Set Name	
Vial:	56	Acq. Method Set:	10%
Injection #:	1	Processing Method	5 159 2
Injection Volume:	10.00 ul	Channel Name:	2998 Ch1 254nm@1.2nm
Run Time:	100.0 Minutes	Proc. Chnl. Descr.:	2998 Ch1 254nm@1.2nm
Date Acquired:	6/18/2019 6:54:46 PM CST		
Date Processed:	8/3/2019 3:26:09 PM CST		

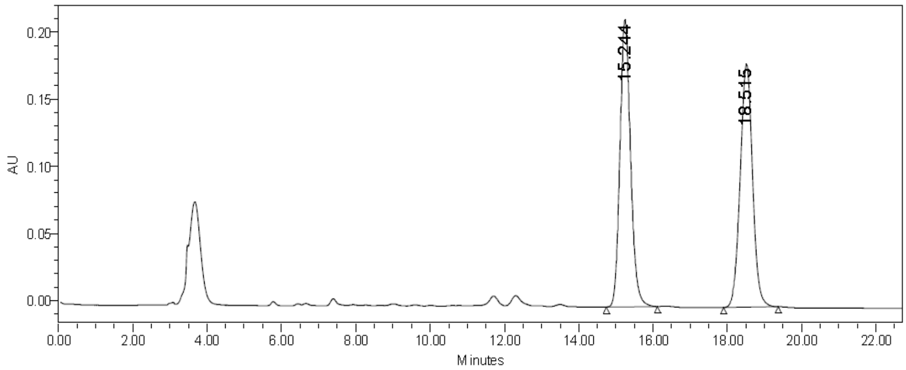

	RT	Area	% Area	Height
1	15.244	4234718	50.08	213977
2	18.515	4220834	49.92	181236

Figure S136. HPLC spectra of **6b**, related to **Scheme 3**.

SAMPLE INFORMATION

Sample Name:	why-g05-169-2-IA-10%	Acquired By:	System
Sample Type:	Unknown	Sample Set Name	
Vial:	106	Acq. Method Set:	10%
Injection #:	1	Processing Method	5 169 2
Injection Volume:	10.00 ul	Channel Name:	2998 Ch1 254nm@1.2nm
Run Time:	100.0 Minutes	Proc. Chnl. Descr.:	2998 Ch1 254nm@1.2nm
Date Acquired:	6/24/2019 6:55:34 PM CST		
Date Processed:	8/3/2019 3:28:10 PM CST		

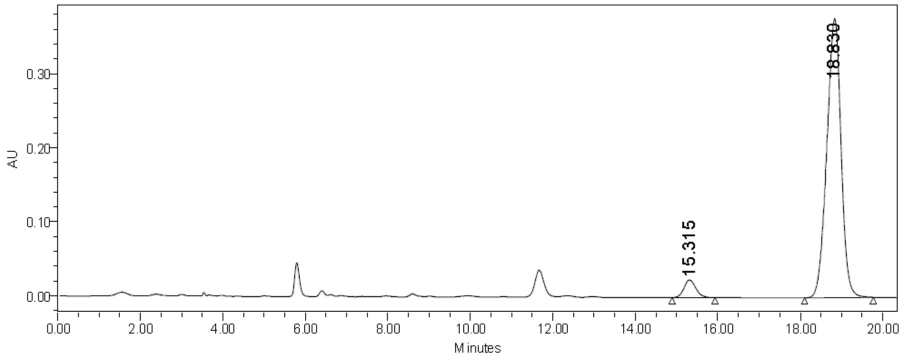

	RT	Area	% Area	Height
1	15.315	467017	4.94	23666
2	18.830	8989230	95.06	376102

Figure S137. HPLC spectra of *rac*-**6c**, related to **Scheme 3**.

SAMPLE INFORMATION

Sample Name:	why-g05-189-1-IA-10%	Acquired By:	System
Sample Type:	Unknown	Sample Set Name	
Vial:	27	Acq. Method Set:	10%
Injection #:	1	Processing Method	5 189 1
Injection Volume:	10.00 ul	Channel Name:	2998 Ch1 254nm@1.2nm
Run Time:	100.0 Minutes	Proc. Chnl. Descr.:	2998 Ch1 254nm@1.2nm
Date Acquired:	7/22/2019 8:16:51 PM CST		
Date Processed:	8/3/2019 3:32:13 PM CST		

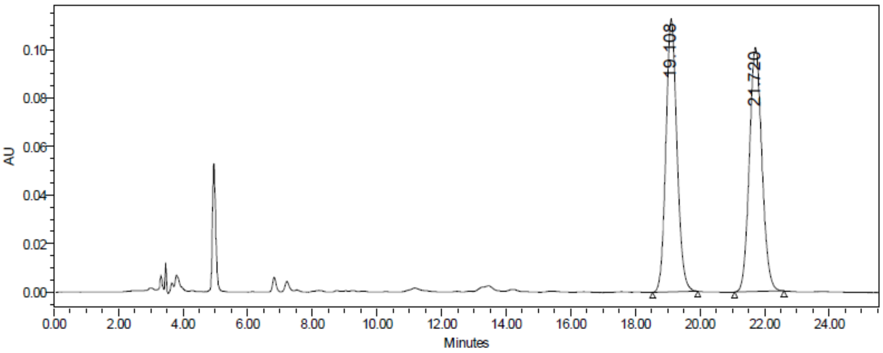

	RT	Area	% Area	Height
1	19.108	2679165	49.92	112252
2	21.720	2687341	50.08	100552

Figure S138. HPLC spectra of **6c**, related to **Scheme 3**.

SAMPLE INFORMATION

Sample Name:	why-g05-189-2-IA-10%	Acquired By:	System
Sample Type:	Unknown	Sample Set Name	
Vial:	45	Acq. Method Set:	10%
Injection #:	1	Processing Method	5 189 2
Injection Volume:	10.00 ul	Channel Name:	2998 Ch1 254nm@1.2nm
Run Time:	100.0 Minutes	Proc. Chnl. Descr.:	2998 Ch1 254nm@1.2nm
Date Acquired:	7/22/2019 2:30:04 PM CST		
Date Processed:	8/3/2019 3:32:41 PM CST		

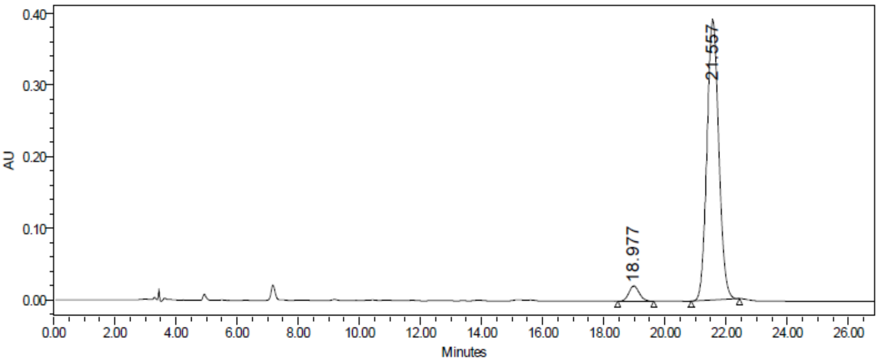

	RT	Area	% Area	Height
1	18.977	504451	4.61	21417
2	21.557	10436432	95.39	391481

Figure S139. HPLC spectra of *rac*-**6d**, related to **Scheme 3**.

SAMPLE INFORMATION

Sample Name:	why-g05-188-1-IA-10%	Acquired By:	System
Sample Type:	Unknown	Sample Set Name	
Vial:	4	Acq. Method Set:	10%
Injection #:	1	Processing Method	5 188 1
Injection Volume:	10.00 ul	Channel Name:	2998 Ch1 254nm@1.2nm
Run Time:	100.0 Minutes	Proc. Chnl. Descr.:	2998 Ch1 254nm@1.2nm
Date Acquired:	7/22/2019 7:50:10 PM CST		
Date Processed:	8/3/2019 3:31:12 PM CST		

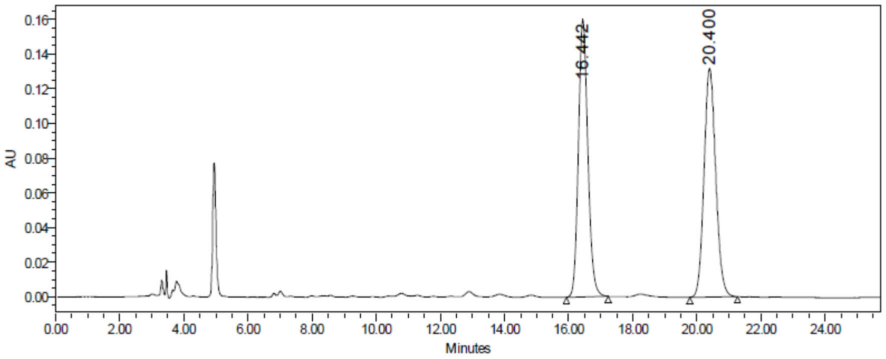

	RT	Area	% Area	Height
1	16.442	3290513	49.87	159858
2	20.400	3307902	50.13	131836

Figure S140. HPLC spectra of **6d**, related to **Scheme 3**.

SAMPLE INFORMATION

Sample Name:	why-g05-188-2-IA-10%	Acquired By:	System
Sample Type:	Unknown	Sample Set Name	
Vial:	2	Acq. Method Set:	10%
Injection #:	1	Processing Method	5 188 2
Injection Volume:	10.00 ul	Channel Name:	2998 Ch1 254nm@1.2nm
Run Time:	100.0 Minutes	Proc. Chnl. Descr.:	2998 Ch1 254nm@1.2nm
Date Acquired:	7/22/2019 2:03:03 PM CST		
Date Processed:	8/3/2019 3:31:41 PM CST		

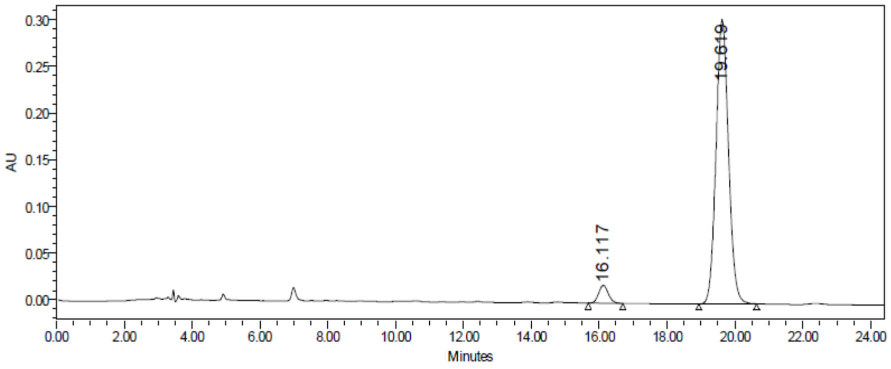

	RT	Area	% Area	Height
1	16.117	385610	4.75	19275
2	19.619	7731158	95.25	303811

Figure S141. HPLC spectra of *rac*-**6e**, related to **Scheme 3**.

SAMPLE INFORMATION

Sample Name:	why-g05-197-2-IA-10%	Acquired By:	System
Sample Type:	Unknown	Sample Set Name	
Vial:	21	Acq. Method Set:	10%
Injection #:	1	Processing Method	5 197 2
Injection Volume:	10.00 ul	Channel Name:	2998 Ch1 254nm@1.2nm
Run Time:	100.0 Minutes	Proc. Chnl. Descr.:	2998 Ch1 254nm@1.2nm
Date Acquired:	7/27/2019 6:48:52 PM CST		
Date Processed:	8/3/2019 3:38:05 PM CST		

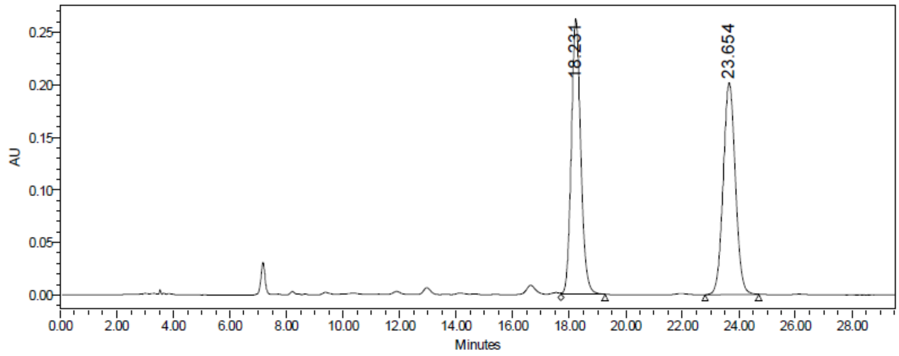

	RT	Area	% Area	Height
1	18.231	6009022	50.18	261771
2	23.654	5965484	49.82	201754

Figure S142. HPLC spectra of **6e**, related to **Scheme 3**.

SAMPLE INFORMATION

Sample Name:	why-g05-194-2-IA-10%	Acquired By:	System
Sample Type:	Unknown	Sample Set Name	
Vial:	16	Acq. Method Set:	10%
Injection #:	1	Processing Method	5 194 2
Injection Volume:	10.00 ul	Channel Name:	2998 Ch1 254nm@1.2nm
Run Time:	100.0 Minutes	Proc. Chnl. Descr.:	2998 Ch1 254nm@1.2nm
Date Acquired:	7/28/2019 9:52:27 AM CST		
Date Processed:	8/3/2019 3:38:37 PM CST		

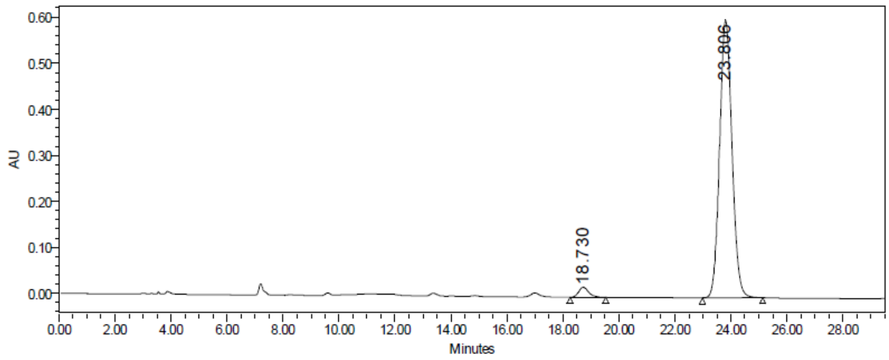

	RT	Area	% Area	Height
1	18.730	536788	2.82	21793
2	23.806	18472791	97.18	602626

Figure S143. HPLC spectra of *rac*-**6f**, related to **Scheme 3**.

SAMPLE INFORMATION

Sample Name:	why-g05-198-2-AD-H15%	Acquired By:	System
Sample Type:	Unknown	Sample Set Name	
Vial:	26	Acq. Method Set:	15%
Injection #:	1	Processing Method	5 198 2
Injection Volume:	10.00 ul	Channel Name:	2998 Ch1 254nm@1.2nm
Run Time:	100.0 Minutes	Proc. Chnl. Descr.:	2998 Ch1 254nm@1.2nm
Date Acquired:	7/27/2019 3:43:43 PM CST		
Date Processed:	8/3/2019 3:42:35 PM CST		

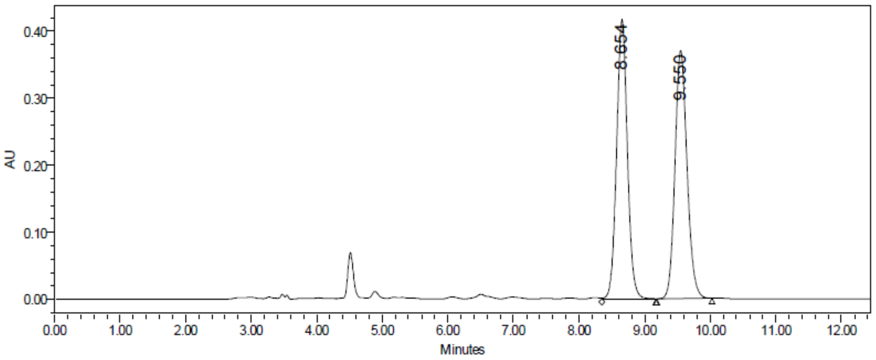

	RT	Area	% Area	Height
1	8.654	4775242	50.08	416049
2	9.550	4759319	49.92	370063

Figure S144. HPLC spectra of **6f**, related to **Scheme 3**.

SAMPLE INFORMATION

Sample Name:	why-g05-195-2-AD-H15%	Acquired By:	System
Sample Type:	Unknown	Sample Set Name	
Vial:	63	Acq. Method Set:	15%
Injection #:	1	Processing Method	5 195 2
Injection Volume:	10.00 ul	Channel Name:	2998 Ch1 254nm@1.2nm
Run Time:	100.0 Minutes	Proc. Chnl. Descr.:	2998 Ch1 254nm@1.2nm
Date Acquired:	7/27/2019 3:57:29 PM CST		
Date Processed:	8/3/2019 3:42:58 PM CST		

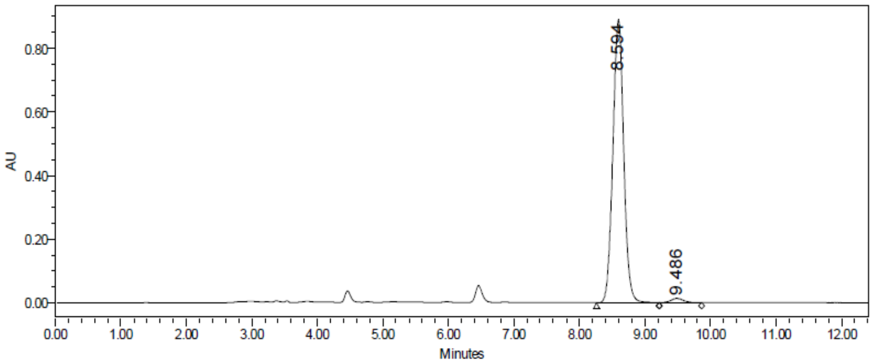

	RT	Area	% Area	Height
1	8.594	10249546	98.27	888091
2	9.486	180751	1.73	13529

Figure S145. HPLC spectra of *rac*-**6g**, related to **Scheme 3**.

SAMPLE INFORMATION

Sample Name:	why-g05-198-1-AD-H15%	Acquired By:	System
Sample Type:	Unknown	Sample Set Name	
Vial:	104	Acq. Method Set:	15%
Injection #:	1	Processing Method	5 198 1
Injection Volume:	10.00 ul	Channel Name:	2998 Ch1 254nm@1.2nm
Run Time:	100.0 Minutes	Proc. Chnl. Descr.:	2998 Ch1 254nm@1.2nm
Date Acquired:	7/27/2019 2:50:56 PM CST		
Date Processed:	8/3/2019 3:41:13 PM CST		

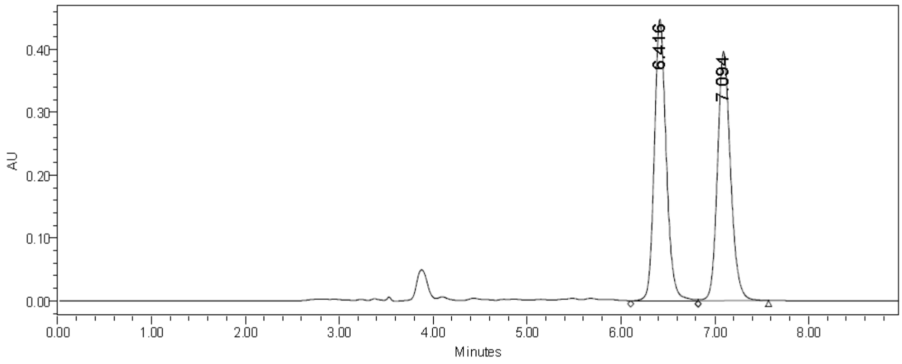

	RT	Area	% Area	Height
1	6.416	3934302	50.62	447162
2	7.094	3838527	49.38	395811

Figure S146. HPLC spectra of **6g**, related to **Scheme 3**.

SAMPLE INFORMATION

Sample Name:	why-g05-195-1-AD-H15%	Acquired By:	System
Sample Type:	Unknown	Sample Set Name	
Vial:	20	Acq. Method Set:	15%
Injection #:	1	Processing Method	5 195 1
Injection Volume:	10.00 ul	Channel Name:	2998 Ch1 254nm@1.2nm
Run Time:	100.0 Minutes	Proc. Chnl. Descr.:	2998 Ch1 254nm@1.2nm
Date Acquired:	7/27/2019 3:01:35 PM CST		
Date Processed:	8/3/2019 3:41:58 PM CST		

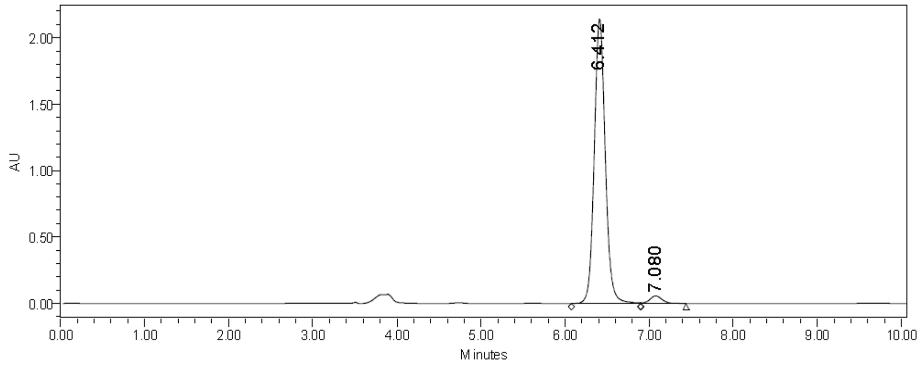

	RT	Area	% Area	Height
1	6.412	19561736	97.09	2137082
2	7.080	585287	2.91	57468

Figure S147. HPLC spectra of *rac*-**6h**, related to **Scheme 3**.

SAMPLE INFORMATION

Sample Name:	why-g05-197-1-IA-10%	Acquired By:	System
Sample Type:	Unknown	Sample Set Name	
Vial:	73	Acq. Method Set:	10%
Injection #:	2	Processing Method	5 197 1
Injection Volume:	10.00 ul	Channel Name:	2998 Ch1 254nm@1.2nm
Run Time:	100.0 Minutes	Proc. Chnl. Descr.:	2998 Ch1 254nm@1.2nm
Date Acquired:	7/28/2019 7:29:54 PM CST		
Date Processed:	8/3/2019 3:36:19 PM CST		

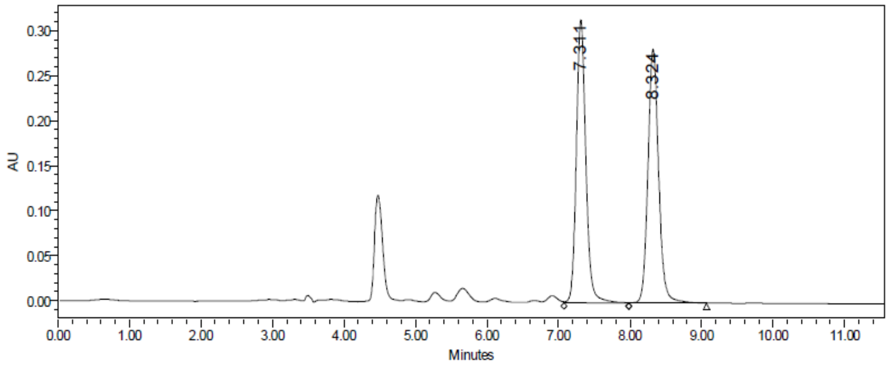

	RT	Area	% Area	Height
1	7.311	2912330	49.94	314167
2	8.324	2919807	50.06	281933

Figure S148. HPLC spectra of **6h**, related to **Scheme 3**.

SAMPLE INFORMATION

Sample Name:	why-g05-194-1-IA-10%	Acquired By:	System
Sample Type:	Unknown	Sample Set Name	
Vial:	97	Acq. Method Set:	10%
Injection #:	1	Processing Method	5 194 1
Injection Volume:	10.00 ul	Channel Name:	2998 Ch1 254nm@1.2nm
Run Time:	100.0 Minutes	Proc. Chnl. Descr.:	2998 Ch1 254nm@1.2nm
Date Acquired:	7/28/2019 7:42:43 PM CST		
Date Processed:	8/3/2019 3:37:24 PM CST		

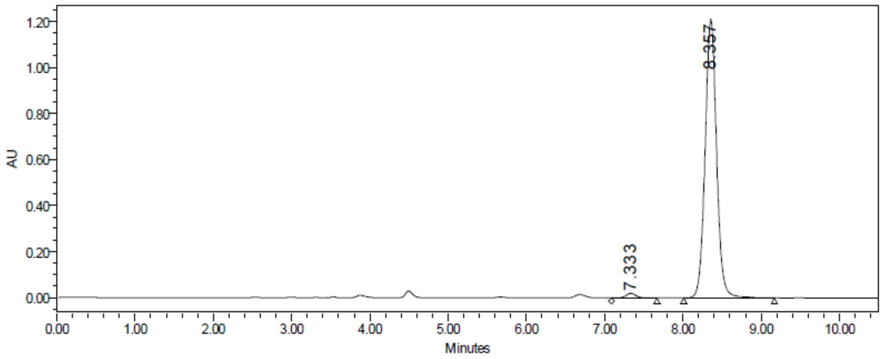

	RT	Area	% Area	Height
1	7.333	176263	1.42	19997
2	8.357	12249569	98.58	1208965

Figure S149. HPLC spectra of *rac*-**7**, related to **Scheme 4**.

SAMPLE INFORMATION

Sample Name:	why-g06-37-1-IA-5%	Acquired By:	System
Sample Type:	Unknown	Sample Set Name	
Vial:	2	Acq. Method Set:	5%
Injection #:	1	Processing Method	6 37 1 rac
Injection Volume:	10.00 ul	Channel Name:	2998 Ch1 254nm@1.2nm
Run Time:	100.0 Minutes	Proc. Chnl. Descr.:	2998 Ch1 254nm@1.2nm
Date Acquired:	8/26/2019 8:45:50 PM CST		
Date Processed:	9/24/2019 2:55:43 PM CST		

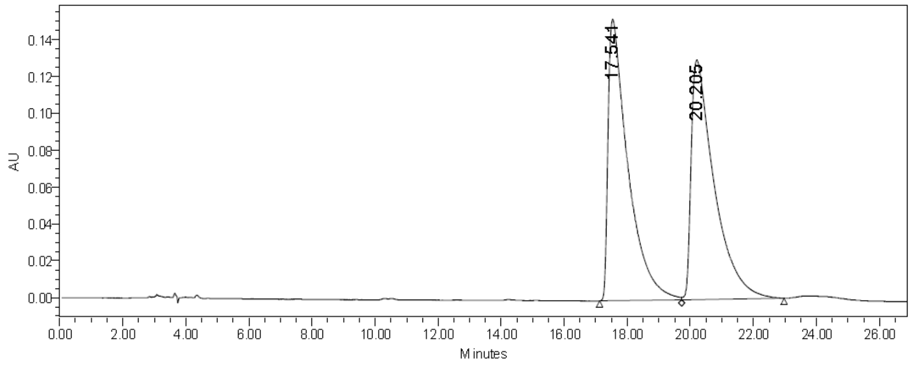

	RT	Area	% Area	Height
1	17.541	6673637	50.20	152291
2	20.205	6620390	49.80	129656

Figure S150. HPLC spectra of **7**, related to **Scheme 4**.

SAMPLE INFORMATION

Sample Name:	why-g06-37-1-asy-IA-5%	Acquired By:	System
Sample Type:	Unknown	Sample Set Name	
Vial:	12	Acq. Method Set:	5%
Injection #:	1	Processing Method	6 37 1 asy
Injection Volume:	10.00 ul	Channel Name:	2998 Ch1 254nm@1.2nm
Run Time:	100.0 Minutes	Proc. Chnl. Descr.:	2998 Ch1 254nm@1.2nm
Date Acquired:	9/21/2019 10:09:33 AM CST		
Date Processed:	9/24/2019 3:03:36 PM CST		

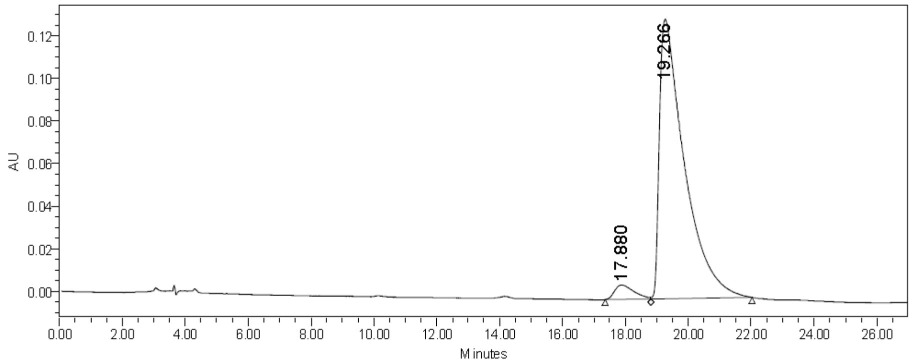

	RT	Area	% Area	Height
1	17.880	289188	3.85	6737
2	19.266	7230632	96.15	131152

Figure S151. HPLC spectra of *rac*-**8**, related to **Scheme 4**.

SAMPLE INFORMATION

Sample Name:	why-g06-38-1-AD-H-10%	Acquired By:	System
Sample Type:	Unknown	Sample Set Name	
Vial:	72	Acq. Method Set:	10%
Injection #:	1	Processing Method	6 38 1 rac
Injection Volume:	10.00 ul	Channel Name:	2998 Ch1 254nm@1.2nm
Run Time:	100.0 Minutes	Proc. Chnl. Descr.:	2998 Ch1 254nm@1.2nm
Date Acquired:	8/28/2019 3:59:26 PM CST		
Date Processed:	9/24/2019 2:57:11 PM CST		

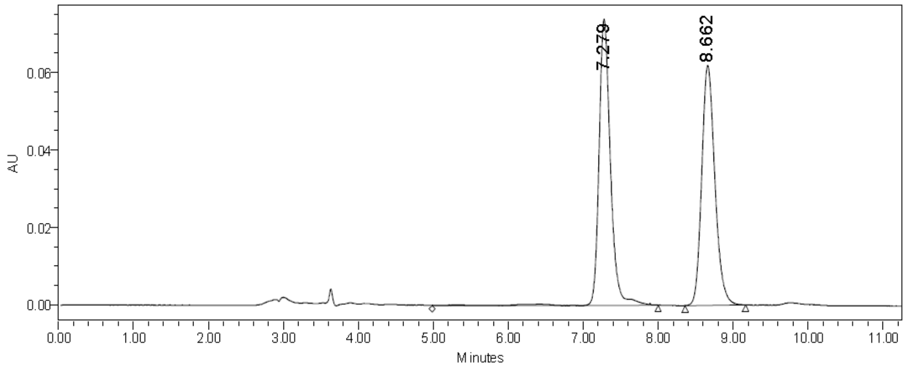

	RT	Area	% Area	Height
1	7.279	788784	50.95	73830
2	8.662	759429	49.05	61938

Figure S152. HPLC spectra of **8**, related to **Scheme 4**.

SAMPLE INFORMATION

Sample Name:	why-g06-38-1-asy-AD-H-10%	Acquired By:	System
Sample Type:	Unknown	Sample Set Name	
Vial:	2	Acq. Method Set:	10%
Injection #:	1	Processing Method	6 38 1 ASY
Injection Volume:	10.00 ul	Channel Name:	2998 Ch1 254nm@1.2nm
Run Time:	100.0 Minutes	Proc. Chnl. Descr.:	2998 Ch1 254nm@1.2nm
Date Acquired:	9/23/2019 11:27:03 AM CST		
Date Processed:	9/23/2019 2:37:07 PM CST		

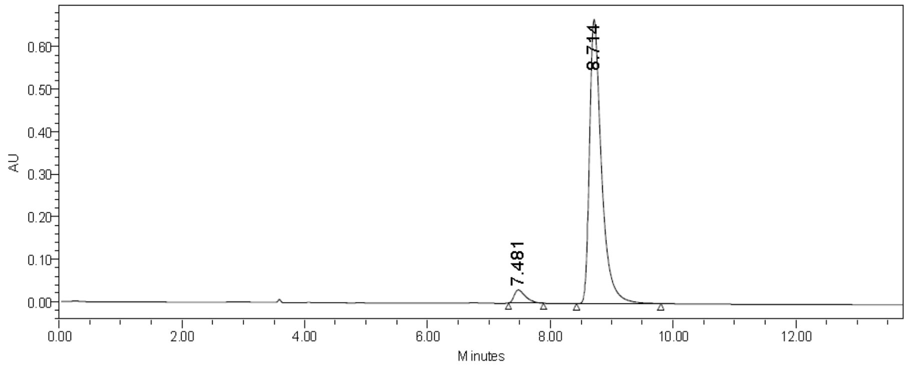

	RT	Area	% Area	Height
1	7.481	402073	4.23	30981
2	8.714	9113769	95.77	667895

Figure S153. HPLC spectra of *rac*-**9**, related to **Scheme 4**.

SAMPLE INFORMATION

Sample Name:	why-g06-39-1-rac-AS-H-30%	Acquired By:	System
Sample Type:	Unknown	Sample Set Name	
Vial:	21	Acq. Method Set:	30%quanbo
Injection #:	1	Processing Method	6 39 1 rac
Injection Volume:	20.00 ul	Channel Name:	254.0nm
Run Time:	100.0 Minutes	Proc. Chnl. Descr.:	2998 PDA 254.0 nm (2998
Date Acquired:	9/20/2019 2:14:32 PM CST		
Date Processed:	9/24/2019 2:59:48 PM CST		

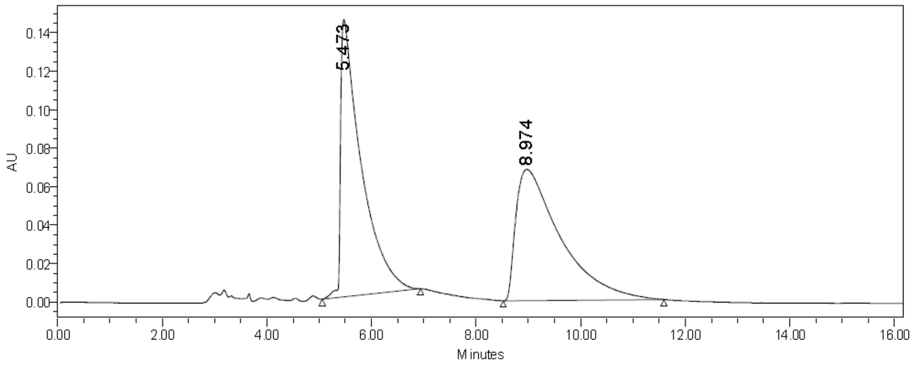

	RT	Area	% Area	Height
1	5.473	3973711	49.57	144135
2	8.974	4042019	50.43	68201

Figure S154. HPLC spectra of **9**, related to **Scheme 4**.

SAMPLE INFORMATION

Sample Name:	why-g06-39-1-asy-AS-H-30%	Acquired By:	System
Sample Type:	Unknown	Sample Set Name	
Vial:	60	Acq. Method Set:	30%quanbo
Injection #:	1	Processing Method	6 39 1 asy
Injection Volume:	20.00 ul	Channel Name:	254.0nm
Run Time:	100.0 Minutes	Proc. Chnl. Descr.:	2998 PDA 254.0 nm (2998
Date Acquired:	9/20/2019 2:31:54 PM CST		
Date Processed:	9/24/2019 3:00:50 PM CST		

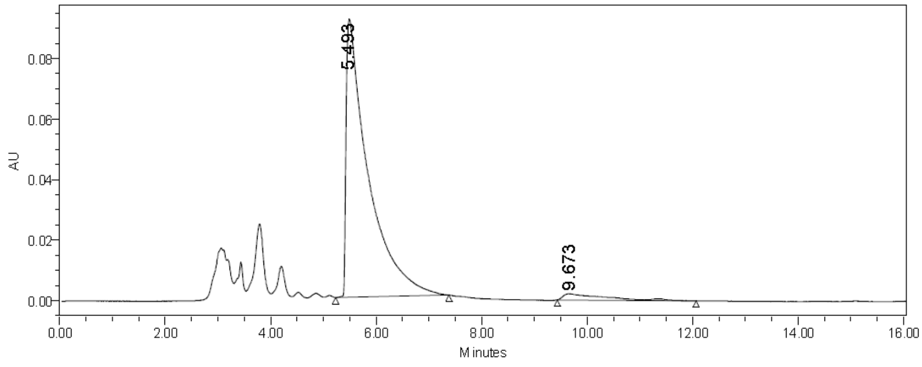

	RT	Area	% Area	Height
1	5.493	2669826	95.84	91846
2	9.673	115750	4.16	2036

Figure S155. HPLC spectra of *rac*-**10**, related to **Table 1**.

SAMPLE INFORMATION

Sample Name:	why-g06-61-2--IA-1%	Acquired By:	System
Sample Type:	Unknown	Sample Set Name	
Vial:	1	Acq. Method Set:	1%
Injection #:	1	Processing Method	6 61 2
Injection Volume:	10.00 ul	Channel Name:	2998 Ch1 254nm@1.2nm
Run Time:	100.0 Minutes	Proc. Chnl. Descr.:	2998 Ch1 254nm@1.2nm
Date Acquired:	10/23/2019 7:49:52 PM CST		
Date Processed:	10/29/2019 7:48:57 PM CST		

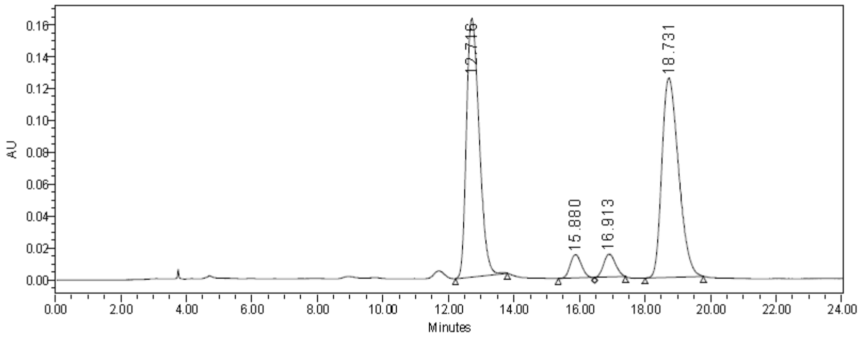

	RT	Area	% Area	Height
1	12.716	4399203	46.14	161787
2	15.880	343025	3.60	14577
3	16.913	360347	3.78	14351
4	18.731	4431866	46.48	124940

Figure S156. HPLC spectra of **10**, related to **Table 1**.

SAMPLE INFORMATION

Sample Name:	why-g06-61-1--IA-1%	Acquired By:	System
Sample Type:	Unknown	Sample Set Name	
Vial:	50	Acq. Method Set:	1%
Injection #:	1	Processing Method	6 61 1
Injection Volume:	10.00 ul	Channel Name:	2998 Ch1 254nm@1.2nm
Run Time:	100.0 Minutes	Proc. Chnl. Descr.:	2998 Ch1 254nm@1.2nm
Date Acquired:	10/23/2019 4:46:41 PM CST		
Date Processed:	10/29/2019 7:53:12 PM CST		

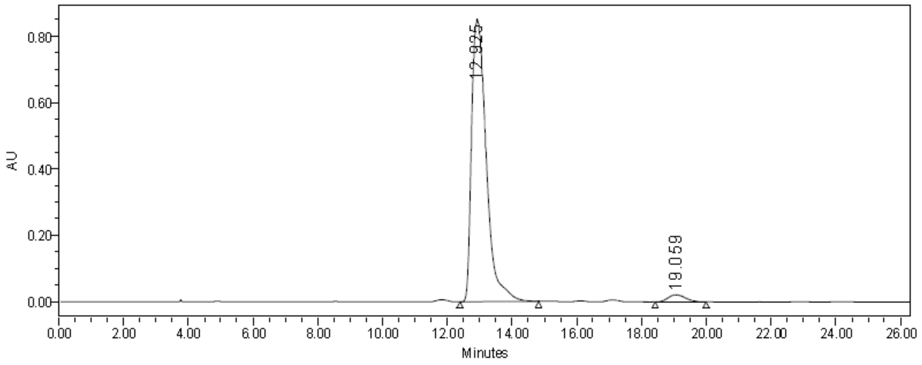

	RT	Area	% Area	Height
1	12.925	26610987	97.04	850338
2	19.059	811472	2.96	21383

Supplemental figures and tables for X-Ray structures

Figure S157. X-Ray crystal data of **3i**, related to **Scheme 2.**

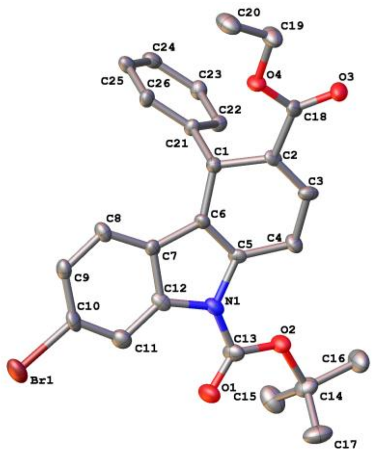

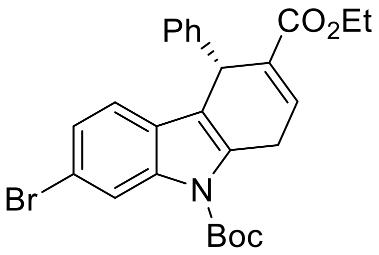

(**3i**)

Chemical formula	$C_{26}H_{26}BrNO_4$
Formula weight	496.39
Space group	P1
Z	4
α, Å	9.1215(5)
b, Å	13.5175(7)
c, Å	19.4869(10)
α, °	99.498(3)
β, °	93.402(3)
γ, °	96.017(3)
V, $Å^3$	2349.5(2)
ρ,g/cm^3	1.403

Figure S158.. X-Ray crystal data of **7**, related to **Scheme 4.**

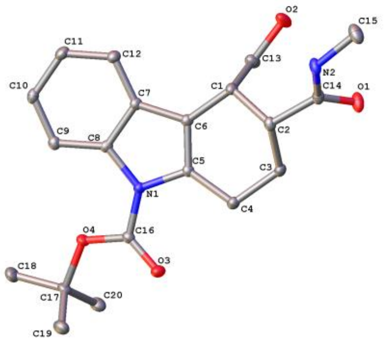

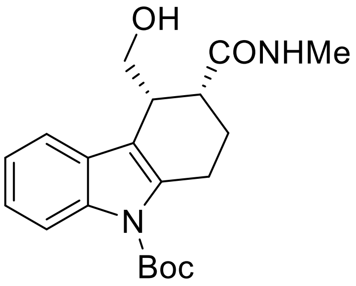

(**7**)

Chemical formula	$C_{20}H_{26}N_2O_4$
Formula weight	358.43
Space group	$P2_12_12_1$
Z	4
α, Å	7.87500(10)
b, Å	10.1406(2)
c, Å	23.0161(4)
α, °	90
β, °	90
γ, °	90
V, $Å^3$	1838.00(5)
ρ,g/cm^3	1.295

Transparent Methods

General Information

Unless otherwise noted, all reagents were purchased from commercial suppliers and used without further purification. NMR spectra were recorded on a Brucker-400 MHz spectrometer. Chemical shifts (δ) are given in ppm relative to TMS. The residual solvent signals were used as references and the chemical shifts converted to the TMS scale ($CHCl_3$: δ 7.26 for proton and δ 77.16 for carbon; DMSO: δ 2.49 for proton and δ 39.51 for carbon; Acetone: δ 2.05 for proton and δ 206.68 for carbon). Multiplicities were given as: s (singlet); d (doublet); t (triplet); q (quartet); dd (doublet of doublets); dt (doublet of triplets); m (multiplets); brs (broad signal). Coupling constants are reported as a *J* value in Hz. High resolution mass spectral analysis (HRMS) was performed on Waters XEVO G2 Q-TOF. The measurement of enantiomeric excesses was performed on Waters-Alliance (2998. Photodiode Array Detector, UV detection monitored at 254 nm). Chiralpak IA, AD-H and AS-H columns were purchased from Daicel Chemical Industries, LTD. The absolute configuration of 3i and 7 were assigned by the X-ray analysis. Optical rotations were determined at 589 nm (sodium D line) by using a Perkin-Elmer-343 polarimeter.

Preparation of Substrates

Preparation of 3-Nitroindoles 1 and 3-Nitrobenzothiophenes 5

The 3-nitroindoles **1**(You et al, 2018), 3-nitrobenzothiophenes **5**(You et al, 2017) and Allenoates **2**(Kwon et al, 2007) were synthesized according to the literature.

Optimize reaction conditions

Table S1: Additives effect the reaction of 3-nitroindoles 1a and allenoates 2a, related to Table 1[a].

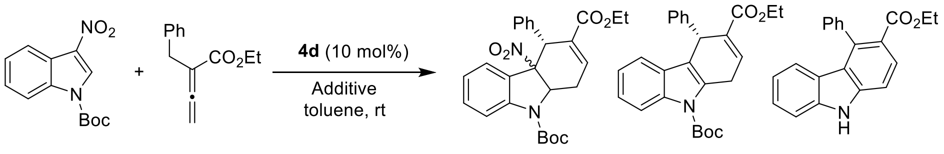

Entry	Additive	T (°C)	t **(h)**	**10** [%][b]	*ee* [%][c]	**3a**[%][b]	*ee* [%][c]	**11** [%][b]
1	-	rt	-	74	94	21	94	-
2	Silica gel (200 mg)	rt	3	8	94	92	94	-
3	$Sc(OTf)_3$ (20 mol%)	50	6	-	-	10	90	59
4	$SnCl_2$ (20 mol%)	50	6	24	94	44	94	
5	PhCOOH (20 mol%)	50	3	67	94	15	94	
6	Et_3N (1.0 eqive)	50	3	66	94	16	94	
7	DABCO (1.0 eqive)	50	3	52	94	14	94	

[a] Reactions were conducted with **1a** (0.1 mmol), **2a** (0.15 mmol) and catalyst **4d** (0.01 mmol) in toluene(1.0 mL) at room temperature. [b] Yield of the isolated product after purification by chromatography on silica gel. [c] Enantiomeric excess determined by HPLC

Table S2: Optimization of the reaction of 3-nitrobenzothiophenes 5a and allenoate 2h, related to Scheme 3[a].

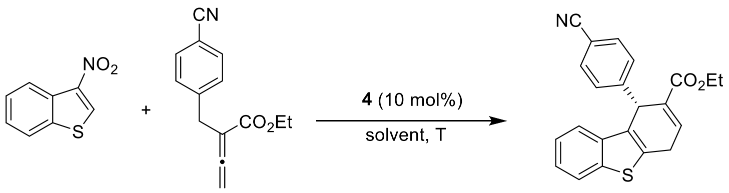

5a **2h** **6a**

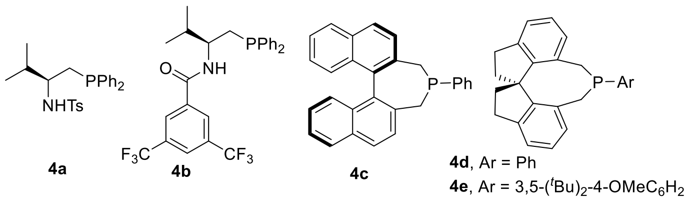

4a **4b** **4c** **4d**, Ar = Ph

4e, Ar = 3,5-(^{t}Bu)$_2$-4-OMeC$_6$H$_2$

Entry	**4**	T (°C)	solvent	**6a** [%][b]	*ee* [%][d]
1	**4a**	rt	toluene	-	-
2	**4b**	rt	toluene	-	-
3	**4c**	rt	toluene	trace	-
4	**4d**	rt	toluene	50	82
5	**4e**	rt	toluene	24	12
6	**4d**	rt	THF	49	87
7	**4d**	rt	DCM	36	86
8	**4d**	rt	dioxane	trace	-
9	**4d**	0	THF	65	90
10	**4d**	0	toluene	72	90
11	**4d**	-20	toluene	trace	-

[a] Reactions were conducted with **5a** (0.1 mmol), **2h** (0.15 mmol) and catalyst **4** (0.01 mmol) in toluene(1.0 mL). [b] Yield of the isolated product after purification by chromatography on silica gel. [c] Enantiomeric excess determined by HPLC analysis.

General procedure

All the racemic products were obtained by use of Cy_3P as catalyst.

Scheme S1. General procedure for phosphine-catalyzed enantioselective [4+2] annulation reaction of 3-nitroindoles 1 and allenoates 2, related to Scheme 2.

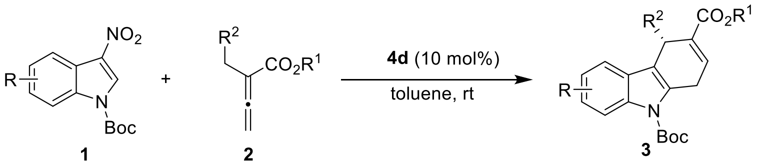

1 **2** **3**

A dried tube with a magnetic stir bar was charged with 3-nitroindole derivative **1** (0.10 mmol), allenoate derivative **2** (0.15 mmol, 1.5 equiv.), catalyst **4d** (10 mol%), followed by the addition of toluene (1.0 mL), and the reaction mixture was stirred at room temperature. When the reaction was finished (determined by TLC). The mixture were added silica gel and toluene (1.0 mL) continued stir at room temperature when the aromatization process was finished (determined by TLC). Then solvent was evaporated and the residue was purified by column chromatography on silica gel using hexane/ethyl acetate as the eluent to afford the products **3**.

Scheme S2. General procedure for phosphine-catalyzed enantioselective [4+2]

annulation reaction of 3-nitrobenzothiophene 5 and allenoate 2, related to Scheme 3.

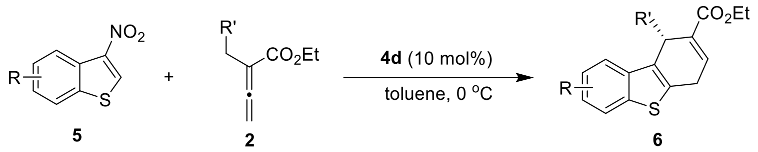

A dried tube with a magnetic stir bar was charged with 3-nitrobenzothiophenes derivative **5** (0.10 mmol), allenoate derivative **2** (0.15 mmol, 1.5 equiv.), catalyst **4d** (10 mol%), followed by the addition of toluene (1.0 mL), and the reaction mixture was stirred at 0 ℃. When the reaction was finished (determined by TLC). The mixture were added silica gel and toluene (1.0 mL) continued stir at room temperature when the aromatization process was finished (determined by TLC). Then solvent was evaporated and the residue was purified by column chromatography on silica gel using hexane/ethyl acetate as the eluent to afford the products **6**.

Scheme S3. 1-mol scale reaction, related to Scheme 2.

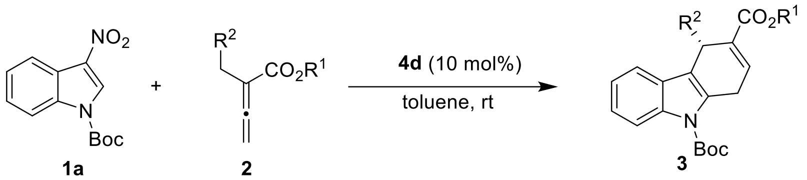

A dried tube with a magnetic stir bar was charged with 3-nitroindole derivative **1a** (1.0 mmol), allenoate derivative **2o** (1.5 mmol, 1.5 equiv.), catalyst **4d** (10 mol%), followed by the addition of toluene (10.0 mL), and the reaction mixture was stirred at room temperature. When the reaction was finished (determined by TLC). The mixture were added silica gel (2.0 g) and toluene (10.0 mL) continued stir at room temperature when the aromatization was finished (determined by TLC). Then solvent was evaporated and the residue was purified by column chromatography on silica gel using hexane/ethyl acetate as the eluent to afford the products **3x** (251.4 mg, 56%, 92% *ee*).

Scheme S3. Synthesis procedure of derivatization reaction, related to Scheme 4.

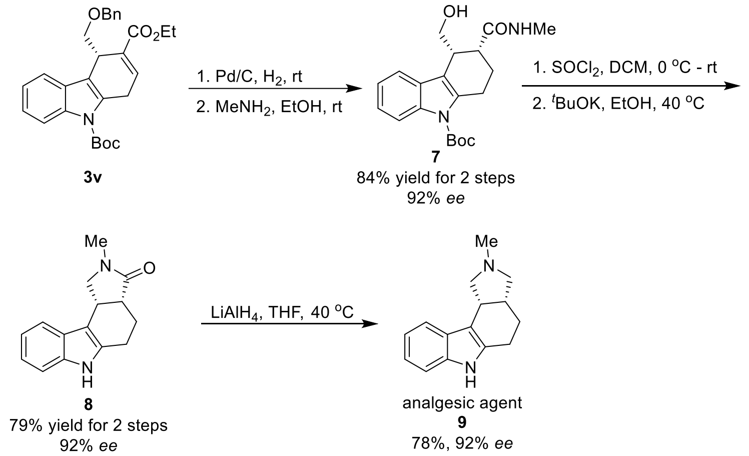

A dried tube with a magnetic stir bar was charged with 3-nitroindole derivative **1a** (1.00 mmol), allenoate derivative **2m** (1.50 mmol, 1.5 equiv.), catalyst **4d** (10 mol%), followed by the addition

of toluene (10.0 mL), and the reaction mixture was stirred at room temperature. When the reaction was finished (determined by TLC). The mixture were added silica gel (2.0 g) and toluene (10.0 mL) continued stir at room temperature when the aromatization process was finished (determined by TLC). Then solvent was evaporated and the residue was purified by column chromatography on silica gel using hexane/ethyl acetate as the eluent to afford the products **3v** (261.6 mg, 57%, 92% *ee*).

A suspension of **3v** (261.6 mg, 0.57 mmol) and 10% palladium on carbon (130.0 mg) in MeOH (10.0 mL) was maintained under an atmosphere of hydrogen gas for 8 h at rt. The insoluble solids were removed by filtration and the filtrate was concentrated. The residue was dissolved in $MeNH_2$ (30 wt. % in absolute EtOH 4.0 mL), and the resulting mixture was stirred 1 h. Then solvent was evaporated and the residue was purified by column chromatography on silica gel using hexane/ethyl acetate as the eluent to afford the product **7** (179.2 mg, 84%, 92% *ee*).

To a solution of **7** (179.2 mg, 0.48 mmol) in anhydrous DCM (5 mL) was slowly added $SOCl_2$ (1 mol/L in DCM, 2.0 mL) at 0 ℃. The suspension was allowed to warm to room temperature and continues to stir 2 h. After that, the reaction mixture was reduced in vacuo. The residue was dissolved in EtOH (5.0 mL) and potassium tert-butoxide (336.0 mg, 3.0 mmol) was added and the reaction stirred at 40 ℃ for 36 h. The solvent was removed and the residue was purified by column chromatography using MeOH/DCM as the eluent to give **8** (91.2 mg, 79%, 92% *ee*).

To a solution of **7** (28.8 mg, 0.12 mmol) in anhydrous THF (5 mL) was added $LiAlH_4$ (45.6 mg, 1.2 mmol) at 0 ℃. The suspension was allowed to warm to room temperature and continues to stir at 40 ℃ for 36 h. After that, saturated aqueous Na_2SO_4 (4 mL) was added. The solid formed was filtered and washed with DCM. The organic layers were combined and dried with $MgSO_4$. The solvent was removed and the residue was purified by column chromatography (DCM/MeOH/Et_3N = 100/5/1) to give **8** (21.2 mg, 78%, 92% *ee*).

Characterization of products

9-(Tert-butyl) 3-ethyl (*S*)-4-phenyl-1,4-dihydro-9H-carbazole-3,9-dicarboxylate (3a)

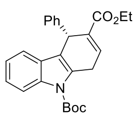

Step 1: 18 h; Step 2: 3 h (Silica gel 200 mg); Total yield: 38.4 mg (92%); **^{1}H NMR (400 MHz, CDCl$_3$)** δ 8.07 (d, *J* = 8.3 Hz, 1H), 7.35 – 7.30 (m, 2H), 7.24 – 7.15 (m, 5H), 7.13 – 7.03 (m, 2H), 5.18 (t, *J* = 5.9 Hz, 1H), 4.18 – 4.00 (m, 4H), 1.70 (s, 9H), 1.21 (t, *J* = 7.1 Hz, 3H). **^{13}C NMR (100 MHz, CDCl$_3$)** δ 166.41, 150.73, 143.15, 136.25, 134.87, 132.60, 130.82, 128.95, 128.27, 128.24, 126.62, 123.91, 122.73, 118.90, 118.13, 115.51, 84.03, 60.69, 40.49, 28.57, 28.43, 14.21. **ESI-MS: calculated [$C_{26}H_{27}NO_4$ + Na]$^+$: 440.1832, found: 440.1833.** $[\alpha]^{20}_D$= 26.8 (c = 0.96, CH_2Cl_2). The product was analyzed by HPLC to determine the enantiomeric excess: 94% *ee* (CHIRALPAK IA, hexane/*i*-PrOH =97/3, detector: 254 nm, T = 30 ℃, flow rate: 1 mL/min), t_1(minor) = 5.72 min, t_2(major) =7.46 min.

9-(Tert-butyl) 3-ethyl (*S*)-6-methyl-4-phenyl-1,4-dihydro-9H-carbazole-3,9-dicarboxy-

late (3b)

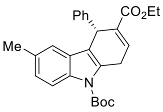

Step 1: 20 h; Step 2: 2 h (Silica gel 200 mg); Total yield: 36.3 mg (84%); **^{1}H NMR (400 MHz, $CDCl_3$)** δ 7.93 (d, *J* = 8.4 Hz, 1H), 7.36 – 7.30 (m, 2H), 7.24 – 7.17 (m, 3H), 7.14 – 7.08 (m, 1H), 7.05 – 6.97 (m, 2H), 5.16 (t, *J* = 5.8 Hz, 1H), 4.18 – 4.00 (m, 4H), 2.31 (s, 3H), 1.69 (s, 9H), 1.22 (t, *J* = 7.1 Hz, 3H). **^{13}C NMR (100 MHz, $CDCl_3$)** δ 166.46, 150.78, 143.24, 134.93, 134.45, 132.72, 132.15, 130.89, 128.95, 128.42, 128.28, 126.59, 125.25, 118.86, 117.93, 115.17, 83.85, 60.70, 40.45, 28.62, 28.47, 21.46, 14.23. **ESI-MS: calculated $[C_{27}H_{29}NO_4+ Na]^+$:454.1989, found: 454.1989.** $[\alpha]^{20}_{D}$= 4.8 (c = 0.98, CH_2Cl_2). The product was analyzed by HPLC to determine the enantiomeric excess: 92% *ee* (CHIRALPAK IA, hexane/*i*-PrOH =97/3, detector: 254 nm, T = 30 ℃, flow rate: 1 mL/min), t_1(minor) = 5.12 min, t_2(major) =10.56 min.

9-(Tert-butyl) 3-ethyl (*S*)-6-fluoro-4-phenyl-1,4-dihydro-9H-carbazole-3,9-dicarboxylate (3c)

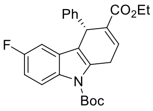

Step 1: 24 h; Step 2: 4 h (Silica gel 400 mg); Total yield: 22.8 mg (52%); **^{1}H NMR (400 MHz, $CDCl_3$)** δ 8.05 – 7.97 (m, 1H), 7.34 – 7.26 (m, 2H), 7.25 – 7.18 (m, 3H), 7.17 – 7.10 (m, 1H), 6.93 – 6.83 (m, 2H), 5.12 (t, *J* = 5.9 Hz, 1H), 4.18 – 3.99 (m, 4H), 1.70 (s, 9H), 1.21 (t, *J* = 7.1 Hz, 3H). **^{13}C NMR (100 MHz, $CDCl_3$)** δ 166.31, 159.13 (*J* = 239.3 Hz), 150.46, 142.75, 134.62, 132.61, 132.51, 129.27, 129.18, 128.89, 128.43, 126.84, 117.93 (d, *J* = 3.8 Hz), 116.48 (d, *J* = 8.9 Hz), 111.46 (d, *J* = 24.8 Hz), 104.62 (d, *J* = 23.9 Hz). 84.37, 60.78, 40.46, 28.63, 28.44, 14.22. **^{19}F NMR (377 MHz, $CDCl_3$)** δ -120.41. **ESI-MS: calculated $[C_{26}H_{27}FNO_4 + H]^+$: 436.1919, found: 436.1924.** $[\alpha]^{20}_{D}$= 21.3 (c = 1.07, CH_2Cl_2). The product was analyzed by HPLC to determine the enantiomeric excess: 91% *ee* (CHIRALPAK IA, hexane/*i*-PrOH =98.5/1.5, detector: 254 nm, T = 30 ℃, flow rate: 1 mL/min), t_1(minor) = 7.04 min, t_2(major) =9.21 min.

9-(Tert-butyl) 3-ethyl (*S*)-6-chloro-4-phenyl-1,4-dihydro-9H-carbazole-3,9-dicarboxylate (3d)

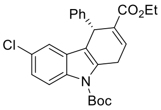

Step 1: 24 h; Step 2: 12 h (Silica gel 400 mg); Total yield: 34.4 mg (76%); **^{1}H NMR (400 MHz, $CDCl_3$)** δ 7.99 (d, *J* = 8.8 Hz, 1H), 7.34 – 7.27 (m, 2H), 7.26 – 7.17 (m, 4H), 7.17 – 7.10 (m, 2H), 5.13 (t, *J* = 5.8 Hz, 1H), 4.18 – 3.99 (m, 4H), 1.70 (s, 9H), 1.21 (t, *J* = 7.1 Hz, 3H). **^{13}C NMR (100 MHz, $CDCl_3$)** δ 166.25, 150.36, 142.70, 134.70, 134.53, 132.56, 132.26, 129.45, 128.85, 128.45, 128.32, 126.87, 124.06, 118.49, 117.59, 116.57, 84.55, 60.79, 40.34, 28.57, 28.42, 14.21. **ESI-MS: calculated $[C_{26}H_{26}ClNO_4 + H]^+$: 452.1623, found: 452.1628.** $[\alpha]^{20}_{D}$= 24.3 (c = 0.97, CH_2Cl_2). The product was analyzed by HPLC to determine the enantiomeric excess: 92% *ee* (CHIRALPAK IA, hexane/*i*-PrOH =98.5/1.5, detector: 254 nm, T = 30 ℃, flow rate: 1 mL/min), t_1(minor) = 7.00 min, t_2(major) =9.44 min.

9-(Tert-butyl) 3-ethyl (*S*)-6-bromo-4-phenyl-1,4-dihydro-9H-carbazole-3,9-dicarboxy-late (3e)

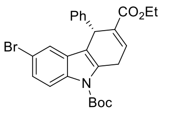

Step 1: 24 h; Step 2: 16 h (Silica gel 400 mg); Total yield: 30.3 mg (61%); **^{1}H NMR (400 MHz, $CDCl_3$)** δ 7.95 (d, *J* = 8.8 Hz, 1H), 7.35 (d, *J* = 1.8 Hz, 1H), 7.32 – 7.20 (m, 6H), 7.17 – 7.10 (m, 1H), 5.12 (t, *J* = 5.8 Hz, 1H), 4.21 – 3.99 (m, 4H), 1.70 (s, 9H), 1.21 (t, *J* = 7.1 Hz, 3H). **^{13}C NMR (100 MHz, $CDCl_3$)** δ 166.24, 150.34, 142.68, 135.08, 134.51, 132.57, 132.13, 129.95, 128.83, 128.46, 126.88, 126.77, 121.52, 117.51, 116.99, 116.09, 84.59, 60.79, 40.31, 28.54, 28.42, 14.21. **ESI-MS: calculated [$C_{26}H_{26}BrNO_4$ + H]$^+$: 496.1118, found: 496.1100.** $[\alpha]^{20}_{D}$= -38.3 (c = 1.01, CH_2Cl_2). The product was analyzed by HPLC to determine the enantiomeric excess: 97% *ee* (CHIRALPAK IA, hexane/*i*-PrOH =98.5/1.5, detector: 254 nm, T = 30 ℃, flow rate: 1 mL/min), t_1(major) = 9.77 min, t_2(minor) =12.54 min.

9-(Tert-butyl) 3-ethyl 6-methyl (*S*)-4-phenyl-1,4-dihydro-9H-carbazole-3,6,9-tricarboxylate (3f)

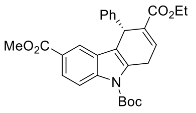

Step 1: 24 h; Step 2: 12 h (Silica gel 400 mg); Total yield: 23.8 mg (50%); **^{1}H NMR (400 MHz, $CDCl_3$)** δ 8.12 (d, *J* = 8.8 Hz, 1H), 7.99 (s, 1H), 7.89 (d, *J* = 8.8 Hz, 1H), 7.38 – 7.30 (m, 2H), 7.25 – 7.20 (m, 3H), 7.16 – 7.07 (m, 1H), 5.23 (t, *J* = 5.8 Hz, 1H), 4.19 – 4.02 (m, 4H), 3.88 (s, 3H), 1.71 (s, 9H), 1.22 (t, *J* = 7.1 Hz, 3H). **^{13}C NMR (100 MHz, $CDCl_3$)** δ 167.51, 166.27, 150.36, 142.85, 139.11, 134.59, 132.65, 132.22, 128.82, 128.43, 128.04, 126.84, 125.39, 124.70, 120.97, 118.67, 115.23, 84.81, 60.81, 52.11, 40.32, 28.58, 28.42, 14.22. **ESI-MS: calculated [$C_{28}H_{29}NO_6$ + H]$^+$: 476.2068, found:476.2068.** $[\alpha]^{20}_{D}$= -49.2 (c = 0.96, CH_2Cl_2). The product was analyzed by HPLC to determine the enantiomeric excess: 92% *ee* (CHIRALPAK IA, hexane/*i*-PrOH =90/10, detector: 254 nm, T = 30 ℃, flow rate: 1 mL/min), t_1(minor) =5.61 min, t_2(major) =6.62 min.

9-(Tert-butyl) 3-ethyl (*S*)-7-methyl-4-phenyl-1,4-dihydro-9H-carbazole-3,9-dicarboxylate (3g)

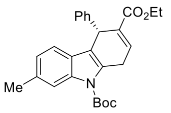

Step 1: 24 h; Step 2: 0.5 h (Silica gel 200 mg); Total yield: 37.3 mg (86%); **^{1}H NMR (400 MHz, $CDCl_3$)** δ 7.94 (s, 1H), 7.33 – 7.28 (m, 2H), 7.24 – 7.16 (m, 3H), 7.14 – 7.05 (m, 2H), 6.92 – 6.86 (m, 1H), 5.16 (t, *J* = 6.0 Hz, 1H), 4.20 – 3.97 (m, 4H), 2.39 (s, 3H), 1.70 (s, 9H), 1.21 (t, *J* = 7.1 Hz, 3H). **^{13}C NMR (100 MHz, $CDCl_3$)** δ 166.47, 150.80, 143.22, 136.74, 134.95, 133.81, 132.60, 129.91, 128.94, 128.26, 126.59, 125.98, 124.07, 118.48, 118.11, 115.92, 83.90, 60.68, 40.55, 28.65, 28.44, 22.10, 14.22. **ESI-MS: calculated [$C_{27}H_{29}NO_4$ + H]$^+$: 432.2169, found: 432.2170.** $[\alpha]^{20}_{D}$= 15.0 (c = 1.02, CH_2Cl_2). The product was analyzed by HPLC to determine the enantiomeric excess: 95% *ee* (CHIRALPAK IA, hexane/*i*-PrOH =97/3, detector: 254 nm, T = 30 ℃, flow rate: 1 mL/min), t_1(minor) = 5.48min, t_2(major) =8.14 min.

9-(Tert-butyl) 6-ethyl 2-methyl (*S*)-5-phenyl-5,8-dihydro-9H-carbazole-2,6,9-tricarboxylate (3h)

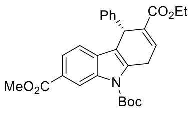

Step 1: 24 h; Step 2: 6 h (Silica gel 400 mg); Total yield: 24.4 mg (51%); **^{1}H NMR (400 MHz, $CDCl_3$)** δ 8.81 (s, 1H), 7.77 (dd, *J* = 8.2, 1.2 Hz, 1H), 7.34 – 7.29 (m, 2H), 7.27 – 7.19 (m, 4H), 7.15 – 7.07 (m, 1H), 5.20 (t, *J* = 5.8 Hz, 1H), 4.19 – 4.06 (m, 4H), 3.89 (s, 3H), 1.74 (s, 9H), 1.22 (t, *J* = 7.1 Hz, 3H). **^{13}C NMR (100 MHz, $CDCl_3$)** δ 167.84, 166.27, 150.33, 142.80, 135.68, 134.47, 134.28, 132.50, 131.86, 128.90, 128.39, 126.83, 125.51, 124.06, 118.50, 118.21, 117.53, 84.80, 60.82, 52.16, 40.35, 28.56, 28.38, 14.21. **ESI-MS: calculated [$C_{28}H_{29}NO_6$ + H]$^+$: 476.2068, found:476.2083.** $[\alpha]^{20}_{D}$= 15.5 (c = 0.51, CH_2Cl_2). The product was analyzed by HPLC to determine the enantiomeric excess: 86% *ee* (CHIRALPAK IA, hexane/*i*-PrOH =95/5, detector: 254 nm, T = 30 ℃, flow rate: 1 mL/min), t_1(minor) = 7.49 min, t_2(major) =8.38 min.

9-(Tert-butyl) 3-ethyl (*S*)-7-bromo-4-phenyl-1,4-dihydro-9H-carbazole-3,9-dicarboxylate (3i)

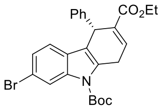

Step 1: 24 h; Step 2: 6 h (Silica gel 200 mg); Total yield: 30.3 mg (61%); **^{1}H NMR (400 MHz, $CDCl_3$)** δ 8.29 (s, 1H), 7.32 – 7.27 (m, 2H), 7.24 – 7.15 (m, 4H), 7.15 – 7.09 (m, 1H), 7.09 – 7.04 (m, 1H), 5.14 (t, *J* = 5.8 Hz, 1H), 4.20 – 3.97 (m, 4H), 1.71 (s, 9H), 1.21 (t, *J* = 7.1 Hz, 3H). **^{13}C NMR (100 MHz, $CDCl_3$)** δ 166.29, 150.29, 142.84, 137.00, 134.58, 132.48, 131.37, 128.88, 128.37, 127.06, 126.81, 125.94, 119.93, 118.81, 117.95, 117.68, 84.72, 60.78, 40.38, 28.51, 28.39, 14.22. **ESI-MS: calculated [$C_{26}H_{26}BrNO_4$ + H]$^+$: 496.1118, found:496.1121.** $[\alpha]^{20}_{D}$= -24.8 (c = 0.97, CH_2Cl_2). The product was analyzed by HPLC to determine the enantiomeric excess: 91% *ee* (CHIRALPAK IA, hexane/*i*-PrOH =97/3, detector: 254 nm, T = 30 ℃, flow rate: 1 mL/min), t_1(minor) = 5.87 min, t_2(major) =7.63 min.

9-(Tert-butyl) 3-ethyl (*S*)-8-methyl-4-phenyl-1,4-dihydro-9H-carbazole-3,9-dicarboxy-late (3j)

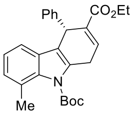

Step 1: 18 h; Step 2: 6 h (Silica gel 400 mg); Total yield: 23.9 mg (55%); **^{1}H NMR (400 MHz, $CDCl_3$)** δ 7.33 – 7.28 (m, 2H), 7.24 – 7.17 (m, 3H), 7.13 – 7.06 (m, 2H), 7.00 – 6.95 (m, 2H), 5.17 (t, *J* = 5.9 Hz, 1H), 4.18 – 4.04 (m, 2H), 4.00 – 3.78 (m, 2H), 2.45 (s, 3H), 1.68 (s, 9H), 1.21 (t, *J* = 7.1 Hz, 3H). **^{13}C NMR (100 MHz, $CDCl_3$)** δ 166.45, 150.35, 143.09, 135.84, 134.33, 133.16, 130.71, 129.23, 129.01, 128.26, 127.06, 126.60, 124.68, 122.90, 117.27, 116.65, 84.01, 77.48, 77.16, 76.84, 60.73, 40.70, 28.25, 27.97, 21.36, 14.22. **ESI-MS: calculated [$C_{27}H_{29}NO_4$ + Na]$^+$:454.1989, found: 454.1993.** $[\alpha]^{20}_{D}$= 21.5 (c = 0.51, CH_2Cl_2). The product was analyzed by HPLC to determine the enantiomeric excess: 90% *ee* (CHIRALPAK IA, hexane/*i*-PrOH =97/3, detector: 254 nm, T = 30 ℃, flow rate: 1 mL/min), t_1(major) =7.25min , t_2(minor) = 7.98 min.

9-(Tert-butyl) 3-ethyl (*S*)-4-(p-tolyl)-1,4-dihydro-9H-carbazole-3,9-dicarboxylate (3k)

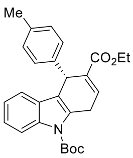

Step 1: 24 h; Step 2: 3 h (Silica gel 200 mg); Total yield: 33.2 mg (77%); **^{1}H NMR (400 MHz, $CDCl_3$)** δ 8.07 (d, *J* = 8.3 Hz, 1H), 7.28 – 7.24 (m, 1H), 7.23 – 7.15 (m, 4H), 7.09 – 7.04 (m, 1H), 7.01 (d, *J* = 7.9 Hz, 2H), 5.15 (t, *J* = 5.8 Hz, 1H), 4.20 – 4.00 (m, 4H), 2.24 (s, 3H), 1.70 (s, 9H), 1.23 (t, *J* = 7.1 Hz, 3H). **^{13}C NMR (100**

MHz, $CDCl_3$) δ 166.52, 150.78, 140.13, 136.28, 136.08, 134.70, 132.76, 130.76, 129.02, 128.76, 128.30, 123.90, 122.74, 118.94, 118.34, 115.52, 84.03, 60.71, 40.04, 28.59, 28.47, 21.19, 14.25. **ESI-MS: calculated [$C_{27}H_{29}NO_4$ + H]$^+$: 432.2169, found: 432.21625.** $[\alpha]^{20}_D$= 11.7 (c = 0.38, CH_2Cl_2). The product was analyzed by HPLC to determine the enantiomeric excess: 94% *ee* (CHIRALPAK IA, hexane/*i*-PrOH =97/3, detector: 254 nm, T = 30 ℃, flow rate: 1 mL/min), t_1(minor) = 5.47 min, t_2(major) =6.64 min.

9-(Tert-butyl) 3-ethyl (*S*)-4-(4-fluorophenyl)-1,4-dihydro-9H-carbazole-3,9-dicarboxy-late (3l)

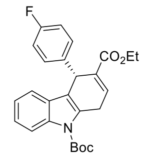

Step 1: 12 h; Step 2: 2 h (Silica gel 200 mg); Total yield: 40.1 mg (92%); **^{1}H NMR (400 MHz, $CDCl_3$)** δ 8.08 (d, *J* = 8.3 Hz, 1H), 7.32 – 7.26 (m, 2H), 7.24 – 7.16 (m, 3H), 7.11 – 7.05 (m, 1H), 6.93 – 6.86 (m, 2H), 5.18 (t, *J* = 5.9 Hz, 1H), 4.24 – 3.94 (m, 4H), 1.71 (s, 9H), 1.23 (t, *J* = 7.1 Hz, 3H). **^{13}C NMR (100 MHz, $CDCl_3$)** δ 166.32, 161.60 (*J* = 244.5 Hz), 150.71, 138.91 (*J* = 3.0 Hz), 136.26, 134.99, 132.44, 130.91, 130.41 (*J* = 8.0 Hz), 128.07, 124.04, 122.78, 118.79, 117.84, 115.60, 115.11 (*J* = 21.4 Hz), 84.17, 60.78, 39.72, 28.52, 28.45, 14.25. **^{19}F NMR (376 MHz, $CDCl_3$)** δ -116.42. **ESI-MS: calculated [$C_{26}H_{26}FNO_4$ + Na]$^+$: 458.1738, found: 458.1746.** $[\alpha]^{20}_D$= 27.6 (c = 1.00, CH_2Cl_2). The product was analyzed by HPLC to determine the enantiomeric excess: 94% *ee* (CHIRALPAK IA, hexane/*i*-PrOH =97/3, detector: 254 nm, T = 30 ℃, flow rate: 1 mL/min), t_1(minor) = 5.58 min, t_2(major) =7.38 min.

9-(Tert-butyl) 3-ethyl (*S*)-4-(4-chlorophenyl)-1,4-dihydro-9H-carbazole-3,9-dicarboxyl-ate (3m)

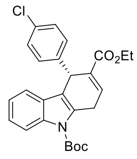

Step 1: 12 h; Step 2: 2 h (Silica gel 200 mg); Total yield: 41.3 mg (91%); **^{1}H NMR (400 MHz, $CDCl_3$)** δ 8.08 (d, *J* = 8.3 Hz, 1H), 7.28 – 7.16 (m, 7H), 7.11 – 7.03 (m, 1H), 5.16 (t, *J* = 5.9 Hz, 1H), 4.23 – 4.00 (m, 4H), 1.71 (s, 9H), 1.23 (d, *J* = 7.1 Hz, 3H). **^{13}C NMR (100 MHz, $CDCl_3$)** δ 166.22, 150.69, 141.77, 136.25, 135.27, 132.31, 132.17, 130.98, 130.32, 128.47, 127.99, 124.10, 122.82, 118.74, 117.59, 115.61, 84.21, 60.83, 39.88, 28.53, 28.45, 14.26. **ESI-MS: calculated [$C_{26}H_{26}ClNO_4$ + Na]$^+$:474.1443, found:474.1443.** $[\alpha]^{20}_D$= 13.5 (c = 1.01, CH_2Cl_2). The product was analyzed by HPLC to determine the enantiomeric excess: 94% *ee* (CHIRALPAK IA, hexane/*i*-PrOH =97/3, detector: 254 nm, T = 30 ℃, flow rate: 1 mL/min), t_1(minor) = 6.06 min, t_2(major) =7.57 min.

9-(Tert-butyl) 3-ethyl (*S*)-4-(4-bromophenyl)-1,4-dihydro-9H-carbazole-3,9-dicarboxy-late (3n)

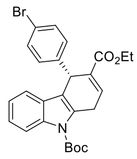

Step 1: 18 h; Step 2: 2 h (Silica gel 200 mg); Total yield: 34.6 mg (70%); **^{1}H NMR (400 MHz, $CDCl_3$)** δ 8.08 (d, *J* = 8.3 Hz, 1H), 7.36 – 7.30 (m, 2H), 7.26 – 7.15 (m, 5H), 7.11 – 7.03 (m, 1H), 5.16 (t, *J* = 5.9 Hz, 1H), 4.21 – 4.09 (m, 2H), 4.09 – 4.02 (m, 2H), 1.71 (s, 9H), 1.24 (t, *J* = 7.1 Hz, 3H). **^{13}C NMR (100 MHz, $CDCl_3$)** δ 166.21, 150.69, 142.31, 136.25, 135.32, 132.10, 131.42, 130.99, 130.72, 127.98, 124.12, 122.84, 120.48, 118.74, 117.52, 115.62, 84.23, 60.85, 39.96, 28.54, 28.46, 14.27. **ESI-MS:**

calculated [$C_{26}H_{26}BrNO_4$ + H]$^+$: 496.1118, found: 496.1111. [α]$^{20}_{D}$= 9.0 (c = 1.01, CH_2Cl_2). The product was analyzed by HPLC to determine the enantiomeric excess: 93% *ee* (CHIRALPAK IA, hexane/*i*-PrOH =97/3, detector: 254 nm, T = 30 ℃, flow rate: 1 mL/min), t_1(minor) = 5.73 min, t_2(major) =7.02 min.

9-(Tert-butyl) 3-ethyl (*S*)-4-(4-(trifluoromethyl)phenyl)-1,4-dihydro-9H-carbazole-3,9-dicarboxylate (3o)

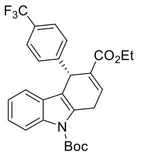

Step 1: 10 h; Step 2: 2 h (Silica gel 200 mg); Total yield: 38.7 mg (80%); **^{1}H NMR (400 MHz, $CDCl_3$)** δ 8.09 (d, *J* = 8.3 Hz, 1H), 7.52 – 7.40 (m, 4H), 7.32 – 7.27 (m, 1H), 7.24 – 7.16 (m, 2H), 7.13 – 7.05 (m, 1H), 5.26 (t, *J* = 5.9 Hz, 1H), 4.21 – 3.98 (m, 4H), 1.71 (s, 9H), 1.23 (t, *J* = 7.1 Hz, 3H). **^{13}C NMR (100 MHz, $CDCl_3$)** δ 166.07, 150.67, 147.35, 136.27, 135.79, 131.88, 131.14, 129.29, 128.88 (*J* = 32.2 Hz), 127.88, 125.21 (*J* = 3.7 Hz), 124.30 (*J* = 270.3 Hz), 124.19, 122.88, 118.62, 117.30, 115.68, 84.32, 60.91, 40.36, 28.59, 28.45, 14.23. **ESI-MS: calculated [$C_{27}H_{26}F_3NO_4$ + H]$^+$: 486.1887, found: 486.1896.** [α]$^{20}_{D}$= 18.8 (c = 1.00, CH_2Cl_2). The product was analyzed by HPLC to determine the enantiomeric excess: 94% *ee* (CHIRALPAK IA, hexane/*i*-PrOH =97/3, detector: 254 nm, T = 30 ℃, flow rate: 1 mL/min), t_1(minor) = 5.67min, t_2(major) =6.86 min.

9-(Tert-butyl) 3-ethyl (*S*)-4-(4-(methoxycarbonyl)phenyl)-1,4-dihydro-9H-carbazole-3,9-dicarboxylate (3p)

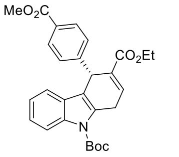

Step 1: 8 h; Step 2: 2 h (Silica gel 200 mg); Total yield: 40.9 mg (86%); **^{1}H NMR (400 MHz, $CDCl_3$)** δ 8.08 (d, *J* = 8.5 Hz, 1H), 7.90 (d, *J* = 8.3 Hz, 2H), 7.41 (d, *J* = 8.3 Hz, 2H), 7.28 (t, *J* = 3.5 Hz, 1H), 7.22 – 7.17 (m, 2H), 7.08 – 7.03 (m, 1H), 5.24 (t, *J* = 5.9 Hz, 1H), 4.19 – 4.03 (m, 4H), 3.85 (s, 3H), 1.71 (s, 9H), 1.19 (d, *J* = 4.9 Hz, 3H). **^{13}C NMR (100 MHz, $CDCl_3$)** δ 167.09, 166.15, 150.68, 148.58, 136.24, 135.68, 131.87, 131.03, 129.72, 129.07, 128.59, 127.96, 124.11, 122.83, 118.68, 117.33, 115.61, 84.26, 60.86, 52.10, 40.56, 28.58, 28.45, 14.23. **ESI-MS: calculated [$C_{28}H_{29}NO_6$ + H]$^+$: 476.2068, found: 476.2063.** [α]$^{20}_{D}$= 15.2 (c = 1.02, CH_2Cl_2). The product was analyzed by HPLC to determine the enantiomeric excess: 94% *ee* (CHIRALPAK IA, hexane/*i*-PrOH =97/3, detector: 254 nm, T = 30 ℃, flow rate: 1 mL/min), t_1(minor) =10.97 min, t_2(major) =15.22 min.

9-(Tert-butyl) 3-ethyl (*S*)-4-(4-cyanophenyl)-1,4-dihydro-9H-carbazole-3,9-dicarboxylate (3q)

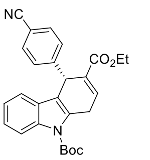

Step 1: 12 h; Step 2: 2 h (Silica gel 200 mg); Total yield: 38.4 mg (87%); **^{1}H NMR (400 MHz, $CDCl_3$)** δ 8.09 (d, *J* = 8.4 Hz, 1H), 7.52 (d, *J* = 8.3 Hz, 2H), 7.45 (d, *J* = 8.3 Hz, 2H), 7.31 (t, *J* = 3.4 Hz, 1H), 7.25 – 7.19 (m, 1H), 7.16 – 7.12 (m, 1H), 7.11 – 7.05 (m, 1H), 5.24 (t, *J* = 5.9 Hz, 1H), 4.20 – 3.99 (m, 4H), 1.71 (s, 9H), 1.23 (t, *J* = 7.1 Hz, 3H). **^{13}C NMR (100 MHz, $CDCl_3$)** δ 165.89, 150.60, 148.84, 136.21, 132.23, 131.41, 131.23, 129.79, 127.69, 124.30, 124.08, 122.93, 119.04, 118.42, 116.79, 115.72, 110.54, 84.43, 60.98, 40.62, 28.56, 28.43, 14.24. **ESI-MS: calculated**

$[C_{27}H_{26}N_2O_4 + H]^+$**: 443.1965, found: 443.1966.** $[\alpha]^{20}_D$= 8.7 (c = 1.01, CH_2Cl_2). The product was analyzed by HPLC to determine the enantiomeric excess: 94% *ee* (CHIRALPAK IA, hexane/*i*-PrOH =90/10, detector: 254 nm, T = 30 ℃, flow rate: 1 mL/min), t_1(minor) = 7.03 min, t_2(major) =8.21 min.

9-(Tert-butyl) 3-ethyl (*S*)-4-(m-tolyl)-1,4-dihydro-9H-carbazole-3,9-dicarboxylate (3r)

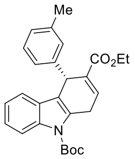

Step 1: 20 h; Step 2: 2 h (Silica gel 200 mg); Total yield: 25.0 mg (58%); **^{1}H NMR (400 MHz, $CDCl_3$)** δ 8.07 (d, *J* = 8.3 Hz, 1H), 7.29 – 7.15 (m, 4H), 7.13 – 7.03 (m, 3H), 6.92 (d, *J* = 7.2 Hz, 1H), 5.15 (t, *J* = 5.8 Hz, 1H), 4.19 – 4.01 (m, 4H), 2.26 (s, 3H), 1.71 (s, 9H), 1.22 (t, *J* = 7.1 Hz, 3H). **^{13}C NMR (100 MHz, $CDCl_3$)** δ 166.53, 150.80, 143.02, 137.73, 136.28, 134.79, 132.73, 130.80, 129.56, 128.36, 128.07, 127.47, 126.20, 123.89, 122.75, 118.99, 118.24, 115.51, 84.05, 60.70, 40.46, 28.59, 28.48, 21.62, 14.22. **ESI-MS: calculated $[C_{27}H_{29}NO_4 + Na]^+$: 454.1989, found: 454.2002.** $[\alpha]^{20}_D$= 27.5 (c = 0.94, CH_2Cl_2). The product was analyzed by HPLC to determine the enantiomeric excess: 92% *ee* (CHIRALPAK IA, hexane/*i*-PrOH =98.5/1.5, detector: 254 nm, T = 30 ℃, flow rate: 1 mL/min), t_1(minor) = 6.35 min, t_2(major) =10.35 min.

9-(Tert-butyl) 3-ethyl (*S*)-4-(3-chlorophenyl)-1,4-dihydro-9H-carbazole-3,9-dicarboxy-late (3s)

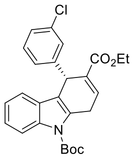

Step 1: 12 h; Step 2: 2 h (Silica gel 100 mg); Total yield: 23.5 mg (52%); **^{1}H NMR (400 MHz, $CDCl_3$)** δ 8.09 (d, *J* = 8.3 Hz, 1H), 7.29 – 7.18 (m, 5H), 7.18 – 7.06 (m, 3H), 5.16 (t, *J* = 5.8 Hz, 1H), 4.21 – 4.02 (m, 4H), 1.71 (s, 9H), 1.24 (t, *J* = 7.1 Hz, 3H). **^{13}C NMR (100 MHz, $CDCl_3$)** δ 166.18, 150.69, 145.33, 136.29, 135.59, 134.09, 131.99, 131.07, 129.50, 128.99, 128.00, 127.42, 126.96, 124.11, 122.85, 118.73, 117.39, 115.63, 84.25, 60.87, 40.26, 28.57, 28.47, 14.24. **ESI-MS: calculated $[C_{26}H_{26}ClNO_4 + H]^+$: 452.1623, found: 452.1616.** $[\alpha]^{20}_D$= 17.4 (c = 1.03, CH_2Cl_2). The product was analyzed by HPLC to determine the enantiomeric excess: 92% *ee* (CHIRALPAK IA, hexane/*i*-PrOH =97/3, detector: 254 nm, T = 30 ℃, flow rate: 1 mL/min), t_1(minor) = 5.39min, t_2(major) =7.82 min.

9-(Tert-butyl) 3-ethyl (*S*)-4-(3-(methoxycarbonyl)phenyl)-1,4-dihydro-9H-carbazole-3,9-dicarboxylate (3t)

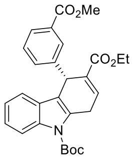

Step 1: 12 h; Step 2: 2 h (Silica gel 100 mg); Total yield: 29.4 mg (62%); **^{1}H NMR (400 MHz, $CDCl_3$)** δ 8.10 – 8.05 (m, 1H), 8.00 (t, *J* = 1.6 Hz, 1H), 7.85 – 7.78 (m, 1H), 7.57 – 7.51 (m, 1H), 7.34 – 7.26 (m, 2H), 7.22 – 7.15 (m, 2H), 7.09 – 7.02 (m, 1H), 5.26 – 5.22 (m, 1H), 4.19 – 4.05 (m, 4H), 3.87 (s, 3H), 1.71 (s, 9H), 1.22 (t, *J* = 7.1 Hz, 3H). **^{13}C NMR (100 MHz, $CDCl_3$)** δ 167.22, 166.21, 150.69, 143.76, 136.27, 135.66, 133.75, 132.01, 131.08, 130.19, 130.10, 128.36, 128.05, 128.01, 124.05, 122.80, 118.71, 117.46, 115.60, 84.21, 60.82, 52.16, 40.42, 28.58, 28.45, 14.18. **ESI-MS: calculated $[C_{28}H_{29}NO_6 + Na]^+$: 498.1887, found: 498.1890.** $[\alpha]^{20}_D$= 14.3 (c = 1.01, CH_2Cl_2). The product was analyzed by HPLC to determine the enantiomeric excess: 94% *ee*

(CHIRALPAK IA, hexane/*i*-PrOH =97/3, detector: 254 nm, T = 30 ℃, flow rate: 1 mL/min), t_1(minor) = 8.06 min, t_2(major) =13.97 min.

9-(Tert-butyl) 3-ethyl (*S*)-4-(naphthalen-2-yl)-1,4-dihydro-9H-carbazole-3,9-dicarboxylate (3u)

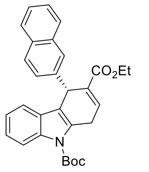

Step 1: 36 h; Step 2: 3 h (Silica gel 200 mg); Total yield: 41.0 mg (88%); **^{1}H NMR (400 MHz, $CDCl_3$)** δ 8.06 (d, *J* = 8.3 Hz, 1H), 7.85 (s, 1H), 7.78 (d, *J* = 7.9 Hz, 1H), 7.72 (d, *J* = 7.8 Hz, 1H), 7.67 (d, *J* = 8.5 Hz, 1H), 7.44 – 7.32 (m, 3H), 7.29 – 7.22 (m, 2H), 7.15 (t, *J* = 7.7 Hz, 1H), 7.01 (t, *J* = 7.5 Hz, 1H), 5.37 (t, *J* = 6.0 Hz, 1H), 4.21 – 3.98 (m, 4H), 1.71 (s, 9H), 1.19 (t, *J* = 7.1 Hz, 3H). **^{13}C NMR (100 MHz, $CDCl_3$)** δ 166.42, 150.79, 140.58, 136.26, 135.04, 133.48, 132.53, 132.50, 130.96, 128.25, 128.01, 127.93, 127.81, 127.69, 127.13, 125.90, 125.55, 123.96, 122.77, 118.95, 117.97, 115.52, 84.12, 60.74, 40.64, 28.65, 28.47, 14.23. **ESI-MS: calculated [$C_{30}H_{29}NO_4$ + Na]$^+$: 490.1989, found: 490.1994.** $[\alpha]^{20}_D$= 48.3 (c = 0.99, CH_2Cl_2). The product was analyzed by HPLC to determine the enantiomeric excess: 93% *ee* (CHIRALPAK IA, hexane/*i*-PrOH =97/3, detector: 254 nm, T = 30 ℃, flow rate: 1 mL/min), t_1(minor) = 6.66 min, t_2(major) =10.38 min.

9-(Tert-butyl) 3-ethyl (*S*)-4-((benzyloxy)methyl)-1,4-dihydro-9H-carbazole-3,9-dicarboxylate (3v)

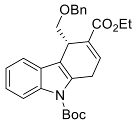

Step 1: 24 h; Step 2: 2 h (Silica gel 200 mg); Total yield: 30.2 mg (65%); **^{1}H NMR (400 MHz, $CDCl_3$)** δ 8.14 (d, *J* = 8.2 Hz, 1H), 7.55 (d, *J* = 7.5 Hz, 1H), 7.28 – 7.17 (m, 6H), 7.14 – 7.07 (m, 2H), 4.38 – 4.31 (m, 3H), 4.23 (q, *J* = 7.1 Hz, 2H), 3.99 – 3.85 (m, 2H), 3.84 – 3.77 (m, 2H), 1.69 (s, 9H), 1.31 (t, *J* = 7.1 Hz, 3H). **^{13}C NMR (100 MHz, $CDCl_3$)** δ 166.87, 150.65, 138.59, 137.69, 136.34, 132.35, 130.10, 128.44, 128.20, 123.86, 122.68, 118.85, 116.39, 115.67, 83.92, 73.17, 72.65, 60.78, 35.15, 28.77, 28.44, 14.39. **ESI-MS: calculated [$C_{28}H_{31}NO_5$ + Na]$^+$: 484.2094, found: 484.2103.** $[\alpha]^{20}_D$= -34.2 (c = 1.15, CH_2Cl_2). The product was analyzed by HPLC to determine the enantiomeric excess: 92% *ee* (CHIRALPAK IA, hexane/*i*-PrOH =97/3, detector: 254 nm, T = 30 ℃, flow rate: 1 mL/min), t_1(major) = 8.15 min, t_2(minor) =9.94 min.

9-(Tert-butyl) 3-ethyl (*S*)-4-propyl-1,4-dihydro-9H-carbazole-3,9-dicarboxylate (3w)

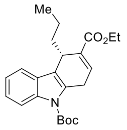

Step 1: 36 h; Step 2: 4 h (Silica gel 400 mg); Total yield: 28.9 mg (75%); **^{1}H NMR (400 MHz, $CDCl_3$)** δ 8.14 (d, *J* = 7.8 Hz, 1H), 7.54 (d, *J* = 7.1 Hz, 1H), 7.32 – 7.23 (m, 3H), 7.20 (s, 1H), 4.36 – 4.20 (m, 3H), 3.97 – 3.72 (m, 2H), 1.95 – 1.80 (m, 2H), 1.69 (s, 9H), 1.36 (t, *J* = 7.0 Hz, 3H), 1.17 – 1.04 (m, 1H), 1.00 – 0.87 (m, 1H), 0.74 (t, *J* = 7.2 Hz, 3H). **^{13}C NMR (100 MHz, $CDCl_3$)** δ 166.95, 150.69, 136.55, 136.36, 132.27, 131.83, 128.45, 123.80, 122.67, 118.63, 118.10, 115.70, 83.89, 60.72, 36.08, 33.27, 28.74, 28.45, 17.93, 14.45, 14.35. **ESI-MS: calculated [$C_{23}H_{29}NO_4$ + H]$^+$: 384.2169, found: 384.2161.** $[\alpha]^{20}_D$= -71.1 (c = 0.39, CH_2Cl_2). The product was analyzed by HPLC to determine the enantiomeric excess: 90% *ee* (CHIRALPAK IA, hexane/*i*-PrOH =99/1, detector: 254 nm, T = 30 ℃, flow rate: 1 mL/min), t_1(minor) = 5.54 min, t_2(major) =7.23 min.

9-(Tert-butyl) 3-ethyl (*S*)-4-(3-ethoxy-3-oxopropyl)-1,4-dihydro-9H-carbazole-3,9-dicarboxylate (3x)

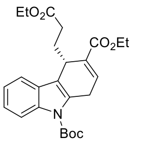

Step 1: 36 h; Step 2: 4 h (Silica gel 400 mg); Total yield: 30.8 mg (70%); **^{1}H NMR (400 MHz, CDCl$_3$)** δ 8.14 (d, *J* = 8.0 Hz, 1H), 7.55 (d, *J* = 7.2 Hz, 1H), 7.35 – 7.18 (m, 3H), 4.41 – 4.34 (m, 1H), 4.32 – 4.22 (m, 2H), 3.98 – 3.88 (m, 3H), 3.87 – 3.77 (m, 1H), 2.37 – 2.27 (m, 2H), 2.12 – 2.01 (m, 1H), 1.98 – 1.88 (m, 1H), 1.70 (s, 9H), 1.37 (t, *J* = 7.1 Hz, 3H), 1.12 (t, *J* = 7.1 Hz, 3H). **^{13}C NMR (100 MHz, CDCl$_3$)** δ 173.65, 166.46, 150.57, 137.70, 136.40, 132.28, 130.99, 127.99, 124.06, 122.86, 118.53, 116.56, 115.75, 84.08, 60.88, 60.30, 32.42, 29.58, 28.71, 28.43, 28.08, 14.42, 14.19. **ESI-MS: calculated [$C_{25}H_{31}NO_6$ + H]$^+$: 442.2224, found: 442.2208.** $[\alpha]^{20}_{D}$= -47.5 (c = 0.51, CH_2Cl_2). The product was analyzed by HPLC to determine the enantiomeric excess: 81% *ee* (CHIRALPAK AS-H, hexane/*i*-PrOH =97/3, detector: 254 nm, T = 30 ℃, flow rate: 1 mL/min), t_1(major) =5.74 min, t_2(minor) =6.62 min.

3-Benzyl 9-(tert-butyl) (*S*)-4-phenyl-1,4-dihydro-9H-carbazole-3,9-dicarboxylate (3y)

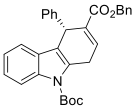

Step 1: 18 h; Step 2: 1.5 h (Silica gel 200 mg); Total yield: 32.9 mg (69%); **^{1}H NMR (400 MHz, CDCl$_3$)** δ 8.07 (d, *J* = 8.3 Hz, 1H), 7.34 – 7.27 (m, 6H), 7.26 – 7.15 (m, 6H), 7.14 – 7.09 (m, 1H), 7.08 – 7.02 (m, 1H), 5.20 (t, *J* = 5.9 Hz, 1H), 5.10 (dd, *J* = 39.1, 12.4 Hz, 2H), 4.15 – 3.95 (m, 2H), 1.69 (s, 9H). **^{13}C NMR (100 MHz, CDCl$_3$)** δ 166.25, 150.71, 143.00, 136.26, 136.00, 135.59, 132.38, 130.69, 128.98, 128.60, 128.36, 128.26, 128.23, 128.19, 126.68, 123.95, 122.75, 118.91, 118.08, 115.53, 84.07, 66.57, 40.53, 28.64, 28.45. **ESI-MS: calculated [$C_{31}H_{29}NO_4$ + Na]$^+$: 502.1989, found: 502.1987.** $[\alpha]^{20}_{D}$= 24.5 (c = 0.97, CH_2Cl_2). The product was analyzed by HPLC to determine the enantiomeric excess: 93% *ee* (CHIRALPAK IA, hexane/*i*-PrOH = 97/3, detector: 254 nm, T = 30 ℃, flow rate: 1 mL/min), t_1(minor) = 8.10 min, t_2(major) =11.15 min.

Di-tert-butyl (*S*)-4-phenyl-1,4-dihydro-9H-carbazole-3,9-dicarboxylate (3z)

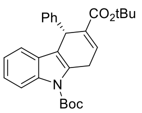

Step 1: 36 h; Step 2: 1.5 h (Silica gel 200 mg); Total yield: 33.7 mg (76%); **^{1}H NMR (400 MHz, CDCl$_3$)** δ 8.07 (d, *J* = 8.3 Hz, 1H), 7.34 – 7.27 (m, 3H), 7.26 – 7.17 (m, 3H), 7.17 – 7.10 (m, 2H), 7.09 – 7.04 (m, 1H), 5.12 (t, *J* = 6.0 Hz, 1H), 4.12 – 3.93 (m, 2H), 1.70 (s, 9H), 1.36 (s, 9H). **^{13}C NMR (100 MHz, CDCl$_3$)** δ 165.89, 150.76, 143.28, 136.29, 133.95, 133.89, 130.91, 129.02, 128.35, 128.18, 126.56, 123.85, 122.69, 118.95, 118.12, 115.52, 84.02, 80.92, 40.72, 28.53, 28.46, 28.10. **ESI-MS: calculated [$C_{28}H_{31}NO_4$ + H]$^+$: 446.2326, found: 446.2318.** $[\alpha]^{20}_{D}$= 26.9 (c = 2.0, CH_2Cl_2). The product was analyzed by HPLC to determine the enantiomeric excess: 92% *ee* (CHIRALPAK IA, hexane/*i*-PrOH =98.5/1.5, detector: 254 nm, T = 30 ℃, flow rate: 1 mL/min), t_1(minor) = 5.32 min, t_2(major) = 6.60 min.

Ethyl (*R*)-1-(4-bromophenyl)-1,4-dihydrodibenzo[b,d]thiophene-2-carboxylate (6a)

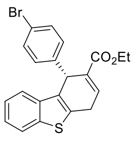

Step 1: 48 h; Step 2: 5 h (Silica gel 300 mg); Total yield: 24.4 mg (59%); **^{1}H NMR (400 MHz, $CDCl_3$)** δ 7.77 – 7.71 (m, 1H), 7.48 – 7.42 (m, 1H), 7.34 – 7.30 (m, 2H), 7.28 – 7.25 (m, 1H), 7.24 – 7.16 (m, 4H), 5.27 (t, *J* = 5.2 Hz, 1H), 4.26 – 4.06 (m, 2H), 4.02 – 3.89 (m, 1H), 3.87 – 3.74 (m, 1H), 1.27 (t, *J* = 7.1 Hz, 3H). **^{13}C NMR (100 MHz, $CDCl_3$)** δ 166.04, 141.73, 139.23, 137.89, 134.40, 133.13, 132.89, 131.50, 130.77, 130.44, 124.30, 124.28, 122.48, 121.78, 120.62, 60.97, 41.47, 28.07, 14.30. **ESI-MS: calculated [$C_{21}H_{17}BrN_2S$ + H]$^+$: 413.0205, found: 413.0213.** $[\alpha]^{20}_{D}$= -36.9 (c = 0.81, CH_2Cl_2). The product was analyzed by HPLC to determine the enantiomeric excess: 95% *ee* (CHIRALPAK IA, hexane/*i*-PrOH =90/10, detector: 254 nm, T = 30 ℃, flow rate: 1 mL/min), t_1(minor) = 7.53 min, t_2(major) =8.22min.

Ethyl (*R*)-1-(4-cyanophenyl)-1,4-dihydrodibenzo[b,d]thiophene-2-carboxylate (6b)

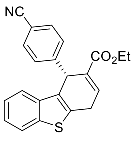

Step 1: 48 h; Step 2: 5 h (Silica gel 300 mg); Total yield: 25.7 mg (72%); **^{1}H NMR (400 MHz, $CDCl_3$)** δ 7.79 – 7.74 (m, 1H), 7.53 – 7.48 (m, 2H), 7.45 – 7.38 (m, 3H), 7.36 – 7.32 (m, 1H), 7.29 – 7.19 (m, 2H), 5.36 (t, *J* = 5.1 Hz, 1H), 4.25 – 4.08 (m, 2H), 4.03 – 3.94 (m, 1H), 3.91 – 3.78 (m, 1H), 1.26 (t, *J* = 7.1 Hz, 3H). **^{13}C NMR (100 MHz, $CDCl_3$)** δ 165.76, 148.20, 139.27, 137.64, 135.25, 133.35, 132.50, 132.30, 129.86, 129.69, 124.47, 124.45, 122.62, 121.49, 118.95, 110.68, 61.11, 42.09, 28.12, 14.29. **ESI-MS: calculated [$C_{22}H_{17}NO_2S$ + H]$^+$: 360.1053, found: 360.1055.** $[\alpha]^{20}_{D}$= -34.2 (c = 1.15, CH_2Cl_2). The product was analyzed by HPLC to determine the enantiomeric excess: 90% *ee* (CHIRALPAK IA, hexane/*i*-PrOH =90/10, detector: 254 nm, T = 30 ℃, flow rate: 1 mL/min), t_1(minor) = 15.32 min, t_2(major) = 18.83 min.

Ethyl (*R*)-8-bromo-1-(4-cyanophenyl)-1,4-dihydrodibenzo[b,d]thiophene-2-carboxylate (6c)

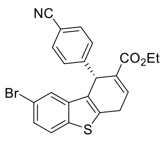

Step 1: 60 h; Step 2: 8 h (Silica gel 500 mg); Total yield: 23.7 mg (54%); **^{1}H NMR (400 MHz, $CDCl_3$)** δ 7.61 (d, *J* = 8.5 Hz, 1H), 7.56 – 7.50 (m, 3H), 7.41 (d, *J* = 8.3 Hz, 2H), 7.37 – 7.29 (m, 2H), 5.30 (t, *J* = 5.1 Hz, 1H), 4.30 – 4.08 (m, 2H), 4.04 – 3.91 (m, 1H), 3.90 – 3.77 (m, 1H), 1.26 (t, *J* = 7.1 Hz, 3H). **^{13}C NMR (100 MHz, $CDCl_3$)** δ 165.55, 139.30, 137.86, 135.38, 134.93, 132.45, 132.40, 129.72, 129.15, 127.52, 124.24, 123.91, 118.86, 118.56, 110.95, 61.19, 41.89, 28.09, 14.28. **ESI-MS: calculated [$C_{22}H_{16}BrNO_2S$ + H]$^+$: 438.0158, found: 438.0164.** $[\alpha]^{20}_{D}$= -100.3 (c = 0.74, CH_2Cl_2). The product was analyzed by HPLC to determine the enantiomeric excess: 91% *ee* (CHIRALPAK IA, hexane/*i*-PrOH =90/10, detector: 254 nm, T = 30 ℃, flow rate: 1 mL/min), t_1(minor) = 18.98 min, t_2(major) =21.56 min.

Ethyl (*R*)-8-chloro-1-(4-cyanophenyl)-1,4-dihydrodibenzo[b,d]thiophene-2-carboxylate (6d)

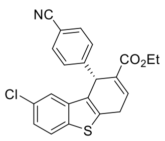

Step 1: 60 h; Step 2: 8 h (Silica gel 500 mg); Total yield: 24.2 mg (61%); **^{1}H NMR (400 MHz, $CDCl_3$)** δ 7.66 (d, *J* = 8.5 Hz, 1H), 7.53 (d, *J* = 8.2 Hz, 2H), 7.42 (d, *J* = 8.2 Hz, 2H), 7.35 (d, *J* = 1.9 Hz, 1H), 7.34 – 7.30 (m, 1H), 7.21 (dd,

J = 8.5, 1.9 Hz, 1H), 5.30 (t, J = 5.1 Hz, 1H), 4.25 – 4.09 (m, 2H), 4.04 – 3.93 (m, 1H), 3.90 – 3.77 (m, 1H), 1.26 (t, J = 7.1 Hz, 3H). **^{13}C NMR (100 MHz, $CDCl_3$)** δ 165.45, 147.57, 138.76, 137.24, 135.44, 134.83, 132.34, 132.28, 130.68, 129.63, 129.13, 124.80, 123.48, 121.08, 118.75, 110.83, 61.09, 41.81, 28.02, 14.18. **ESI-MS: calculated [$C_{22}H_{16}ClNO_2S$ + H]$^+$: 394.0663, found: 394.0667.** $[\alpha]^{20}_D$= -100.0 (c = 1.01, CH_2Cl_2). The product was analyzed by HPLC to determine the enantiomeric excess: 91% *ee* (CHIRALPAK IA, hexane/*i*-PrOH =90/10, detector: 254 nm, T = 30 ℃, flow rate: 1 mL/min), t_1(minor) = 16.12 min, t_2(major) = 19.62min.

Ethyl (*R*)-7-bromo-1-(4-cyanophenyl)-1,4-dihydrodibenzo[b,d]thiophene-2-carboxylate (6e)

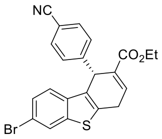

Step 1: 60 h; Step 2: 5 h (Silica gel 600 mg); Total yield: 22.5 mg (51%); **^{1}H NMR (400 MHz, $CDCl_3$)** δ 7.88 (d, J = 1.6 Hz, 1H), 7.51 (d, J = 8.2 Hz, 2H), 7.40 (d, J = 8.3 Hz, 2H), 7.34 – 7.28 (m, 2H), 7.23 (d, J = 8.5 Hz, 1H), 5.31 (t, J = 5.2 Hz, 1H), 4.27 – 4.07 (m, 2H), 4.03 – 3.89 (m, 1H), 3.89 – 3.75 (m, 1H), 1.26 (t, J = 7.1 Hz, 3H). **^{13}C NMR (100 MHz, $CDCl_3$)** δ 165.59, 147.80, 140.74, 136.42, 134.91, 134.03, 132.35, 129.78, 129.48, 127.87, 125.16, 122.53, 118.80, 118.30, 110.89, 61.16, 41.97, 28.01, 14.27. **ESI-MS: calculated [$C_{22}H_{16}NO_2S$ + H]$^+$: 438.0158, found: 438.0167.** $[\alpha]^{20}_D$= -11.8 (c = 0.49, CH_2Cl_2). The product was analyzed by HPLC to determine the enantiomeric excess: 94% *ee* (CHIRALPAK IA, hexane/*i*-PrOH =90/10, detector: 254 nm, T = 30 ℃, flow rate: 1 mL/min), t_1(minor) = 18.73 min, t_2(major) = 23.81min.

Ethyl (*R*)-8-bromo-1-(4-bromophenyl)-1,4-dihydrodibenzo[b,d]thiophene-2-carboxylate (6f)

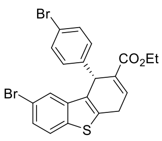

Step 1: 60 h; Step 2: 24 h (Silica gel 600 mg); Total yield: 31.9 mg (65%); **^{1}H NMR (400 MHz, $CDCl_3$)** δ 7.61 – 7.52 (m, 2H), 7.37 – 7.29 (m, 3H), 7.25 – 7.23 (m, 1H), 7.19 – 7.14 (m, 2H), 5.21 (t, J = 5.1 Hz, 1H), 4.26 – 4.07 (m, 2H), 4.00 – 3.89 (m, 1H), 3.85 – 3.73 (m, 1H), 1.27 (t, J = 7.1 Hz, 3H). **^{13}C NMR (101 MHz, $CDCl_3$)** δ 165.83, 141.24, 139.57, 137.86, 134.91, 134.07, 133.08, 131.68, 130.61, 129.96, 127.34, 124.52, 123.79, 120.90, 118.45, 61.05, 41.31, 28.06, 14.30. **ESI-MS: calculated [$C_{21}H_{16}Br_2O_2S$ + H]$^+$: 492.9290, found: 492.9296.** $[\alpha]^{20}_D$= -112.6 (c = 1.00, CH_2Cl_2). The product was analyzed by HPLC to determine the enantiomeric excess: 97% *ee* (CHIRALPAK AD-H, hexane/*i*-PrOH =85/15, detector: 254 nm, T = 30 ℃, flow rate: 1 mL/min), t_1(major) = 8.59 min, t_2(minor) = 9.49 min.

Ethyl (*R*)-8-bromo-1-(4-(trifluoromethyl)phenyl)-1,4-dihydrodibenzo[b,d]thiophene-2-carboxylate (6g)

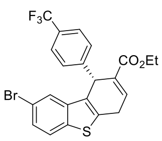

Step 1: 60 h; Step 2: 24 h (Silica gel 600 mg); Total yield: 33.7 mg (70%); **^{1}H NMR (400 MHz, $CDCl_3$)** δ 7.61 – 7.54 (m, 2H), 7.49 (d, J = 8.2 Hz, 2H), 7.41 (d, J = 8.2 Hz, 2H), 7.35 – 7.27 (m, 2H), 5.32 (t, J = 5.0 Hz, 1H), 4.28 – 4.07 (m, 2H), 4.05 – 3.92 (m, 1H), 3.89 – 3.75 (m, 1H), 1.26 (t, J = 7.1 Hz, 3H). **^{13}C NMR (100 MHz, $CDCl_3$)** δ 165.71, 146.27, 139.50, 137.92, 135.19, 134.52, 132.95, 129.74, 129.27

(d, J = 32.4 Hz), 129.23, 127.45, 125.59 (q, J = 3.8 Hz), 124.43, 123.85, 118.54, 61.12, 41.69, 28.13, 14.28. **^{19}F NMR (377 MHz, $CDCl_3$)** δ -62.45. **ESI-MS: calculated [$C_{22}H_{16}BrF_3O_2S$ + H]$^+$: 481.0079, found: 481.0090.** $[\alpha]^{20}_{D}$= -57.1 (c = 0.99, CH_2Cl_2). The product was analyzed by HPLC to determine the enantiomeric excess: 94% *ee* (CHIRALPAK AD-H, hexane/*i*-PrOH =85/15, detector: 254 nm, T = 30 ℃, flow rate: 1 mL/min), t_1(minor) = 6.41min, t_2(major) = 7.08 min.

Ethyl (*R*)-8-chloro-1-phenyl-1,4-dihydrodibenzo[b,d]thiophene-2-carboxylate (6h)

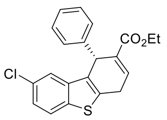

Step 1: 60 h; Step 2: 24 h (Silica gel 600 mg); Total yield: 16.7 mg (45%); **^{1}H NMR (400 MHz, $CDCl_3$)** δ 7.62 (d, J = 8.5 Hz, 1H), 7.47 (d, J = 1.9 Hz, 1H), 7.32 – 7.27 (m, 2H), 7.26 – 7.10 (m, 5H), 5.24 (t, J = 5.1 Hz, 1H), 4.25 – 4.05 (m, 2H), 4.03 – 3.89 (m, 1H), 3.87 – 3.71 (m, 1H), 1.25 (t, J = 7.1 Hz, 3H). **^{13}C NMR (100 MHz, $CDCl_3$)** δ 166.09, 142.16, 139.43, 137.37, 134.82, 133.67, 130.72, 130.54, 128.94, 128.55, 126.98, 124.55, 123.35, 121.68, 60.92, 41.98, 28.15, 14.27. **ESI-MS: calculated [$C_{21}H_{17}ClO_2S$ + H]$^+$: 369.0711, found: 369.0712.** $[\alpha]^{20}_{D}$= -109.0 (c = 0.52, CH_2Cl_2). The product was analyzed by HPLC to determine the enantiomeric excess: 97% *ee* (CHIRALPAK IA, hexane/*i*-PrOH =90/10, detector: 254 nm, T = 30 ℃, flow rate: 1 mL/min), t_1(minor) = 7.33min, t_2(major) = 8.36 min.

Tert-butyl(3R,4R)-4-(hydroxymethyl)-3-(methylcarbamoyl)-1,2,3,4-tetrahydro-9H-carbazole-9-carboxylate (7)

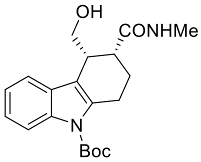

^{1}H NMR (400 MHz, $CDCl_3$) δ 8.10 (d, J = 8.1 Hz, 1H), 7.47 (d, J = 7.3 Hz, 1H), 7.25 – 7.13 (m, 2H), 6.08 (brs, 1H), 4.01 (dd, J = 12.0, 8.1 Hz, 1H), 3.79 (d, J = 10.7 Hz, 1H), 3.70 (brs, 1H), 3.41 (s, 1H), 3.27 (dd, J = 18.4, 5.8 Hz, 1H), 2.95 – 2.82 (m, 4H), 2.65 – 2.56 (m, 1H), 2.42 – 2.27 (m, 1H), 2.08 – 1.99 (m, 1H), 1.66 (s, 9H). **^{13}C NMR (100 MHz, $CDCl_3$)** δ 177.26, 150.61, 136.11, 135.70, 128.41, 123.89, 122.83, 117.66, 116.02, 115.76, 83.91, 62.69, 44.39, 38.03, 28.40, 26.72, 25.55, 22.34. **ESI-MS: calculated [$C_{20}H_{26}N_2O_4S$ + H]$^+$: 359.1965, found: 359.1966.** The product was analyzed by HPLC to determine the enantiomeric excess: 92% *ee* (CHIRALPAK IA, hexane/*i*-PrOH =95/5, detector: 254 nm, T = 30 ℃, flow rate: 1 mL/min), t_1(minor) = 17.88min, t_2(major) =19.27 min.

(3aR,10cR)-2-methyl-1,3a,4,5,6,10c-hexahydropyrrolo[3,4-c]carbazol-3(2H)-one (8)

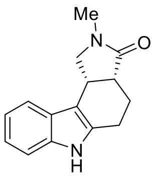

^{1}H NMR (400 MHz, $CDCl_3$) δ 8.39 (brs, 1H), 7.43 (d, J = 7.6 Hz, 1H), 7.30 (d, J = 7.8 Hz, 1H), 7.12 (m, 2H), 4.62 – 4.36 (m, 2H), 3.89 – 3.76 (m, 1H), 3.22 – 3.08 (m, 1H), 2.96 (s, 3H), 2.92 – 2.80 (m, 1H), 2.70 – 2.57 (m, 1H), 2.41 – 2.27 (m, 1H), 2.10 – 1.95 (m, 1H). **^{13}C NMR (100 MHz, $CDCl_3$)** δ 165.58, 136.06, 135.49, 126.71, 121.58, 119.61, 117.60, 111.03, 107.64, 73.59, 39.37, 34.85, 34.12, 22.31, 19.60. **ESI-MS: calculated [$C_{15}H_{16}N_2O$ + H]$^+$: 235.1335, found: 235.1331**. The product was analyzed by HPLC to determine the enantiomeric excess: 92% *ee* (CHIRALPAK

AD-H, hexane/*i*-PrOH =90/10, detector: 254 nm, T = 30 ℃, flow rate: 1 mL/min), t_1(minor) = 7.48min, t_2(major) =8.71 min.

(3aR,10cR)-2-methyl-1,2,3,3a,4,5,6,10c-octahydropyrrolo[3,4-c]carbazole (9)

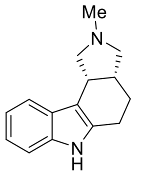

^{1}H NMR (400 MHz, DMSO) δ 10.66 (s, 1H), 7.40 (d, *J* = 7.6 Hz, 1H), 7.23 (d, *J* = 7.8 Hz, 1H), 7.01 – 6.86 (m, 2H), 3.62 – 3.52 (m, 2H), 3.25 – 3.14 (m, 1H), 2.76 – 2.58 (m, 4H), 2.31 (s, 3H), 2.17 – 2.04 (m, 1H), 1.83 – 1.65 (m, 2H). **^{13}C NMR (100 MHz, DMSO)** δ 136.10, 135.00, 126.92, 120.01, 118.10, 117.29, 110.67, 109.71, 61.79, 54.49, 37.08, 35.72, 23.31, 22.69. **ESI-MS: calculated [$C_{15}H_{18}N_2$ + H]$^+$: 227.1543, found:227.1541.** The product was analyzed by HPLC to determine the enantiomeric excess: 92% *ee* (CHIRALPAK AS-H, hexane/*i*-PrOH =70/30, detector: 254 nm, T = 30 ℃, flow rate: 1 mL/min), t_1(major) = 5.49min, t_2(minor) =9.67 min.

9-(Tert-butyl) 3-ethyl (4*R*)-4a-nitro-4-phenyl-1,4,4a,9a-tetrahydro-9H-carbazole-3,9-dicarboxylate (10)

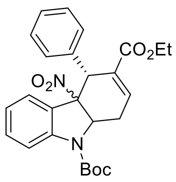

Total yield: 34.6 mg (74%); **^{1}H NMR (400 MHz, Acetone)** δ 7.92 – 7.49 (m, 2H), 7.44 (t, *J* = 7.7 Hz, 1H), 7.40 – 7.26 (m, 5H), 7.19 – 7.12 (m, 2H), 5.86 (d, *J* = 8.5 Hz, 1H), 5.45 (s, 1H), 4.16 – 4.03 (m, 2H), 3.74 – 3.49 (m, 1H), 2.70 (d, *J* = 18.6 Hz, 1H), 1.61 (s, 9H), 1.17 (t, *J* = 7.4 Hz, 3H). **^{13}C NMR (100 MHz, Acetone)** δ 171.38, 165.60, 140.51, 138.62, 133.33, 131.59, 130.08, 129.90, 129.30, 126.01, 124.73, 116.84, 62.03, 61.02, 59.69, 45.78, 34.27, 28.94, 21.33, 14.98, 14.81.**ESI-MS: calculated [$C_{26}H_{28}N_2O_6$ + Na]$^+$: 487.1845, found: 487.1869.** $[\alpha]^{20}_D$= -246.5 (c = 0.45, CH_2Cl_2). The product was analyzed by HPLC to determine the enantiomeric excess: 94% *ee* (CHIRALPAK IA, hexane/*i*-PrOH =99/1, detector: 254 nm, T = 30 ℃, flow rate: 1 mL/min), t_1(major) = 12.93 min, t_2(minor) =19.06 min.

Supplemental References

1. Cheng, Q., Zhang, F., Cai, Y., Guo, Y.-L., and You, S.-L. (2018). Stereodivergent synthesis of tetrahydrofuroindoles through Pd-catalyzed asymmetric dearomative formal [3+2] cycloaddition. Angew. Chem. Int. Ed. *57*, 2134-2138.
2. Cheng, Q., Zhang, H.-J., Yue, W.-J., You, S.-L. (2017). Palladium-catalyzed highly stereoselective dearomative [3 + 2] cycloaddition of nitrobenzofurans. Chem *3*, 428-436.
3. Tran, Y. S., and Kwon, O. (2007). Phosphine-catalyzed [4 + 2] annulation: synthesis of cyclohexenes. J. Am. Chem. Soc. *129*, 12632-12633.
